# On-Cycle Enforcement in Water: Trimetallic Assemblies Preventing Pd Black Formation While Accelerating Selective Suzuki–Miyaura Coupling without Olefin Interference

**DOI:** 10.1021/jacsau.6c00796

**Published:** 2026-07-31

**Authors:** Sudripet Sharma, Uyen Duong, Maarten Nachtegaal, Adam H. Clark, Ramesh Hiralal Choudhary, Jacek B. Jasinski, Wilfried Braje, Sachin Handa

**Affiliations:** † Department of Chemistry, 5170 University of Louisville, Louisville, Kentucky 40292, United States; ‡ Materials Characterization, Conn Center for Renewable Energy Research, 5170 University of Louisville, Louisville, Kentucky 40292, United States; § Department of Chemistry, 14716 University of Missouri, Columbia, Missouri 65211, United States; ∥ Center for Photon Science, 28498 PSI, Villigen CH-5232, Switzerland; ⊥ Center for Energy and Environmental Sciences, 28498 PSI, Villigen CH-5232, Switzerland; # Department of Chemistry, ETH Zürich, Zürich CH-8093, Switzerland; ¶ Small Molecule Therapeutics & Platform Technologies, 385234 AbbVie Deutschland GmbH & Co. KG, Ludwigshafen 67061, Germany

**Keywords:** nanoparticle, Pd–Cu–Mn trimetallic ensemble, micellar catalysis, selective Suzuki–Miyaura coupling, redox buffering, catalyst longevity

## Abstract

Palladium-catalyzed cross-couplings are frequently limited by off-cycle deactivation pathways, particularly phosphine deligation followed by aggregation of Pd(0) to catalytically inactive palladium black. We report an “on-cycle enforcement” strategy that employs a cooperative, nonalloyed trimetallic assembly—Pd(0), Cu­(I), and Mn­(III)—confined within PS-750-M/HPMC micelles in water. In this architecture, Pd mediates the canonical oxidative addition and reductive elimination steps, Cu­(I) accelerates transmetalation via a Cu-nucleophile intermediate, and Mn­(II/III) serves as a redox buffer that likely continuously reoxidizes off-cycle Pd(0) to micelle-soluble Pd­(II), followed by its religation and reduction, thereby preventing Pd black formation. Spectroscopic analysis—transmission electron microscopy, scanning transmission electron microscopy–high-angle annular dark-field imaging, energy-dispersive X-ray spectroscopy, X-ray photoelectron spectroscopy, and X-ray absorption spectroscopy—reveals that the three metals remain spatially separated in the assembly yet electronically complementary within a phosphorus-rich matrix, enabling persistent catalytic activity. The resulting platform supports rapid, selective C–C couplings at 45 °C with broad substrate compatibility, including heteroarenes and terminal olefin—containing partners, without olefin interference. The catalyst displays exceptional longevity, minimal metal leaching (<1 ppm), and robust scalability, with multicycle reactions and gram-scale processes proceeding efficiently even after extended catalyst storage. These findings establish redox-buffered, compartmentalized trimetallic assemblies as a general solution to enforcing on-cycle Pd catalysis in water.

## Graphical abstract

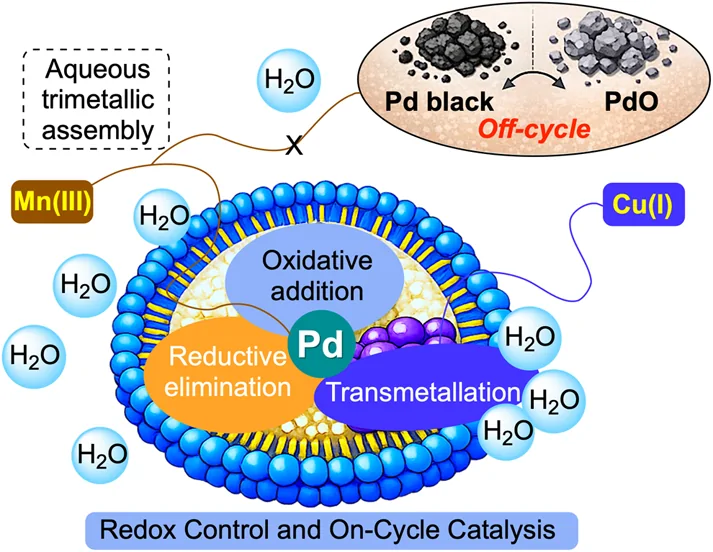

## Introduction

Palladium (Pd)-catalyzed cross-couplings have transformed organic synthesis.^1–5^ The general catalytic cycle operates via oxidative addition, base-assisted nucleophile activation/transmetalation, and reductive elimination. However, both the ligated homogeneous and heterogeneous Pd catalysts often become off-cycle, initiated by phosphine deligation, releasing metallic Pd(0), which can either aggregate into Pd black (aggregated elemental Pd formed by decomposition or clustering of Pd species) or convert to PdO.^6,7^ Both Pd black and PdO are in situ-generated catalytically inactive species,^8–10^ thus necessitating higher catalyst or specialized ligand loadings to enable successful catalytic transformations. Recent studies challenge the long-standing assumption that some heterogeneous surface-ligated Pd species are often off-cycle catalytically dead, revealing unexpected catalytic competence under certain conditions.^11–13^ Emerging mechanistic work shows that ligated Pd nanoparticles (NPs) can directly engage in key elementary steps: oxidative addition at NP edges is kinetically facile and thermodynamically favorable, implying that heterogeneous Pd can be on-cycle, not just a reservoir of molecular Pd.^14–16^ This level of catalytic activity of NP also indicates that Pd surfaces can readily interface with complementary metal suspended in the same system, creating pathways where cooperative reagent transfer can become mechanistically relevant. In this regard, dual-metal cooperation is also observed, e.g., in Pd-catalyzed Sonogashira coupling, where Cu­(I) generates organocopper intermediates that undergo nucleophile transfer from Cu to Pd.^17,18^ Building on these cooperative surface-level interactions, a compartmentalized reaction environment can further modulate catalyst behavior by organizing reactive species and enhancing the performance of nanoparticle or multimetallic assemblies. Despite significant progress in these areas, including catalyst engineering, there remains no general strategy that actively and continuously returns off-cycle Pd to the productive manifold without bespoke ligand sets.

In micellar catalysis, the formation of off-cycle Pd species can be prevented by controlling ligand loss while promoting a potentially rate-limiting, medium-sensitive transmetalation step, and maintaining catalyst integrity without leaching or uncontrolled aggregation.^19,20^ In this regard, we hypothesize that a nonalloyed, trimetallic assembly containing a mixture of Pd(0), Cu­(I), and Mn­(II/III), co-localized within PS-750-M micelles in water, can likely enforce on-cycle catalysis by combining task partitioning (e.g., efficient transmetalation and selective oxidative addition) with redox buffering.^21–23^ We design our catalyst system so that Pd conducts oxidative addition and reductive elimination at accessible sites (on-cycle activity), Cu­(I) accelerates transmetalation via Cu–organic intermediates that deliver the nucleophile to Pd, and Mn­(II/III) acts as a redox buffer in water (Figure 1). Upon phosphine deligation, Mn­(II/III) oxidizes off-cycle Pd(0) to micelle-soluble Pd­(II), promoting instant religation; subsequent in-cycle steps reduce Pd­(II) back to Pd(0). For redox buffering, we chose Mn (Mn­(II/III)) due to its redox flexibility.

**Figure 1:**
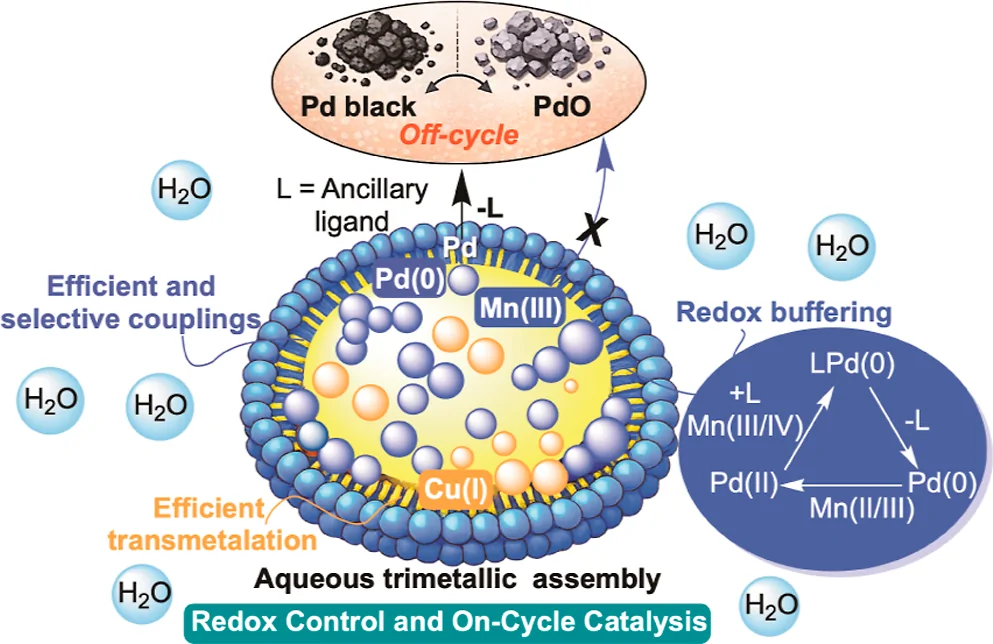
“On-cycle” palladium: Trimetallic system for redox buffering of Pd(0) by Mn­(II/III) and enhanced transmetalation by Cu­(I).

## Results and Discussion

Guided by our working hypothesis, we synthesized a trimetallic catalyst system containing Pd(0), Cu­(I), and Mn­(III) ligated by SPhos. As shown in Figure 1, the assembly of the multimetallic construct required combining Pd­(OAc)_2_, Cu­(CH_3_CN)_4_PF_6_, Mn­(OAc)_2_, and SPhos in the molar ratios 1:2:6:4, respectively. Subsequent treatment of the mixture with MeMgBr served as the reductant and initiator for the formation of trimetallic assemblies. Quenching with an aqueous mixture of 3 wt % PS-750-M and 3 wt % HPMC (4:1) generated a well-dispersed trimetallic catalyst system where none of the three metals have direct contact with each other, as supported by spectroscopic analysis (vide infra). The inclusion of HPMC was crucial for maintaining the colloidal stability and uniform dispersion during catalyst isolation and storage.

Structural analysis of the trimetallic assemblies revealed that the reaction medium plays a decisive role in directing catalyst morphology, homogeneity, and elemental distribution. When the trimetallic catalyst was co-assembled in the mixed HPMC/PS-750-M environment, high-resolution transmission electron microscopy (HRTEM) (Figure 2A,B) showed uniformly dispersed, well-defined ultrasmall assemblies. The corresponding scanning transmission electron microscopy-high-angle annular dark-field (STEM-HAADF) imaging and its EDX mapping (Figure 2C,D) confirmed a homogeneous distribution of Mn, Pd, and Cu throughout the assembly’s volume, although Cu is more buried in the interior, showing a lesser amount on the surface in comparison to Pd. Notably, these distinct ultrasmall assemblies were observed only with mixed-HPMC/PS-750-M confinement, likely due to synergistic coordination interactions that stabilize multiple metal centers simultaneously.

**Figure 2:**
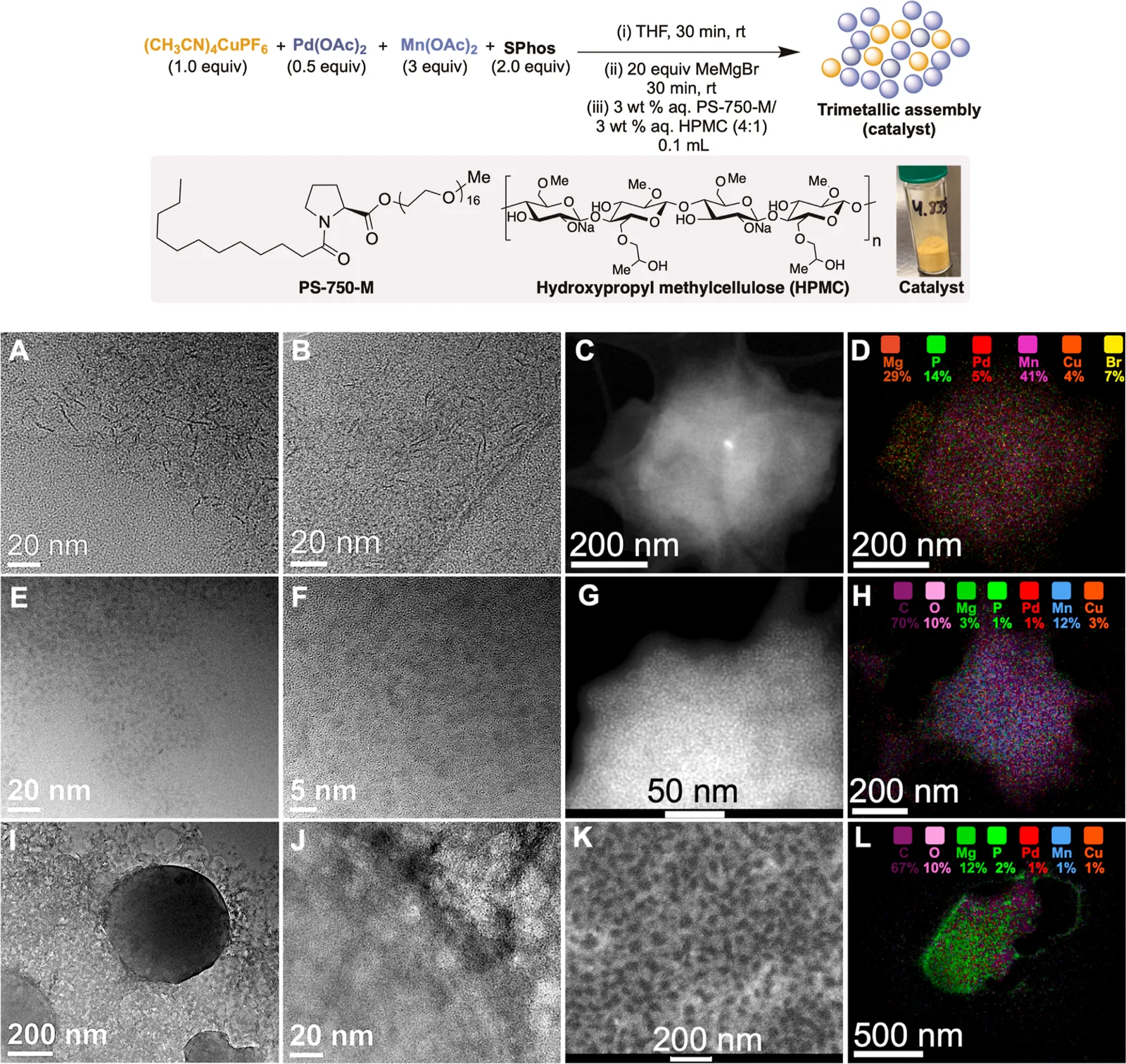
Synthesis and structural characterization of the trimetallic ensemble (i) synthesized in aqueous HPMC/PS-750-M mixed media, (ii) exclusively in aqueous PS-750-M, and (iii) exclusively in aqueous HPMC. (A–D) Trimetallic assemblies synthesized in the HPMC/PS-750-M mixture: (A,B) high-resolution transmission electron microscopy (HRTEM) images; (C) scanning transmission electron microscopy-high-angle annular dark-field (STEM-HAADF) image; and (D) energy-dispersive X-ray spectroscopy (EDX) analysis. (E–H) Trimetallic ensemble synthesized only in aqueous PS-750-M: (E,F) HRTEM images; (G) STEM-HAADF image; and (H) EDX analysis. (I–L) Trimetallic ensemble synthesized only in aqueous HPMC: (I,J) HRTEM images; (K) STEM-HAADF image; and (L) EDX analysis.

In contrast, the corresponding trimetallic system synthesized using only aqueous PS-750-M, in the absence of HPMC exhibited markedly different structural features, that is, a nanoparticle-like structure. HRTEM images (Figure 2E,F) revealed relatively larger, less uniform aggregates compared to those synthesized in mixed media. STEM-HAADF imaging (Figure 2G) showed that the particles are mostly covered with organic material, PS-750-M, which was used in this case. EDX analysis (Figure 2H) indicated less Pd at the surface in contrast to trimetallic assemblies achieved in the HPMC/PS-750-M mix that may be responsible for less effective catalysis in the absence of HPMC. These observations indicate that trimetallic assemblies are controlled by HPMC; without it, kinetically driven particles form.

Synthesis exclusively in aqueous HPMC produced yet another morphology, with relatively network-like structures (Figure 2I,J). The STEM-HAADF imaging (Figure 2K) exhibited diffuse contrast, consistent with poorly ordered metal–polymer (HPMC) composites. Correspondingly, EDX mapping (Figure 2L) revealed Pd, Cu, and Mn accumulation embedded within an HPMC-rich matrix. This likely indicates strong HPMC–metal interactions, which reduce catalysis efficiency (see Supporting Information, Figure S3, pages S22 and S23). Thus, these results collectively demonstrate that the balance between HPMC coordination strength and the confinement environment dictates the structural evolution required for catalyst efficiency, i.e., smaller trimetallic assemblies under micellar confinement.

X-ray photoelectron spectroscopy (XPS) of the Pd–Mn–Cu trimetallic platform reveals Cu predominantly in a low-valent state, i.e., Cu­(I), manganese (Mn) as both Mn­(II) and Mn­(III), and Pd as a mixture of Pd(0) and Pd­(II) species (Figure 3). The high-resolution Cu 2p spectrum displays a sharp Cu 2p_3/2_ feature at 932.5 eV, with its spin–orbit partner, Cu 2p_1/2_, at higher binding energy, and no discernible shakeup satellites in the 940–945 eV region, consistent with Cu(0)/Cu­(I) rather than Cu­(II) (Figure 3A). The Mn 2p region shows a Mn 2p_3/2_ component at ca. 641.3 eV accompanied by a characteristic shakeup feature near 645–646 eV, a fingerprint of Mn­(II) (e.g., MnO-like environments). The breadth of the spectrum also suggests a contribution from higher-valent Mn (Mn­(III)/Mn­(IV)), but Mn­(II) clearly dominates under the preparation/handling conditions (Figure 3B). In the Pd 3d region, deconvolution reveals two Pd 3d_5/2_ components at ca. 335.5 and 336.9 eV, corresponding to Pd(0) and Pd­(II), respectively, along with their related 3d_3/2_ partners at higher binding energy (Figure 3C). The oxidized Pd fraction is greater than the metallic component, plausibly arising from air exposure of catalyst clusters.

**Figure 3:**
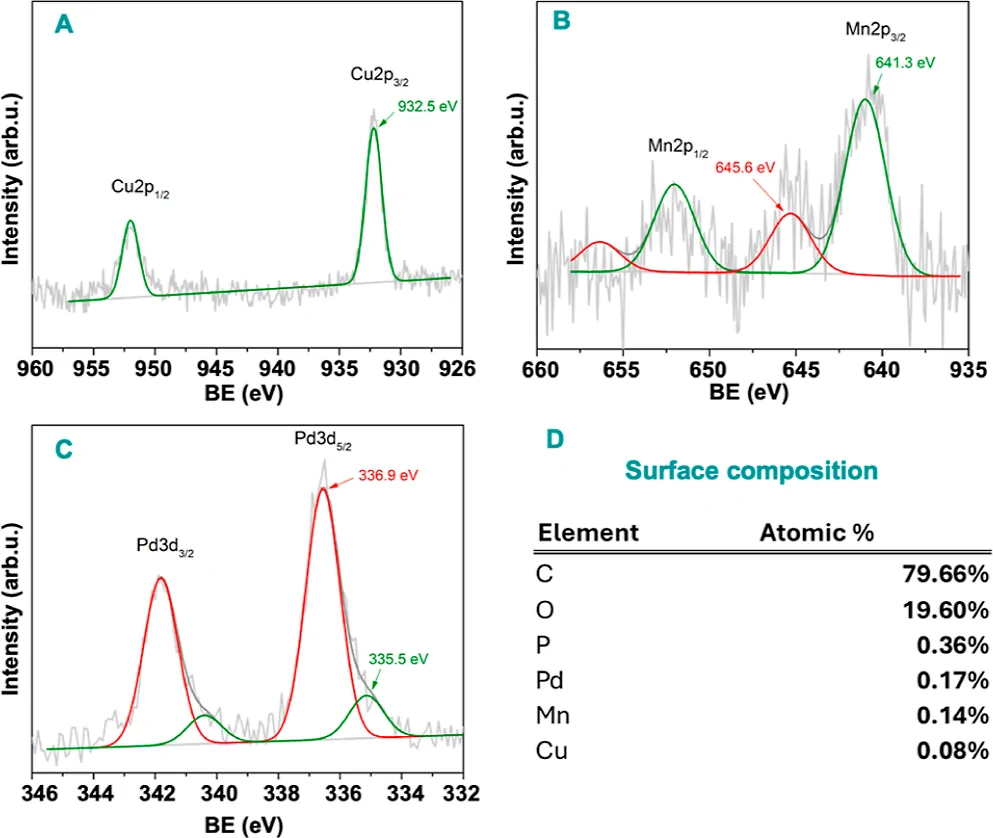
X-ray photoelectron spectroscopy analysis of Pd–Mn–Cu trimetallic assemblies for (A) copper, (B) manganese, (C) palladium, and (D) their surface composition.

A survey spectrum composition yields C 79.66%, O 19.60%, and a low nominal surface loading of the metals (Pd 0.17%, Mn 0.14%, and Cu 0.08%) (Figure 3D). Although the trimetallic assemblies contain more Cu and Mn, the survey spectrum showing more Pd than Mn and Cu reveals a nonuniform distribution of Pd across the trimetallic assemblies or MnO clusters. The strong C/O signals are consistent with a PS-750-M capping layer and surface support from HPMC. Thus, the XPS data indicate that these metal clusters or assemblies contain Cu­(I), Mn­(II)/(III), and Pd(0), all of which are available for efficient catalysis.

To elucidate the local coordination environments, metal–metal interactions, and oxidation states of the three metal centers within the trimetallic assemblies, we conducted X-ray absorption spectroscopy (XAS) at the Mn, Cu, and Pd K-edges (Figure 4). The Mn K-edge X-ray absorption near-edge structure (XANES) spectra (Figure 4A) exhibit an edge position falling between MnO and Mn_2_O_3_, indicating a mixed-valent Mn­(II/III) environment, which is in agreement with XPS data. This electronic structure is consistent with the redox-active role of Mn during catalyst formation and suggests partial oxidation during the formation of trimetallic catalyst assemblies. Extended X-ray absorption fine structure (EXAFS) analysis indicates a similar local environment to Mn_2_O_3_ with a distorted Mn–O consistent with a Jahn–Teller active d^4^ center (Mn–O at 1.93, 2.18 Å). A weak Mn–Mn interaction at 3.21 Å (coordination number ≈ 2.6) conforms with the Mn_2_O_3_ structure and suggests the presence of small, highly distorted Mn clusters rather than extended oxide-like domains. An additional coordination is found directly between Mn and P at a distance of 2.37 Å (coordination number 0.5), suggesting that a portion of the Mn interacts with SPhos ligands.

**Figure 4:**
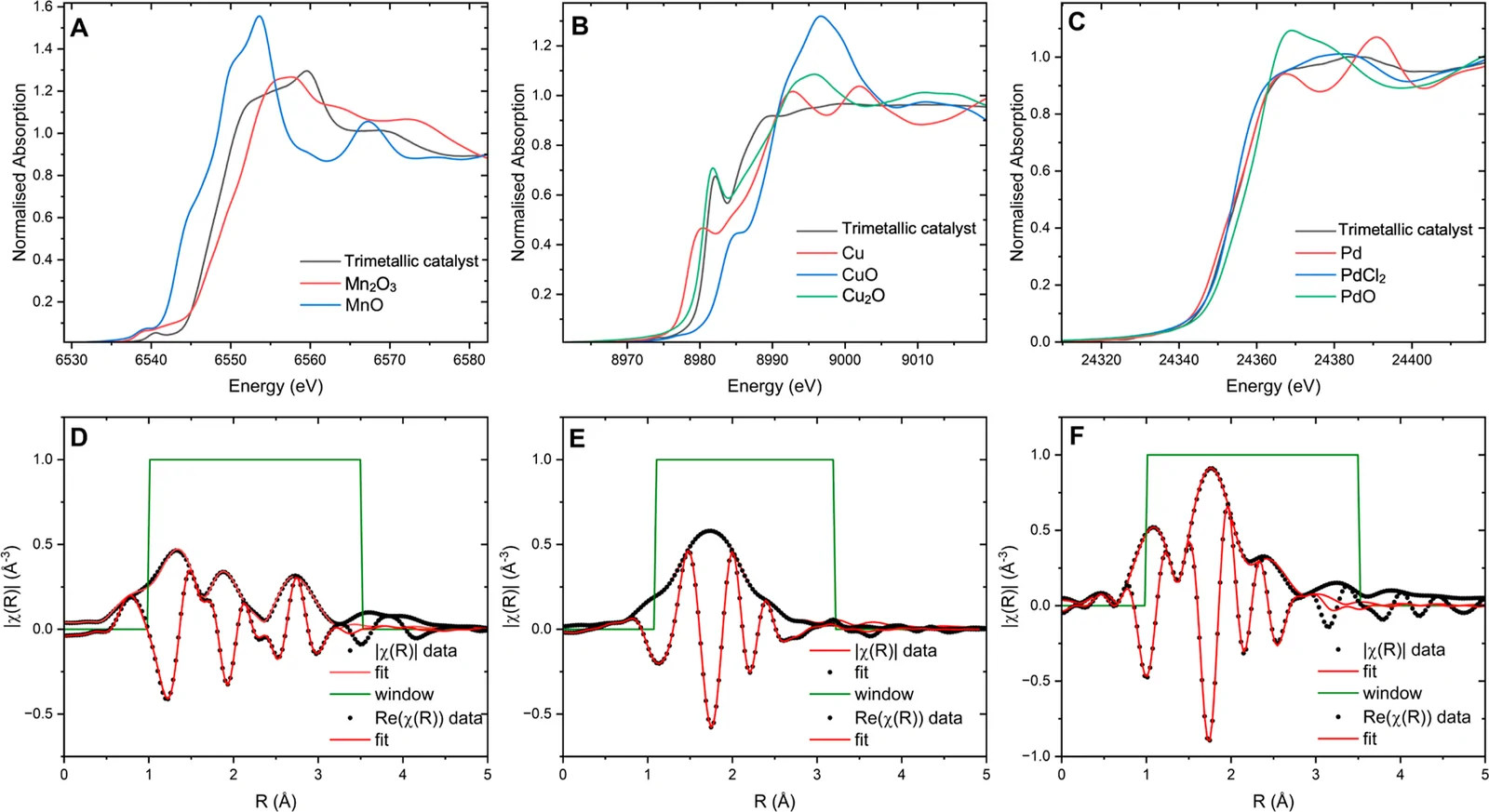
X-ray absorption spectroscopy analysis of trimetallic catalyst (trimetallic assemblies in PS-750-M/HPMC). XANES spectra for the Mn (A), Cu (B), and Pd (C) K-edges. FT-EXAFS spectra with corresponding fits for Mn (D), Cu (E), and Pd (F).

Cu K-edge XANES data identify Cu as primarily Cu­(I), with an edge energy consistent with Cu_2_O and the appearance of the 1s–4p transition at ∼8980 eV. No Cu­(II) contribution was detected (Figure 4B). EXAFS fitting indicates coordination with a light atom, with a Cu–O/N distance of 1.95 Å (likely with PS-750-M/HPMC) and, similar to Mn, an interaction directly with the P atom of SPhos at 2.23 Å (Figure 4E; for details, see Supporting Information, page S14). The very weak Cu–Cu metal-like scattering at 2.49 Å is additionally resolved (coordination number 1.0), suggesting that Cu exists primarily as isolated Cu­(I) sites within the matrix; however, small clusters cannot be entirely excluded.

Pd K-edge XANES (Figure 4C) indicates a spectrum with a line shape reminiscent of PdCl_2_ more than of PdO and notably different from the metallic reference. The EXAFS of the trimetallic catalyst displayed a strong Pd–P feature at 2.32 Å with a coordination number of ca. 1.8. A minor Pd–Pd interaction at 2.93 Å (coordination number ≈ 1.6), which together is consistent with the formation of low coordinate Pd–P structures (Figure 4C,F; for details, see Supporting Information, page S14).

Collectively, the Mn, Cu, and Pd XAS data describe an architecture composed of heterovalents, nonalloyed metal centers embedded in a phosphorus-rich SPhos matrix. Mn forms ultrasmall distorted Mn­(III)–P/O clusters, Cu primarily remains as isolated Cu­(I) sites, and Pd exists primarily as isolated low-coordinate Pd phosphide. The absence of Mn–Cu, Cu–Pd, or Mn–Pd scattering confirms spatial separation of the metals, with phosphorus serving as a key structural linker for Mn and Pd.

A ^31^P NMR control study was also conducted to elucidate the ligation behavior in the trimetallic ensemble (see Supporting Information, Figure S2, page S17). Based on the control ^31^P NMR data shown, the trimetallic ensemble generated after treatment of the preassembled SPhos–Pd–Cu–Mn system with MgMeBr appears to be a new, well-defined phosphorus-containing species that is electronically distinct from any of the individual bimetallic or monometallic complexes. Free SPhos shows a resonance near −9.68 ppm, while the independently prepared SPhos–Pd, SPhos–Cu, and SPhos–Mn complexes resonate at 48.71, 18.33, and approximately −9.77 ppm, respectively. The mixture containing all three metals prior to Grignard addition exhibits two major signals at 46.80 and 12.78 ppm, consistent with the simultaneous presence of Pd- and Cu-bound phosphine species. Upon addition of MgMeBr, however, these resonances disappear and are replaced by a new set of closely spaced signals centered at ∼59–60 ppm (59.97, 59.48, and 58.93 ppm). The substantial downfield shift relative to the Pd complex alone indicates a significant change in the electronic environment at phosphorus, suggesting formation of structurally unique Pd-centered species. The appearance of a highly diagnostic cluster of resonances, rather than a broad distribution of signals, supports the formation of a discrete trimetallic assembly rather than a simple mixture of independent Pd, Cu, and Mn complexes. The small multiplicity around 59–60 ppm may arise from closely related coordination environments. Collectively, these observations are consistent with the generation of a cooperative Pd–Cu–Mn trimetallic ensemble.

To evaluate the catalytic efficiency, longevity, and selectivity of the trimetallic catalyst system, we examined its performance at a loading possessing only 0.5 mol % Pd (Scheme 1A). The coupling of 4-bromostyrene **1** and naphthylboronic acid **2** was carried out using K_3_PO_4_ as the base in the 4:1 mixture of aqueous 3 wt % PS-750-M and 3 wt % HPMC. As anticipated, the reaction reached full conversion within 10 min. To probe whether the catalyst remained active without forming Pd black and losing activity, the reaction mixture was exposed to ambient temperature for 30 min before a second portion of coupling partners and base was added (for details, see Supporting Information, page S18). Again, complete conversion was achieved within 10 min. The reaction mixture was again kept in a dormant state for 12 h without isolating the product. Next, the reaction mixture was diluted to 0.25 M with aqueous PS-750-M, and a third portion of substrates and base was added; the reaction once more proceeded to completion—this time within 45 min. The slower rate is attributed to a low global concentration, but this verified the catalyst’s longevity. These observations strongly support our hypothesis that Mn-mediated redox buffering enforces the “on-cycle” state of Pd, thereby preventing deactivation and enabling long catalyst lifetime. Notably, the catalyst exhibited excellent efficiency and chemoselectivity throughout the sequence, with no competitive Heck-type reaction observed at the styrenyl position. The overall isolated yield of product **3** across the three cycles was 90% (Scheme 1A).

**Scheme 1:**
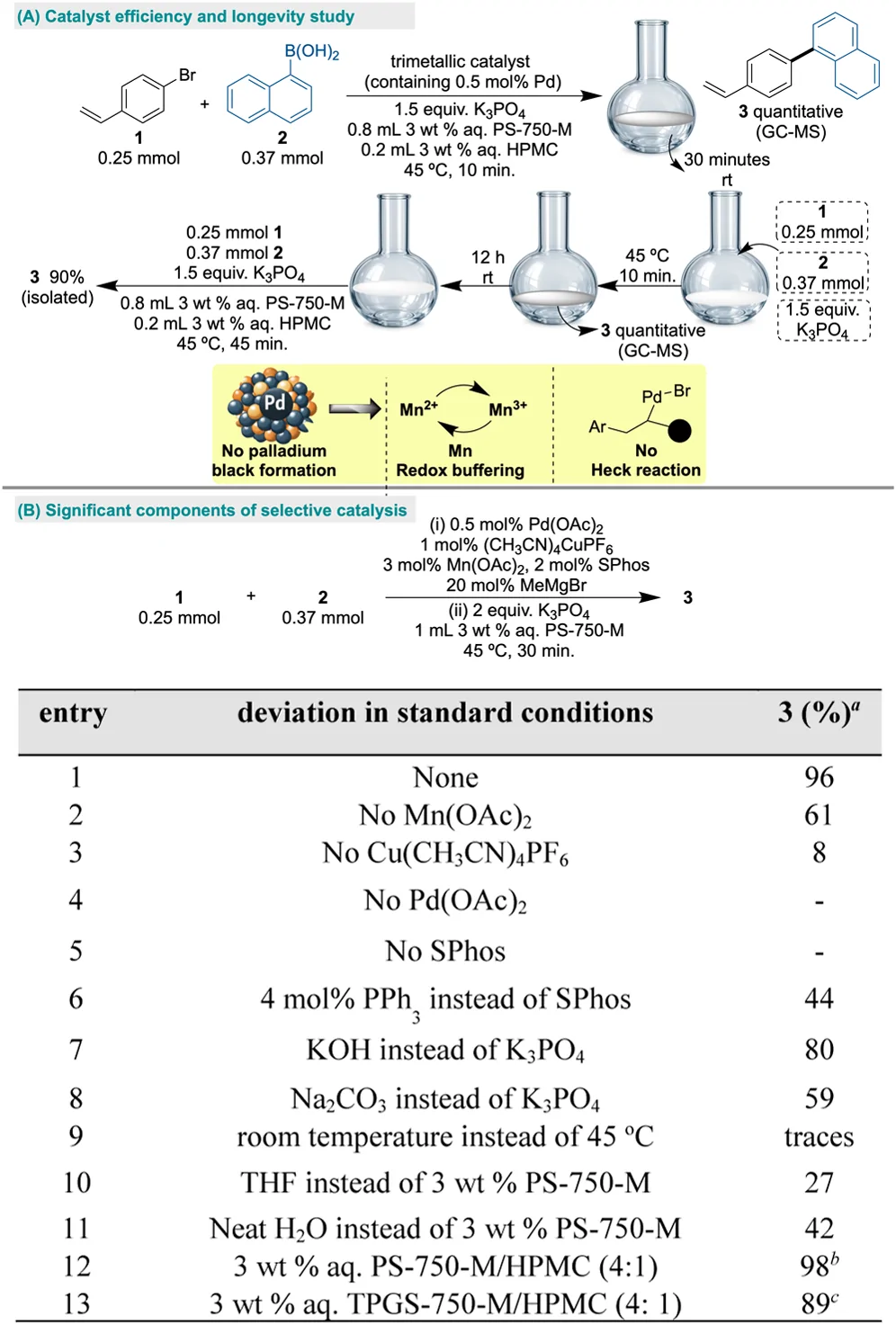
Essential Components of a Catalyst for Longevity and Efficiency

(A) Evaluation of catalyst efficiency and longevity for the Mn-buffered Pd-catalyzed selective Suzuki–Miyaura coupling. (B) Control experiments identifying components essential for selective catalysis. *^a^* Yields based on GC–MS conversions; ^*b*^reaction time was 10 min. ^*c*^Isolated yield. 0.1 mL of THF was added as a cosolvent in all reactions.

Next, we evaluated the role of each metallic component in achieving efficient, selective catalysis using in situ-generated trimetallic assemblies and their use in 3 wt % aq PS-750-M (Scheme 1B, entry 1). Removal of Mn from the catalyst resulted in only 61% yield of product **3**, and the catalyst became inactive after approximately 30 min (entry 2). Similarly, omission of Cu led to a dramatic loss of activity, providing only 8% of product **3** (entry 3), whereas exclusion of Pd resulted in no detectable product formation (entry 4). These observations collectively support a division of labor in which Pd is crucial for the fundamental elementary steps of cross-coupling, Cu promotes what is likely the rate-limiting transmetalation step, and Mn helps maintain Pd in the active catalytic cycle. The ligand SPhos was crucial for the catalytic activity, and no reaction was observed when SPhos was omitted (entry 5). The reaction was not as effective when SPhos was replaced with PPh_3_, and only 44% of product **3** was observed (entry 6). Among the bases examined, K_3_PO_4_ outperformed both KOH and Na_2_CO_3_ (entries 7, 8). The strong basicity of KOH likely disrupts trimetallic assemblies, while the comparatively weak basicity of Na_2_CO_3_ renders it ineffective for transmetalation. Mild heating was also required to enable efficient exchange within the compartmentalized system; at room temperature, only trace amounts of product were observed (entry 9). Reactions conducted in neat THF or neat water were ineffective (entries 10, 11). However, the reaction time drastically dropped to 10 min when a 4:1 ratio of aq PS-750-M and HPMC was employed as a solvent system, providing 98% of product **3** (entry 12). Further, replacing the PS-750-M surfactant with TPGS-750-M resulted in a slightly lower conversion to product **3**; likewise, the TritonX100/HPMC combination afforded a slightly lower conversion (entry 13 and Table S11 on page S8 of the Supporting Information). This underscores the essential and synergistic roles of both PS-750-M and HPMC in enabling productive catalysis.

The trimetallic catalyst also proved broadly effective across a diverse range of (hetero)­aryl bromides and boronic acids, delivering selective Suzuki–Miyaura couplings under exceptionally mild conditions (≤45 °C) while leaving the terminal olefin motif fully intact (Scheme 2A, **3–34**). Electron-rich aryl bromides (**7**, **29**, **30**, **32**) reacted rapidly—often within minutes to a few hours—highlighting the accelerated oxidative addition enabled by the Pd-containing trimetallic assembly. Electron-neutral substrates (**3**, **14**, **18**, **28**) similarly furnished products in excellent yields, consistent with a catalytic system that is not strongly dependent on electronic activation. These results highlight the catalyst’s notable capacity to accommodate coupling partners known for slower oxidative addition and that require elevated temperatures.^24^ Electrophile-containing heteroaryl rings—including Indoles (**15–17**, **21**, **24**) and thiophenes (**5**, **12**, **20**)—also reacted smoothly to provide the desired cross-coupled products in good-to-excellent yields, despite their well-known tendency to poison the nanocatalyst.^25^ The compatibility of multiple heteroaromatic substrates highlights the stabilizing and protective effect of the compartmentalized matrix provided by HPMC and PS-750-M.

**Scheme 2:**
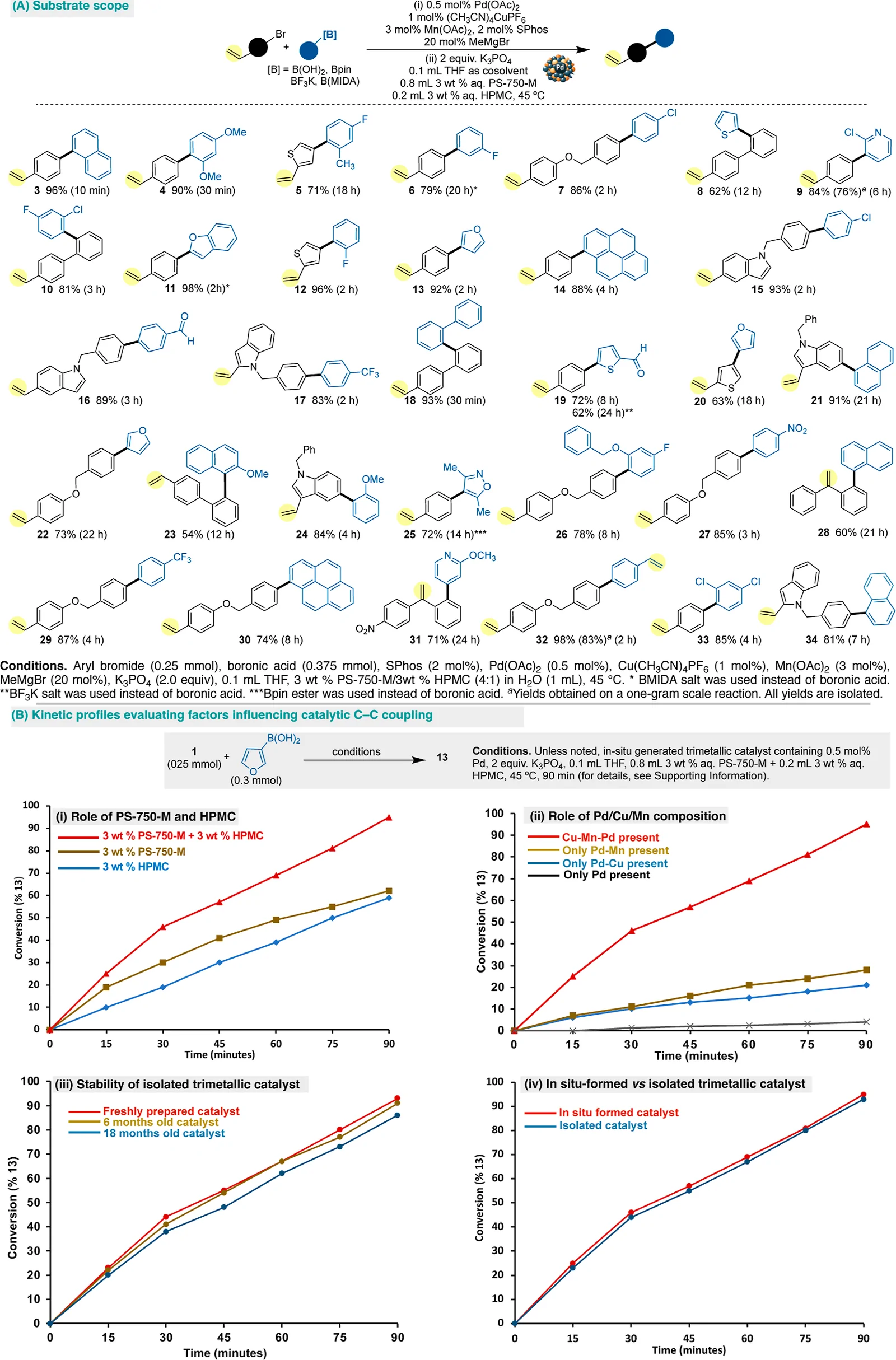
Trimetallic Catalyst Ensemble-Mediated C–C Coupling: Substrate Scope and Mechanistic Kinetic Evaluation^a^

a (A) Substrate scope. (B) Time-course profiles illustrating factors affecting reactivity mediated by trimetallic assemblies.

A broad set of boronic acid partners was likewise well tolerated. Electron-rich (**23**, **25**) and electron-poor (**6**, **7**, **9**, **10**, **12**, **15**–**17**, **19**, **20**, **27**, **29**, **33**) substrates consistently provided the corresponding biaryl products in high yields, demonstrating the catalyst system’s generality and robustness. Substrates bearing nitro (**27**), aldehyde (**16**, **19**), chloro (**7**, **9**, **10**, **15**, **33**), thiophene (**8**, **19**), pyridine (**9**, **31**), benzofuran (**11**), furan (**13**, **20**, **22**), and isoxazole (**25**) substituents all participated without noticeable loss of efficiency, further emphasizing the mildness and functional-group compatibility of the platform.

Notably, substrates containing labile halogens (**9**, **10**) did not undergo S_N_Ar or other undesired pathways. Pyrene-containing boronic acids also coupled efficiently, indicating that potential π–π interactions did not impede productive catalysis (**14**, **30**). Owing to the catalyst’s efficiency and the mild conditions, product **18** did not undergo any side reactivity or polymerization, even though it contains a reactive styrenyl group conjugated to an extended π-system. Similarly, example **32**, which contains two terminal olefins, remained intact and showed no evidence of olefin polymerization. In addition to boronic acids, alternative boron species—including BMIDA esters (**6**, **11**), BF_3_K salts (**19b**), and Bpin esters (**25**)—coupled efficiently to furnish the desired products.

Across all cases, both mono- (**3**–**27**, **29**, **30**, **33**, **34**) and di-substituted (**28**, **32**) terminal olefin functionality remained untouched, enabling direct access to a variety of styrenyl monomers relevant to polymer and materials research. Further supporting the integrity of the trimetallic architecture, inductively coupled plasma mass spectrometry (ICP–MS) analysis of representative products **3** and **18** revealed Mn and Pd levels <1 ppm in the isolated materials (see Supporting Information, page S28). This exceptionally low metal content, consistent with the outcomes of live-catalyst studies, indicates minimal catalyst degradation or leaching under reaction conditions and reinforces the stability of the integrated Pd–Cu–Mn ensemble during turnover. Taken together, these findings support a mechanistic picture in which the compartmentalized trimetallic framework enables rapid C–C bond formation while minimizing off-cycle deactivation pathways that typically limit catalytic efficiency in multimetallic systems.

The scalability of the trimetallic catalytic system was further assessed through two representative gram-scale reactions (**9** and **32**). For the synthesis of **9**, a 1 month-old catalyst was used without reactivation, and the transformation proceeded smoothly to afford the product in nearly identical yields on small and gram scales (84% and 76%, respectively), demonstrating excellent catalyst longevity and shelf-stability. Similarly, the gram-scale preparation of **32**, employing a freshly generated in situ catalyst, delivered the cross-coupled product in 83% isolated yield, confirming that catalytic efficiency is fully retained upon scale-up (for details, see Supporting Information, pages S20 and S21). Together, these results highlight the operational robustness of the trimetallic catalyst and its suitability for larger-scale synthetic applications.

To further elucidate the origin of the enhanced reactivity of the trimetallic catalyst platform, a series of comparative kinetic experiments were performed using coupling partners **1** and 3-furanylboronic acid to obtain coupling product **13** (Scheme 2B). These studies evaluated (i) the role of the PS-750-M/HPMC matrix, (ii) the individual contributions of Pd, Cu, and Mn within the trimetallic assembly, (iii) the stability of isolated trimetallic ensemble over extended storage, and (iv) potential differences between isolated versus in situ-generated catalyst.

As shown in Scheme 2B­(i), the combination of PS-750-M with HPMC produced a pronounced rate acceleration relative to either component alone. The full PS-750M/HPMC system delivered nearly quantitative conversion within 90 min, whereas reactions conducted with either PS-750 M or HPMC alone exhibited significantly slower kinetics. These results indicate that the hybrid matrix is crucial for the observed high catalytic activity, presumably by simultaneously stabilizing and dispersing the trimetallic ensemble and enabling efficient access of the substrate to the active metal sites.

Kinetic analysis using the catalyst containing single or binary metal combinations revealed a clear synergistic effect among Pd, Cu, and Mn (Scheme 2B­(ii)). The full Pd–Cu–Mn trimetallic ensemble showed dramatically higher reaction rates than Pd only, Pd/Cu, or Pd/Mn, each of which displayed substantially diminished activity. The enhanced performance of the trimetallic system suggests that effective transmetalation is enabled by Cu­(I) and redox buffering by Mn­(III), keeping the Pd in the “on cycle” while facilitating faster oxidative addition, transmetalation, and reductive elimination steps.

The isolated trimetallic catalyst was further evaluated after extended storage to assess the catalyst longevity. Freshly prepared, 6 month-old, and 18 month-old trimetallic catalyst samples all displayed virtually insignificant differences in kinetic profiles (Scheme 2B­(iii)), demonstrating the exceptional temporal stability of the trimetallic catalyst architecture. These findings are consistent with the low metal leaching observed by ICP–MS and reinforce the structural robustness of the trimetallic ensemble over long time periods.

Finally, a comparison of an in situ generated trimetallic catalyst with a preisolated and redispersed catalyst revealed closely matching kinetic profiles (Scheme 2B­(iv)). Both materials exhibited rapid, nearly superimposable rates, indicating that the isolated trimetallic catalyst reproduces the catalytic performance of the in situ formed species. This result confirms that the active catalyst corresponds to the intact trimetallic ensemble rather than a transient or dynamically evolving species formed under reaction conditions.

These kinetic studies provide strong mechanistic support for the cooperative and persistent nature of the trimetallic catalyst ensemble. The synergy arising from enhanced transmetalation and redox buffering of Pd in the Pd–Cu–Mn ensemble—combined with the stabilizing effect of the PS-750-M/HPMC matrix—enables sustained high activity, exceptional stability, and reproducible performance across both small- and gram-scale reactions.

## Conclusions

This work introduces a cooperative trimetallic (Pd–Cu–Mn) catalytic architecture that actively enforces on-cycle Pd catalysis by preventing Pd(0) aggregation and promoting sustained Suzuki–Miyaura couplings under mild aqueous micellar conditions. Through a combination of oxidative-addition-competent Pd species, transmetalation-accelerating Cu­(I) sites, and Mn­(II/III)-based redox buffering, the system likely dynamically returns off-cycle Pd(0) to Pd­(II), short-circuiting the deactivation pathways that traditionally limit Pd-catalyzed cross-couplings. Comprehensive spectroscopic analysis confirms a nonalloyed, spatially segregated, phosphorus-supported trimetallic framework that remains structurally intact and catalytically competent over extended storage and repeated use. The platform enables rapid, chemoselective C–C couplings with broad functional group tolerance, excellent olefin compatibility, and negligible metal leaching. The compatibility of this catalytic platform with gram-scale processes and the shelf-stability of the isolated catalyst further highlight its practical relevance. Overall, this trimetallic strategy could serve as a blueprint for designing multimetal catalytic systems that combine compartmentalization, redox control, and cooperative reactivity to overcome long-standing challenges in heterogeneous and hybrid Pd catalysis.

## Supplementary Material



## Notes

General information, materials synthesis, detailed analytical data, details of catalytic activity tests, GC–MS, and NMR data (PDF)
