# Supplementary material for: On-Cycle Enforcement in Water: Trimetallic Assemblies Preventing Pd Black Formation While Accelerating Selective Suzuki–Miyaura Coupling without Olefin Interference
Source: JACS Au. 2026 Jul 31;6(9):5145–53. doi: 10.1021/jacsau.6c00796 (PMC13625518; doi:10.1021/jacsau.6c00796)

On-Cycle Enforcement in Water: Trimetallic Assemblies Preventing Pd Black Formation While Accelerating Selective Suzuki-Miyaura Coupling Without Olefin Interference

Sudripet Sharma,[†#] Uyen Duong,[†#] Maarten Nachtegaal,[⊥∇^] Adam H. Clark,[⊥] Ramesh Hiralal Choudhary,[ǁ] Jacek B. Jasinski,[§] Wilfried Braje,[‡] Sachin Handa[ǁ†*]

[†]Department of Chemistry and [§]Materials Characterization, Conn Center for Renewable Energy Research, University of Louisville, Louisville, KY 40292, United States

[ǁ]Department of Chemistry, University of Missouri, Columbia, MO 65211, United States

[⊥]Center for Photon Science and [∇]Center for Energy and Environmental Sciences PSI, Villigen, CH-5232, Switzerland

[^]Department of Chemistry, ETH Zürich, CH-8093, Zürich, Switzerland

[‡] Small Molecule Therapeutics & Platform Technologies, AbbVie Deutschland GmbH & Co. KG, Ludwigshafen, Germany

[#]These authors contributed equally

*sachin.handa@missouri.edu

Supporting Information

Contents **Page**

1. General experimental details S1
2. Optimization study S2-S8
3. General procedure for trimetallic catalyst synthesis S9
4. General procedure for coupling reactions S10, S11
5. HRTEM and XPS analysis S12, S13
6. XAS analysis of Pd/Cu/Mn trimetallic catalyst S14
7. Control ^{31}P NMR experiments S15, S16
8. Catalytic efficiency and longevity study S17
9. Substrate scope S18
10. Gram scale reactions S19, S20
11. Kinetic study S21-S26
12. ICP-MS analysis S27
13. Analytical data S28-S41
14. References S42
15. NMR data S43-S77
16. HRMS data S78-S107

1. GENERAL EXPERIMENTAL DETAILS

All manipulations were carried out under an argon atmosphere in an oven-dried 4 mL glass vial containing a PTFE-coated magnetic stir bar unless otherwise noted. The solvent molarity listed in the reaction schemes is relative to the limiting reagent. All experiments were monitored by analytical thin-layer chromatography (TLC) and GC-MS. For reaction workup, solvents were removed under vacuum, and the sample was heated in a water bath at 40 °C. Columns were packed as a slurry of silica gel in hexanes.

1.1 Chemicals and supply

Chromatography. TLC plates (UV 254 indicator, aluminum-backed, 175–225 µm thickness, standard grade silica gel, 230–400 mesh) were supplied by Merck; silica gel (60 Å pore size, 230-400 mesh) was purchased from Silicycle.

Solvents. Acetone, THF, ethyl acetate, hexanes, dichloromethane, and HPLC-grade water were purchased from Fisher Scientific and used without further purification. NMR solvents were obtained from Cambridge Isotopes Laboratories; aqueous solutions of PS-750-M surfactant and HPMC were prepared in HPLC-grade water and thoroughly purged with argon before use. TPGS-750-M wax was supplied by ARC biochemical technology, and Triton X-100 was purchased from Sigma; poly(ethylene glycol) methyl ether 750 (MPEG-750-M) was supplied by Alfa-Aesar.

Reagents. Tetrakis(acetonitrile)copper(I) hexafluorophosphate, palladium (II) acetate, manganese (II) acetate, and MeMgBr (3M in THF/Et_2O) were purchased from Sigma Aldrich; commercially available boronic acid, MIDA boronate esters, and aryl halides were either supplied by Sigma-Aldrich, Combi-Blocks Inc., or Oakwood chemicals.

1.2 General instrumentation

All products were purified by flash chromatography using a manual column or Teledyne Isco CombiFlash R_f 150. GC-MS data were obtained using a Thermo Scientific Trace 1300 Gas Chromatograph coupled with a Thermo Scientific ISQ-QD Single Quadrupole Mass Spectrometer. All NMR spectra were recorded at 23 °C on Varian MR-400, Varian Unity INOVA 500, and Varian VNMRS 700 spectrometers (400, 500, and 700 MHz, respectively). Melting points were determined using a MEL-TEMP II melting point apparatus with samples in Kimble Kimex 51 capillaries (1.5-1.8 x 90 mm). Reported chemical shifts are referenced to residual solvent peaks.[1]

2. OPTIMIZATION STUDY

Table S1. Screening metals [M] for the redox buffering

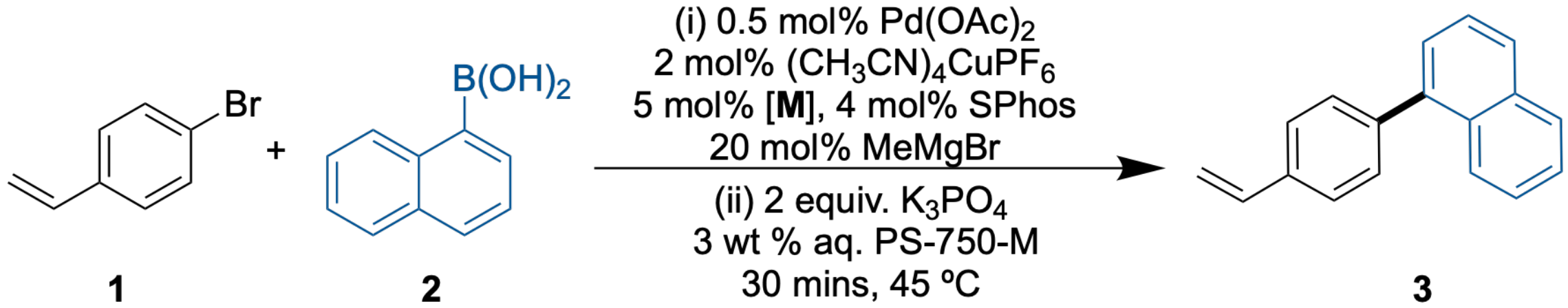

entry	[M] (5 mol%)	**3** (%)[a]
1	-	61
2	**$Mn(OAc)_2$**	**94**
3	$CoSO_4$	74
4	$CoCl_2$	78
5	$MnCl_2$	51
6	TiO_2	75
7	Ferrocene	92

Conditions: **1** (0.25 mmol), **2** (0.3 mmol), SPhos (4 mol%), $Pd(OAc)_2$ (0.5 mol%), $Cu(CH_3CN)_4PF_6$ (2 mol%), M (5 mol%), MeMgBr (20 mol%), K_3PO_4 (2.0 equiv.), 0.1 mL THF, 3 wt % PS-750-M in H_2O (1.0 mL), 45 °C, 30 minutes. [a]Yields based on GC-MS conversions.

Table S2. Screening of copper sources

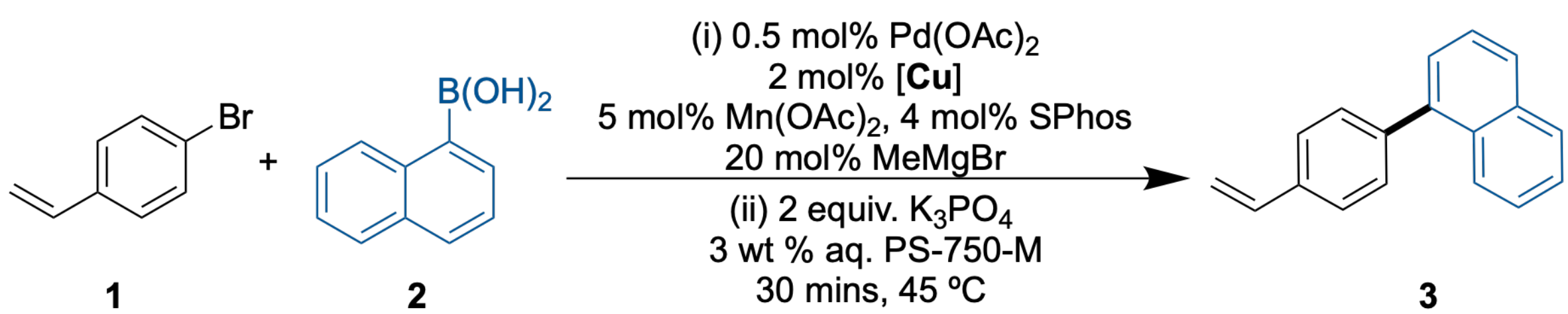

entry	[Cu] (2 mol%)	**3** (%)[a]
1	-	8
2	$Cu(OAc)_2$	65
3	CuCl	83
**4**	**$Cu(CH_3CN)_4PF_6$**	**94**

Conditions: **1** (0.25 mmol), **2** (0.3 mmol), SPhos (4 mol%), $Pd(OAc)_2$ (0.5 mol%), [Cu] (2 mol %), $Mn(OAc)_2$ (5 mol%), MeMgBr (20 mol%), K_3PO_4 (2.0 equiv.), 0.1 mL THF, 3 wt % PS-750-M in H_2O (1.0 mL), 45 °C, 30 minutes. [a]Yields based on GC-MS.

Table S3. Screening of ligands

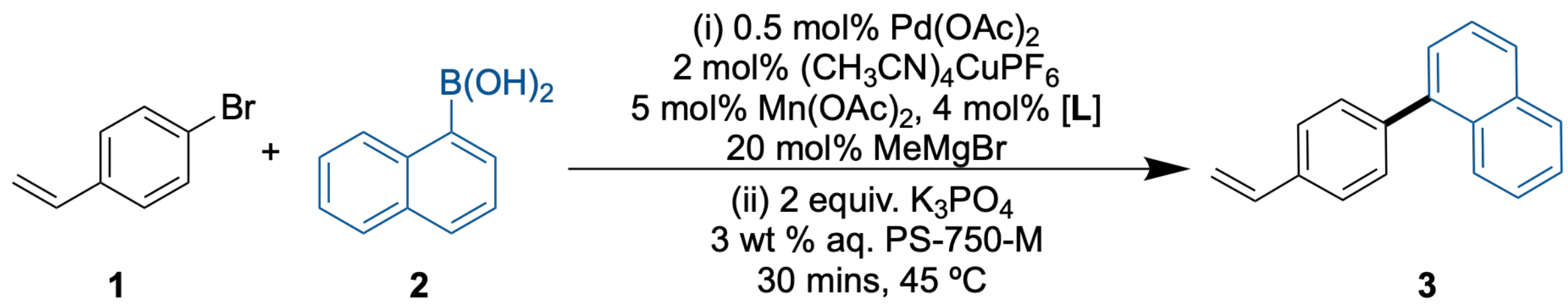

entry	ligand (L) (4 mol%)	**3** (%)[a]
1	Ph_3P	44
2	*t*-BuXPhos	54
3	DTBPF	74
4	**SPhos**	**94**
5	-	0

Conditions: **1** (0.25 mmol), **2** (0.3 mmol), ligand (4 mol%), $Pd(OAc)_2$ (0.5 mol%), $Cu(CH_3CN)_4PF_6$ (2 mol%), $Mn(OAc)_2$ (5 mol%), MeMgBr (20 mol%), K_3PO_4 (2.0 equiv.), 0.1 mL THF, 3 wt % PS-750-M in H_2O (1.0 mL), 45 °C, 30 minutes. [a]Yields based on GC-MS.

Table S4. Screening of base

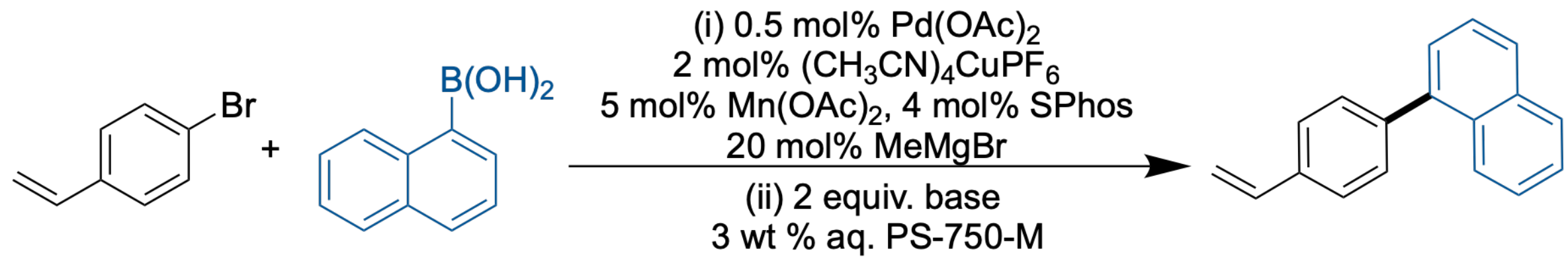

1 **2** **3**

entry	base	**3** (%)[a]
1	KOH	80
2	NaOH	86
3	Na_2CO_3	59
4	Cs_2CO_3	42
5	LiO*t*Bu	71
6	Et_3N	85
7	DIPEA	87
8	NaO*t*Bu	85
**9**	**K_3PO_4**	**94**

Conditions: **1** (0.25 mmol), **2** (0.3 mmol), SPhos (4 mol%), $Pd(OAc)_2$ (0.5 mol%), $Cu(CH_3CN)_4PF_6$ (2 mol%), $Mn(OAc)_2$ (5 mol%), MeMgBr (20 mol%), base (2.0 equiv.), 0.1 mL THF, 3 wt % PS-750-M in H_2O (1.0 mL), 45 °C, 30 minutes. [a]Yields based on GC-MS conversions.

Table S5. Optimization of Pd loading

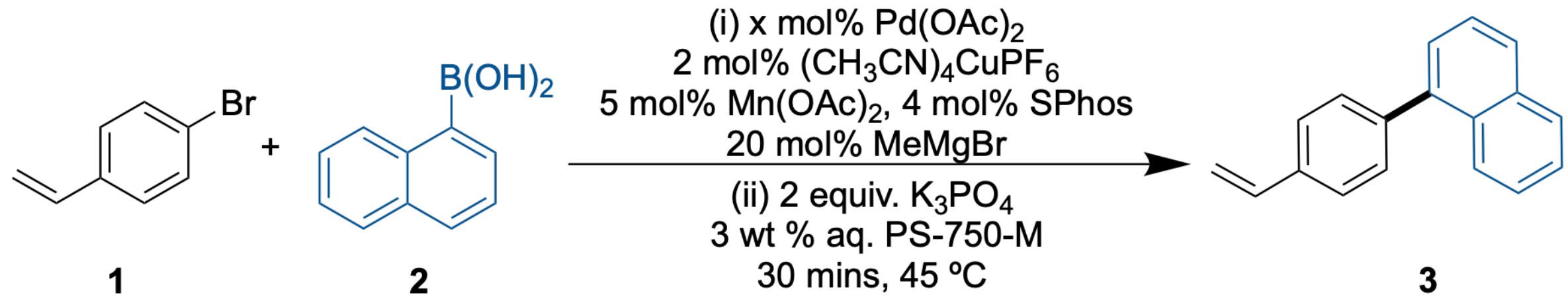

entry	$Pd(OAc)_2$ (mol%)	**3** (%)[a]
1	0.1	65
2	0.3	83
**3**	**0.5**	**96**
4	1	81

[a]**Conditions**: **1** (0.25 mmol), **2** (0.3 mmol), SPhos (4 mol%), $Pd(OAc)_2$, $Cu(CH_3CN)_4PF_6$ (2 mol%), $Mn(OAc)_2$ (5 mol%), MeMgBr (20 mol%), K_3PO_4 (2.0 equiv.), 0.1 mL THF, 3 wt % PS-750-M in H_2O (1.0 mL), 45 °C, 30 minutes. [a]Yields based on GC-MS analysis.

Table S6. Optimization of $Mn(OAc)_2$ loading

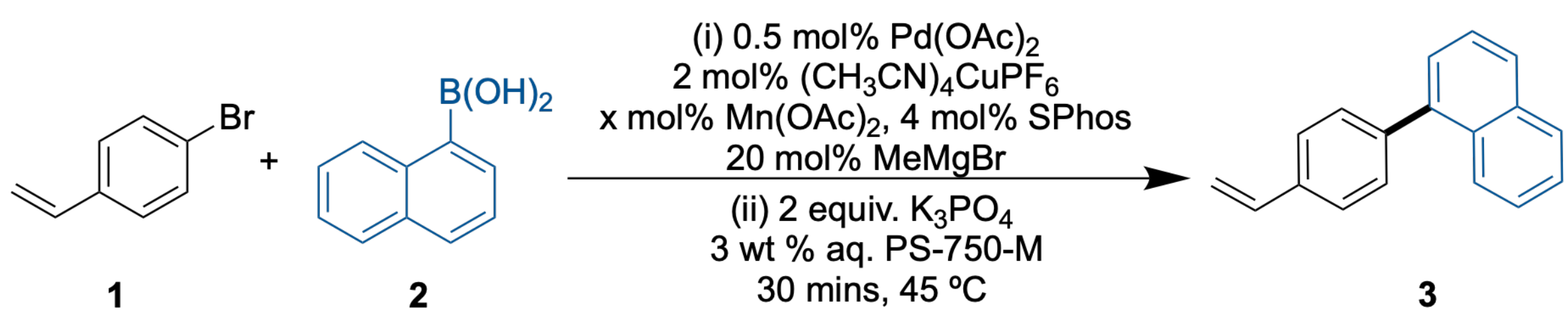

entry	$Mn(OAc)_2$ (mol%)	**3** (%)[a]
1	-	61
2	1	81
3	2	89
**4**	**3**	**97**
5	4	96
6	5	97

Conditions: 1 (0.25 mmol), **2** (0.3 mmol), SPhos (4 mol%), $Pd(OAc)_2$ (0.5 mol%), $Cu(CH_3CN)_4PF_6$ (2 mol%), $Mn(OAc)_2$ (x mol%), MeMgBr (20 mol%), K_3PO_4 (2.0 equiv.), 0.1 mL THF, 3 wt % PS-750-M in H_2O (1.0 mL), 45 °C, 30 minutes. [a]Yields based on GC-MS analysis.

Table S7. Optimization of $Cu(CH_3CN)_4PF_6$ loading

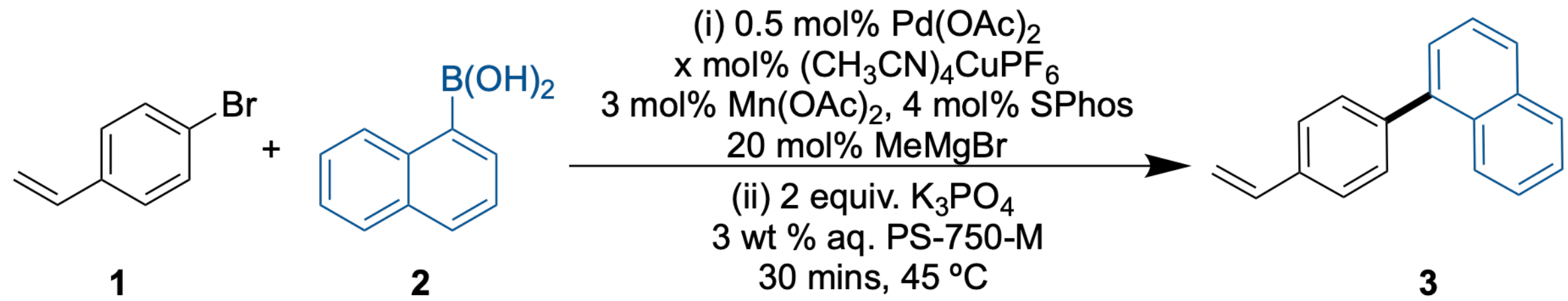

entry	$Cu(CH_3CN)_4PF_6$ (mol%)	**3** (%)[a]
1	-	8
2	0.5	74
**3**	**1**	**97**
4	2	97
5	3	97

Conditions: **1** (0.25 mmol), **2** (0.3 mmol), SPhos (4 mol%), $Pd(OAc)_2$ (0.5 mol%), $Cu(CH_3CN)_4PF_6$ (x mol %), $Mn(OAc)_2$ (3 mol%), MeMgBr (20 mol%), K_3PO_4 (2.0 equiv.), 0.1 mL THF, 3 wt % PS-750-M in H_2O (1.0 mL), 45 °C, 30 minutes. [a]Yields based on GC-MS analysis.

Table S8. Optimization of SPhos loading

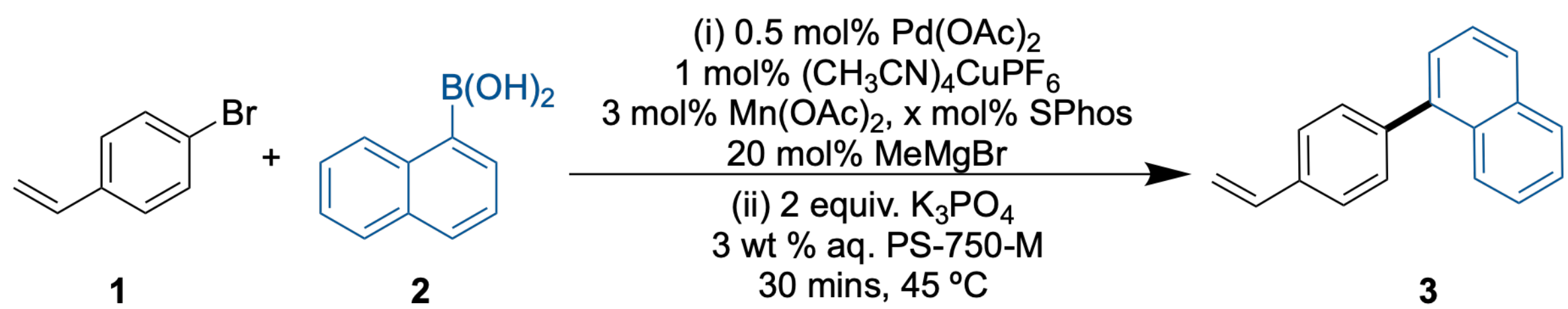

entry	SPhos (mol%)	**3** (%)[a]
1	-	-
2	1	77
**3**	**2**	**96**
4	3	96
5	4	96

Conditions: **1** (0.25 mmol), **2** (0.3 mmol), SPhos (x mol%), $Pd(OAc)_2$ (0.5 mol%), $Cu(CH_3CN)_4PF_6$ (1 mol%), $Mn(OAc)_2$ (3 mol%), MeMgBr (20 mol%), K_3PO_4 (2.0 equiv.), 0.1 mL THF, 3 wt % PS-750-M in H_2O (1.0 mL), 45 °C, 30 minutes. [a]Yields based on GC-MS.

Table S9. Optimization of temperature

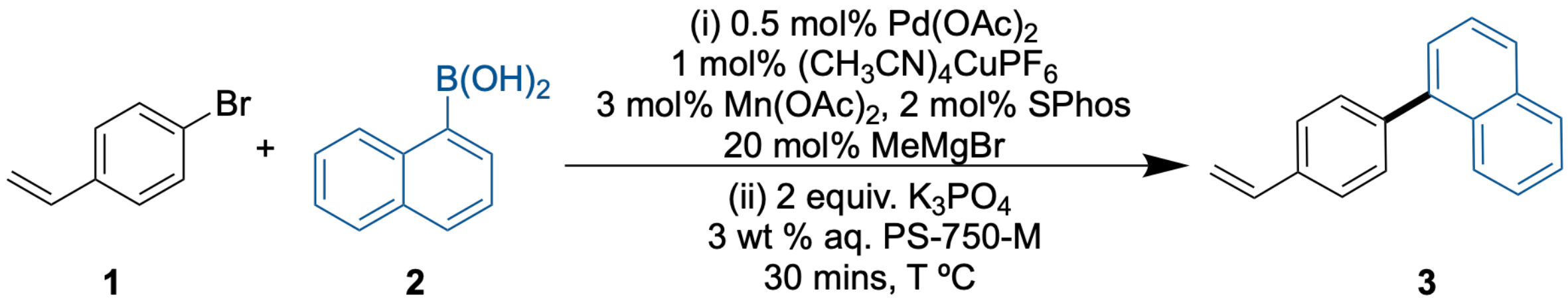

entry	temperature (°C)	**3** (%)[a]
1	rt	traces
2	35	77
**3**	**45**	**96**
4	60	96

Conditions: **1** (0.25 mmol), **2** (0.3 mmol), SPhos (2 mol%), $Pd(OAc)_2$ (0.5 mol%), $Cu(CH_3CN)_4PF_6$ (1 mol%), $Mn(OAc)_2$ (3 mol%), MeMgBr (20 mol%), K_3PO_4 (2.0 equiv.), 0.1 mL THF, 3 wt % PS-750-M in H_2O (1.0 mL), 30 minutes. [a]Yields based on GC-MS.

Table S10. Optimization of the base equivalent

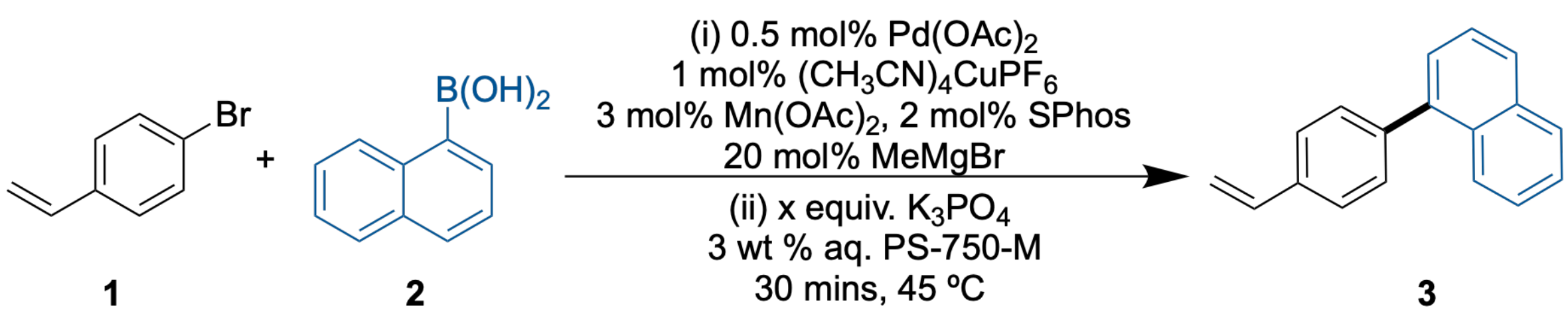

entry	base (equiv.)	**3** (%)[a]
1	1.0	51
2	1.5	79
**3**	**2.0**	**96**

Conditions: **1** (0.25 mmol), **2** (0.3 mmol), SPhos (2 mol%), $Pd(OAc)_2$ (0.5 mol%), $Cu(CH_3CN)_4PF_6$ (1 mol%), $Mn(OAc)_2$ (3 mol%), MeMgBr (20 mol%), K_3PO_4 (2.0 equiv.), 0.1 mL THF, 3 wt % PS-750-M in H_2O (1.0 ml), 45 °C, 30 minutes. [a]Yields based on GC-MS.

Table S11. Optimal reaction medium

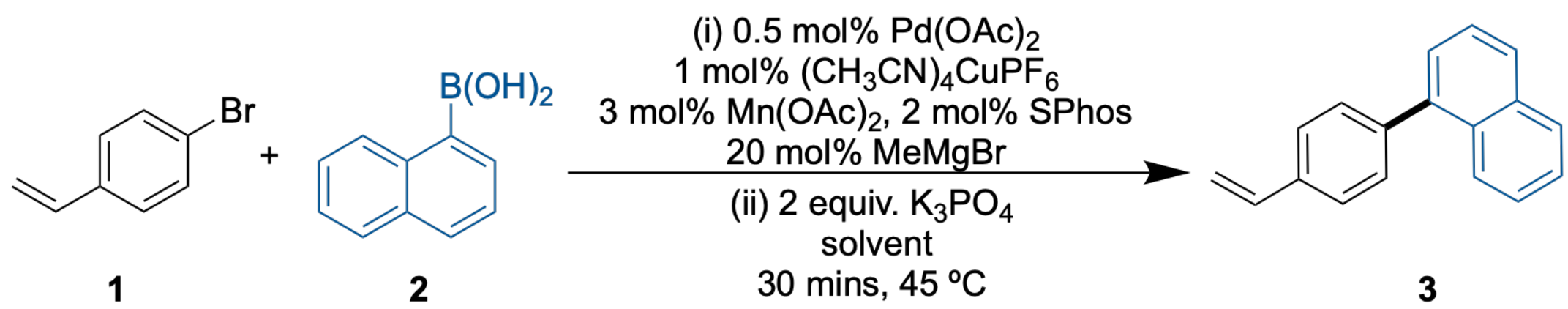

entry	solvent	**3** (%)[a]
1	THF	27
2	DMF	-
3	neat H_2O	42
**4**	**3 wt % aq. PS-750-M/HPMC (4:1)**	**98**[b]
5	3 wt % aq. TPGS-750-M/HPMC (4:1)	89[c]
6	3 wt % aq. Triton X-100/HPMC (4:1)	86[c]

Conditions: **1** (0.25 mmol), **2** (0.3 mmol), SPhos (2 mol%), $Pd(OAc)_2$ (0.5 mol%), $Cu(CH_3CN)_4PF_6$ (1 mol%), $Mn(OAc)_2$ (3 mol%), MeMgBr (20 mol%), K_3PO_4 (2.0 equiv.), 0.1 mL THF, solvent (1.0 mL), 45 °C, 30 minutes. [a]Yields based on GC-MS conversions. [b]reaction time 10 minutes. [c]Isolated yield.

Table S12. Optimization of HPMC/PS-750-M ratio

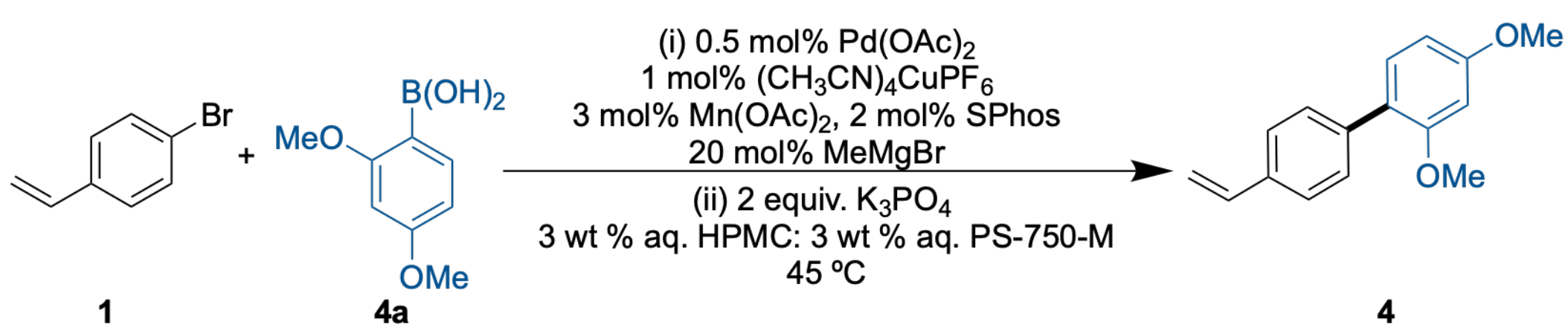

entry	3 wt % HPMC (mL)	3 wt % PS-750-M (mL)	**4** after 10 min (%)[a]	**4** after 30 min (%)[a]
1	0.9	0.1	79	82
2	0.8	0.2	84	90
3	0.7	0.3	87	94
4	0.6	0.4	95	100
**5**	**0.5**	**0.5**	**100**	**100**
6	0.4	0.6	94	100
7	0.3	0.7	90	97
**8**	**0.2**	**0.8**	**100**	**100**
9	0.1	0.9	96	100

Conditions: **1** (0.25 mmol), **4a** (0.3 mmol), SPhos (2 mol%), $Pd(OAc)_2$ (0.5 mol%), $Cu(CH_3CN)_4PF_6$ (1 mol%), $Mn(OAc)_2$ (3 mol%), MeMgBr (20 mol%), K_3PO_4 (2.0 equiv.), 0.1 mL THF, 3 wt % HPMC in H_2O (x mL) : 3 wt % PS-750-M in H_2O (x mL), 45 °C. [a]Yields based on GC-MS.

3. GENERAL PROCEDURE FOR TRIMETALLIC CATALYST SYNTHESIS

In an oven-dried 25 mL round-bottom reaction flask containing a PTFE-coated stir bar, SPhos (118.3 mg, 0.289 mmol, 4 equiv.), $Pd(OAc)_2$ (16.2 mg, 0.072 mmol, 1.0 equiv.), $Cu(CH_3CN)_4PF_6$ (53.7 mg, 0.144 mmol, 2 equiv.), $Mn(OAc)_2$ (75 mg, 0.432 mmol, 6 equiv.) were added. The reaction vial was closed with a rubber septum. The reaction vial was evacuated and then backfilled with argon three times. Then, 9 mL of dry THF was added to the reaction mixture, which was stirred for 30 min at rt. To the reaction mixture, 0.96 mL of a solution of MeMgBr (3M solution in THF) (20 equiv.) was added dropwise. During the addition of MeMgBr, the reaction mixture turned from yellow to brown. The reaction mixture was stirred for an additional 30 min at rt. 0.1 mL of 3 wt % PS-750-M/HPMC (4: 1) in H_2O was used to quench the excess of MeMgBr. Finally, volatiles were removed under reduced pressure, and the resulting solid material was dried under vacuum for an hour. The resulting brown solid (yield 600 mg) was used as such a catalyst for the selective couplings. The catalyst is stable under air and can be stored under ambient conditions for months.

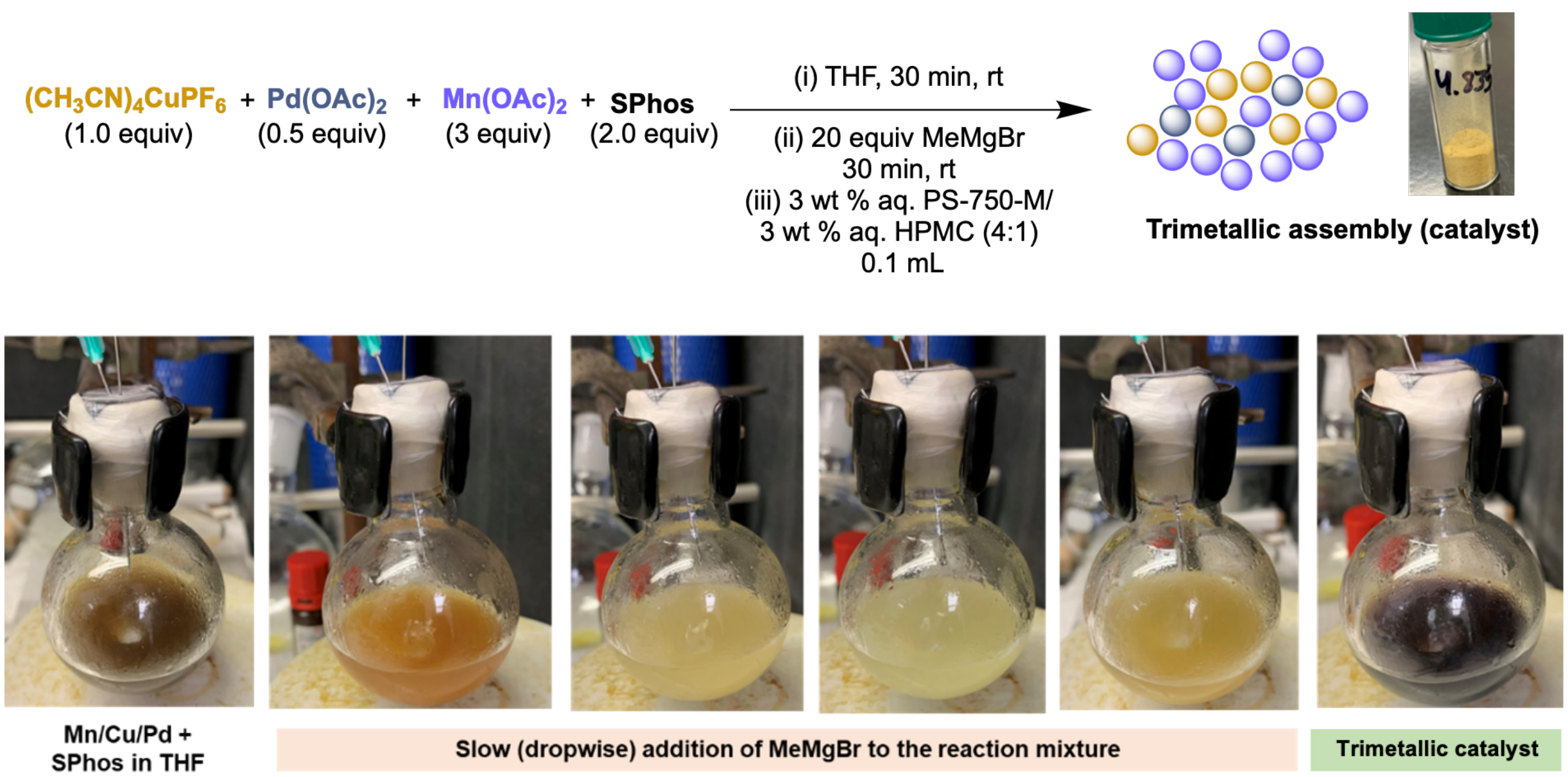

Figure S1. Reaction progress for the synthesis of trimetallic catalyst.

4. GENERAL PROCEDURE FOR COUPLING REACTIONS

4.1. Coupling reactions with *in-situ* generated trimetallic catalyst: SPhos (2.0 mg, 2 mol%), $Pd(OAc)_2$ (0.28 mg, 0.5 mol%) (stock solution in CH_2Cl_2), $Cu(CH_3CN)_4PF_6$ (0.8 mg, 1 mol%), $Mn(OAc)_2$ (1.2 mg, 3 mol%) were added into the 4 mL oven-dried round-bottomed flask containing PTFE-coated stirrer bar. The reaction vial was evacuated and backfilled with argon three times, then 0.1 mL of THF was added to the reaction mixture, which was stirred for 10 min at rt. After 10 min, 3.0 M MeMgBr solution in THF (0.017 mL, 20 mol%) was added to the reaction mixture. Upon the addition of the Grignard reagent, the mixture turned dark brown. The reaction mixture was stirred for 10 minutes at rt. 0.8 mL 3.0 wt % PS-750-M in H_2O and 0.2 mL 3.0 wt % HPMC in H_2O were added to the reaction mixture, which was stirred for 10 minutes at rt. (Hetero)aryl bromide (0.25 mmol), arylboronic acid (0.375 mmol), and K_3PO_4 (106 mg, 0.5 mmol) were added to the reaction mixture under an argon atmosphere. The reaction vial was evacuated and then backfilled with argon. The reaction mixture was stirred at 45 °C until the starting materials were completely consumed. After complete consumption of starting materials as monitored by TLC or GC-MS, the reaction mixture was allowed to cool to rt. 1 mL of EtOAc was added to the reaction mixture, and the mixture was stirred for 1 min. The organic layer was withdrawn using a syringe needle. This procedure was repeated twice (2 x 1 mL EtOAc). The combined organic layer was dried over anhydrous sodium sulfate. Finally, volatiles were removed under reduced pressure to obtain crude product, which was further purified by flash chromatography over silica gel using EtOAc/hexanes as eluent.

4.2. Coupling reactions with the isolated trimetallic catalyst: The trimetallic catalyst was prepared according to the general procedure provided above (section 3, page 10). (Hetero)aryl bromide (0.25 mmol), arylboronic acid (0.375 mmol), isolated catalyst (17.4 mg), and K_3PO_4 (106 mg, 0.5 mmol) were added to the 4 mL oven-dried round-bottomed flask containing PTFE-coated stirrer bar under argon atmosphere, then the septum was closed. 0.8 mL 3.0 wt % PS-750-M in H_2O and 0.2 mL 3.0 wt % HPMC in H_2O were added to the reaction mixture, followed by 0.1 mL THF as co-solvent. The reaction mixture was stirred at 45 °C until the starting materials were completely consumed. After complete consumption of starting materials as monitored by TLC or GC-MS, the reaction mixture was allowed to cool to rt. 1 mL of EtOAc was added to the reaction mixture, and the mixture was stirred for 1 minute. The organic layer was withdrawn using a syringe

needle. This procedure was repeated twice (2 x 1 mL EtOAc). The combined organic layer was dried over anhydrous sodium sulfate. Finally, volatiles were removed under reduced pressure to obtain a semi-pure product, which was further purified by flash chromatography on silica gel using EtOAc/hexanes as eluent.

5. HRTEM AND XPS ANALYSIS

5.1a. HRTEM analysis of trimetallic catalyst prepared in aq. 3 wt% PS-750-M/HPMC mixed media

Sample preparation: The trimetallic catalyst was prepared using the general procedure described in section 3, page S10. Once prepared, the catalyst was suspended in a 3 wt % aq. PS-750-M/ HPMC mixed media was subjected to HRTEM analysis.

HRTEM and EDS analysis: High-resolution transmission electron microscopy (HRTEM) and scanning transmission electron microscopy-based high-angle annular dark-field imaging (STEM-HAADF) were used to evaluate the morphology and size distribution of the trimetallic catalyst assembly. In addition, energy-dispersive X-ray spectroscopy (EDX) was used to analyze the sample's composition. HRTEM, STEM-HAADF, and EDX analysis were conducted using a 200 kV field-emission gun FEI Tecnai F20 microscope, and specimens were prepared by drop-casting catalyst dispersions onto commercial copper grid-supported amorphous holey carbon films. HRTEM and STEM-HAADF analysis showed the formation of a small nanoparticle assemblies like catalyst. EDS mapping of the sample confirmed the presence of carbon, oxygen, and copper. The sample was also analyzed by EDX to determine its chemical composition.

5.1b. HRTEM analysis of trimetallic catalyst prepared in only aq. 3 wt% PS-750-M

Sample preparation: The trimetallic catalyst was prepared with the general procedure described in section 3, page S10, but only PS-750-M was used in the quenching process. Once prepared, the catalyst was suspended in 3 wt % aq. PS-750-M solution and was subjected to HRTEM, STEM-HAADF, and EDX analysis.

5.1c. HRTEM analysis of trimetallic catalyst prepared in only aq. 3 wt% HPMC

Sample Preparation: The trimetallic catalyst was prepared with the general procedure described in section 3, page S10, but only aq. HPMC was used in the quenching process. Once prepared, the catalyst was suspended in a 3 wt % aq. HPMC solution was subjected to HRTEM, STEM-HAADF, and EDX analysis.

5.2 XPS analysis of trimetallic catalyst. The trimetallic catalyst was prepared with the general procedure described in section 3, page S10. Once prepared, the catalyst was suspended in a 3 wt % aq. PS-750-M/ HPMC mixed media. The sample was dried and analyzed by XPS.

6. XAS ANALYSIS OF Pd/Cu/Mn TRIMETALLIC CATALYST

A) Mn K edge

Table S13. Best fitting parameters for the Mn K edge EXAFS spectra. The amplitude reduction factor was fitted from the Mn reference foil as 0.88, the E_0 was refined as a global parameter for the sample and was found as -8.2 eV.

Scattering path	N	R(Å)	σ^2(Å^2)
Mn-O	1.8 ± 0.6	1.93 ± 0.03	0.007 ± 0.003
Mn-O	0.9 ± 0.3	2.18 ± 0.07	0.007 ± 0.003
Mn-P	0.5 ± 0.3	2.37 ± 0.04	0.003 ± 0.003
Mn-Mn	2.6 ± 0.7	3.21 ± 0.03	0.013 ± 0.006

B) Cu K edge

Table S14. Best fitting parameters for the Cu K edge EXAFS spectra. The amplitude reduction factor was fitted from the Cu reference foil as 0.86, the E_0 was refined as a global parameter for the sample and was found as 0.7 eV.

Scattering path	N	R(Å)	σ^2(Å^2)
Cu-O/N	1.2 ± 0.6	1.95 ± 0.06	0.008 ± 0.004
Cu-P	1.2 ± 0.6	2.22 ± 0.03	0.008 ± 0.005
Cu-Cu	1.0 ± 0.3	2.49 ± 0.02	0.008 ± 0.002

C) Pd K edge

Table S15. Best fitting parameters for the Pd K edge EXAFS spectra The amplitude reduction factor was fitted from the Pd reference foil as 0.95, the E_0 was refined as a global parameter for the sample and was found as 3.9 eV.

Scattering path	N	R(Å)	σ^2(Å^2)
Pd-P	1.8 ± 0.3	2.32 ± 0.02	0.003 ± 0.001
Pd-Pd	1.6 ± 0.5	2.96 ± 0.03	0.005 ± 0.002

7. Control ^{31}P NMR EXPERIMENTS

All experiments were carried out in Varian Innova 400 MHz NMR at 25 °C using $CDCl_3$ as NMR solvent. A sealed capillary containing PPh_3 dissolved in $CDCl_3$ was used as an internal standard.

7.1 Procedure for ligation of SPhos with metal precursors

In a 4 mL reaction vial containing a PTFE-coated magnetic stir bar, 0.005 mmol SPhos and 0.005 mmol of metal salt were dissolved in 0.5 mL of THF under an argon atmosphere. The mixture was stirred for 10 minutes at 45 °C. The solution was transferred to an NMR tube for ^{31}P NMR analysis.

7.2 Procedure for the synthesis of a trimetallic catalyst (before addition of MeMgBr)

Three sets of reactions were set up. In 4 mL reaction vials containing a PTFE-coated magnetic stir bar, 0.005 mmol of SPhos was mixed with 0.005 mmol of $Pd(OAc)_2$ in 0.5 mL of THF under an argon atmosphere. To a separate reaction vial, 0.005 mmol of SPhos was mixed with 0.005 mmol of $Cu(CH_3CN)_4PF_6$ in 0.5 mL of THF under an argon atmosphere. In the third reaction vial, 0.005 mmol SPhos was mixed with 0.005 mmol of $Mn(OAc)_2$ in 0.5 mL of THF. All three reaction vials were stirred for 10 minutes at 45 °C. After 10 minutes, the three reaction mixtures were mixed together and stirred again for another 10 minutes at 45 °C. The resulting Pd/Cu/Mn-SPhos complex was transferred to an NMR tube for ^{31}P NMR analysis.

7.3 Procedure for the synthesis of a trimetallic catalyst (after addition of MeMgBr)

Three sets of reactions were set up. In 4 mL reaction vials containing a PTFE-coated magnetic stir bar, 0.005 mmol of SPhos was mixed with 0.005 mmol of $Pd(OAc)_2$ in 0.5 mL of THF under an argon atmosphere. To a separate reaction vial, 0.005 mmol of SPhos was mixed with 0.005 mmol of $Cu(CH_3CN)_4PF_6$ in 0.5 mL of THF under an argon atmosphere. In the third reaction vial, 0.005 mmol SPhos was mixed with 0.005 mmol of $Mn(OAc)_2$ in 0.5 mL of THF. All three reaction vials were stirred for 10 minutes at 45 °C. After 10 minutes, the three reaction mixtures were mixed and stirred again for another 10 minutes at 45 °C. After 10 minutes, the mixture was cooled to rt followed by the addition of 20 mol % 3 M MeMgBr in THF. The reaction mixture was stirred for another 10 minutes. To the reaction mixture, 0.1 mL of a mixture of 3 wt % PS-750-M/HPMC (4:1) in D_2O was added. The resulting Pd/Cu/Mn-SPhos catalyst was transferred to an NMR tube for ^{31}P NMR analysis.

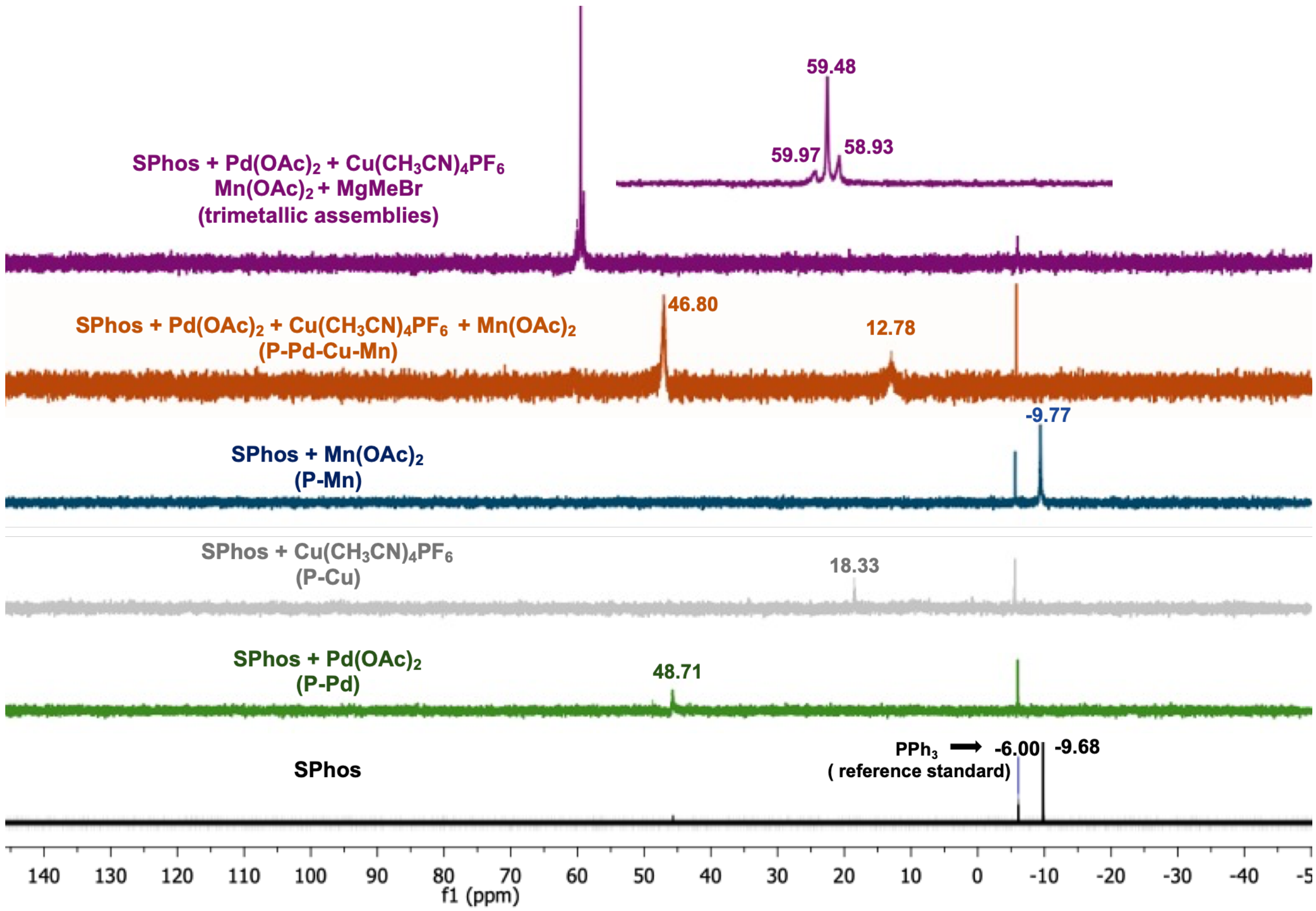

Figure S2. ^{31}**P NMR experiments**

8. CATALYTIC EFFICIENCY AND LONGEVITY STUDY

Procedure. SPhos (4.1 mg, 4 mol%), $Pd(OAc)_2$ (0.28 mg, 0.5 mol%) (from stock solution in CH_2Cl_2), $Cu(CH_3CN)_4PF_6$ (1.86 mg, 2 mol%), $Mn(OAc)_2$ (2.16 mg, 5 mol%) were added into the 4 mL oven-dried round-bottomed flask containing PTFE-coated stirrer bar. The reaction vial was evacuated and backfilled with argon three times, then 0.1 mL of THF was added to the reaction mixture, which was stirred for 10 min at rt. After 10 min, a 3.0 M MeMgBr solution in Et_2O (0.017 mL, 20 mol%) was added to the reaction mixture using a microliter syringe. Upon the addition of the Grignard reagent, the mixture turned dark brown. The reaction mixture was stirred for 10 minutes at rt. Then, 1 mL of 3 wt % PS-750-M and HPMC (4:1) solution in H_2O was added to the reaction mixture, and the mixture was stirred for 10 minutes. Then, **1** (0.25 mmol), **2** (0.375 mmol), and K_3PO_4 (80 mg, 0.375 mmol) were added to the reaction mixture under an argon atmosphere. The reaction vial was evacuated and then backfilled with argon. The reaction mixture was then stirred at 45 °C for 10 mins. After complete consumption of starting materials as monitored by TLC and GC-MS, the reaction mixture was allowed to cool to rt and left as is for the next 30 mins. Then, the vial was opened, and the second portion of **1** (0.25 mmol), **2** (0.375 mmol), and K_3PO_4 (80 mg, 0.375 mmol) were quickly added, and the reaction was again stirred at 45 °C for 10 mins. The completion of the reaction was monitored by TLC and GC-MS (by the removal of 0.1 mL of the analyte). Then, the mixture was again kept at rt as is for 12 h.

Then, the third portion of **1** (0.25 mmol), **2** (0.375 mmol), and K_3PO_4 (80 mg, 0.375 mmol) were quickly added, and the mixture was diluted with an additional 1 mL of 3 wt % aq. PS-750-M/HPMC solution and mixture were stirred at 45 °C till complete consumption of the starting material (i.e., 45 minutes). The mixture was diluted with 1 mL of distilled H_2O, and 2 mL of EtOAc was added, and the mixture was stirred for 1 minute. The organic layer was withdrawn using a syringe needle. This procedure was repeated twice (2 x 2 mL EtOAc). The combined organic layer was dried over anhydrous sodium sulfate. Finally, volatiles were removed under reduced pressure to obtain crude product, which was further purified by flash chromatography over silica gel using (1: 20) EtOAc/hexanes as eluent, affording **3** in 90% (155 mg) isolated yield.

9. SUBSTRATE SCOPE

Table S16. Substrate scope

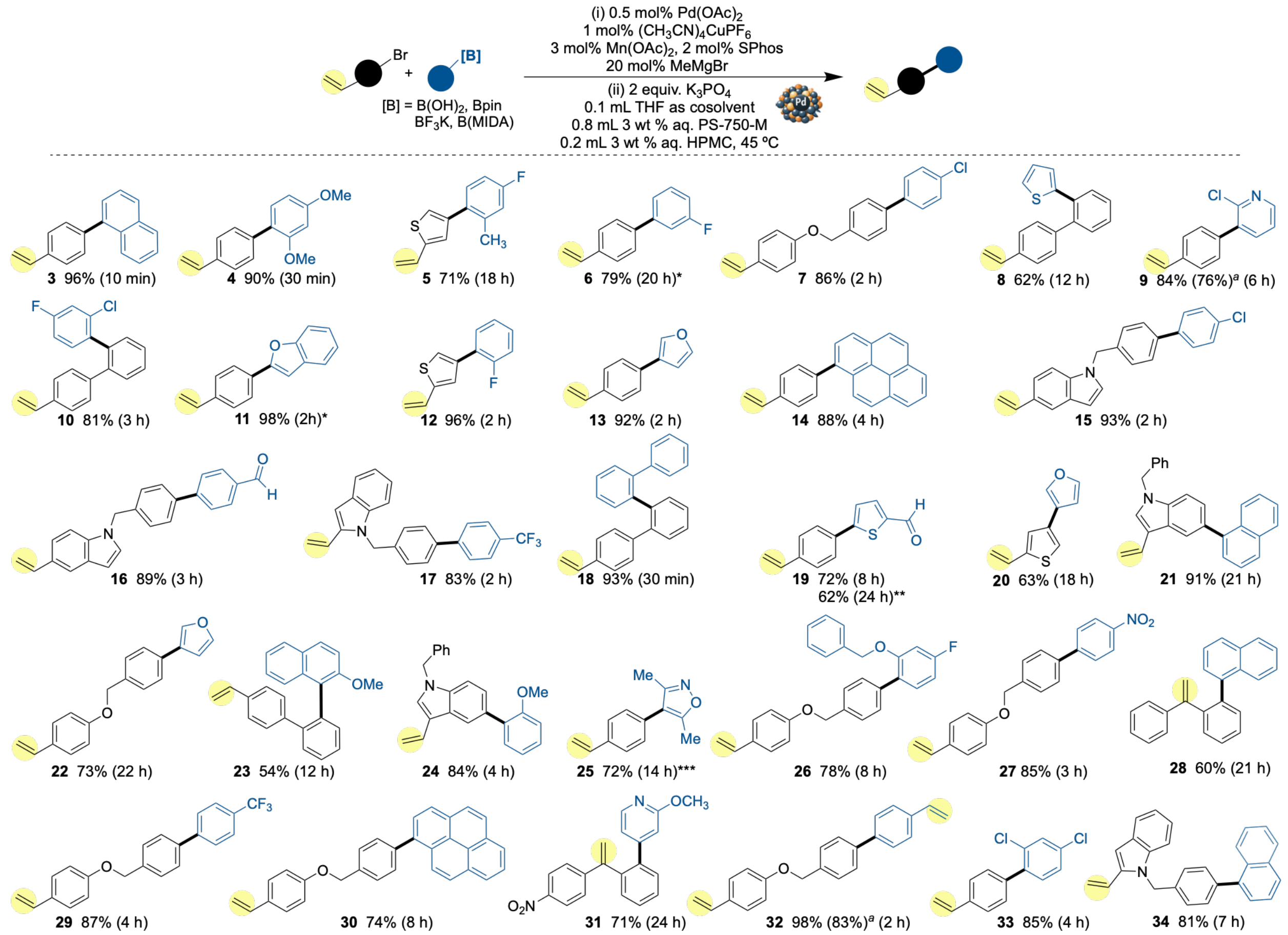

Conditions. Aryl bromide (0.25 mmol), boronic acid (0.375 mmol), SPhos (2 mol%), $Pd(OAc)_2$ (0.5 mol%), $Cu(CH_3CN)_4PF_6$ (1 mol%), $Mn(OAc)_2$ (3 mol%), MeMgBr (20 mol%), K_3PO_4 (2.0 equiv), 3 wt % PS-750-M/3wt % HPMC (4:1) in H_2O (1 mL), 45 °C. * BMIDA salt was used instead of boronic acid. **BF_3K salt was used instead of boronic acid. ***Bpin ester was used instead of boronic acid. All yields are isolated.

10. GRAM SCALE REACTIONS

10.1. Gram scale reaction with isolated catalyst

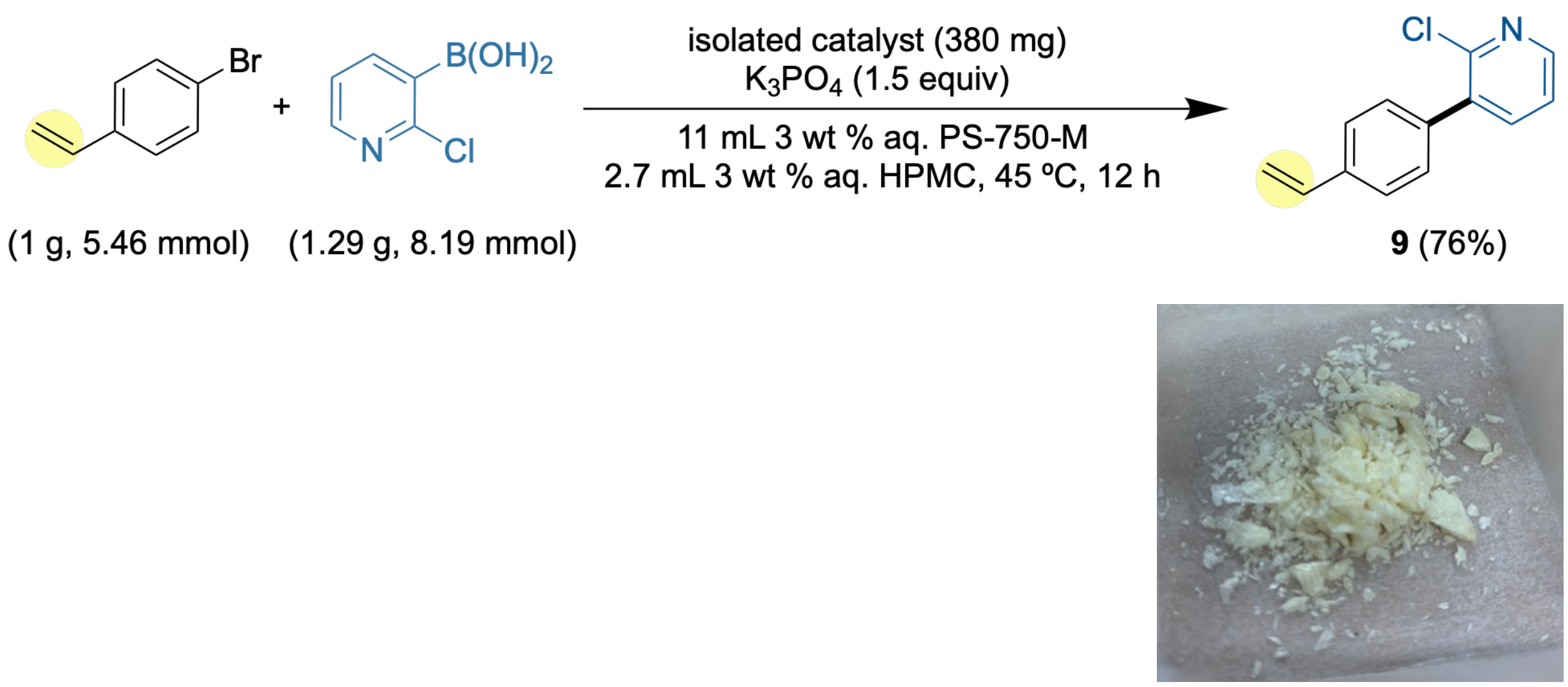

Scheme S1. Synthesis of **9** at a gram scale.

Procedure. 1-Bromo-4-vinylbenzene **1** (1 g, 5.46 mmol), (2-chloropyridin-4-yl)boronic acid (1.29 g, 8.19 mmol), isolated catalyst (380 mg, 5000 ppm Pd), and K_3PO_4 (1.1 g, 5.19 mmol) were added to a 25 mL oven-dried round-bottom flask containing a PTFE-coated stir bar under argon atmosphere, then the flask was closed with a rubber septum. 11 mL of freshly degassed 3 wt% PS-750-M in H_2O and 2.7 mL of 3 wt% HPMC in H_2O were added to the reaction mixture. The reaction mixture was stirred at 45 ºC until complete consumption of starting materials, as monitored by TLC or GC-MS. At this point, the flask was allowed to cool to rt, 5 mL EtOAc was added to the reaction mixture, and the mixture was stirred at rt for a minute. The organic layer was withdrawn with the aid of a syringe. The same protocol was repeated twice (2 mL of EtOAc/time). The combined organic layer was dried over anhydrous sodium sulfate. Volatiles were removed under reduced vacuum using a rotary evaporator to obtain the desired product as a light-yellow solid **9** (yield 0.89 g, 76%, Scheme S1).

10.2. Gram scale reaction with in-situ formed catalyst

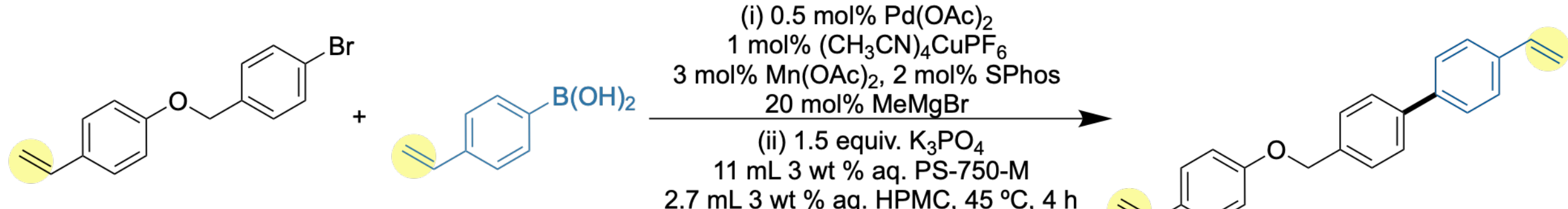

(1 g, 3.46 mmol) (0.768 g, 5.19 mmol) **32** (83%)

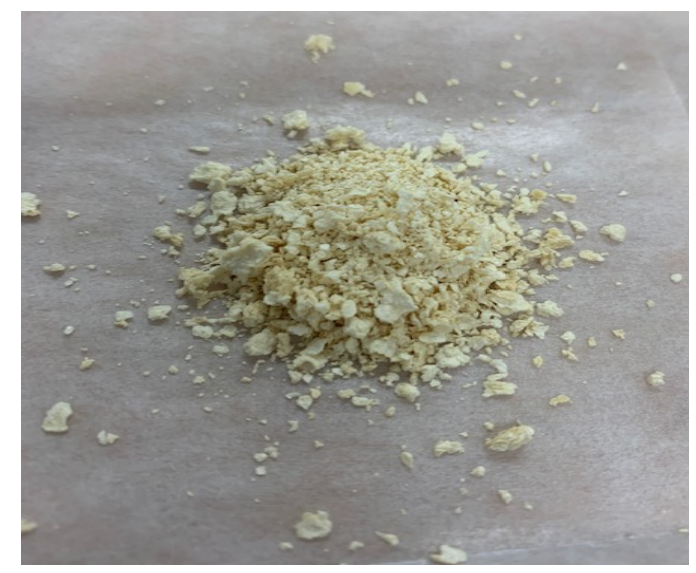

Scheme S2. Synthesis of **32** at a gram scale.

Procedure. SPhos (27 mg, 2 mol%), $Pd(OAc)_2$ (3.88 mg, 0.5 mol%), $Cu(CH_3CN)_4PF_6$ (13 mg, 1 mol%), $Mn(OAc)_2$ (18 mg, 3 mol%) were added to a 25 mL oven-dried round-bottom flask containing a PTFE-coated stir bar. The reaction vial was evacuated and backfilled with argon three times, then 4 mL of dry THF was added to the reaction mixture and stirred for 10 min at rt. After 10 min, 3M MeMgBr solution in Et_2O (0.23 mL, 20 mol%) was added to the reaction mixture. Upon the addition of the Grignard reagent, the reaction mixture turned dark brown. Reaction mixture was additionally stirred for 10 mins at rt, then 11 mL 3 wt% PS-750-M in H_2O and 2.7 mL 3 wt% HPMC in H_2O were introduced to the reaction mixture, and the reaction mixture was stirred for 10 mins at rt. 1-bromo-4-((4-vinylphenoxy)methyl)benzene (1 g, 3.46 mmol), (4-vinylphenyl)boronic acid (0.768 g, 5.19 mmol), and K_3PO_4 (1.1 g, 5.19 mmol) were added to the reaction mixture under an argon atmosphere, the flask was closed with a rubber septum, followed by three cycles of evacuation and backfill with argon. The reaction mixture was stirred at 45 ºC until complete consumption of starting materials, as monitored by TLC or GC-MS. At this point, the flask was allowed to cool to rt, 5 mL EtOAc was added to the reaction mixture, and the mixture was stirred at rt for a minute. The organic layer was withdrawn with the aid of a syringe. The same protocol was repeated twice (2 mL of EtOAc/time). The combined organic layer was dried over anhydrous sodium sulfate. Volatiles were removed under reduced vacuum using a rotary evaporator to obtain the desired product as a light-yellow solid **32** (yield 0.9 g, 83%, Scheme S2).

11. KINETIC STUDY

11.1. Role of PS-750-M and HPMC on the reaction outcomes

Note: Slow-reacting substrates were chosen for this study to avoid any errors.

A) Reaction under standard conditions

A batch of six reactions was set up according to the general procedure provided in section 4.1 (using an in-situ synthesized trimetallic catalyst). Every 15 minutes, one reaction vial was allowed to cool to room temperature. 1 mL H_2O was added to the reaction, followed by 1 mL EtOAc and 0.25 mmol mesitylene as an internal standard. The reaction mixture was stirred for 2 minutes, and the reaction progress and formation of **13** were analyzed using GC-MS. The same analysis procedure was repeated for the remaining five reactions at 30, 45, 60, 75, and 90 minutes, respectively.

B). Reaction progress using aq. 3 wt % HPMC as reaction medium

A batch of six reactions was set up according to the general procedure provided in section 4 (using in-situ synthesized trimetallic catalyst). Every 15 minutes, one reaction vial was allowed to cool to room temperature. 1 mL H_2O was added to the reaction, followed by 1 mL EtOAc and 0.25 mmol mesitylene as an internal standard. The reaction mixture was stirred for 2 minutes, and the reaction progress and formation of **13** were analyzed using GC-MS. The same analysis procedure was repeated for the remaining five reactions at 30, 45, 60, 75, and 90 minutes, respectively.

C). Reaction progress using aq. 3 wt % PS-750-M as reaction medium.

A batch of six reactions was set up according to the general procedure provided in section 4 (using in-situ synthesized trimetallic catalyst). Every 15 minutes, one reaction vial was allowed to cool to room temperature. 1 mL H_2O was added to the reaction, followed by 1 mL EtOAc and 0.25 mmol mesitylene as an internal standard. The reaction mixture was stirred for 2 minutes, and the reaction progress and the formation of **13** were analyzed by GC-MS. The same analysis procedure was repeated for the remaining five reactions at 30, 45, 60, 75, and 90 minutes, respectively.

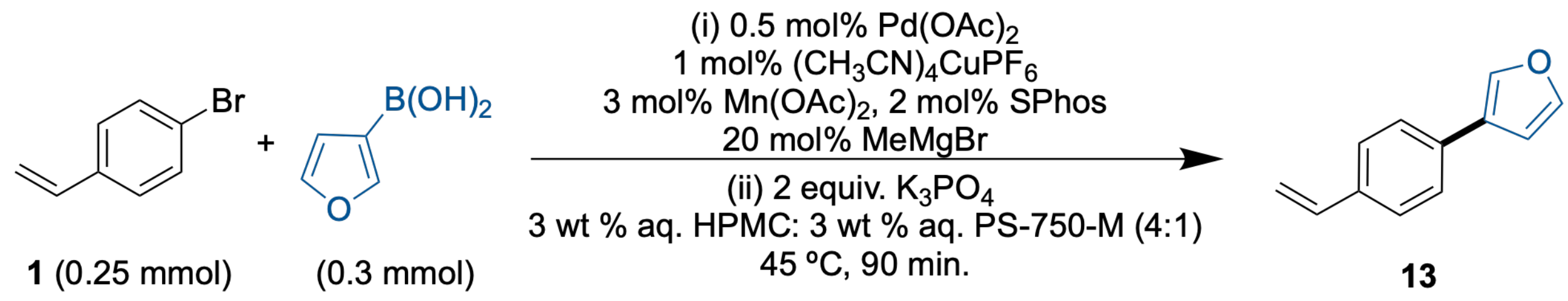

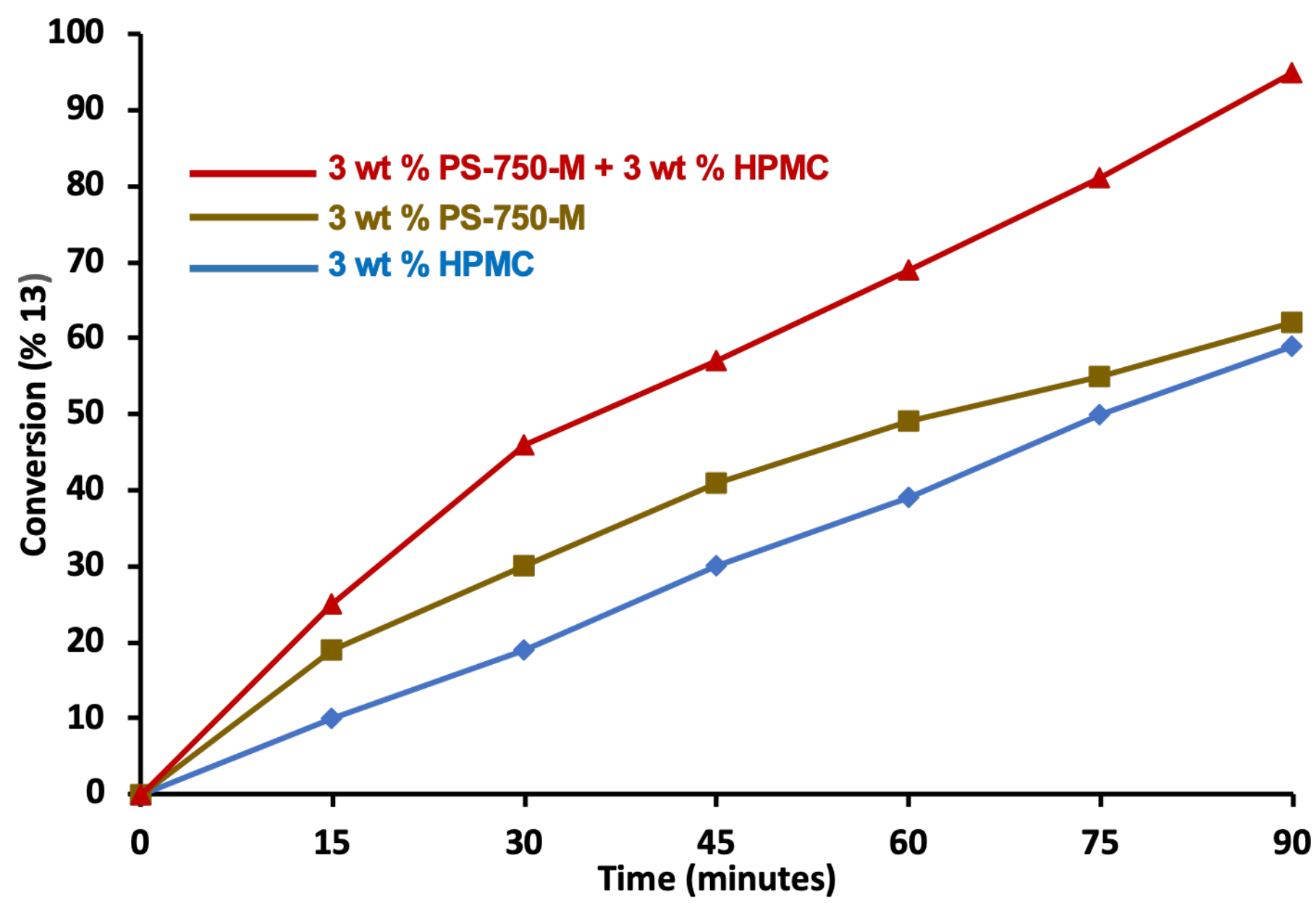

Figure S3. Role of PS-750-M and HPMC in the reaction.

11.2. Role of Pd/Cu/Mn in trimetallic catalyst

A) Reaction progress in the absence of [Cu] (Pd/Mn as a catalyst)

A batch of six reactions was set up according to the general procedure provided in section 4 (using in-situ-synthesized catalyst), but no $Cu(CH_3CN)_4PF_6$ was added during catalyst preparation. Every 15 minutes, one reaction vial was allowed to cool to room temperature. 1 mL H_2O was added to the reaction, followed by 1 mL EtOAc and 0.25 mmol mesitylene as an internal standard. The reaction mixture was stirred for 2 minutes, and the reaction progress and formation of **13** were analyzed using GC-MS. The same analysis procedure was repeated for the remaining five reactions at 30, 45, 60, 75, and 90 minutes, respectively.

B) Reaction progress in the absence of [Mn] – (Pd/Cu as catalyst)

A batch of six reactions was set up according to the general procedure provided in section 4 (using in-situ synthesized catalyst), but no $Mn(OAc)_2$ was added while preparing the catalyst. Every 15

minutes, one reaction vial was allowed to cool to room temperature. 1 mL H_2O was added to the reaction, followed by 1 mL EtOAc and 0.25 mmol mesitylene as an internal standard. The reaction mixture was stirred for 2 minutes, and the reaction progress and formation of **13** were analyzed using GC-MS. The same analysis procedure was repeated for the remaining five reactions at 30, 45, 60, 75, and 90 minutes, respectively.

C) Reaction progress in the absence of [Pd] – (Cu/Mn as catalyst)

A batch of six reactions was set up according to the general procedure provided in section 4 (using in-situ-synthesized catalyst), but no $Pd(OAc)_2$ was added during catalyst preparation. Every 15 minutes, one reaction vial was allowed to cool to rt, and 1 mL EtOAc was added, followed by the addition of 0.25 mmol of mesitylene as an internal standard. The reaction mixture was stirred for 2 minutes, and the reaction progress and formation of **13** were analyzed using GC-MS. The same analysis procedure was repeated for the remaining five reactions at 30, 45, 60, 75, and 90 minutes, respectively.

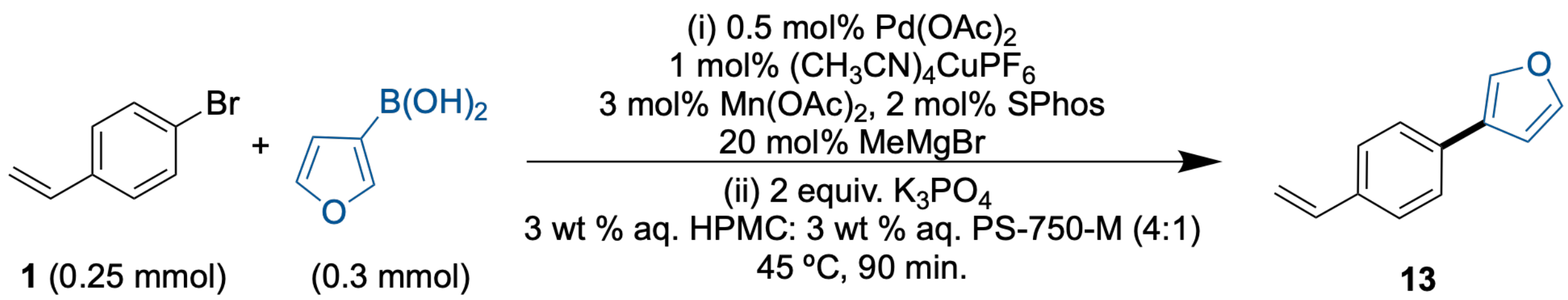

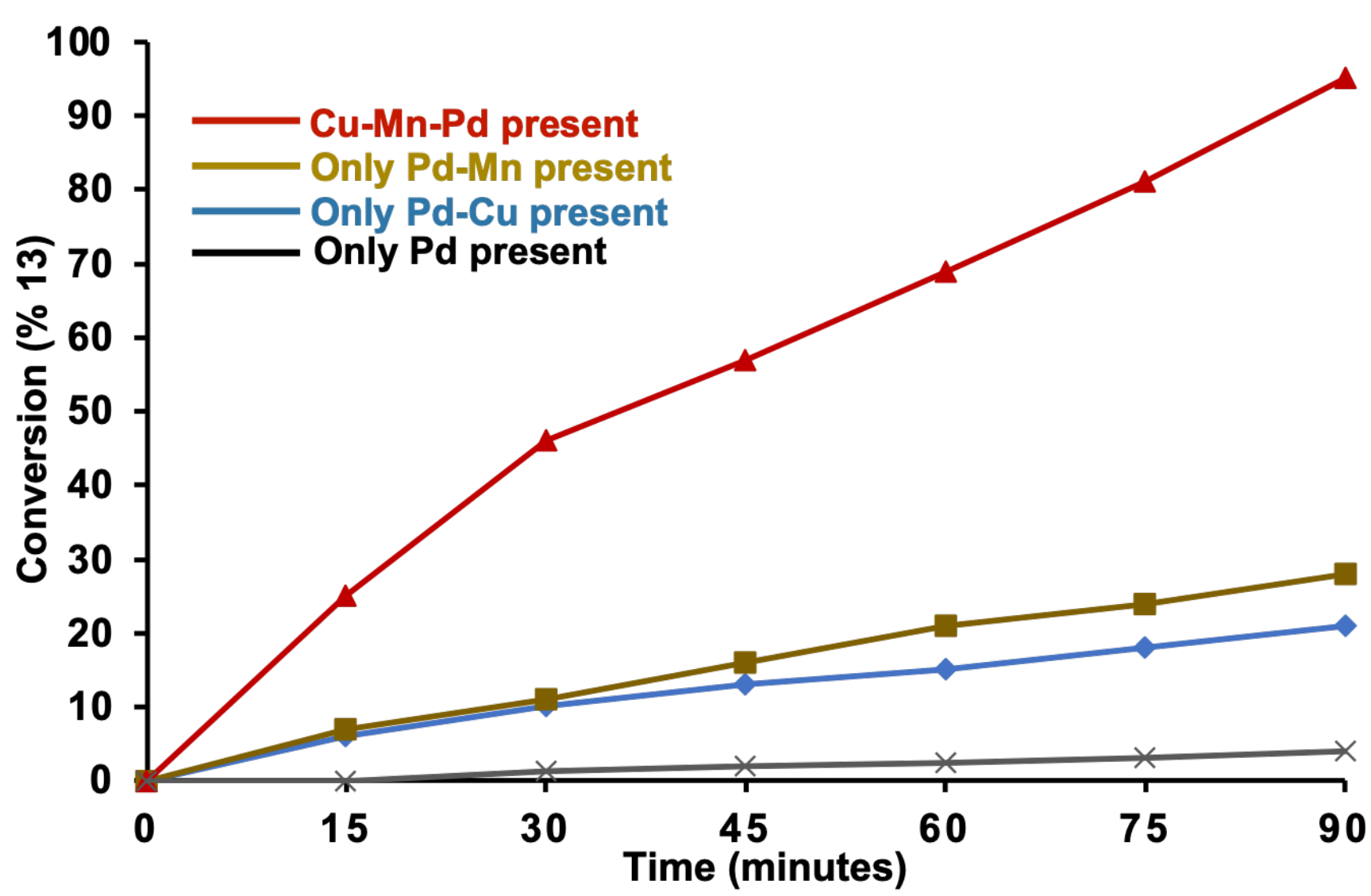

Figure S4. Reaction kinetics – role of each metal in the reaction.

11.3. Stability test of Isolated catalyst

A) Reaction kinetics using freshly prepared catalyst

The trimetallic catalyst was freshly prepared according to the general procedure provided in section 4. A batch of six reactions was set up according to the general procedure provided in section 8.3.4B. Every 15 minutes, one reaction vial was allowed to cool to rt, and 1 mL EtOAc was added, followed by the addition of 0.25 mmol of mesitylene as an internal standard. The reaction mixture was stirred for 2 minutes, and the reaction progress and the formation of **13** were analyzed by GC-MS. The same analysis procedure was repeated for the remaining five reactions at 30, 45, 60, 75, and 90 minutes, respectively.

B) Reaction progress using 6-month-old and 18-month-old catalyst

The trimetallic catalyst was prepared according to the general procedure provided in Section 4. Two separate batches of trimetallic catalyst were kept under ambient conditions for 6 months and for 18 months (under an argon atmosphere).

A set of six reactions was set up according to the general procedure provided in section 4 using a 6-month-old trimetallic catalyst. Another set of six reactions was set up using an 18-month-old catalyst. After every 15 minutes, one reaction vial from each set was allowed to cool to rt and 1 mL H_2O was added. 1 mL EtOAc was added to it followed by the addition of 0.25 mmol of mesitylene as internal standard. The reaction mixtures were stirred for 2 minutes, and the reaction progress and formation of **13** were analyzed using GC-MS. The same analysis procedure was repeated for the remaining five reactions in each set at 30, 45, 60, 75, and 90-minute time intervals.

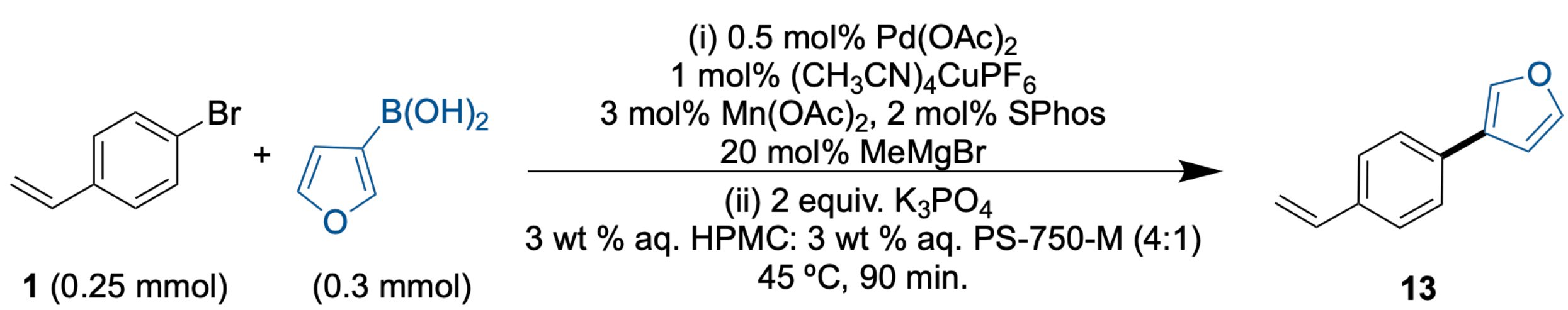

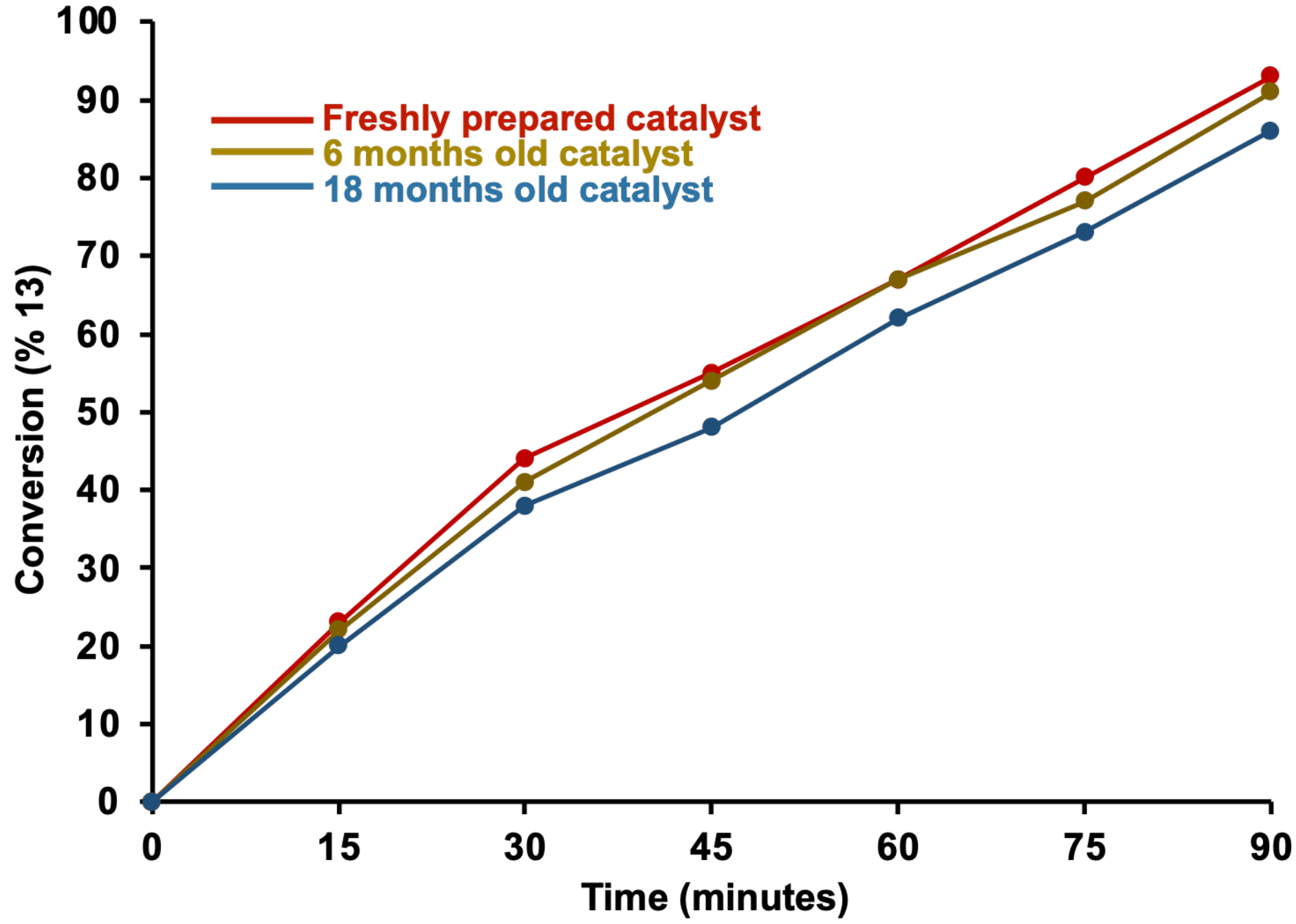

Figure S5. Reaction kinetics –stability of isolated trimetallic catalyst.

11.4. Reaction kinetics – in situ formed versus isolated catalyst

A) Reaction progress using an in-situ formed catalyst

A set of six reactions was set up according to the general procedure, section 4 (using in-situ-synthesized catalyst). Every 15 minutes, one reaction vial was allowed to cool to rt, and 1 mL EtOAc was added, followed by the addition of 0.25 mmol of mesitylene as an internal standard. The reaction mixture was stirred for 2 minutes, and the reaction progress and formation of **13** were analyzed using GC-MS. The same analysis procedure was repeated for the remaining five reactions at 30, 45, 60, 75, and 90 minutes, respectively.

B) Reaction progress using isolated catalyst

A set of six reactions was set up according to the general procedure section 4 (using isolated catalyst). Every 15 minutes, one reaction vial was allowed to cool to rt, and 1 mL EtOAc was added, followed by the addition of 0.25 mmol of mesitylene as an internal standard. The reaction mixture was stirred for 2 minutes, and the reaction progress and formation of **13** were analyzed

using GC-MS. The same analysis procedure was repeated for the remaining five reactions at 30, 45, 60, 75, and 90 minutes, respectively.

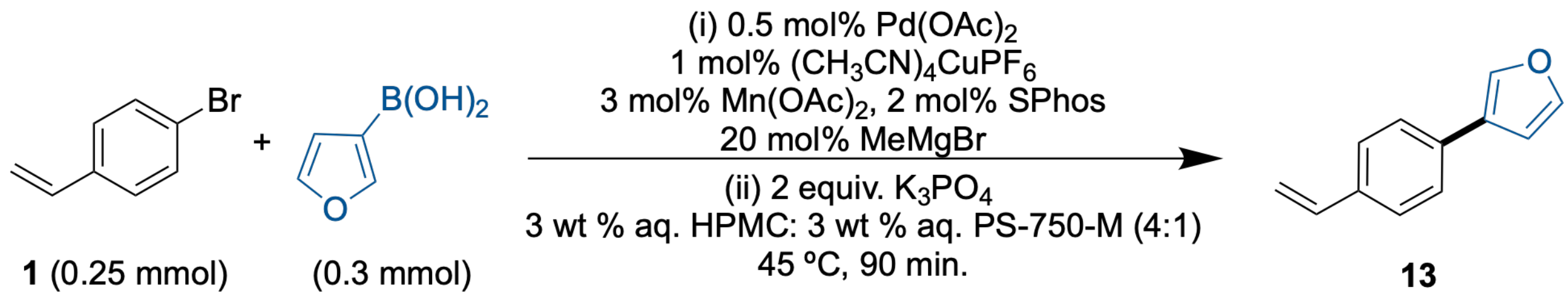

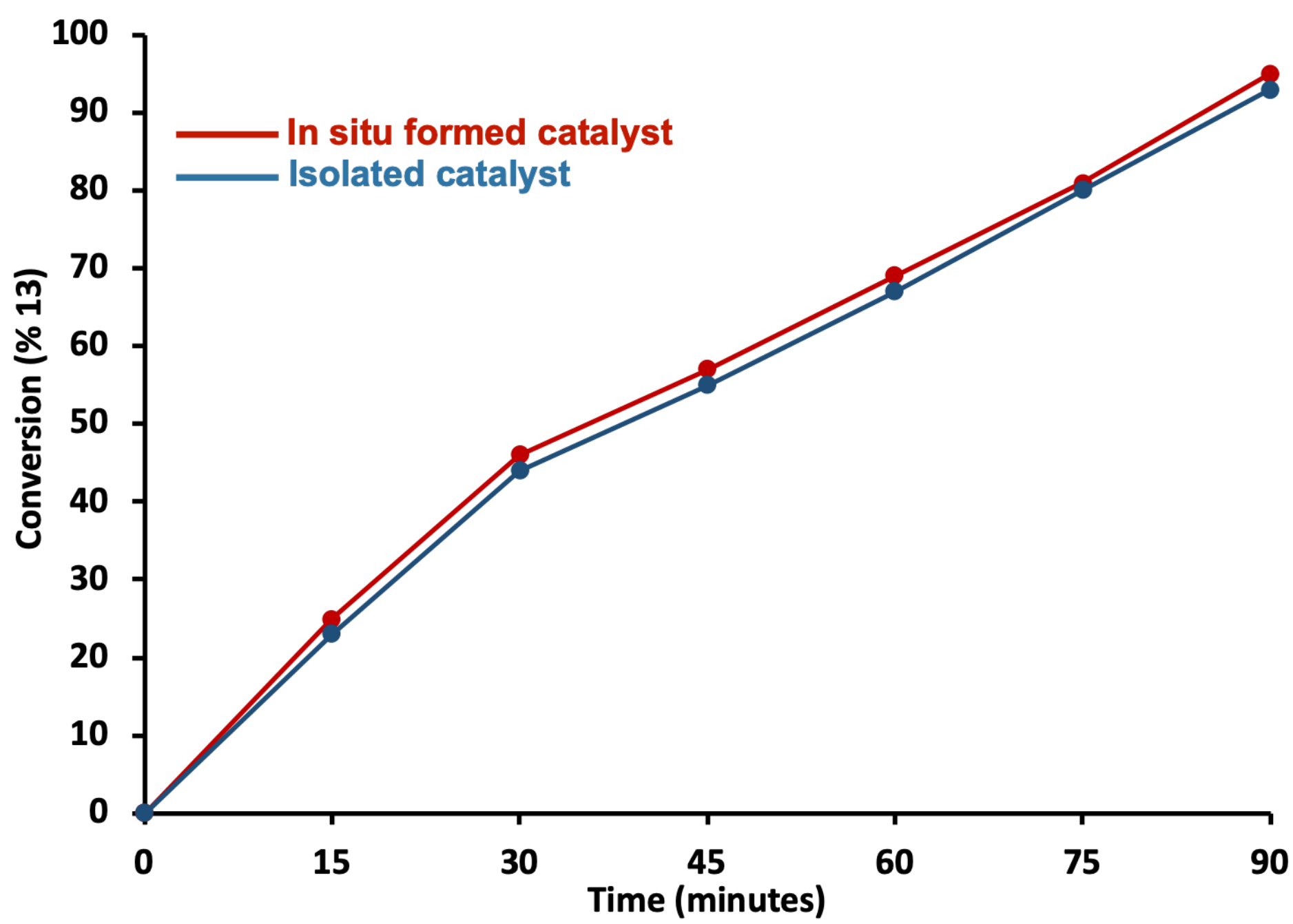

Figure S6. Reaction kinetics –in situ formed versus isolated catalyst.

12. ICP-MS ANALYSIS

Sample #: PLUMMSC1-010-EXP082-6 Test #: 1 Received: 03/13/2020 Completed: 03/16/2020

ICP-OES Palladium : 1 ppm Manganese : <1 ppm Copper : <1 ppm
Magnesium : <1 ppm

Services
ICP-OES

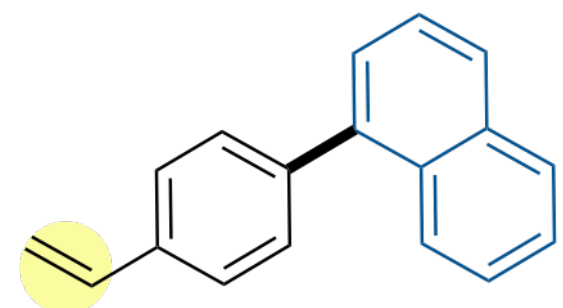

3

Sample #: PLUMMSC1-010-EXP082-7 Test #: 1 Received: 03/13/2020 Completed: 03/16/2020

ICP-OES Palladium : <1 ppm Manganese : <1 ppm Copper : 8 ppm
Magnesium : 107 ppm

Services
ICP-OES

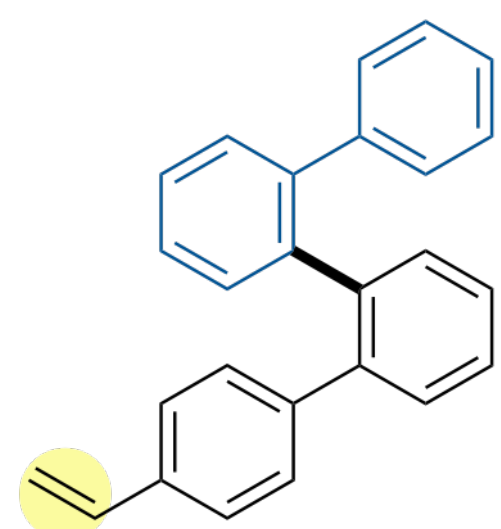

18

13. ANALYTICAL DATA

1-(4-Vinylphenyl)naphthalene (3)[2]

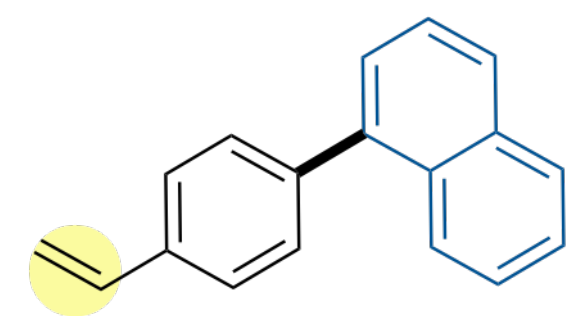

White solid, mp = 55 – 56 °C, yield 96% (55 mg), R_f 0.6 in hexane/EtOAc (9:1). **^{1}H NMR** (400 MHz, $CDCl_3$) δ 7.98 – 7.89 (m, 3H), 7.57 – 7.47 (m, 8H), 6.86 (dd, J = 20.0, 12.0 Hz, 1H), 5.88 (d, J = 20.0 Hz, 1H), 5.35 (d, J = 12.0 Hz, 1H).

2,4-Dimethoxy-4'-vinyl-1,1'-biphenyl (4)

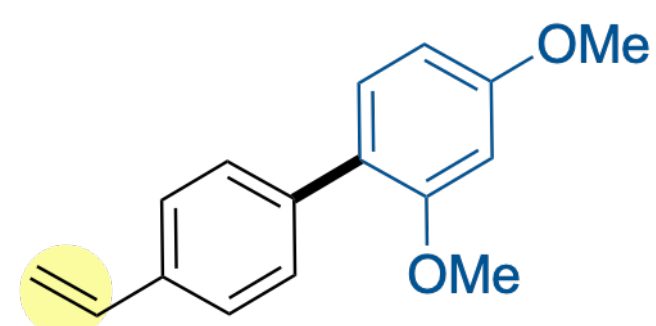

White solid, mp = 64 – 65 °C, yield 90% (54 mg), R_f 0.5 in hexane/EtOAc (9:1). **^{1}H NMR** (500 MHz, $CDCl_3$) δ 7.46 (d, J = 10.0 Hz, 2H), 7.42 (d, J = 10.0 Hz, 2H), 7.23 (d, J = 5.0 Hz, 1H), 6.73 (dd, J = 15.0, 10.0 Hz, 1H), 6.55 (d, J = 5.0 Hz, 2H), 5.73 (d, J = 20.0 Hz, 1H), 5.22 (d, J = 10.0 Hz, 1H), 3.84 (s, 3H), 3.78 (s, 3H). **^{13}C NMR** (100 MHz, $CDCl_3$) δ 160.5, 157.7, 138.1, 136.9, 136.0, 131.3, 129.7, 126.0, 123.4, 113.6, 104.8, 99.2, 55.7, 55.6. **HRMS (EI)** $[C_{16}H_{16}O_2]^+$ calcd = 240.1145, found m/z = 240.1148.

4-(4-Fluoro-2-methylphenyl)-2-vinylthiophene (5)

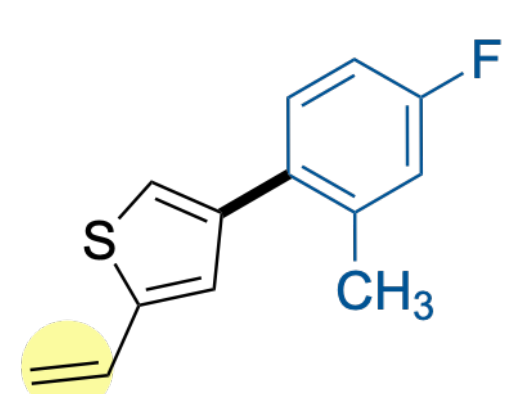

Yellow oil, yield 71% (39 mg), R_f 0.5 in hexane/EtOAc (20:1). **^{1}H NMR** (400 MHz, $CDCl_3$) δ 6.75 (t, J = 8.0 Hz, 1H), 6.51 – 6.42 (m, 4H), 6.34 (dd, J = 16.0, 8.0 Hz, 1H), 5.13 (d, J = 16.0 Hz, 1H), 4.70 (d, J = 12.0 Hz, 1H), 1.85 (s, 3H). **^{19}F NMR** (376 MHz, $CDCl_3$) δ –115.6. **^{13}C NMR**

(100 MHz, $CDCl_3$) δ 163.3, 160.9, 142.1 (d, *J* = 130.0 Hz), 138.1 (d, *J* = 10.0 Hz), 132.6, 131.1 (d, *J* = 10.0 Hz), 129.9, 127.7, 121.8, 117.1 (d, *J* = 20.0 Hz), 113.7, 112.7 (d, *J* = 20.0 Hz), 20.9. **HRMS (EI)** $[C_{13}H_{11}FS]^+$ calcd = 218.0565, found m/z = 218.0562.

3-Fluoro-4'-vinyl-1,1'-biphenyl (6)[3]

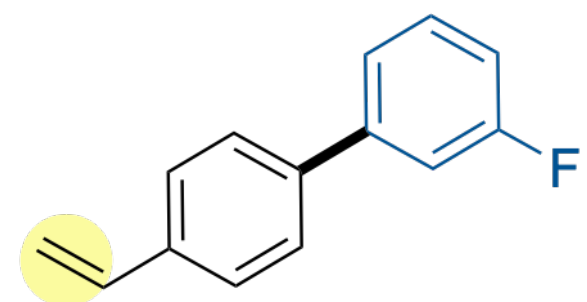

White solid, mp = 74 – 75 °C, yield 79% (39 mg), R_f 0.5 in hexane/EtOAc (20:1). **^{1}H NMR** (400 MHz, $CDCl_3$) δ 7.58 – 7.50 (m, 4H), 7.40 (d, *J* = 8.0 Hz, 2H), 7.31 (d, *J* = 12.0 Hz, 1H), 7.05 (t, *J* = 12.0, 8.0 Hz, 1H), 6.82 – 6.74 (m, 1H), 5.83 (dd, *J* = 20.0, 4.0 Hz, 1H), 5.32 (dd, *J* = 12.0, 4.0 Hz, 1H). **^{19}F NMR** (376 MHz, $CDCl_3$) δ –113.0. **^{13}C NMR** (100 MHz, $CDCl_3$) δ 164.6, 162.1, 143.1 (d, *J* = 7.0 Hz), 139.4, 137.3, 136.4, 130.4 (d, *J* = 9.0 Hz), 127.1 (d, *J* = 45.0 Hz), 122.7, 114.4 (d, *J* = 11.0 Hz), 114.1 (d, *J* = 8.0 Hz), 113.8.

4-Chloro-4'-((4-vinylphenoxy)methyl)-1,1'-biphenyl (7)

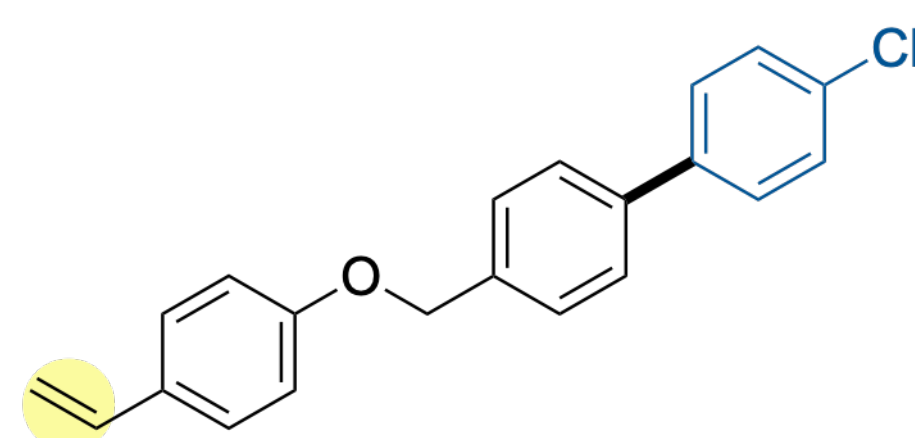

White solid, mp = 154 – 155 °C, yield 86% (69 mg), R_f 0.3 in pure hexane. **^{1}H NMR** (400 MHz, $CDCl_3$) δ 7.57 (d, *J* = 8.0 Hz, 2H), 7.51 (dd, *J* = 8.0, 4.0 Hz, 4H), 7.41 (d, *J* = 8.0 Hz, 2H), 7.36 (d, *J* = 8.0 Hz, 2H), 6.96 (d, *J* = 8.0 Hz, 2H), 6.67 (dd, *J* = 20.0, 12.0 Hz, 1H), 5.14 (d, *J* = 8.0 Hz, 1H), 5.12 (s, 3H). **^{13}C NMR** (100 MHz, $CDCl_3$) δ 158.7, 139.9, 139.4, 136.5, 136.3, 133.7, 131.9, 129.1, 128.5, 128.2, 127.6, 127.4, 115.0, 112.0, 69.9. **HRMS (EI)** $[C_{21}H_{17}ClO + H]^+$ calcd = 321.1041, found m/z = 321.1042.

2-(4'-Vinyl-[1,1'-biphenyl]-2-yl)thiophene (8)

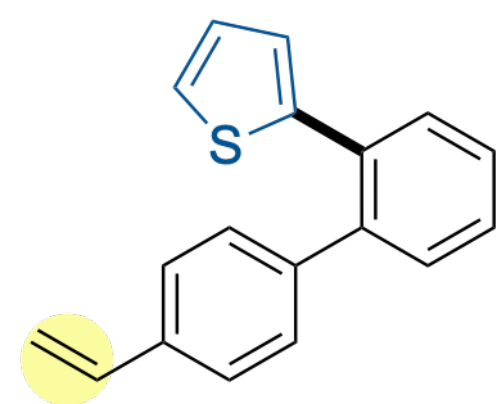

Viscous oil, yield 62% (41 mg), R_f 0.4 in hexane/EtOAc (9:1). **^{1}H NMR** (400 MHz, $CDCl_3$) δ 7.63 – 7.49 (m, 1H), 7.43 – 7.31 (m, 5H), 7.23 – 7.16 (m, 3H), 6.88 (dd, J = 5.1, 3.6 Hz, 1H), 6.80 – 6.66 (m, 2H), 5.76 (dd, J = 17.6, 0.9 Hz, 1H), 5.25 (dd, J = 10.9, 0.9 Hz, 1H). **^{13}C NMR** (100 MHz, $CDCl_3$) δ 141.2, 140.7, 137.8, 136.7, 136.3, 130.8, 130.8, 129.9, 127.9, 127.7, 127.1, 126.8, 126.2, 126.0, 125.6, 113.9. **HRMS (EI)** $[C_{18}H_{14}S + H]^+$ calcd = 262.0811, found m/z = 262.0811.

2-Chloro-3-(4-vinylphenyl)pyridine (9)

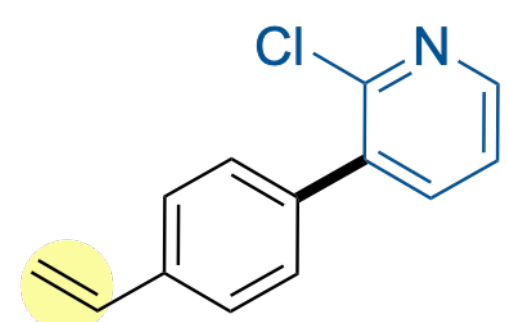

Brown solid, mp = 64 – 65 °C, yield 84% (45 mg), R_f 0.2 in hexane/EtOAc (20:1). **^{1}H NMR** (400 MHz, $CDCl_3$) δ 8.40 (d, J = 8.0 Hz, 1H), 7.67 (d, J = 8.0 Hz, 1H), 7.51 – 7. 41 (m, 4H), 7.33 – 7.29 (m, 1H), 6.77 (dd, J = 16.0, 8.0 Hz, 1H), 5.83 (d, J = 20.0 Hz, 1H), 5.33 (d, J = 12.0 Hz, 1H).**^{13}C NMR** (100 MHz, $CDCl_3$) δ 149.8, 148.5, 139.7, 137.8, 137.0, 136.8, 136.4, 129.7, 126.3, 122.7, 115.0. **HRMS (EI)** $[C_{13}H_{10}ClN + H]^+$ calcd = 216.0575, found m/z = 216.0575.

2-Chloro-4-fluoro-4''-vinyl-1,1':2',1''-terphenyl (10)

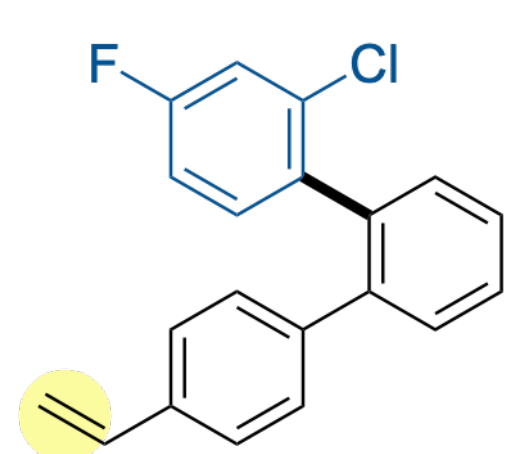

Viscous oil, yield 81% (62 mg), R_f 0.2 in hexane/EtOAc (9:1). **^{1}H NMR** (400 MHz, $CDCl_3$) δ 7.44 – 7.36 (m, 3H), 7.28 (d, J = 4.0 Hz, 1H), 7.22 (d, J = 8.0 Hz, 2H), 7.05 (d, J = 8.0 Hz, 4H), 6.83 (t, J = 16.0, 8.0 Hz, 1H), 6.62 (dd, J = 16.0, 12.0 Hz, 1H), 5.68 (d, J = 12.0 Hz, 1H), 5.18 (d, J =

12.0 Hz, 1H). **^{19}F NMR** (376 MHz, $CDCl_3$) δ –113.9. **^{13}C NMR** (100 MHz, $CDCl_3$) δ 162.9, 160.5, 141.3, 140.6, 137.1, 136.6, 136.0, 134.2 (d, J = 10.0 Hz), 133.1 (d, J = 9.0 Hz), 131.1, 130.2, 129.6, 128.5, 127.2, 125.9, 116.8 (d, J = 24.0 Hz), 113.9, 113.8. **HRMS (EI)** $[C_{20}H_{14}ClF]^+$ calcd = 308.0763, found m/z = 308.0764.

2-(4-Vinylphenyl)benzofuran (11)

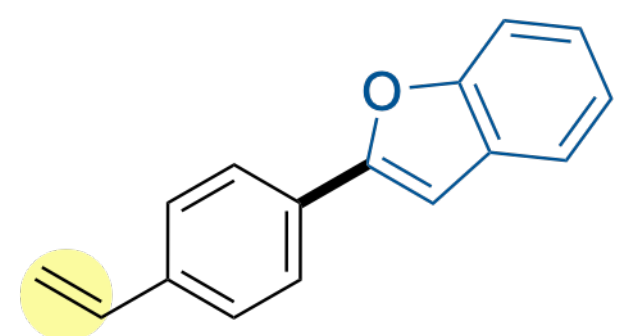

Brown oil, yield 98% (54 mg), R_f 0.4 in hexane/EtOAc (9:1). **^{1}H NMR** (400 MHz, $CDCl_3$) 7.92 (d, J = 8.0 Hz, 2H), 7.68 (d, J = 8.0 Hz, 1H), 7.63 – 7.57 (m, 3H), 7.37 – 7.31 (m, 2H), 7.11 (s, 1H), 6.85 (dd, J = 16.0, 8.0 Hz, 1H), 5.91 (d, J = 16.0 Hz, 1H), 5.41 (d, J = 12.0 Hz, 1H) **^{13}C NMR** (100 MHz, $CDCl_3$) δ 155.8, 155.0, 137.9, 136.4. 129.9, 129.4, 126.8, 125.2, 124.4, 123.0, 121.0, 114.6, 111.3, 101.5. **HRMS (EI)** $[C_{16}H_{12}O]^+$ calcd = 220.0883, found m/z = 220.0886

4-(2-Fluorophenyl)-2-vinylthiophene (12)

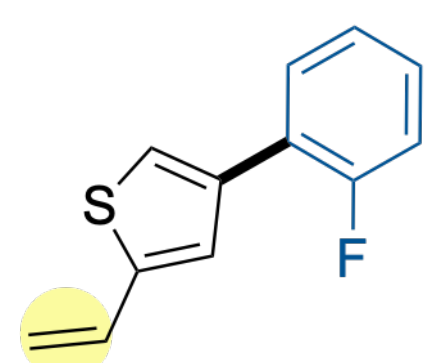

Yellow oil, yield 96% (49 mg), R_f 0.32 in pure hexane. **^{1}H NMR** (400 MHz, $CDCl_3$) δ 7.82 (td, J = 16.0, 8.0 Hz, 1H), 7.72 (s, 1H), 7.59 (s, 1H), 7.54 (q, J = 8.0 Hz, 1H), 7.47 – 7.39 (m, 2H), 7.13 (dd, J = 20.0, 12.0 Hz, 1H), 5.90 (d, J = 20.0 Hz, 1H), 5.48 (d, J = 12.0 Hz, 1H). **^{19}F NMR** (376 MHz, $CDCl_3$) δ –115.3. **^{13}C NMR** (100 MHz, $CDCl_3$) δ 163.3, 160.9, 142.1 (d, J = 126.0 Hz), 138.1 (d, J = 7.0 Hz), 132.6, 131.1 (d, J = 8.0 Hz), 129.9, 127.7, 121.8, 117.1 (d, J = 21.0 Hz), 113.7, 112.7 (d, J = 21.0 Hz). **HRMS (EI)** $[C_{12}H_9FS + H]^+$ calcd = 205.0482, found m/z = 205.0481.

3-(4-Vinylphenyl)furan (13)

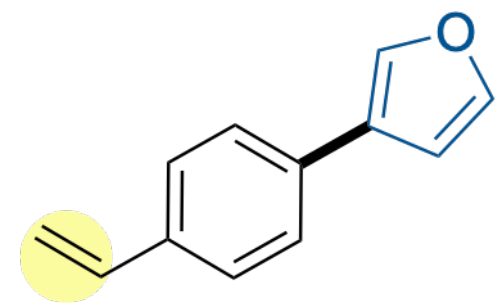

Yellow solid, mp = 91 – 92 °C, yield 95% (40 mg), R_f 0.5 in hexane/EtOAc (9:1). **^{1}H NMR** (400 MHz, $CDCl_3$) δ 7.74 (s, 1H), 7.48 – 7.43 (m, 5H), 6.72 (dd, *J* = 20.0, 12.0 Hz, 2H), 5.76 (d, *J* = 16.0 Hz, 1H), 5.24 (d, *J* = 12.0 Hz, 1H). **^{13}C NMR** (100 MHz, $CDCl_3$) δ 143.8, 138.7, 136.6, 136.5, 132.0, 126.8, 126.3, 126.1, 113.7, 108.9. **HRMS (EI)** $[C_{12}H_{10}O]^+$ calcd = 170.0726 found m/z = 170.0728.

1-(4-Vinylphenyl)pyrene (14)

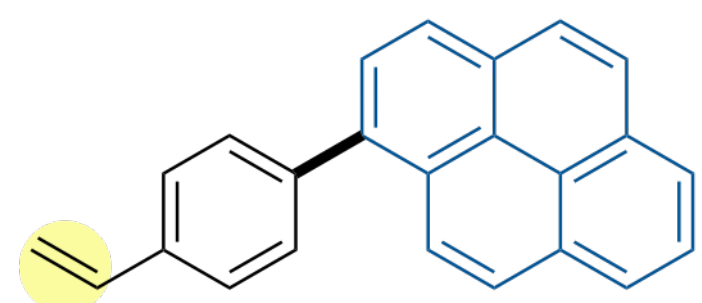

Light green solid, mp = 105 °C, yield 88% (67 mg), R_f 0.5 in hexane/EtOAc (20:1). **^{1}H NMR** (400 MHz, $CDCl_3$) δ 8.24 – 8.16 (m, 4H), 8.09 (d, *J* = 4.0 Hz, 2H), 7.99 (q, *J* = 16.0, 8.0 Hz, 3H), 7.62 (s, 4H), 6.88 (dd, *J* = 20.0, 16.0 Hz, 1H), 5.89 (d, *J* = 20.0 Hz, 1H), 5.36 (d, *J* = 8.0 Hz, 1H). **^{13}C NMR** (100 MHz, $CDCl_3$) δ 140.9, 137.5, 136.7, 131.0, 127.7, 127.6, 127.6, 126.6, 126.2, 125.4, 125.3, 125.0, 124.8, 114.3. **HRMS (EI)** $[C_{24}H_{16}]^+$ calcd = 304.1247, found m/z = 304.1251.

1-((4'-Chloro-[1,1'-biphenyl]-4-yl)methyl)-5-vinyl-1*H*-indole (15)

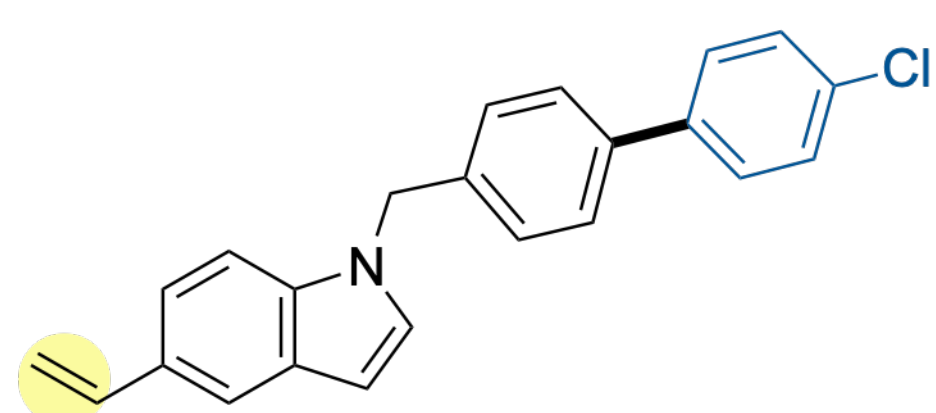

White solid, mp = 161 – 162 °C, yield 93% (79 mg), R_f 0.4 in hexane/EtOAc (9:1). **^{1}H NMR** (400 MHz, $CDCl_3$) δ 7.32 (s, 1H), 7.12 – 7.09 (m, 4H), 7.09 – 6.97 (m, 3H), 6.89 (d, *J* = 8.0 Hz, 1H), 6.82 – 6.78 (m, 3H), 6.48 (dd, *J* = 16.0, 8.0 Hz, 1H), 6.21 (d, *J* = 4.0 Hz, 1H), 5.34 (d, *J* = 20.0 Hz, 1H), 4.99 (s, 2H), 4.79 (d, *J* = 12.0 Hz, 1H). **^{13}C NMR** (100 MHz, $CDCl_3$) δ 139.5, 139.2, 138.0,

137.0, 136.3, 133.7, 129.8, 129.1, 128.9, 128.4, 127.5, 127.4, 120.2, 119.7, 111.2, 109.9, 102.4, 50.0. **HRMS (EI)** $[C_{23}H_{18}ClN + H]^+$ calcd = 344.1201, found m/z = 344.1202.

4'-((5-Vinyl-1*H*-indol-1-yl)methyl)-[1,1'-biphenyl]-4-carbaldehyde (16)

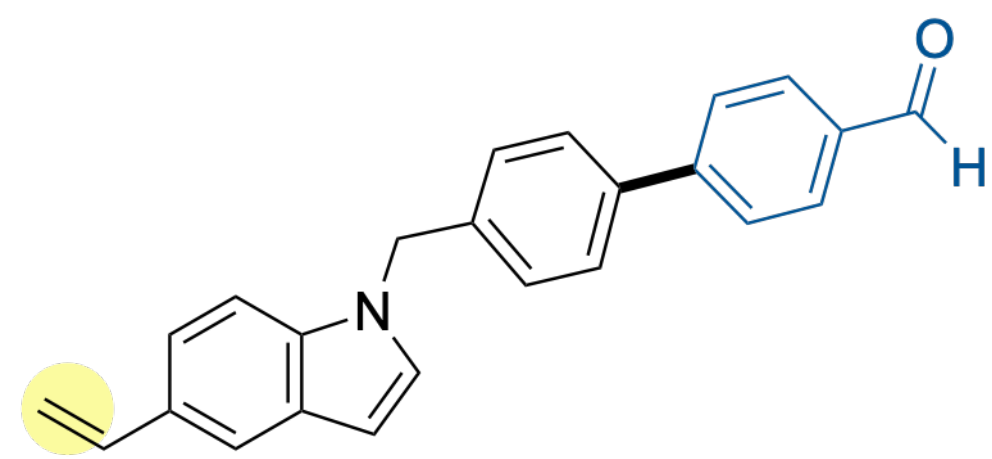

Yellow solid, mp = 138 – 139 °C, yield 89% (75 mg), R_f 0.2 in hexane/EtOAc (20:1). ^{1}**H NMR** (400 MHz, $CDCl_3$) δ 9.49 (s, 1H), 7.38 (d, *J* = 8.0 Hz, 2H), 7.14 (t, *J* = 8.0, 4.0 Hz, 3H), 7.0 (d, *J* = 8.0 Hz, 2H), 6.80 (dd, *J* = 8.0, 4.0 Hz, 1H), 6.71 – 6.80 (m, 4H), 6.30 (dd, *J* = 16.0, 12.0 Hz, 1H), 6.03 (d, *J* = 4.0 Hz, 1H), 5.17 (d, *J* = 20.0 Hz, 1H), 4.81 (s, 2H), 4.61 (d, *J* = 12.0 Hz, 1H). ^{13}**C NMR** (100 MHz, $CDCl_3$) δ 192.0, 146.6, 139.2, 138.0, 137.9, 136.3, 135.4, 130.4, 129.8, 129.1, 128.9, 127.9, 127.7, 127.5, 120.2, 119.7, 111.2, 109.9, 102.5, 50.0. **HRMS (EI)** $[C_{24}H_{19}NO + H]^+$ calcd = 338.1539, found m/z = 338.1540.

1-((4'-(Trifluoromethyl)-[1,1'-biphenyl]-4-yl)methyl)-2-vinyl-1*H*-indole (17)

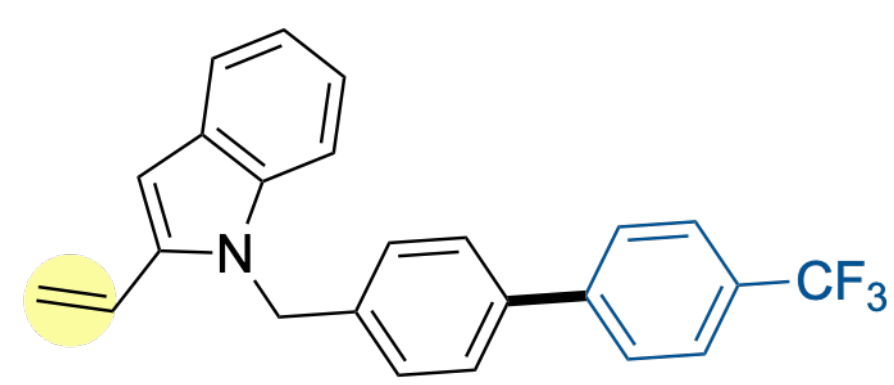

White solid, yield 83% (78 mg), R_f 0.5 in hexane/EtOAc (20:1). ^{1}**H NMR** (400 MHz, $CDCl_3$) δ 7.75 – 7.69 (m, 5H), 7.57 (d, *J* = 8.0 Hz, 2H), 7.34 – 7.32 (m, 1H), 7.24 – 7.18 (m, 4H), 6.88 (s, 1H), 6.81 (dd, *J* = 16.0, 12.0 Hz, 1H), 5.91 (d, *J* = 20.0 Hz, 1H), 5.53 (s, 2H), 5.39 (d, *J* = 16.0 Hz, 1H). ^{19}**F NMR** (376 MHz, $CDCl_3$) δ –63.0. ^{13}**C NMR** (100 MHz, $CDCl_3$) δ 144.2, 139.0, 138.4, 138.0, 137.8, 129.5 (q, *J* = 33.0 Hz), 128.2, 127.8, 127.4, 126.8, 126.6, 125.9, 122.7 (q, *J* = 272.0 Hz), 122.3, 120.9, 120.4, 117.0, 109.7, 99.9, 46.4. **HRMS (EI)** $[C_{24}H_{18}F_3N]^+$ calcd = 377.1386, found (m/z) = 377.1387.

4-Vinyl-1,1':2',1'':2'',1'''-quaterphenyl (18)

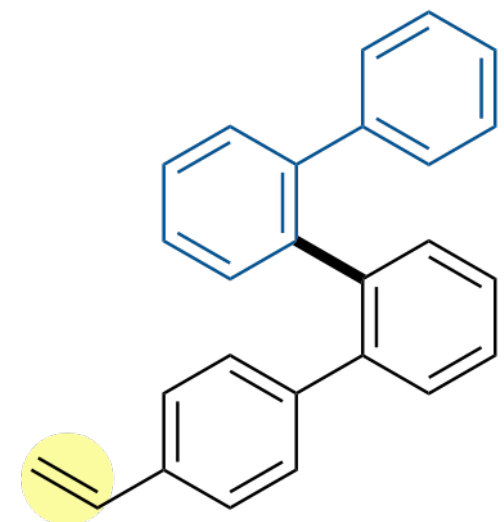

White solid, mp = 65 °C yield 93% (77 mg), R_f 0.5 in hexane/EtOAc (20:1). **^{1}H NMR** (400 MHz, $CDCl_3$) δ 7.44 – 7.31 (m, 6H), 7.20 – 7.16 (m, 2H), 7.09 (q, *J* = 12.0, 8.0 Hz, 3H), 7.00 (t, *J* = 8.0 Hz, 2H), 6.68 – 6.57 (m, 5H), 5.68 (d, *J* = 16.0 Hz, 1H), 5.19 (d, *J* = 12.0 Hz, 1H). **^{13}C NMR** (100 MHz, $CDCl_3$) δ 141.1, 141.1, 140.8, 140.7, 140.1, 136.9, 135.3, 131.9, 131.8, 130.2, 129.9, 129.4, 127.6, 127.6, 127.3, 126.1, 125.5, 113.4. **HRMS (EI)** $[C_{26}H_{20}]^+$ calcd = 332.1560, found m/z = 332.1560.

5-(4-Vinylphenyl)thiophene-2-carbaldehyde (19)

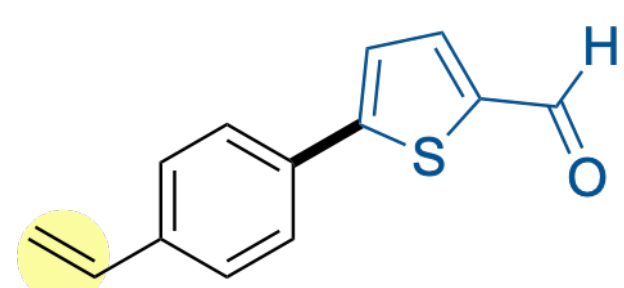

Yellow viscous oil, yield 72% (39 mg), R_f 0.5 in hexane/EtOAc (4:1). **^{1}H NMR** (400 MHz, $CDCl_3$) δ 9.89 (s, 1H), 7.74 (d, *J* = 4.0 Hz, 1H), 7.65 (d, *J* = 8.4 Hz, 2H), 7.47 (d, *J* = 8.3 Hz, 2H), 7.41 (d, *J* = 4.0 Hz, 1H), 6.74 (dd, *J* = 17.6, 10.9 Hz, 1H), 5.83 (dd, *J* = 17.6, 0.8 Hz, 1H), 5.34 (dd, *J* = 10.8, 0.8 Hz, 1H). **^{13}C NMR** (100 MHz, $CDCl_3$) δ 182.9, 169.6, 167.6, 138.9, 137.6, 136.1, 127.1, 126.7, 124.1, 115.3, 110.1. **HRMS (APCI)** $[C_{13}H_{10}OS +H]^+$ calcd = 215.0525, found m/z = 215.0526.

3-(5-Vinylthiophen-3-yl)furan (20)

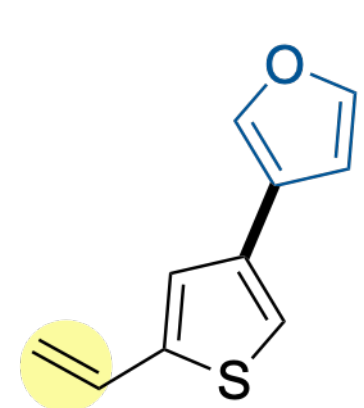

Brown oil, yield 63% (28 mg), R_f 0.45 in pure hexane. **^{1}H NMR** (400 MHz, $CDCl_3$) δ 7.64 (s, 1H), 7.44 (s, 1H), 7.09 (d, *J* = 8.0 Hz, 2H), 6.80 (dd, *J* = 20.0, 12.0 Hz, 1H), 6.60 (s, 1H), 5.59 (d, *J* = 20.0 Hz, 1H), 5.18 (d, *J* = 8.0 Hz, 1H). **^{13}C NMR** (100 MHz, $CDCl_3$) δ 143.8, 143.6, 138.5, 133.6, 129.9, 124.7, 121.9, 118.5, 114.0, 109.2. **HRMS (EI)** $[C_{10}H_8OS]^+$ calcd = 176.0296, found m/z = 176.0286.

1-Benzyl-5-(naphthalen-1-yl)-3-vinyl-1*H*-indole (21)

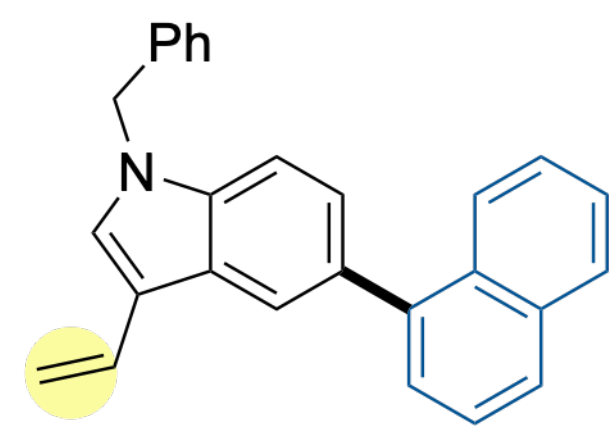

Brown oil, yield 91% (111 mg), R_f 0.4 in hexane/EtOAc (20:1). **^{1}H NMR** (400 MHz, $CDCl_3$) δ 8.00 – 7.96 (m, 2H), 7.88 (dd, *J* = 24.0, 8.0 Hz, 2H), 7.53 – 7.46 (m, 4H), 7.32 – 7.30 (m, 5H), 7.23 (dd, *J* = 16.0, 4.0 Hz, 3H), 6.89 (dd, *J* = 16.0, 8.0 Hz, 1H), 5.67 (d, *J* = 16.0 Hz, 1H), 5.35 (s, 2H), 5.12 (d, *J* = 12.0 Hz, 1H). **^{13}C NMR** (100 MHz, $CDCl_3$) δ 141.5, 137.2, 136.7, 134.0, 133.0, 132.4, 129.3, 129.1, 128.3, 128.3, 128.0, 127.5, 127.3, 127.2, 126.7, 126, 125.8, 125.6, 125.0, 121.9, 115.3, 110.8, 109.7, 50.4. **HRMS (EI)** $[C_{27}H_{21}N + H]^+$ calcd = 360.1747, found m/z = 360.1750.

3-(4-((4-Vinylphenoxy)methyl)phenyl)furan (22)

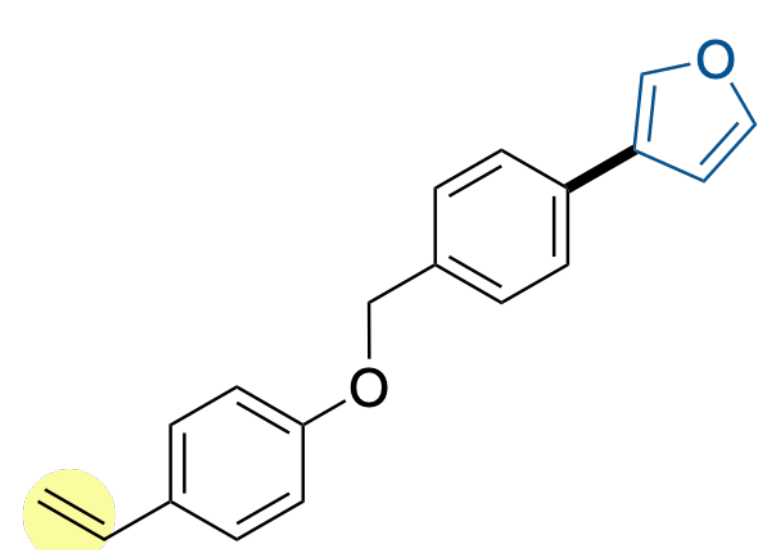

White solid, mp = 158 – 159 °C, yield 73% (50 mg), R_f 0.6 in hexane/EtOAc (20:1). **^{1}H NMR** (400 MHz, $CDCl_3$) δ 7.74 (s, 1H), 7.52 – 7.48 (m, 3H), 7.44 (d, *J* = 8.0 Hz, 2H), 7.35 (d, *J* = 12.0 Hz, 2H), 6.95 (d, *J* = 12.0 Hz, 2H), 6.71 – 6.63 (m, 2H), 5.62 (d, *J* = 16.0 Hz, 1H), 5.14 (d, *J* = 12.0 Hz, 1H), 5.08 (s, 2H). **^{13}C NMR** (100 MHz, $CDCl_3$) δ 158.7, 143.9, 138.9, 138.7, 136.4,

135.8, 132.4, 130.9, 128.2, 127.6, 126.2, 115.0, 111.9, 109.0, 70.0. **HRMS (EI)** $[C_{19}H_{16}O_2 + H]^+$ calcd = 277.1223, found m/z = 277.1224.

2-Methoxy-1-(4'-vinyl-[1,1'-biphenyl]-2-yl)naphthalene (23)

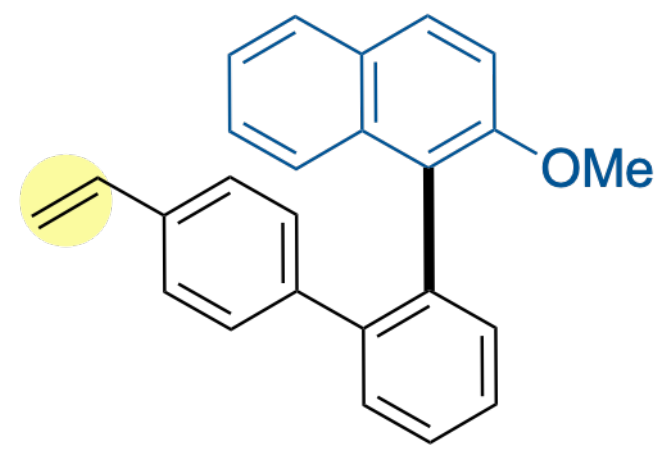

White solid, mp = 87 °C, yield 54% (45 mg), R_f 0.6 in hexane/EtOAc (9:1) **^{1}H NMR** (400 MHz, $CDCl_3$) δ 7.94 – 7.70 (m, 2H), 7.57 – 7.45 (m, 4H), 7.40 – 7.30 (m, 3H), 7.17 – 7.07 (m, 3H), 7.02 (d, J = 8.0 Hz, 2H), 6.57 (dd, J = 17.6, 10.9 Hz, 1H), 5.61 (dd, J = 17.6, 1.0 Hz, 1H), 5.13 (dd, J = 10.9, 1.0 Hz, 1H), 3.54 (s, 3H). **^{13}C NMR** (100 MHz, $CDCl_3$) δ 153.6, 142.7, 141.6, 136.7, 135.5, 134.9, 134.0, 132.1, 129.9, 128.8, 128.1, 127.9, 127.3, 126.5, 125.4, 125.3, 124.5, 123.5, 113.4, 113.3, 56.3. **HRMS (EI)** $[C_{25}H_{20}O + H]^+$ calcd = 337.1587, found m/z = 337.1587.

1-Benzyl-5-(2-methoxyphenyl)-3-vinyl-1*H*-indole (24)

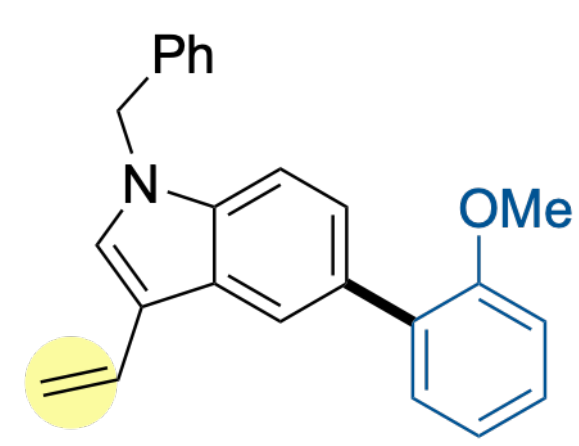

Yellow oil, yield 84% (71 mg), R_f 0.4 in hexane/EtOAc (20:1). **^{1}H NMR** (400 MHz, $CDCl_3$) δ 8.02 (s, 1H), 7.40 (d, J = 8.0 Hz, 2H), 7.35 – 7.28 (m, 5H), 7.22 – 7.16 (m, 3H), 7.07 – 7.00 (m, 2H), 6.89 (dd, J = 16.0, 12.0 Hz, 1H), 5.70 (d, J = 16.0 Hz, 1H), 5.31 (s, 2H), 5.14 (d, J = 12.0 Hz, 1H), 3.82 (s, 3H). **^{13}C NMR** (100 MHz, $CDCl_3$) δ 156.8, 137.3, 136.6, 132.1, 131.5, 130.8, 129.5, 128.2, 127.9, 127.2, 126.6, 124.5, 121.4, 121.0, 115.4, 111.4, 110.5, 109.5, 55.8, 50.3. **HRMS (EI)** $[C_{24}H_{21}NO + H]^+$ calcd = 340.1698, found m/z = 340.1700.

3,5-Dimethyl-4-(4-vinylphenyl)isoxazole (25)

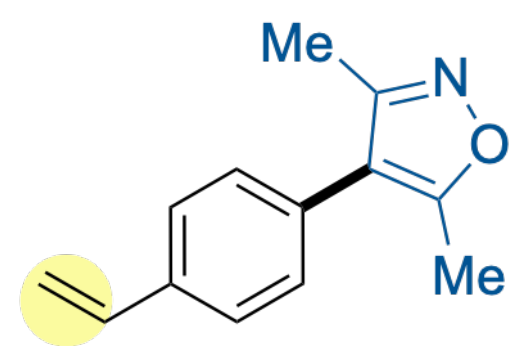

Viscous yellow gel, yield 72% (35 mg), R_f 0.5 in hexane/EtOAc (4:1). **^{1}H NMR** (400 MHz, $CDCl_3$) δ 7.48 (d, *J* = 8.2 Hz, 2H), 7.22 (d, *J* = 8.2 Hz, 2H), 6.75 (dd, *J* = 17.6, 10.9 Hz, 1H), 5.80 (d, *J* = 17.6 Hz, 1H), 5.30 (d, *J* = 10.9 Hz, 1H), 2.41 (s, 3H), 2.28 (s, 3H). **^{13}C NMR** (100 MHz, $CDCl_3$) δ 158.8, 137.0, 136.3, 130.0, 129.3, 128.7, 128.3, 126.7, 114.6, 11.7, 11.0. **HRMS (EI)** $[C_{13}H_{13}NO]^+$ calcd = 199.0992, found m/z = 199.0994.

2-(Benzyloxy)-4-fluoro-4'-((4-vinylphenoxy)methyl)-1,1'-biphenyl (26)

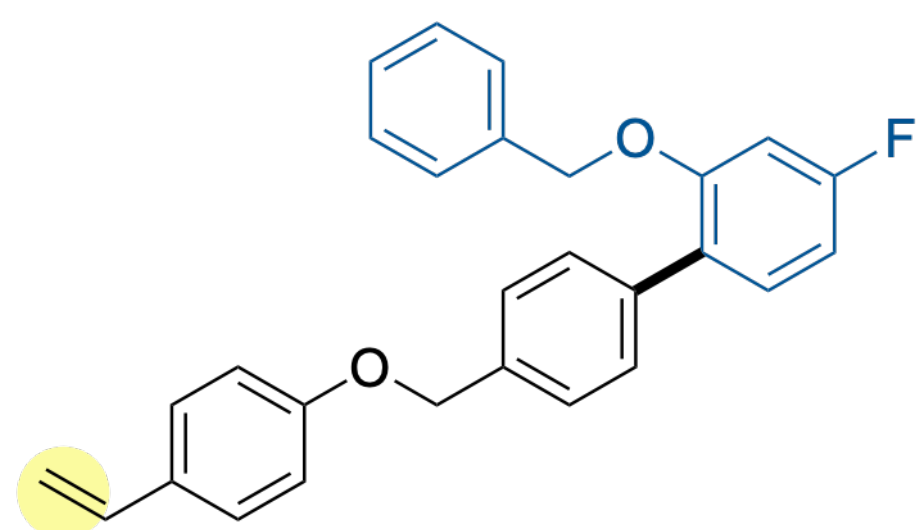

White solid, mp = 107 °C, yield 78% (80 mg), R_f 0.6 in hexane/EtOAc (9:1) **^{1}H NMR** (400 MHz, $CDCl_3$) δ 7.56 (d, *J* = 8.0 Hz, 2H), 7.46 (d, *J* = 8.0 Hz, 2H), 7.38 – 7.28 (m, 8H), 6.97 (d, *J* = 8.0 Hz, 2H), 6.76 (d, *J* = 12.0 Hz, 2H), 6.67 (dd, *J* = 16.0, 8.0 Hz, 1H), 5.62 (d, *J* = 20.0 Hz, 1H), 5.14 (d, *J* = 12.0 Hz, 1H), 5.11 (s, 2H), 5.08 (s, 2H). **^{19}F NMR** (376 MHz, $CDCl_3$) δ –112.8. **^{13}C NMR** (100 MHz, $CDCl_3$) δ 164.3, 161.9, 158.8, 156.7 (d, *J* = 10.0 Hz), 137.6, 136.6, 136.4, 135.7, 131.8 (d, *J* = 10.0 Hz), 130.9, 129.9, 128.7, 128.0, 127.6, 127.3, 127.0, 115.1, 111.9, 107.8 (d, *J* = 21.0 Hz), 101.4 (d, *J* = 25.0 Hz), 70.7, 70.1. **HRMS (EI)** $[C_{28}H_{23}FO_2 +H]^+$ calcd = 411.1755, found m/z = 411.1755.

4-Nitro-4'-((4-vinylphenoxy)methyl)-1,1'-biphenyl (27)

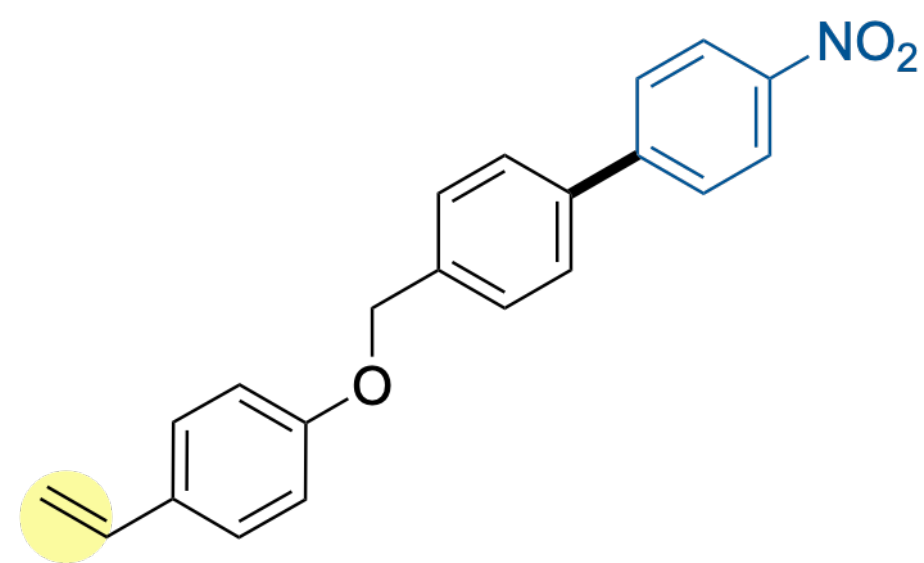

Yellow solid, mp = 160 – 161 °C, yield 85% (70 mg), R_f 0.2 in pure hexane. **^{1}H NMR** (400 MHz, $CDCl_3$) δ 8.37 (d, J = 8.0 Hz, 1H), 8.31 (d, J = 8.0 Hz, 1H), 7.79 (d, J = 8.0 Hz, 1H), 7.74 (d, J = 8.0 Hz, 2H), 7.65 (d, J = 8.0 Hz, 2H), 7.57 (d, J = 8.0 Hz, 2H), 7.36 (d, J = 4.0 Hz, 2H), 6.65 (d, J = 8.0 Hz, 1H), 6.67 (dd, J = 20.0, 12.0 Hz, 1H), 5.62 (d, J = 16.0 Hz, 1H), 5.13 (d, J = 8.0 Hz, 3H). **^{13}C NMR** (100 MHz, $CDCl_3$) δ 147.3, 138.6, 138.1, 136.3, 131.1, 128.5, 128.3, 128.0, 127.8, 127.6, 124.6, 124.3, 115.1, 112.1, 69.7. **HRMS (EI)** $[C_{21}H_{17}NO_3 + H]^+$ calcd = 332.1280, found m/z = 332.1281.

1-(2-(1-Phenylvinyl)phenyl)naphthalene (28)

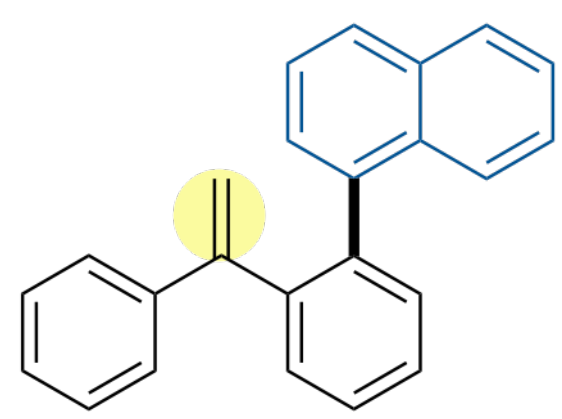

Brown-oil, yield 60% (46 mg), R_f 0.4 in hexane/EtOAc (20:1). **^{1}H NMR** (400 MHz, $CDCl_3$) δ 7.75 (d, J = 8.0 Hz, 1H), 7.64 (d, J = 8.0 Hz, 1H), 7.55 – 7.46 (m, 4H), 7.39 (t, J = 16.0, 8.0 Hz, 2H), 7.30 (t, J = 16.0, 8.0 Hz, 1H), 7.26 – 7.21 (m, 1H), 7.10 (d, J = 8.0 Hz, 1H), 6.94 – 6.91 (m, 3H), 6.86 – 6.83 (m, 2H), 5.29 (s, 1H), 5.16 (s, 1H). **^{13}C NMR** (100 MHz, $CDCl_3$) δ 150.0, 142.5, 141.6, 139.8, 139.3, 133.4, 132, 131.5, 130.6, 128.0, 127.6, 127.6, 127.4, 127.2, 127.0, 126.9, 126.7, 125.4, 124.9, 116.2. **HRMS (EI)** $[C_{24}H_{15} + H]^+$ calcd = 304.1247, found m/z = 304.1251.

4-(Trifluoromethyl)-4'-((4-vinylphenoxy)methyl)-1,1'-biphenyl (29)

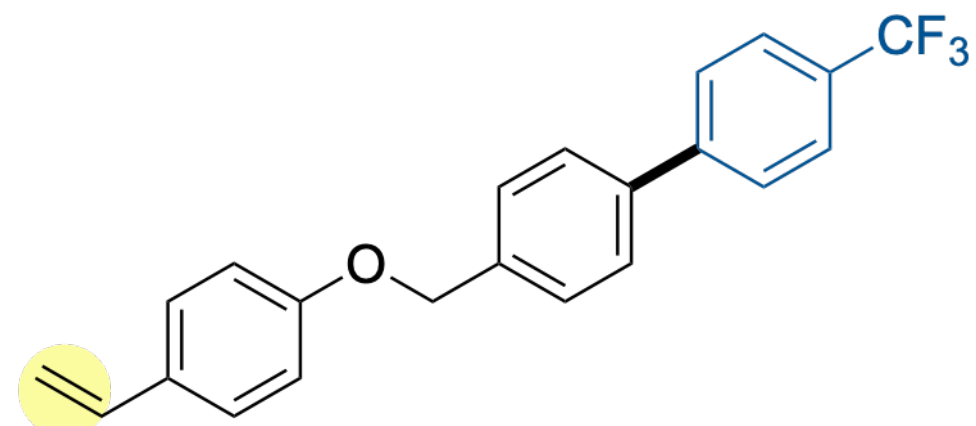

White solid, mp = 173 – 174 °C, yield 87% (77 mg), R_f 0.3 in pure hexane. **^{1}H NMR** (400 MHz, $CDCl_3$) δ 7.70 (s, 4H), 7.62 (d, J = 8.0 Hz, 2H), 7.54 (d, J = 8.0 Hz, 2H), 7.37 (d, J = 8.0 Hz, 2H), 6.96 (d, J = 8.0 Hz, 2H), 6.68 (dd, J = 16.0, 12.0 Hz, 1H), 5.63 (d, J = 16.0 Hz, 1H), 5.15 (d, J = 8.0 Hz, 1H), 5.14 (s, 2H). **^{19}F NMR** (376 MHz, $CDCl_3$) δ –62.47 ppm. **^{13}C NMR** (100 MHz, $CDCl_3$) δ 158.6, 144.4, 139.6, 137.2, 136.3, 130.9, 129.6 (q, J = 32.0 Hz), 128.2, 127.6, 127.6, 127.5, 127.2 (q, J = 272.0 Hz), 125.9 (q, J = 3.0 Hz), 115.0, 111.9, 69.8. **HRMS (EI)** $[C_{22}H_{17}F_3O + H]^+$ calcd = 355.1304, found m/z = 355.1304.

1-(4-((4-Vinylphenoxy)methyl)phenyl)pyrene (30)

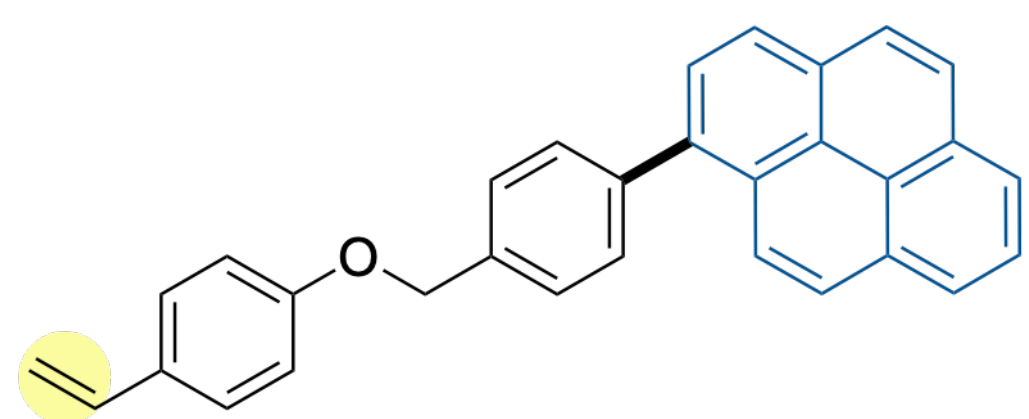

Off-white solid, mp = 158 ºC, yield 74% (76 mg), R_f 0.6 in hexane/EtOAc (20:1). **^{1}H NMR** (400 MHz, $CDCl_3$) δ 8.24 – 8.17 (m, 4H), 8.11 (s, 2H), 8.05 – 7.98 (m, 3H), 7.65 (q, J = 12.0, 8.0 Hz, 4H), 7.41 (d, J = 8.0 Hz, 2H), 7.03 (d, J = 12.0 Hz, 2H), 6.70 (dd, J = 16.0, 12.0 Hz, 1H), 5.65 (d, J = 20.0 Hz, 1H), 5.22 (s, 2H), 5.17 (d, J = 12.0 Hz, 1H).**^{13}C NMR** (100 MHz, $CDCl_3$) δ 158.8, 141.2, 137.4, 136.4, 136.1, 131.7, 131.1, 131.0, 130.8, 127.8, 127.7, 127.7, 127.6, 126.2, 125.4, 125.3, 125.1, 125.0, 124.8, 115.1, 112.0, 70.1. **HRMS (EI)** $[C_{31}H_{22}O + H]^+$ calcd = 411.1743, found m/z = 411.1743.

2-Methoxy-4-(2-(1-(4-nitrophenyl)vinyl)phenyl)pyridine (31)

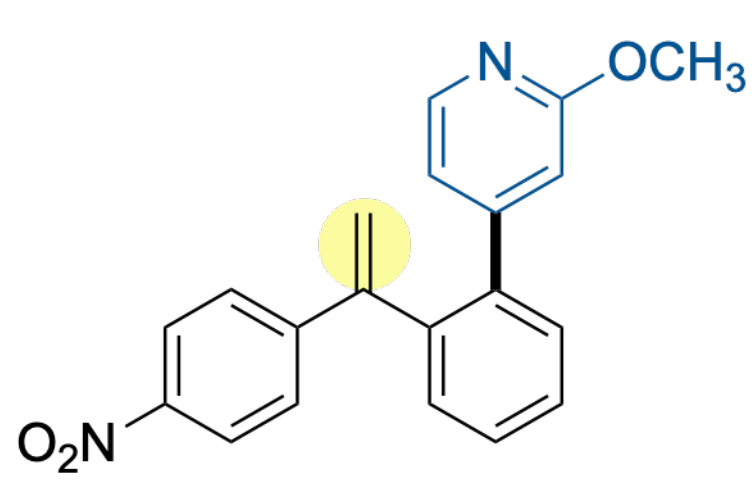

Yellow oil, yield 71% (58 mg), R_f 0.5 in hexane/EtOAc (7:3). **^{1}H NMR** (400 MHz, $CDCl_3$) δ 7.95 (d, *J* = 4.0 Hz, 3H), 7.45 – 7.44 (m, 3H), 7.35 – 7.30 (m, 2H), 7.15 (d, *J* = 12.0 Hz, 2H), 6.50 (d, *J* = 8.0 Hz, 1H), 5.76 (s, 1H), 5.54 (s, 1H), 3.85 (s, 3H). **^{13}C NMR** (100 MHz, $CDCl_3$) δ 163.1, 148.4, 147.7, 147.0, 146.8, 139.9, 139.2, 137.6, 131.1, 130.5, 130.0, 129.0, 128.1, 127.6, 123.4, 120.2, 110.1, 53.6. **HRMS (EI)** $[C_{20}H_{16}N_2O_3 + H]^+$ calcd = 333.1234, found m/z = 333.1236.

4-Vinyl-4'-((4-vinylphenoxy)methyl)-1,1'-biphenyl (32)

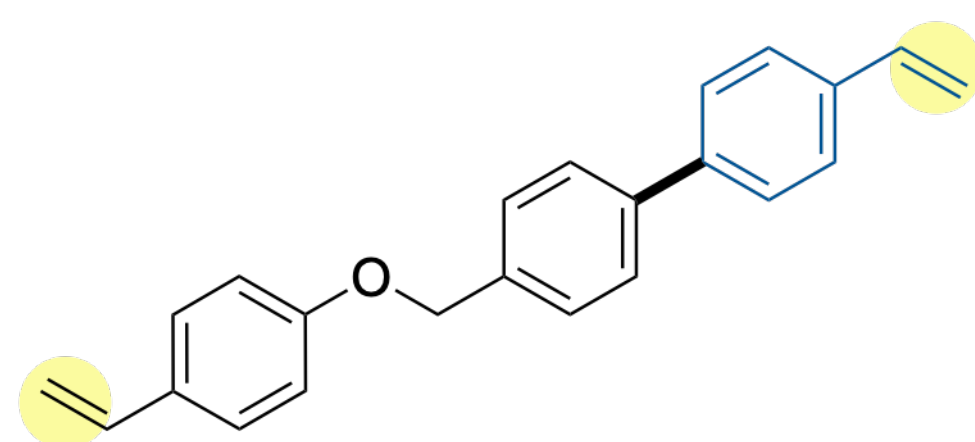

Light yellow solid, mp = 174 – 175 °C, yield 98% (77 mg), R_f 0.3 in hexane/EtOAc (20:1). **^{1}H NMR** (400 MHz, $CDCl_3$) δ 7.62 (d, *J* = 8.0 Hz, 2H), 7.57 (d, *J* = 8.0 Hz, 2H), 7.49 (dd, *J* = 7.6, 3.6 Hz, 4H), 7.37 (d, *J* = 8.4 Hz, 2H), 6.96 (d, *J* = 9.2 Hz, 2H), 6.72 (m, 2H), 5.80 (d, *J* = 17.6 Hz, 1H), 5.62 (d, *J* = 17.2 Hz, 1H), 5.29 (d, *J* = 11.2 Hz, 1H), 5.14 (d, *J* = 11.2 Hz, 1H), 5.12 (s, 2H). **^{13}C NMR** (100 MHz, $CDCl_3$) δ 158.7, 140.7, 140.3, 136.9, 136.5, 136.3, 136.1, 130.9, 128.1, 127.6, 127.3, 126.8, 115.0, 114.1, 111.9, 70.6, 69.9. **HRMS (EI)** $[C_{23}H_{20}O + H]^+$ calcd = 313.1587, found m/z = 313.1589.

2,4-Dichloro-4'-vinyl-1,1'-biphenyl (33)

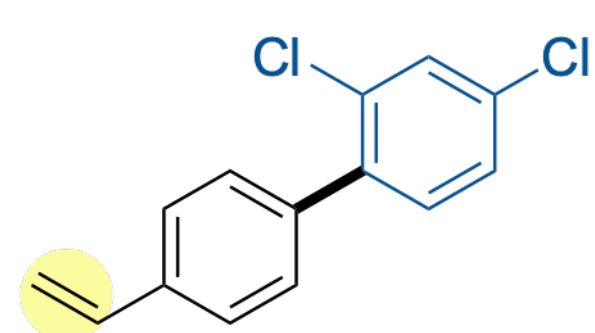

Viscous gel, yield 85% (53 mg), R_f 0.5 in hexane/EtOAc (20:1). **^{1}H NMR** (400 MHz, $CDCl_3$) δ 7.48 (d, *J* = 8.0 Hz, 3H), 7.38 (d, *J* = 8.0 Hz, 2H), 7.32 – 7.28 (m, 2H), 6.76 (dd, *J* = 16.0, 12.0 Hz, 1H), 6.82 (d, *J* = 20.0 Hz, 1H), 5.31 (d, *J* = 12.0 Hz, 1H). **^{13}C NMR** (100 MHz, $CDCl_3$) δ 138.9, 137.8, 137.4, 136.5, 133.9, 133.4, 132.2, 129.9, 129.7, 127.4, 126.2, 114.7. **HRMS (EI)** $[C_{14}H_{10}Cl_2]^+$ calcd = 248.0154, found m/z = 248.0156.

1-(4-(Naphthalen-1-yl)benzyl)-5-vinyl-1*H*-indole (34)

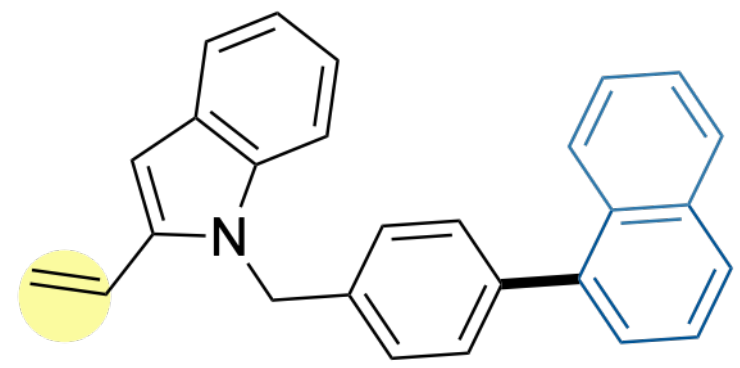

Viscous gel, yield 81% (73 mg), R_f 0.4 in hexane/EtOAc (9:1). ^{1}**H NMR** (400 MHz, $CDCl_3$) δ 7.92 (d, *J* = 8.3 Hz, 1H), 7.87 (d, *J* = 8.3 Hz, 2H), 7.71 (s, 1H), 7.51 (dd, *J* = 15.5, 7.7 Hz, 2H), 7.47 – 7.32 (m, 6H), 7.33 – 7.18 (m, 3H), 6.88 (dd, *J* = 17.6, 10.9 Hz, 1H), 6.61 (d, *J* = 3.1 Hz, 1H), 5.73 (d, *J* = 17.6 Hz, 1H), 5.44 (s, 2H), 5.18 (d, *J* = 10.9 Hz, 1H). ^{13}**C NMR** (100 MHz, $CDCl_3$) δ 140.3, 139.7, 138.0, 136.6, 136.4, 133.9, 131.6, 130.6, 129.8, 129.1, 129.0, 128.4, 127.9, 127.0, 126.8, 126.2, 126.0, 125.9, 125.5, 120.2, 119.6, 111.1, 109.9, 102.3, 50.15. **HRMS (APCI)** $[C_{27}H_{21}N+H]^+$ = 360.1747, found m/z = 360.1748

14. REFERENCES

1) Fulmer, G. R.; Miller, A. J. M.; Sherden, N. H.; Gottlieb, H. E.; Nudelman, A.; Stoltz, B. M.; Bercaw, J. E.; Goldberg, K. I. NMR Chemical Shifts of Trace Impurities: Common Laboratory Solvents, Organics, and Gases in Deuterated Solvents Relevant to the Organometallic Chemist. *Organometallics* **2010**, *29*, 2176–2179 https://doi.org/10.1021/om100106e.
2) Yin, X.; Chen, B.; Qiu, F.; Wang, X.; Liao, Y.; Wang, M.; Lei, X.; Liao, J. Enantioselective Palladium-Catalyzed Hydrofluorination of Alkenylarenes. *ACS Catal.* **2020**, *10*, 1954–1960. https://doi.org/10.1021/acscatal.9b05264.
3) Liu, Y.; Wang, J. Synthesis of 4-Substituted Styrene Compounds via Palladium-Catalyzed Suzuki Coupling Reaction Using Free Phosphine Ligand in Air. *Synth. Commun.* **2009**, *40*, 196–205. https://doi.org/10.1080/00397910902883611.

15. NMR SPECTRA

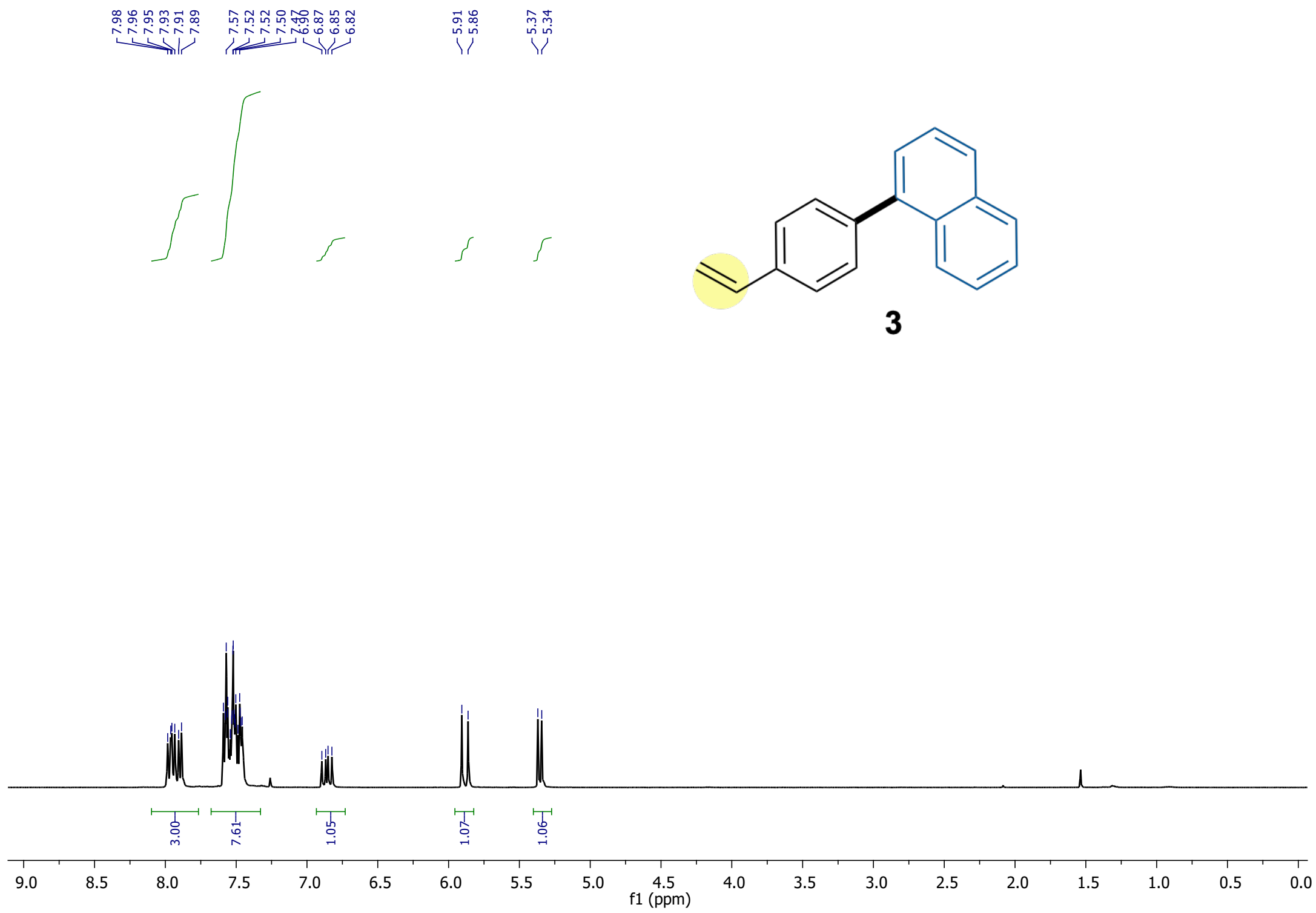

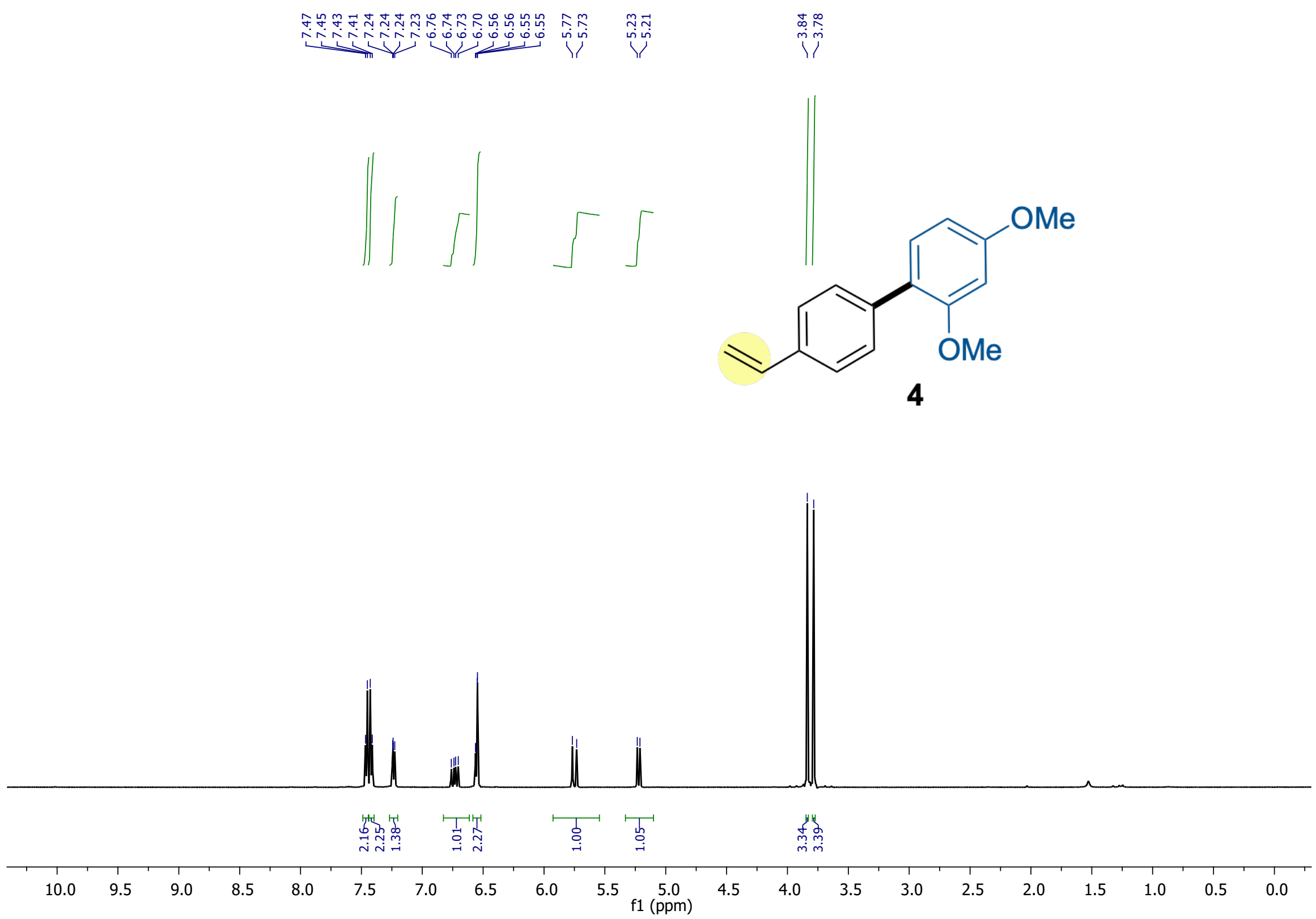

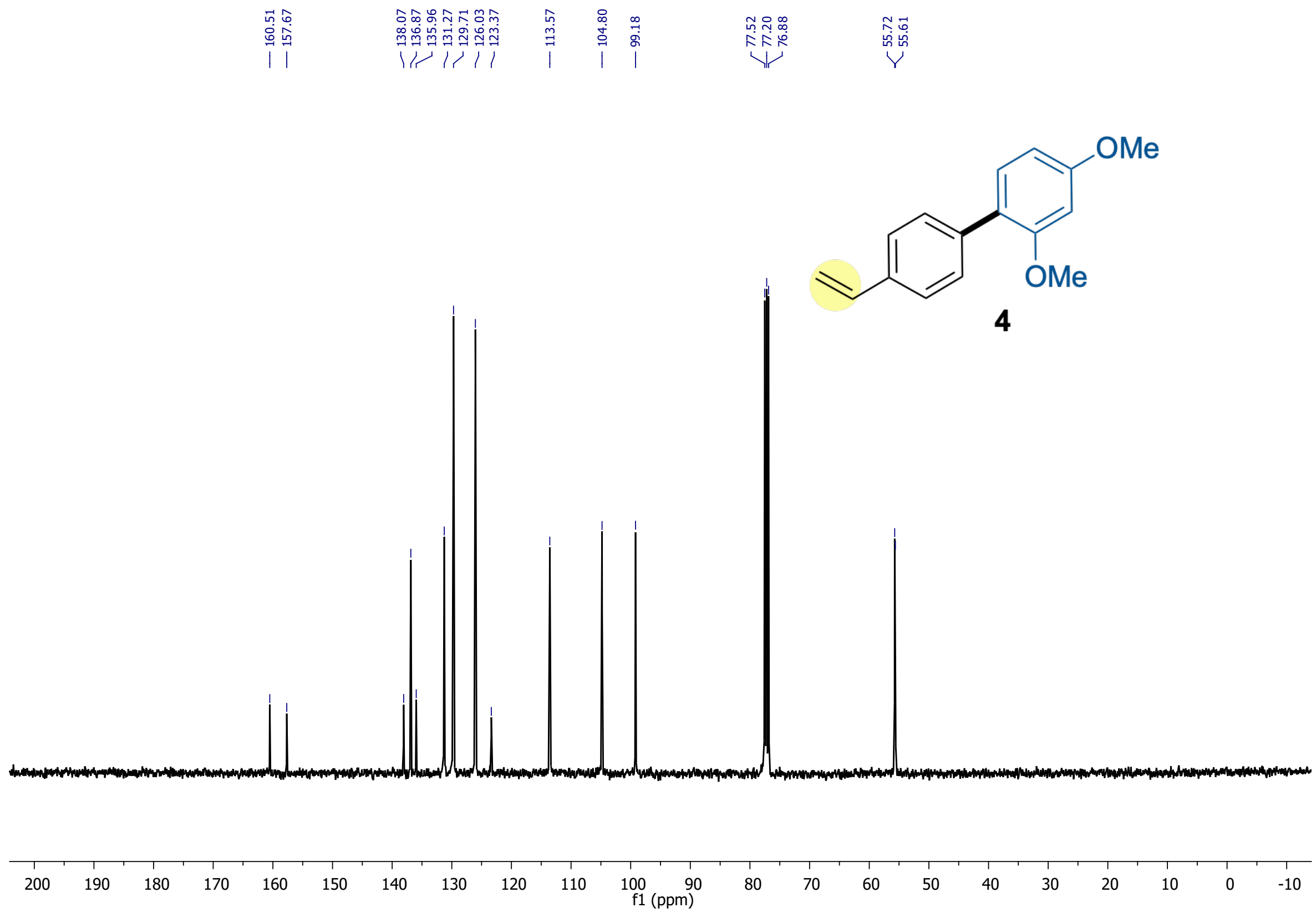
160.51
157.67
138.07
136.87
135.96
131.27
129.71
126.03
123.37
113.57
104.80
99.18
77.52
77.20
76.88
55.72
55.61
OMe
OMe
4
f1 (ppm)

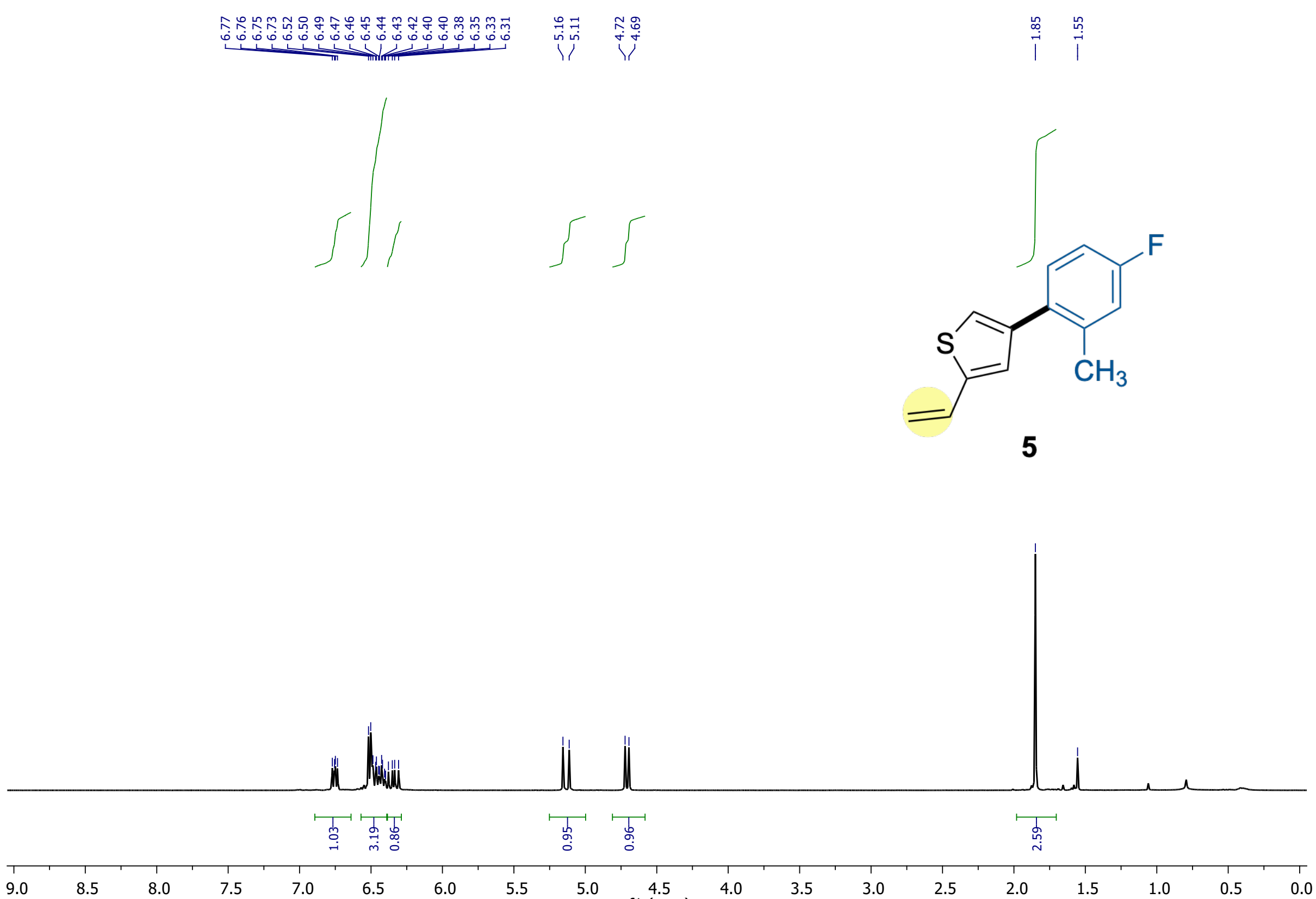
6.77
6.76
6.75
6.73
6.52
6.50
6.49
6.47
6.46
6.45
6.44
6.43
6.42
6.40
6.40
6.38
6.35
6.33
6.31
5.16
5.11
4.72
4.69
1.85
1.55
S
F
CH3
5
1.03
3.19
0.86
0.95
0.96
2.59
f1 (ppm)

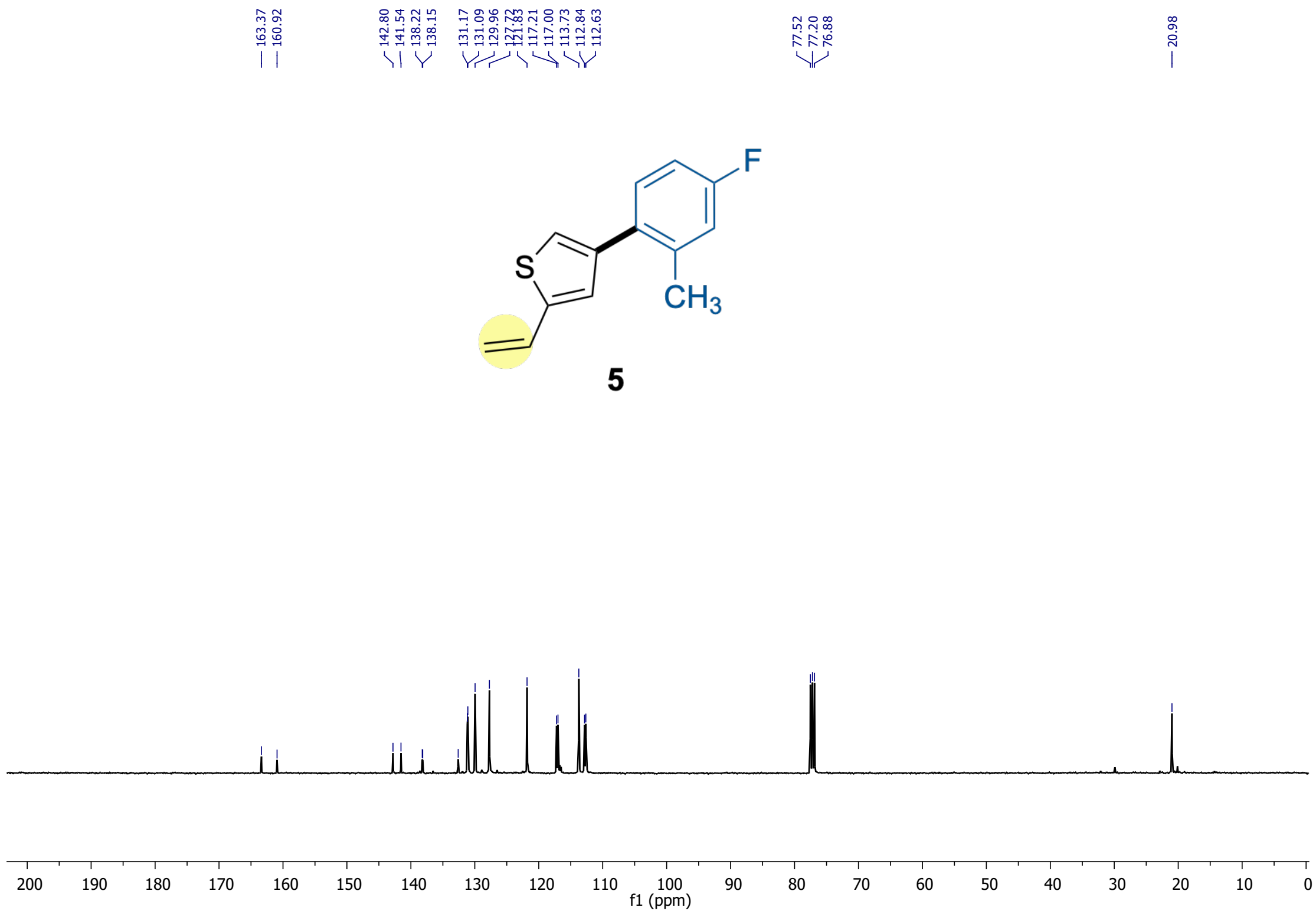
163.37
160.92
142.80
141.54
138.22
138.15
131.17
131.09
129.96
121.83
117.21
117.00
113.73
112.84
112.63
77.52
77.20
76.88
20.98
S
F
CH3
5
f1 (ppm)

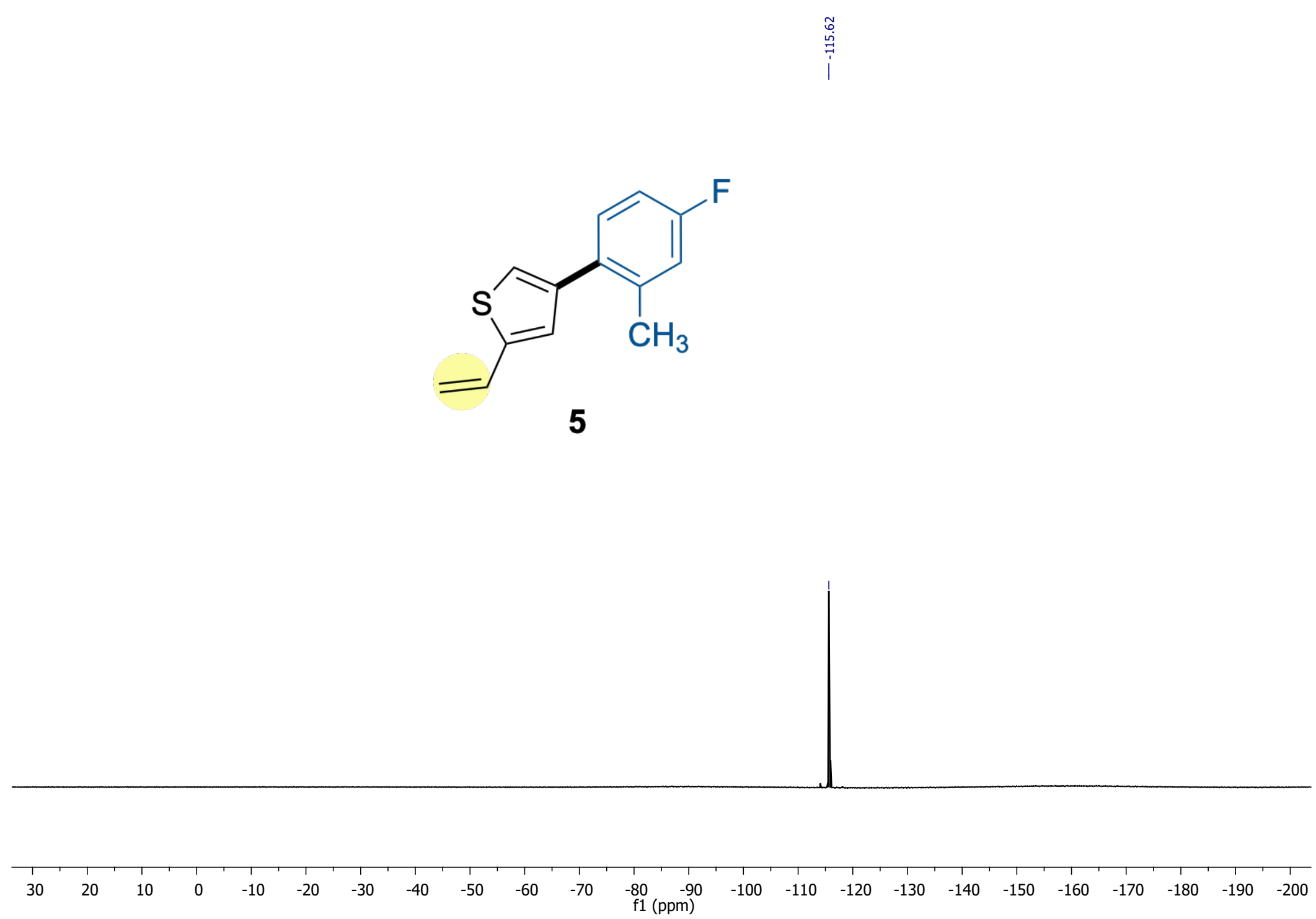
-115.62
S
F
CH3
5
f1 (ppm)

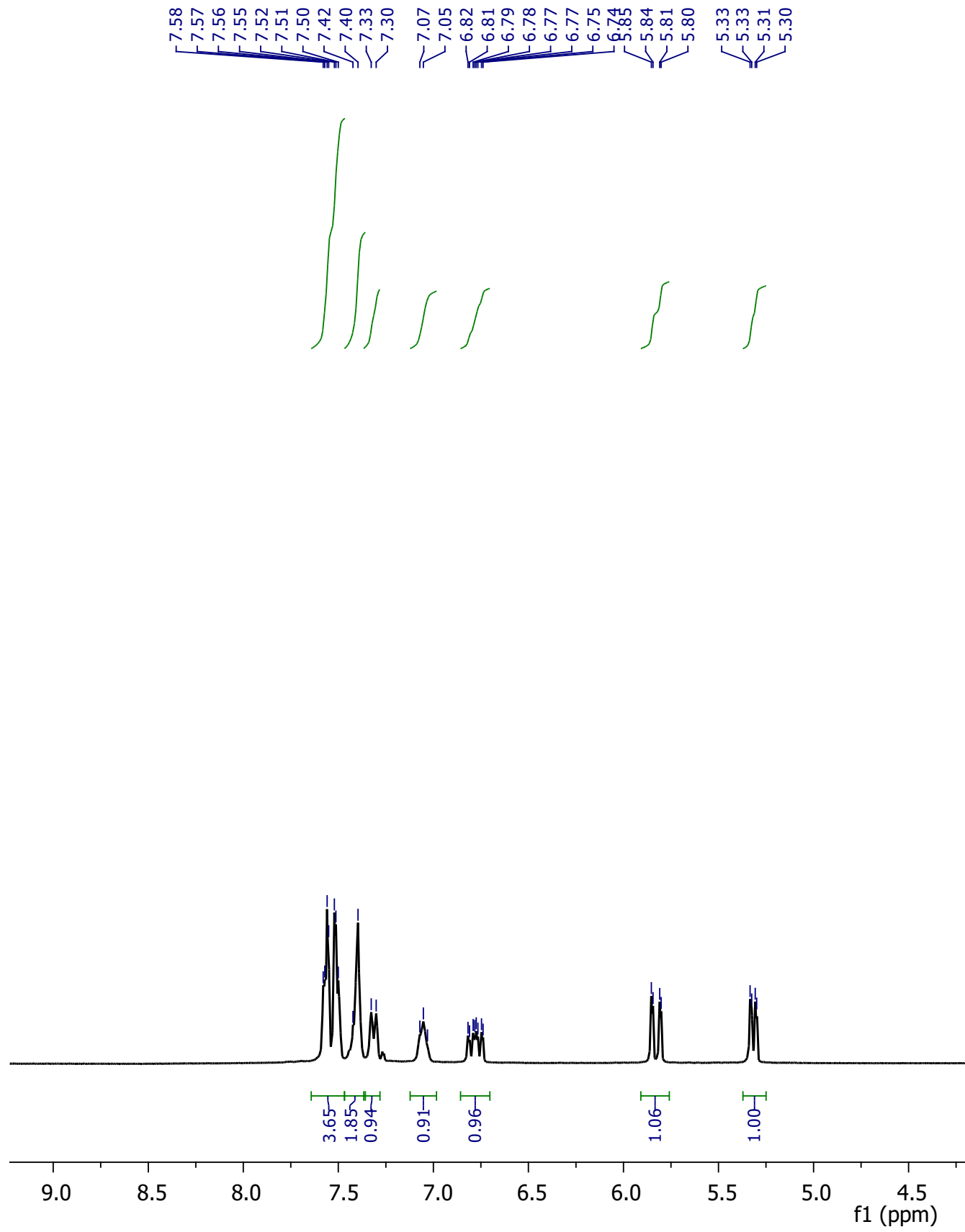
7.58 7.57 7.56 7.55 7.52 7.51 7.50 7.42 7.40 7.33 7.30 7.07 7.05 6.82 6.81 6.79 6.78 6.77 6.77 6.75 6.74 5.85 5.84 5.81 5.80 5.33 5.33 5.31 5.30
3.65 1.85 0.94 0.91 0.96 1.06 1.00
9.0 8.5 8.0 7.5 7.0 6.5 6.0 5.5 5.0 4.5 4.0 3.5 3.0 2.5 2.0 1.5 1.0 0.5 0.0
f1 (ppm)

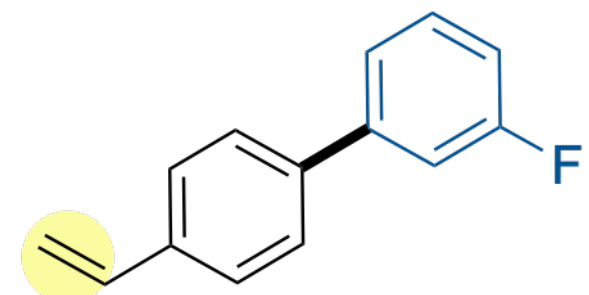
F

6

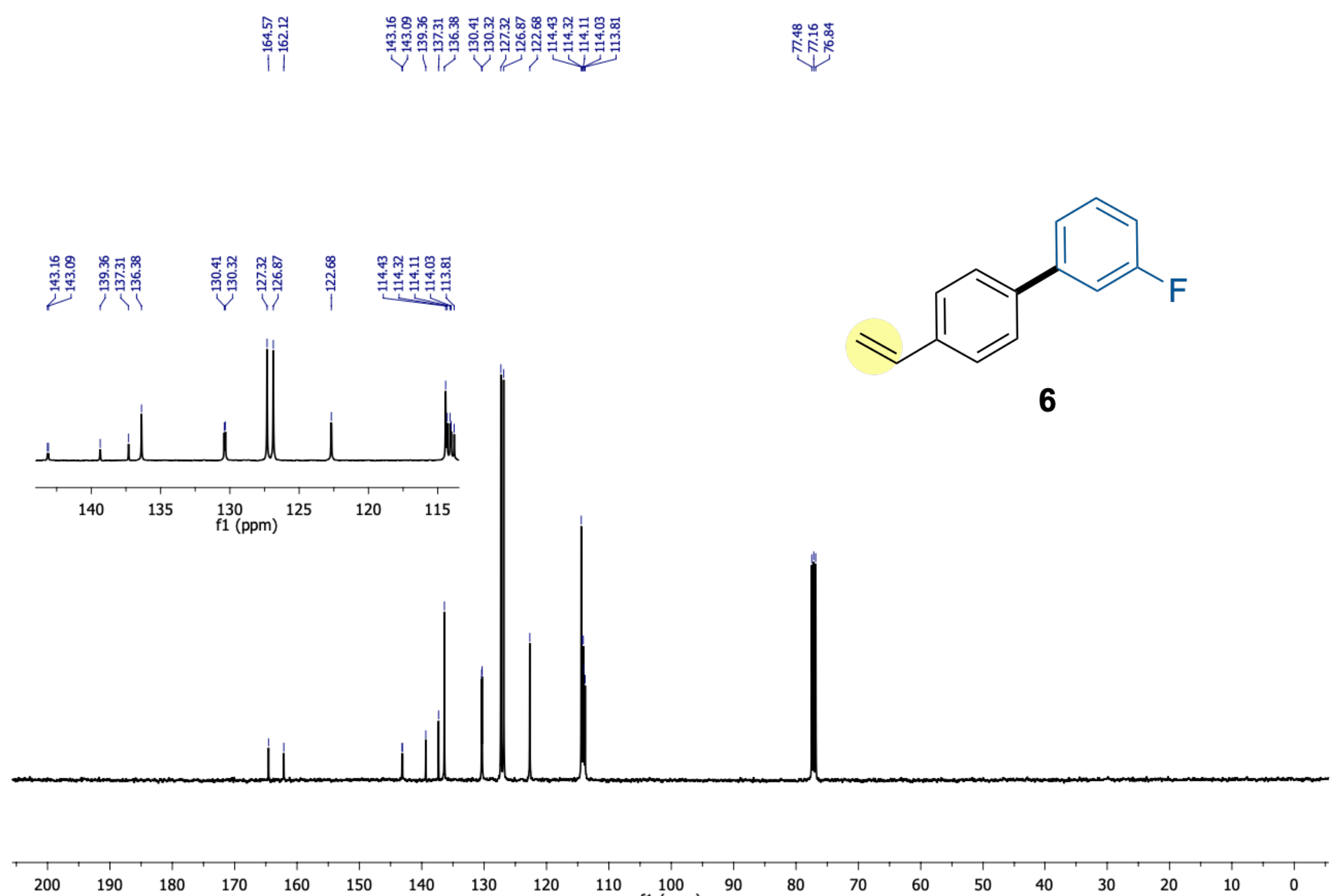
164.57 162.12
143.16 143.09 139.36 137.31 136.38 130.41 130.32 127.32 126.87 122.68 114.43 114.32 114.11 114.03 113.81
77.48 77.16 76.84
140 135 130 125 120 115
f1 (ppm)
200 190 180 170 160 150 140 130 120 110 100 90 80 70 60 50 40 30 20 10 0
f1 (ppm)
F

6

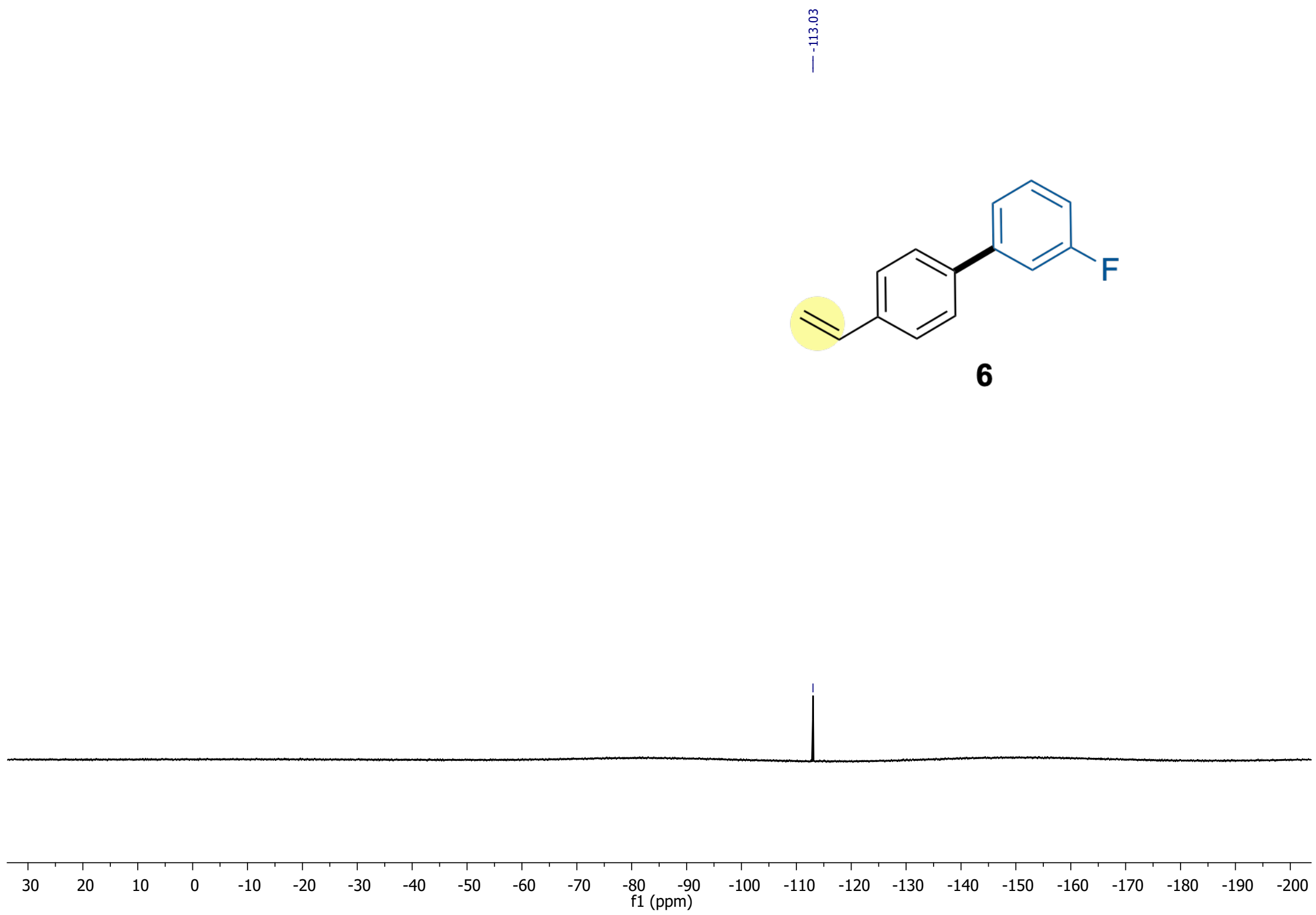
-113.03
F
f1 (ppm)

6

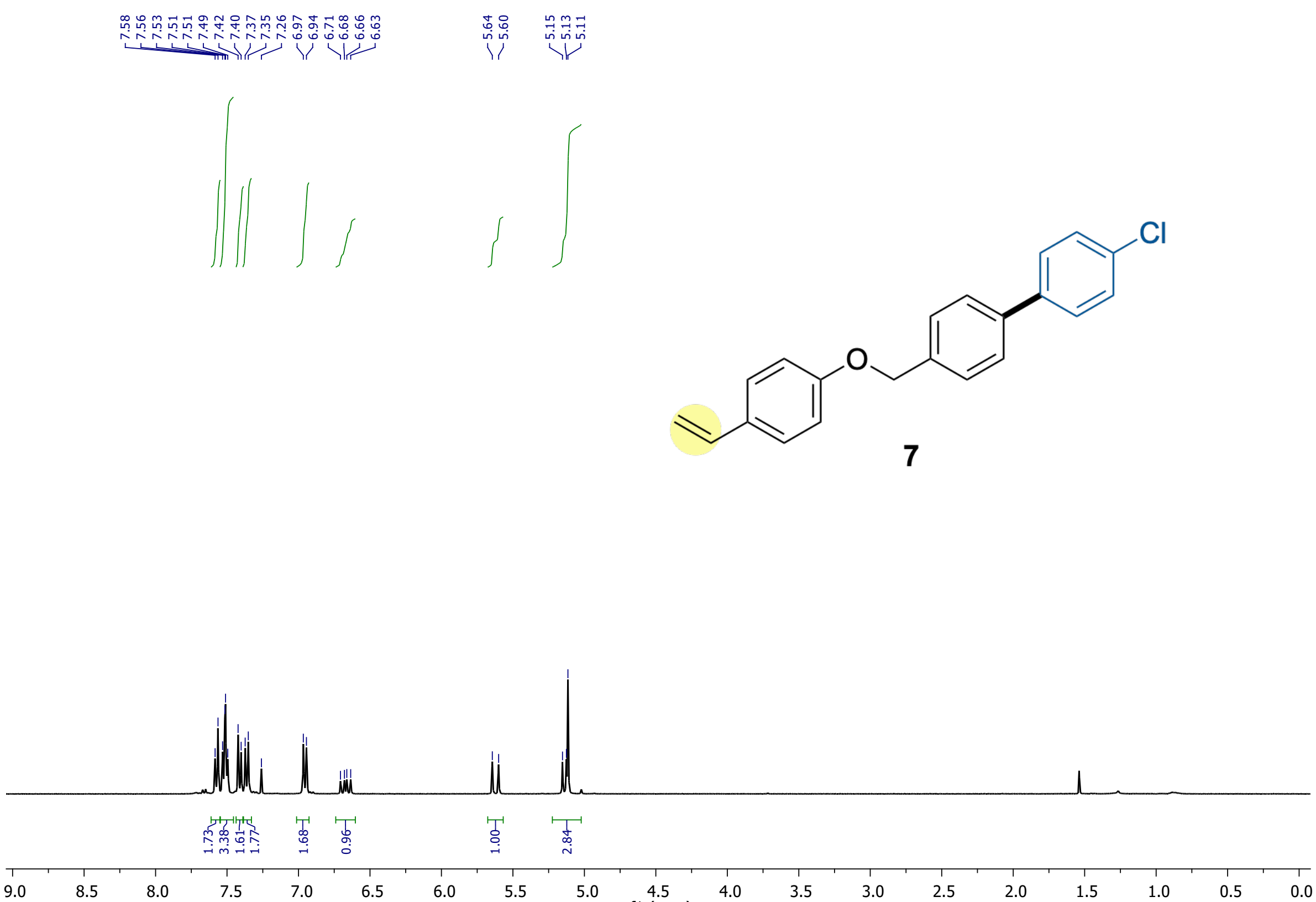
7.58
7.56
7.53
7.51
7.51
7.49
7.42
7.40
7.37
7.35
7.26
6.97
6.94
6.71
6.68
6.66
6.63
5.64
5.60
5.15
5.13
5.11
Cl
O
1.73
3.38
1.61
1.77
1.68
0.96
1.00
2.84
f1 (ppm)

7

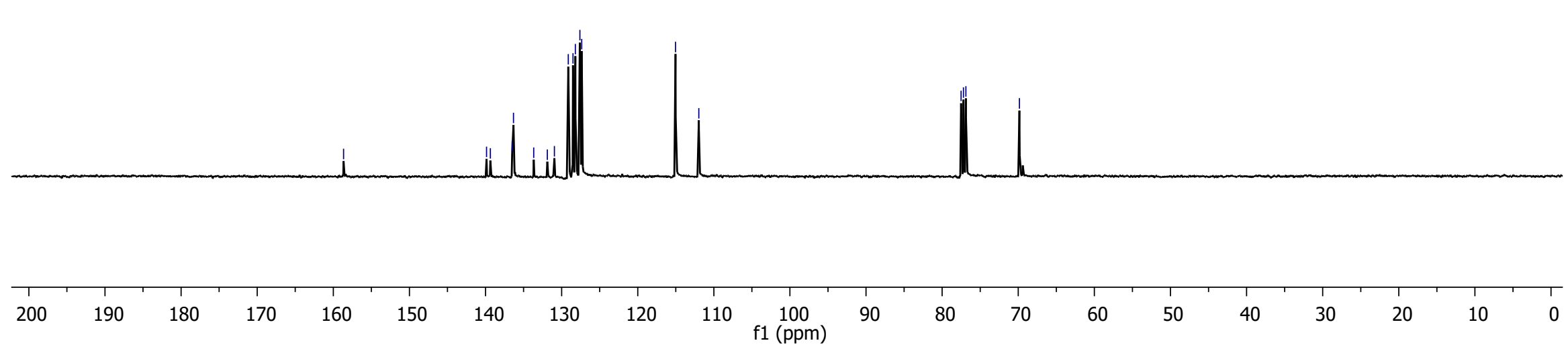
158.65
139.86
139.36
136.50
136.33
133.67
131.88
130.95
129.12
128.51
128.19
127.60
127.35
115.04
111.97
77.52
77.20
76.88
69.85
f1 (ppm)

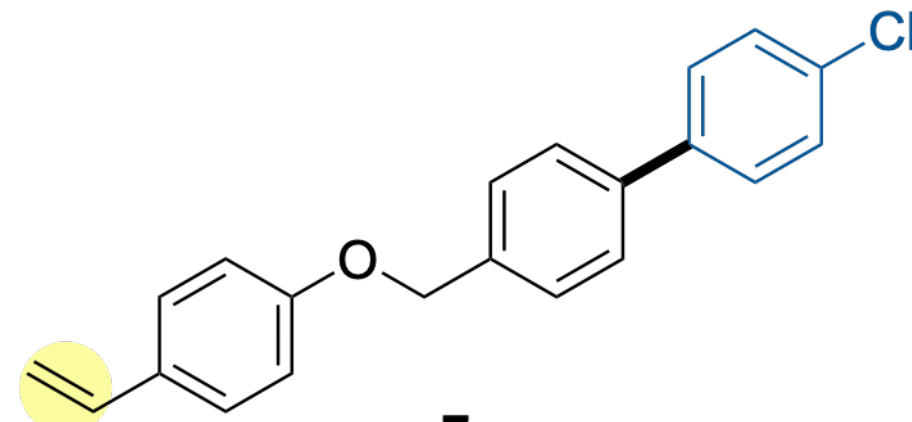
Cl
O

7

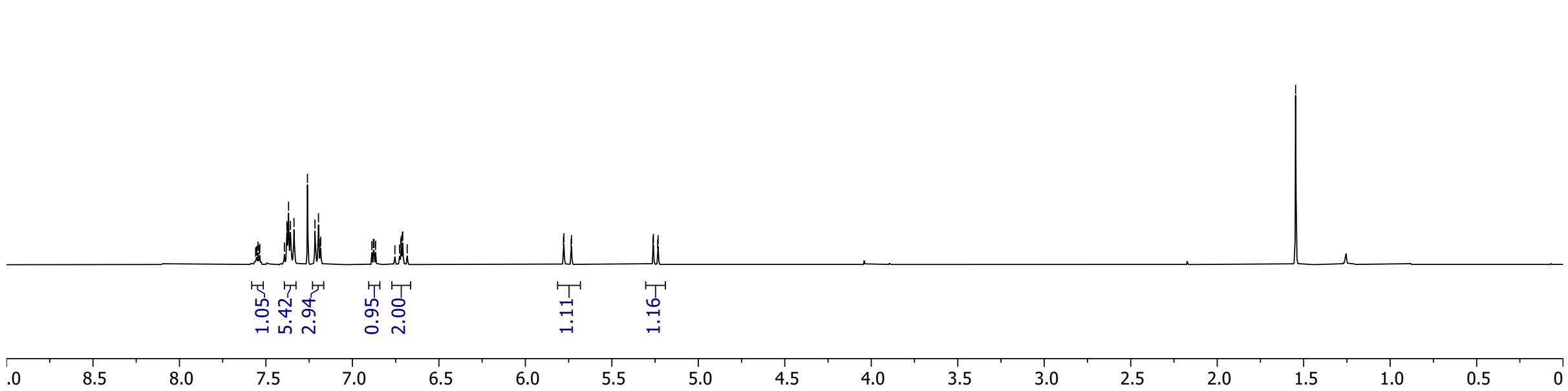
7.55
7.54
7.54
7.39
7.38
7.38
7.37
7.36
7.34
7.26
7.22
7.20
7.19
7.18
6.88
6.88
6.87
6.75
6.73
6.72
6.72
6.71
6.68
5.78
5.78
5.74
5.73
5.26
5.26
5.23
5.23
1.55
1.05
5.42
2.94
0.95
2.00
1.11
1.16
f1 (ppm)

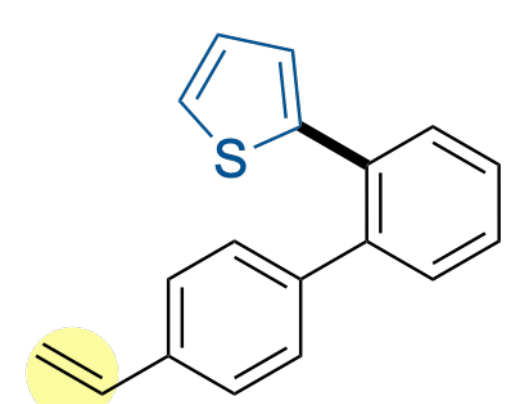
S

8

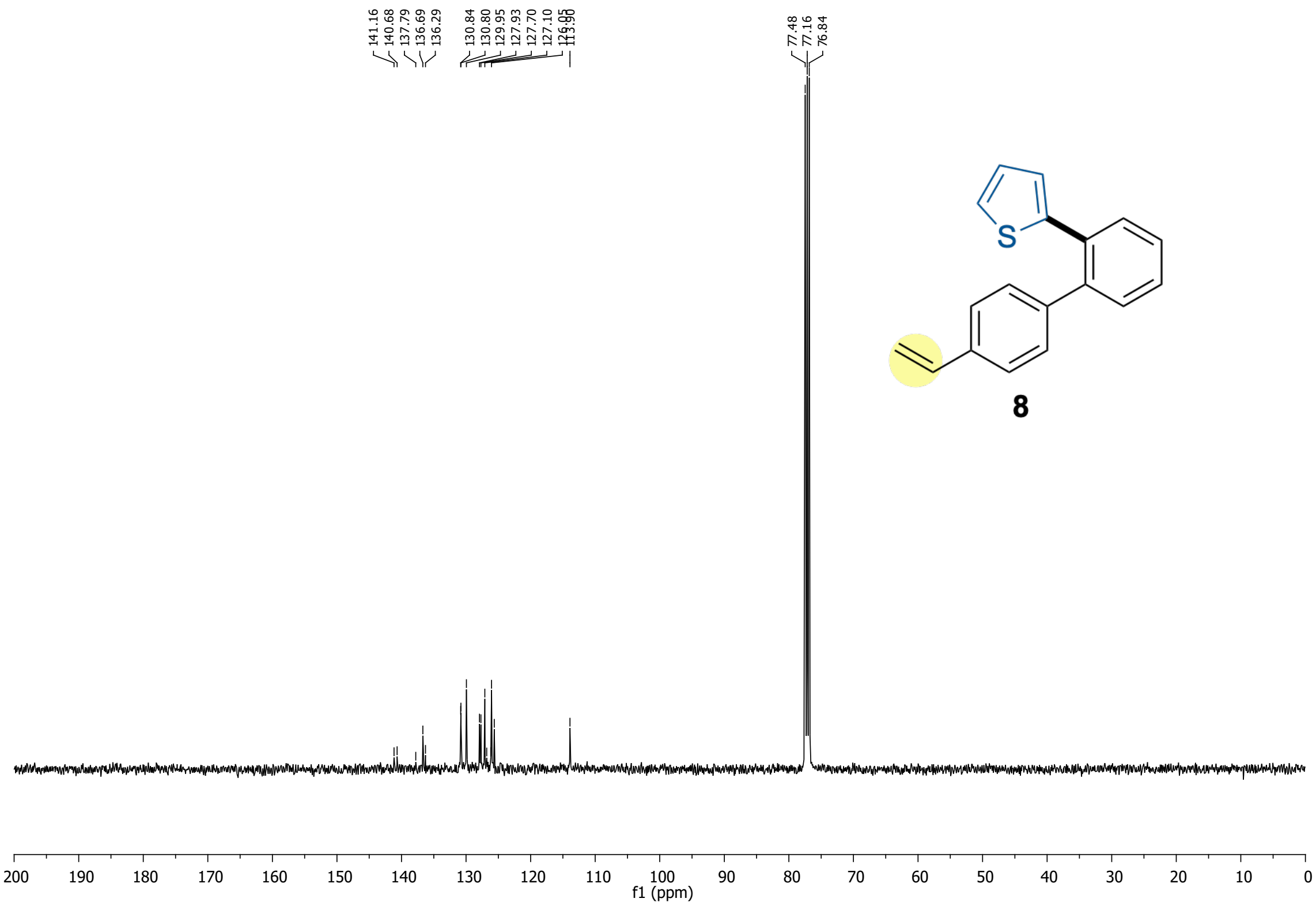
141.16
140.68
137.79
136.69
136.29
130.84
130.80
129.95
127.93
127.70
127.10
126.05
113.90
77.48
77.16
76.84
200 190 180 170 160 150 140 130 120 110 100 90 80 70 60 50 40 30 20 10 0
f1 (ppm)
S

8

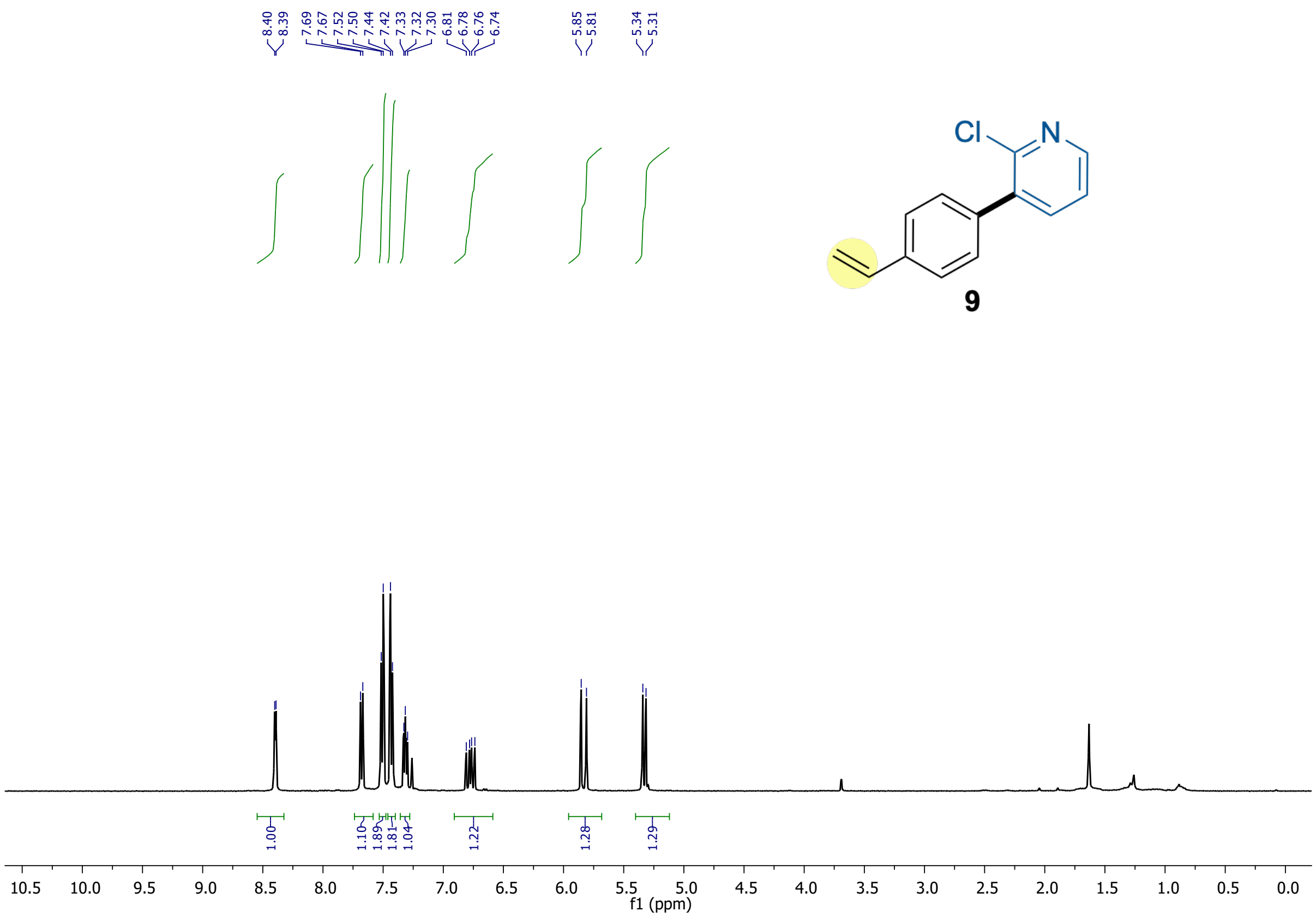
8.40
8.39
7.69
7.67
7.52
7.50
7.44
7.42
7.33
7.32
7.30
6.81
6.78
6.76
6.74
5.85
5.81
5.34
5.31
1.00
1.10
1.89
1.81
1.04
1.22
1.28
1.29
10.5 10.0 9.5 9.0 8.5 8.0 7.5 7.0 6.5 6.0 5.5 5.0 4.5 4.0 3.5 3.0 2.5 2.0 1.5 1.0 0.5 0.0
f1 (ppm)
Cl
N

9

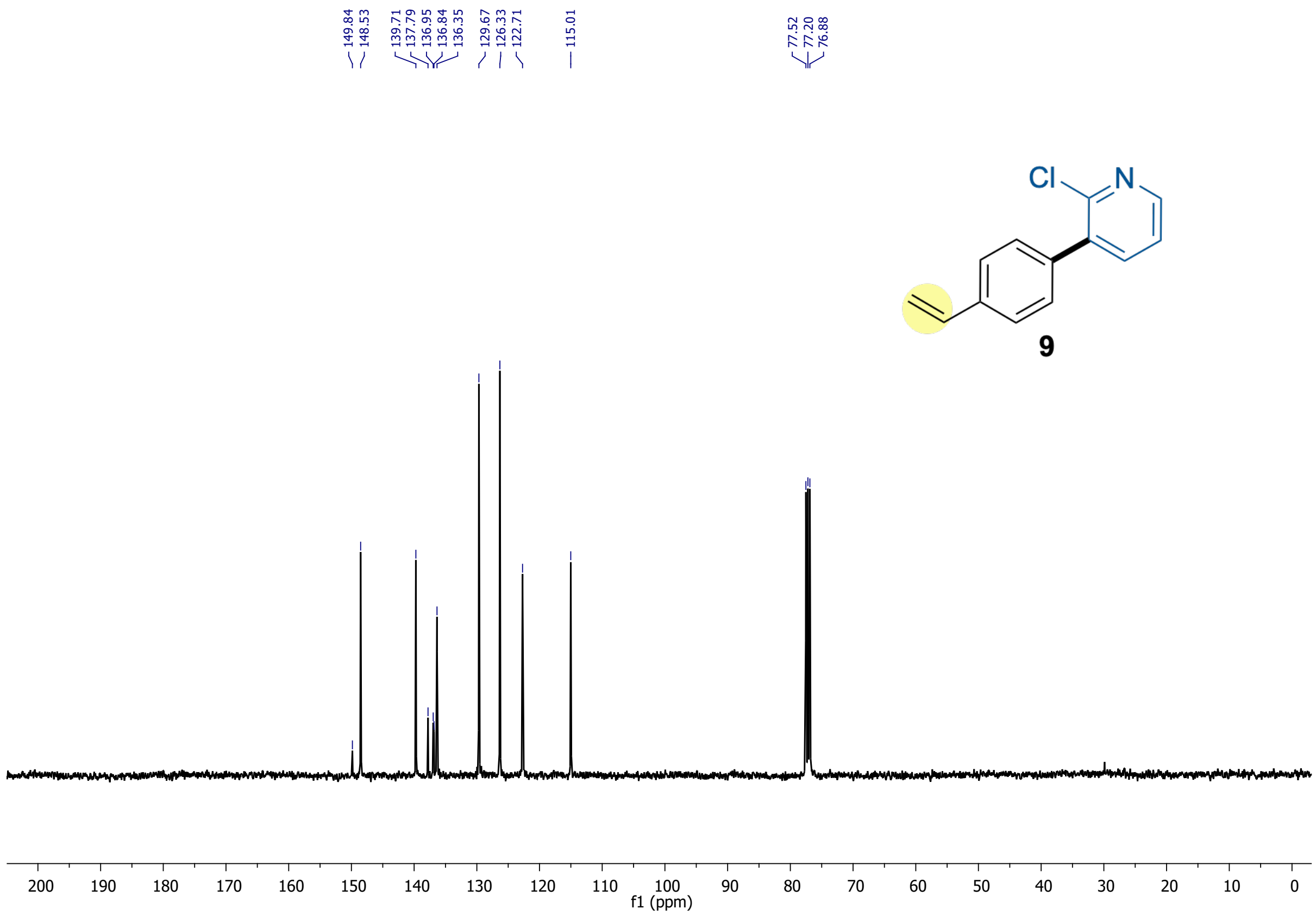
149.84
148.53
139.71
137.79
136.95
136.84
136.35
129.67
126.33
122.71
115.01
77.52
77.20
76.88
Cl
N
9
200 190 180 170 160 150 140 130 120 110 100 90 80 70 60 50 40 30 20 10 0
f1 (ppm)

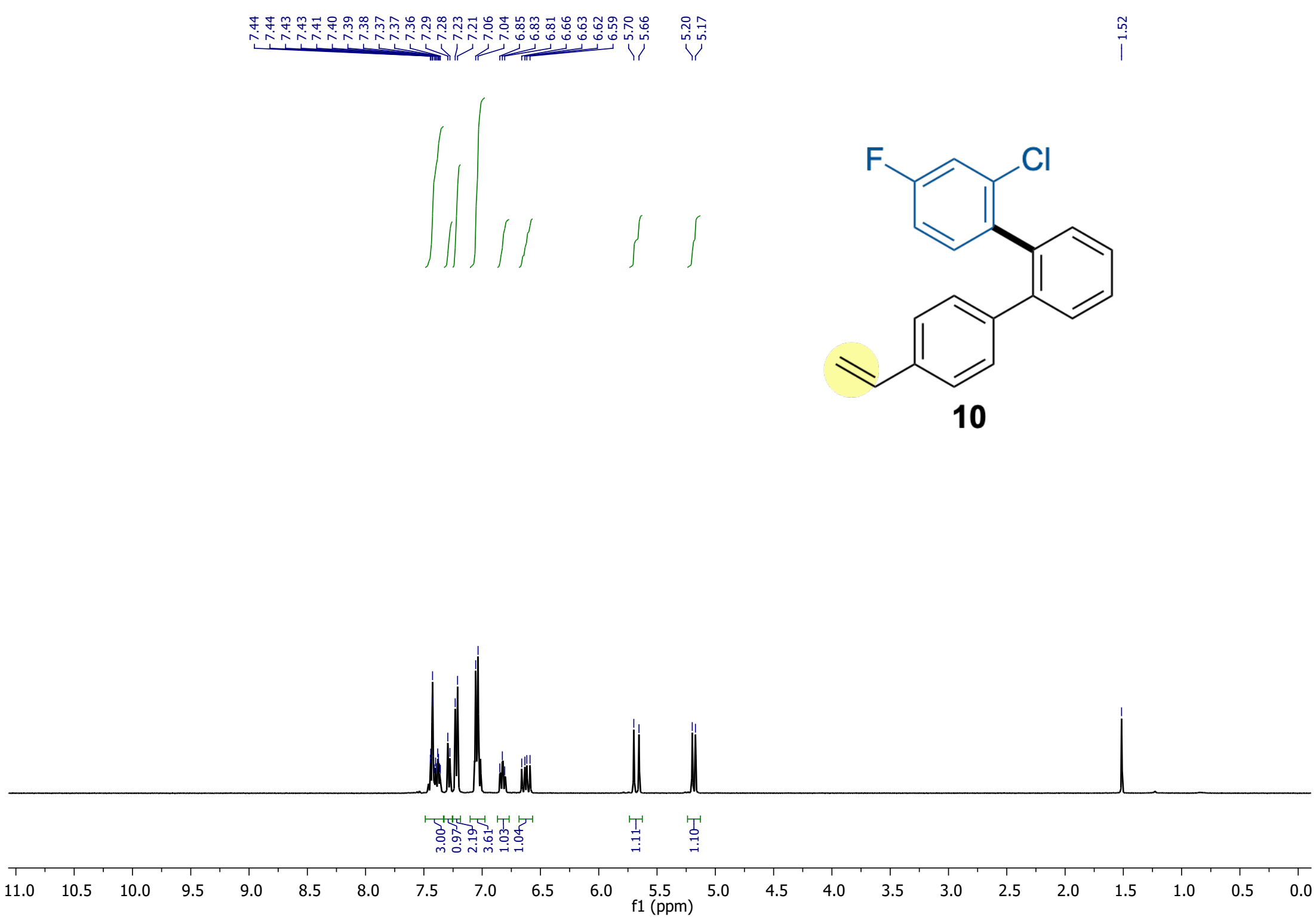
7.44
7.44
7.43
7.43
7.41
7.40
7.39
7.38
7.37
7.37
7.36
7.29
7.28
7.23
7.21
7.06
7.04
6.85
6.83
6.81
6.66
6.63
6.62
6.59
5.70
5.66
5.20
5.17
1.52
F
Cl
10
3.00
0.97
2.19
3.61
1.03
1.04
1.11
1.10
11.0 10.5 10.0 9.5 9.0 8.5 8.0 7.5 7.0 6.5 6.0 5.5 5.0 4.5 4.0 3.5 3.0 2.5 2.0 1.5 1.0 0.5 0.0
f1 (ppm)

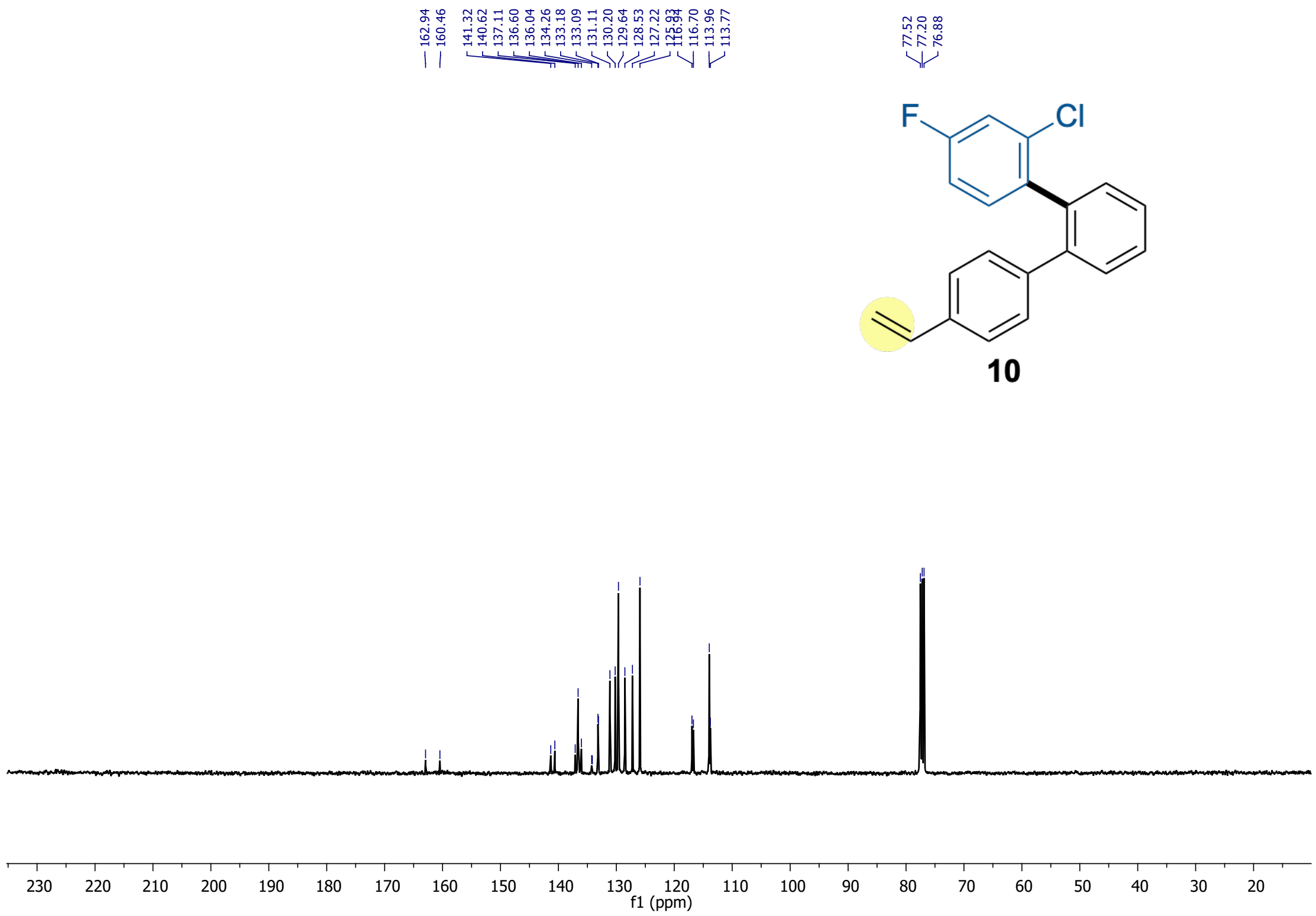
162.94
160.46
141.32
140.62
137.11
136.60
136.04
134.26
133.18
133.09
131.11
130.20
129.64
128.53
127.22
125.93
116.94
116.70
113.96
113.77
77.52
77.20
76.88
230 220 210 200 190 180 170 160 150 140 130 120 110 100 90 80 70 60 50 40 30 20
f1 (ppm)
F
Cl

10

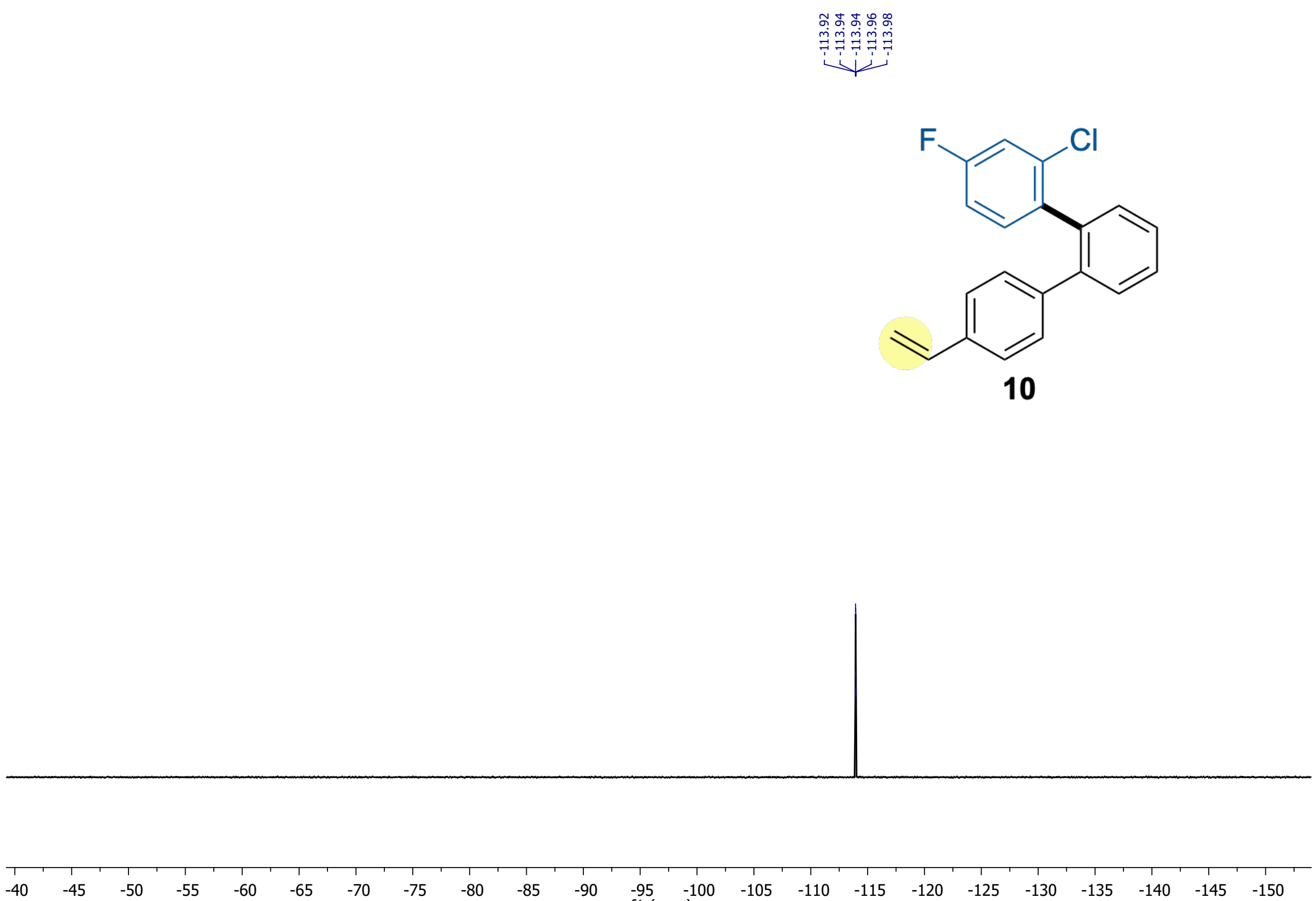
-113.92
-113.94
-113.94
-113.96
-113.98
-40 -45 -50 -55 -60 -65 -70 -75 -80 -85 -90 -95 -100 -105 -110 -115 -120 -125 -130 -135 -140 -145 -150
f1 (ppm)
F
Cl

10

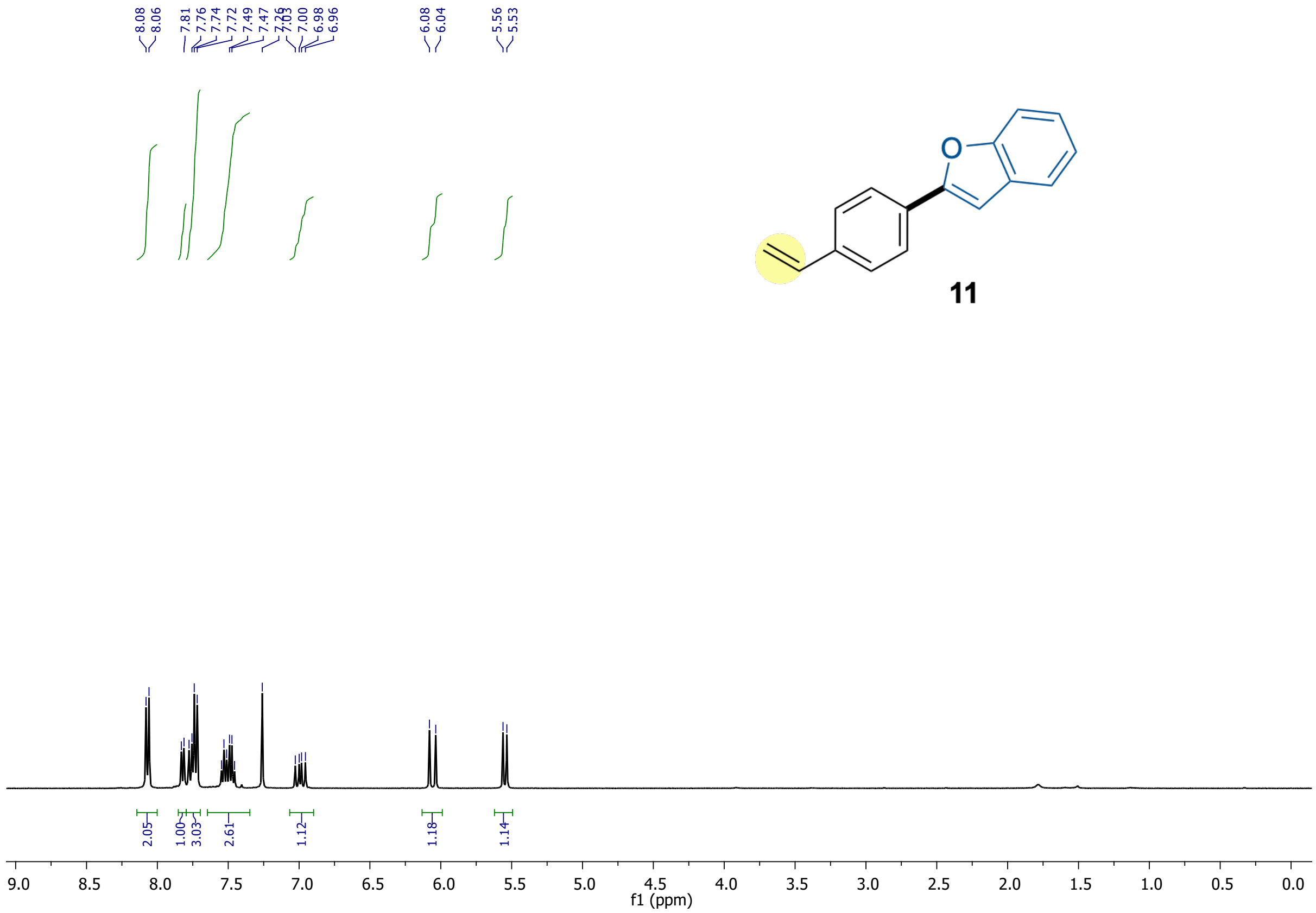

8.08
8.06
7.81
7.76
7.74
7.72
7.49
7.47
7.26
7.03
7.00
6.98
6.96
6.08
6.04
5.56
5.53
2.05
1.00
3.03
2.61
1.12
1.18
1.14
9.0
8.5
8.0
7.5
7.0
6.5
6.0
5.5
5.0
4.5
4.0
3.5
3.0
2.5
2.0
1.5
1.0
0.5
0.0
f1 (ppm)

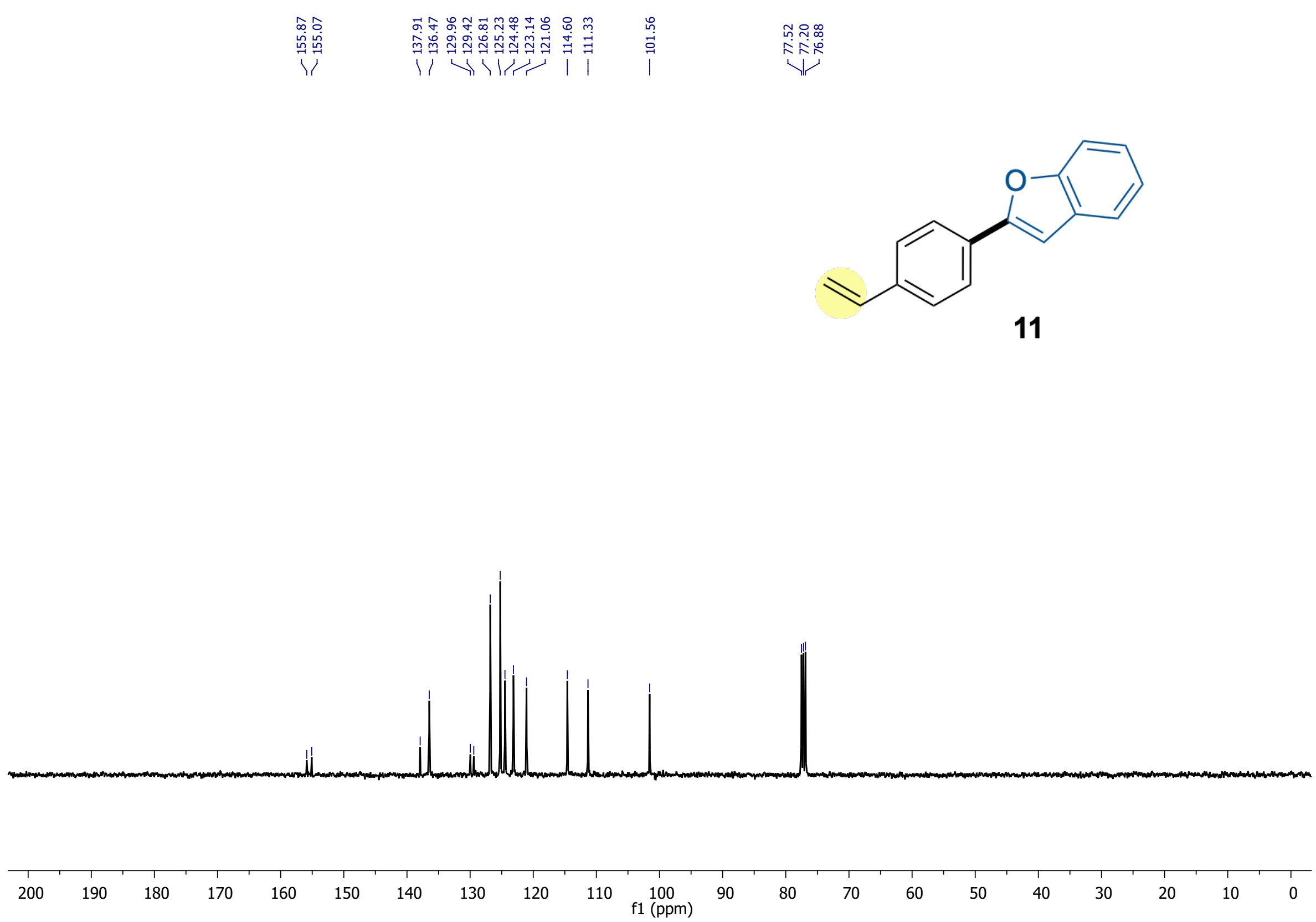

155.87
155.07
137.91
136.47
129.96
129.42
126.81
125.23
124.48
123.14
121.06
114.60
111.33
101.56
77.52
77.20
76.88
200
190
180
170
160
150
140
130
120
110
100
90
80
70
60
50
40
30
20
10
0
f1 (ppm)

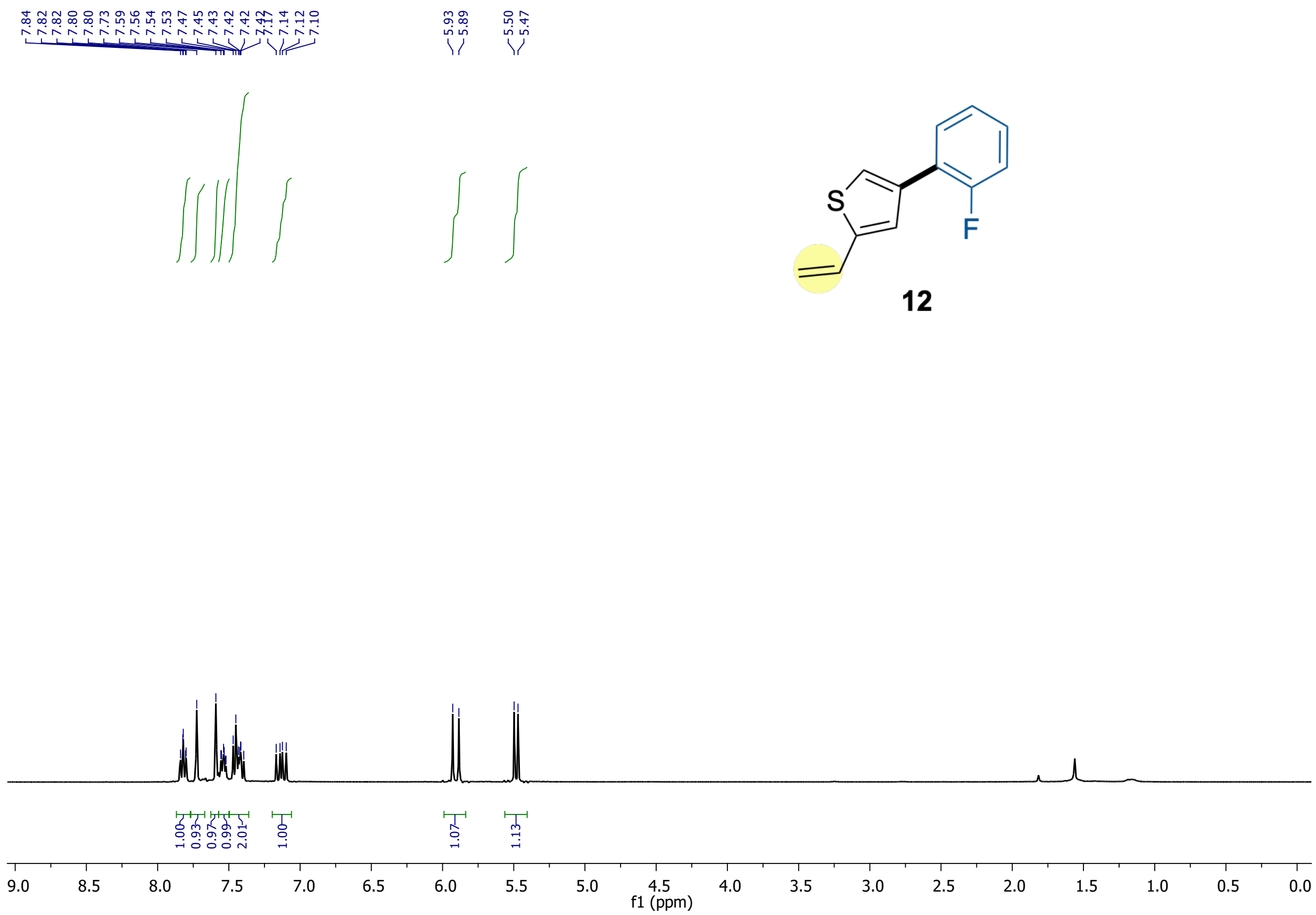
7.84 7.82 7.82 7.80 7.80 7.73 7.59 7.56 7.54 7.53 7.47 7.45 7.43 7.42 7.42 7.42 7.17 7.14 7.12 7.10
5.93 5.89
5.50 5.47
S
F
1.00 0.93 0.97 0.99 2.01 1.00 1.07 1.13
f1 (ppm)

12

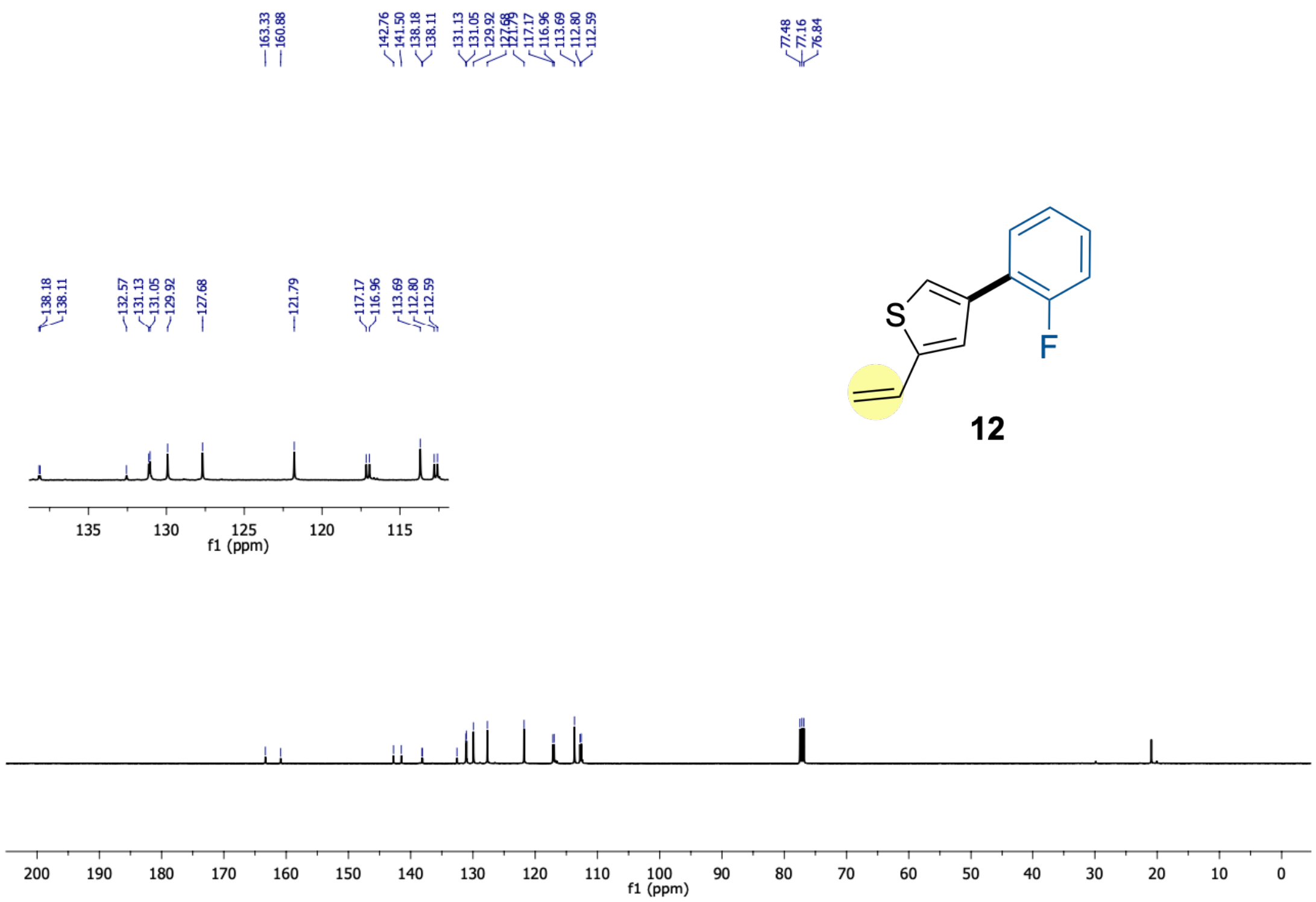
163.33 160.88
142.76 141.50 138.18 138.11 131.13 131.05 129.92 127.68 121.79 117.17 116.96 113.69 112.80 112.59
77.48 77.16 76.84
138.18 138.11 132.57 131.13 131.05 129.92 127.68 121.79 117.17 116.96 113.69 112.80 112.59
S
F
f1 (ppm)

12

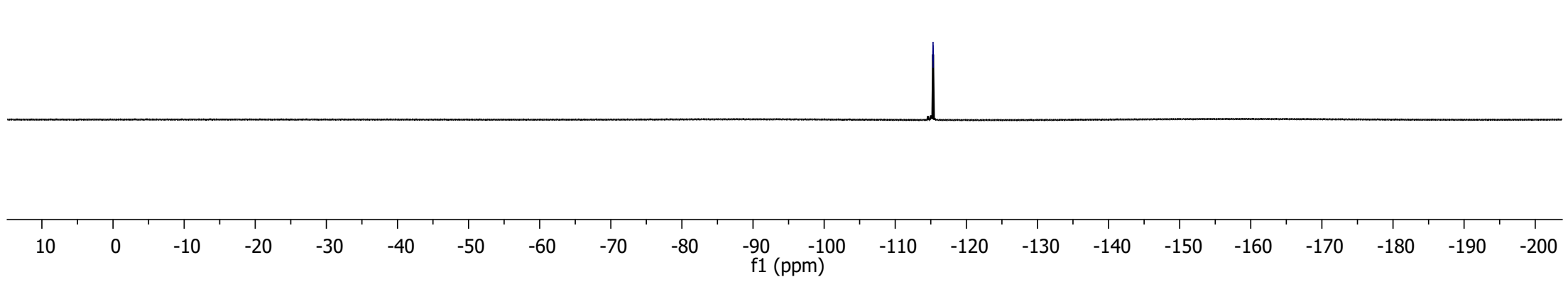
-115.30
-115.31
-115.31
-115.32
-115.32
-115.32
-115.33
10 0 -10 -20 -30 -40 -50 -60 -70 -80 -90 -100 -110 -120 -130 -140 -150 -160 -170 -180 -190 -200
f1 (ppm)

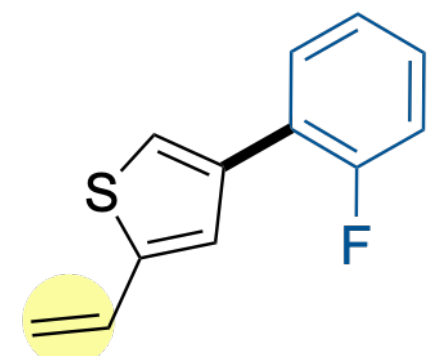
S
F

12

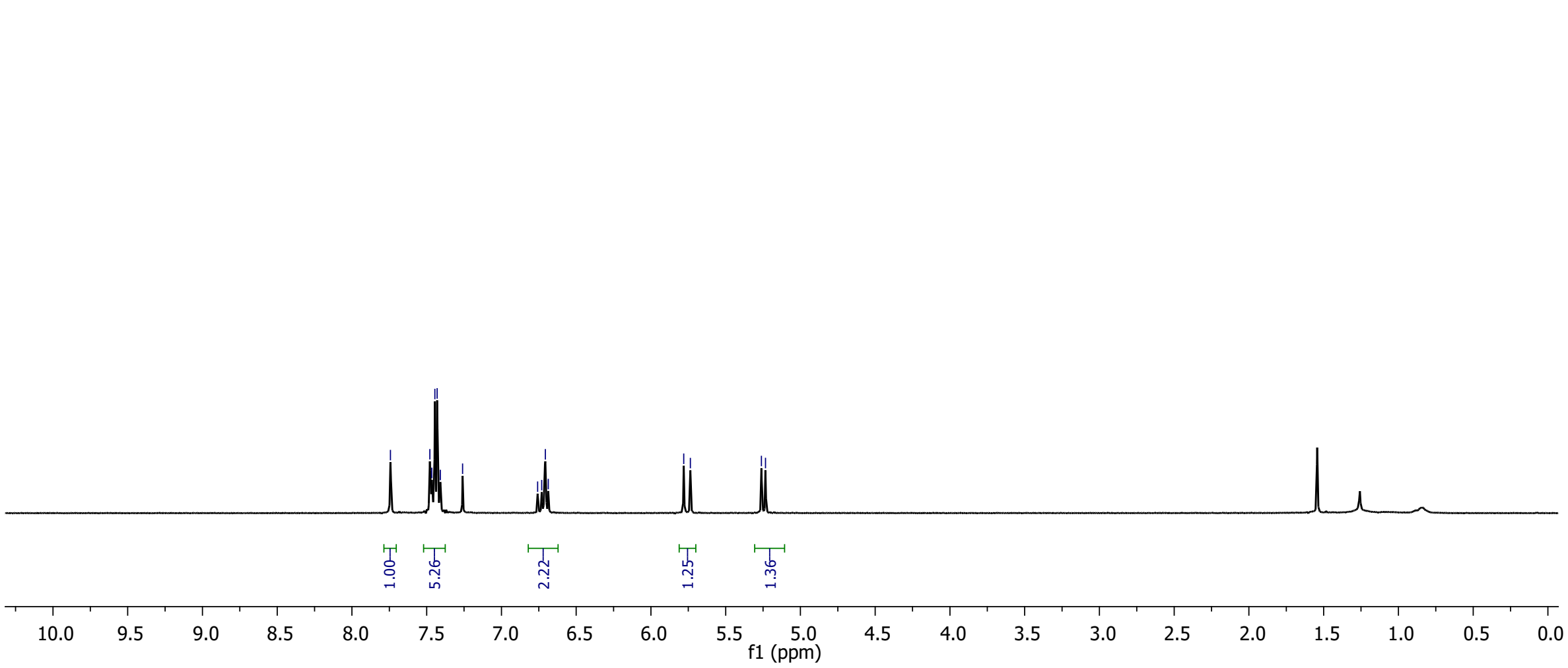
7.74
7.48
7.47
7.45
7.43
7.26
6.76
6.73
6.71
6.69
5.78
5.74
5.26
5.23
1.00
5.26
2.22
1.25
1.36
10.0 9.5 9.0 8.5 8.0 7.5 7.0 6.5 6.0 5.5 5.0 4.5 4.0 3.5 3.0 2.5 2.0 1.5 1.0 0.5 0.0
f1 (ppm)

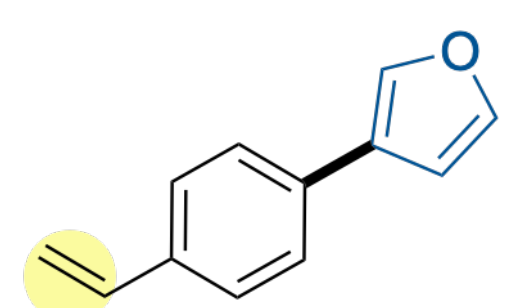
O

13

CARBON_01
ud-lnb2-230-C

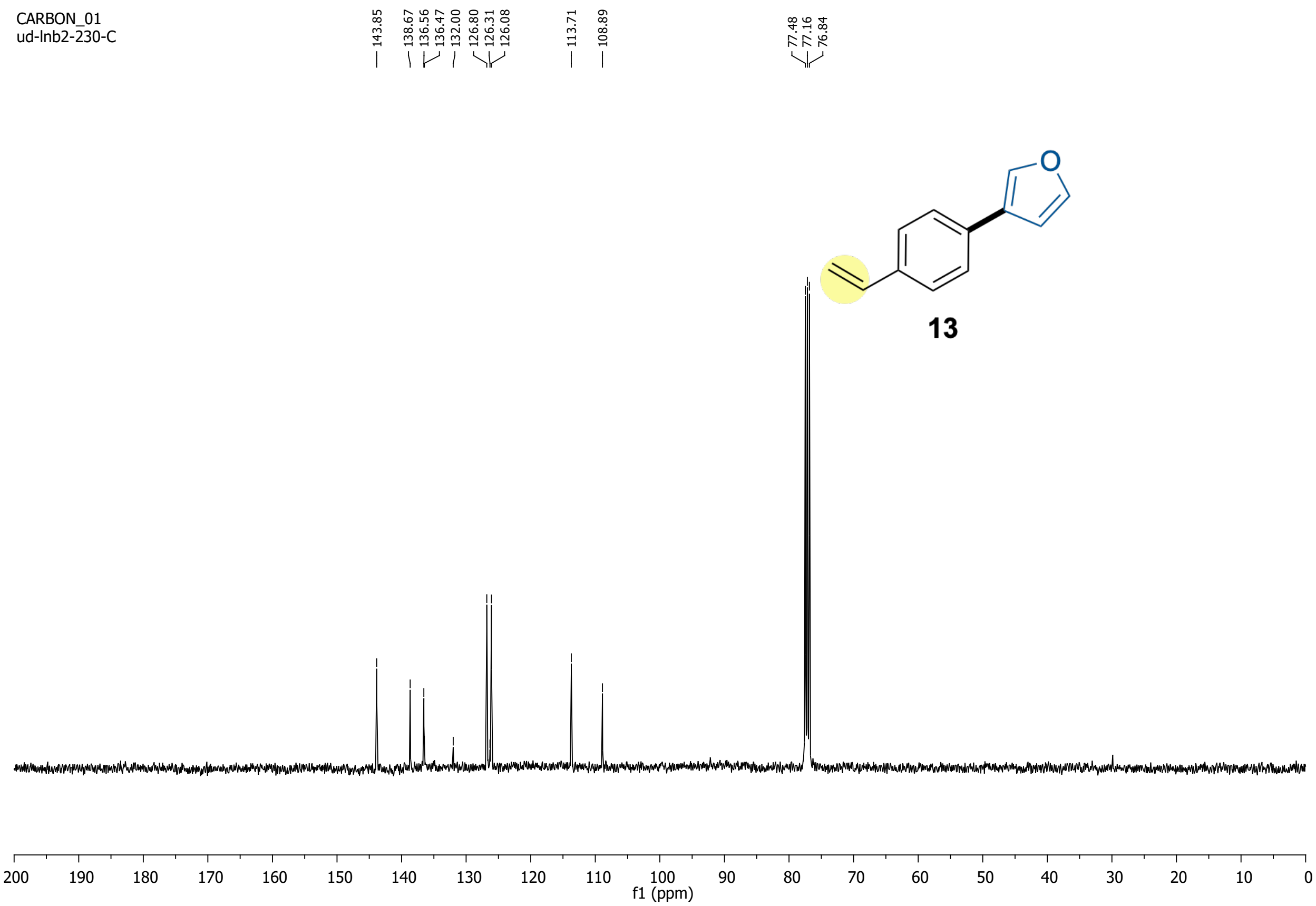

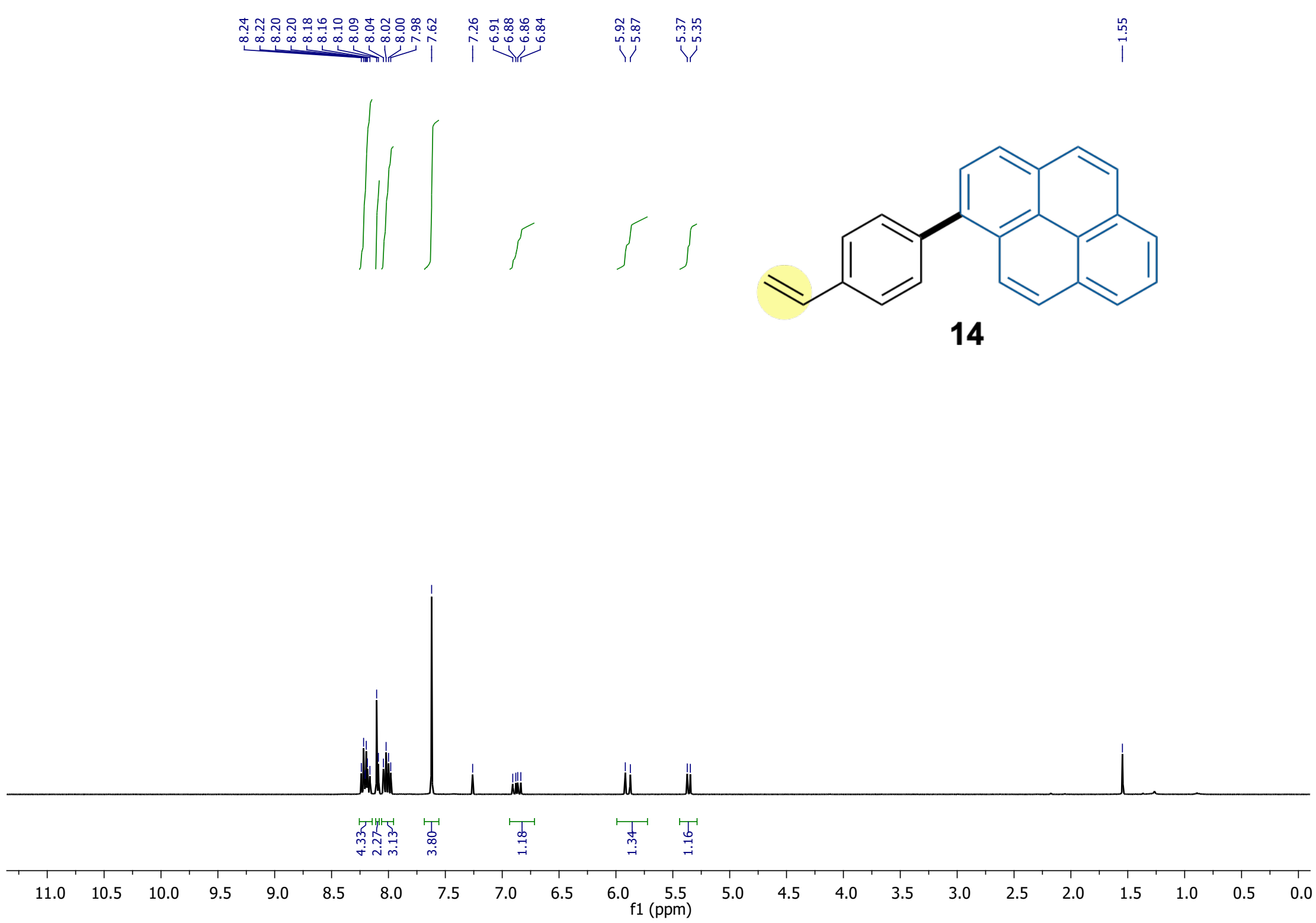

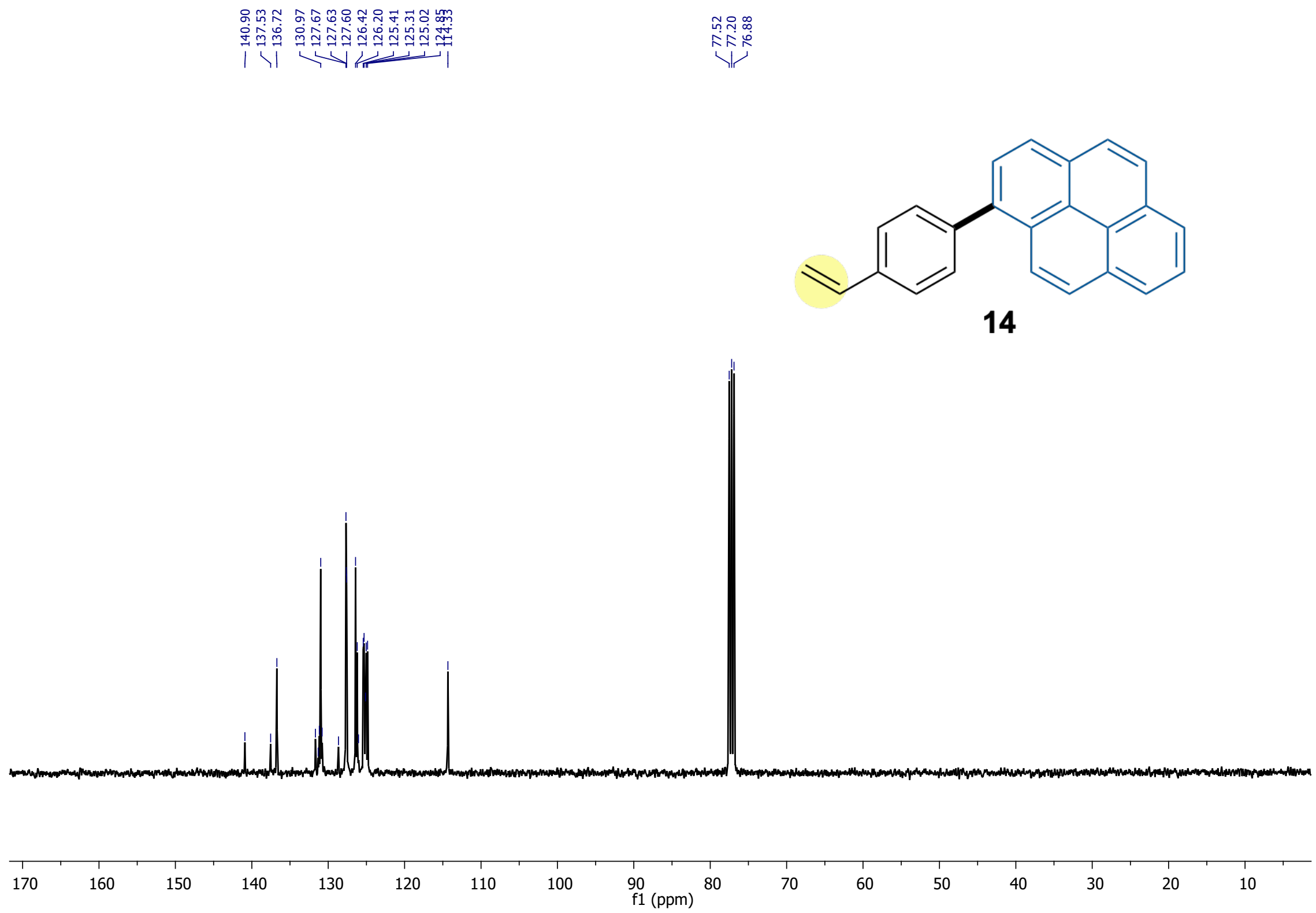
140.90
137.53
136.72
130.97
127.67
127.63
127.60
126.42
126.20
125.41
125.31
125.02
77.52
77.20
76.88
f1 (ppm)

14

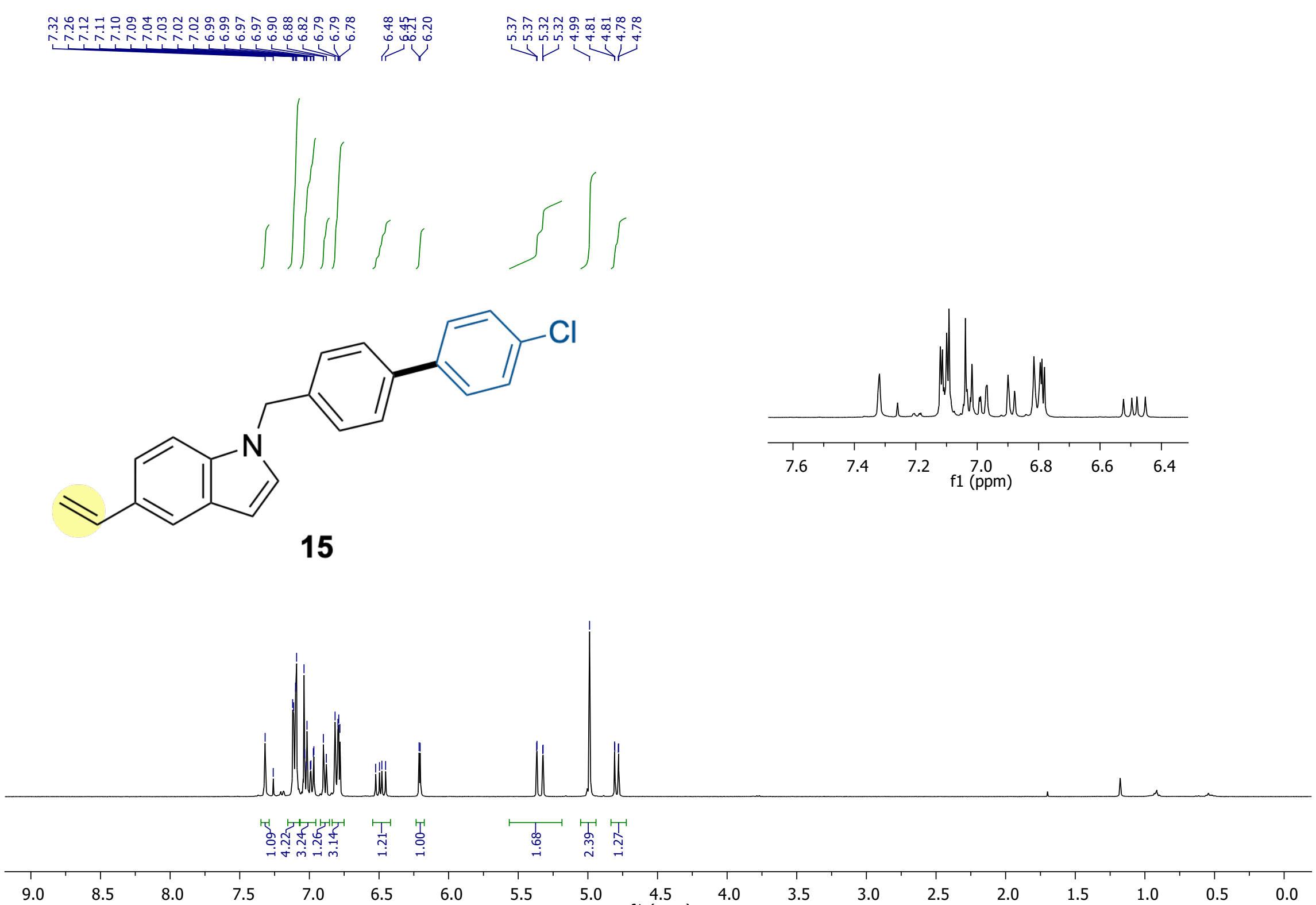
7.32
7.26
7.12
7.11
7.10
7.09
7.04
7.03
7.02
7.02
6.99
6.99
6.97
6.90
6.88
6.82
6.79
6.79
6.78
6.48
6.20
5.37
5.37
5.32
5.32
4.99
4.81
4.81
4.78
4.78
Cl
N
1.09
4.22
3.24
1.26
3.14
1.21
1.00
1.68
2.39
1.27
f1 (ppm)

15

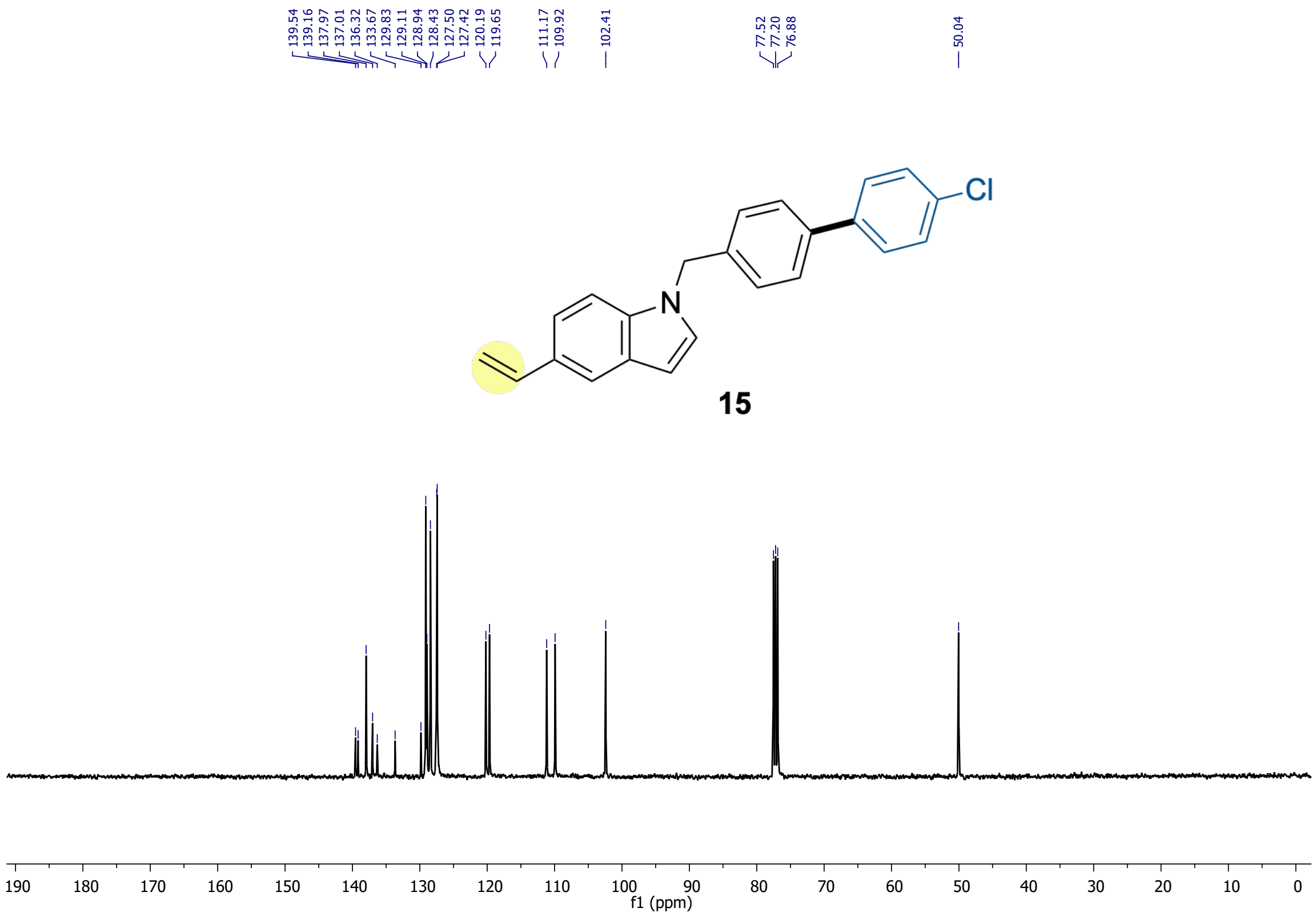

Cl
15

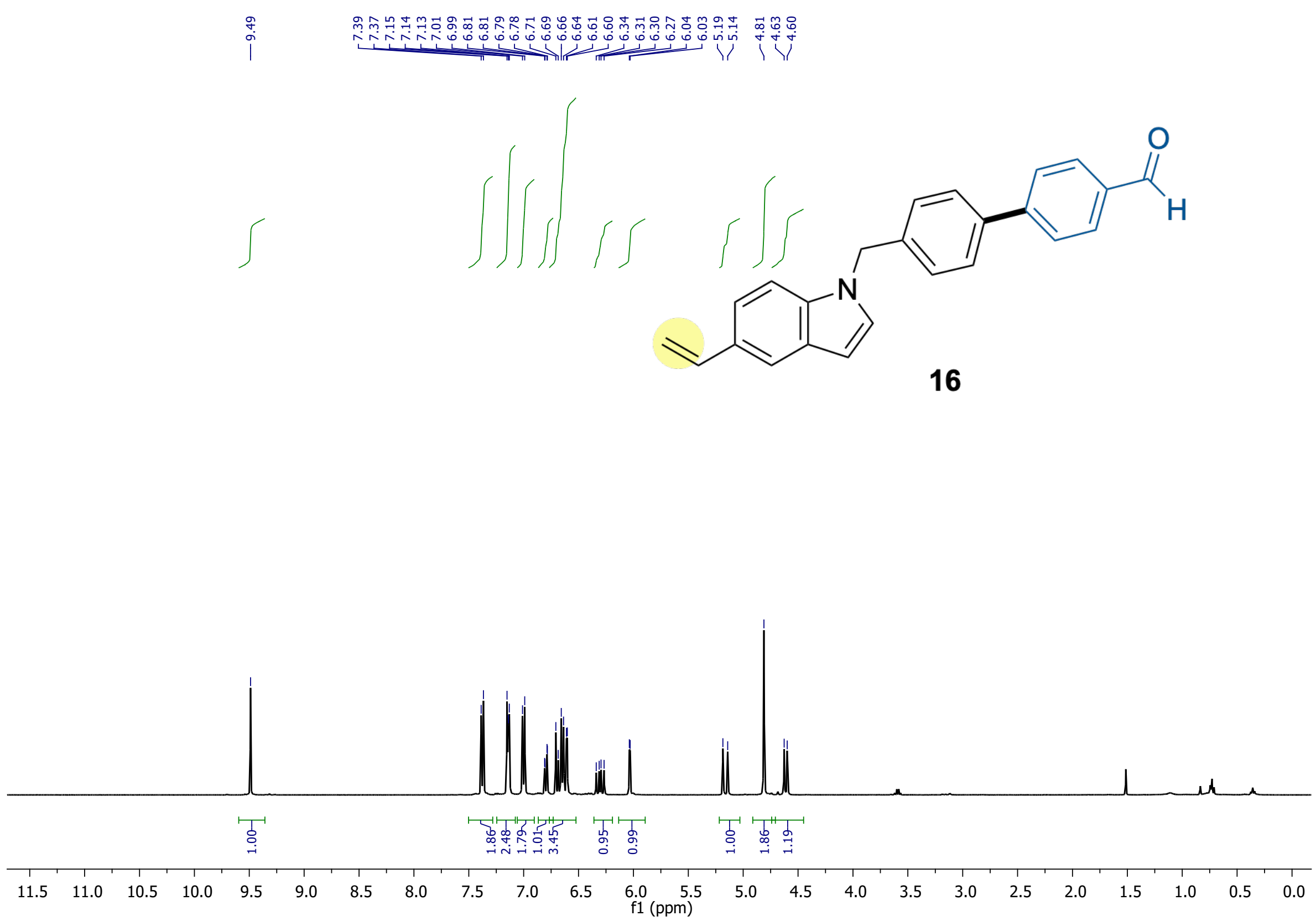

O
H
16

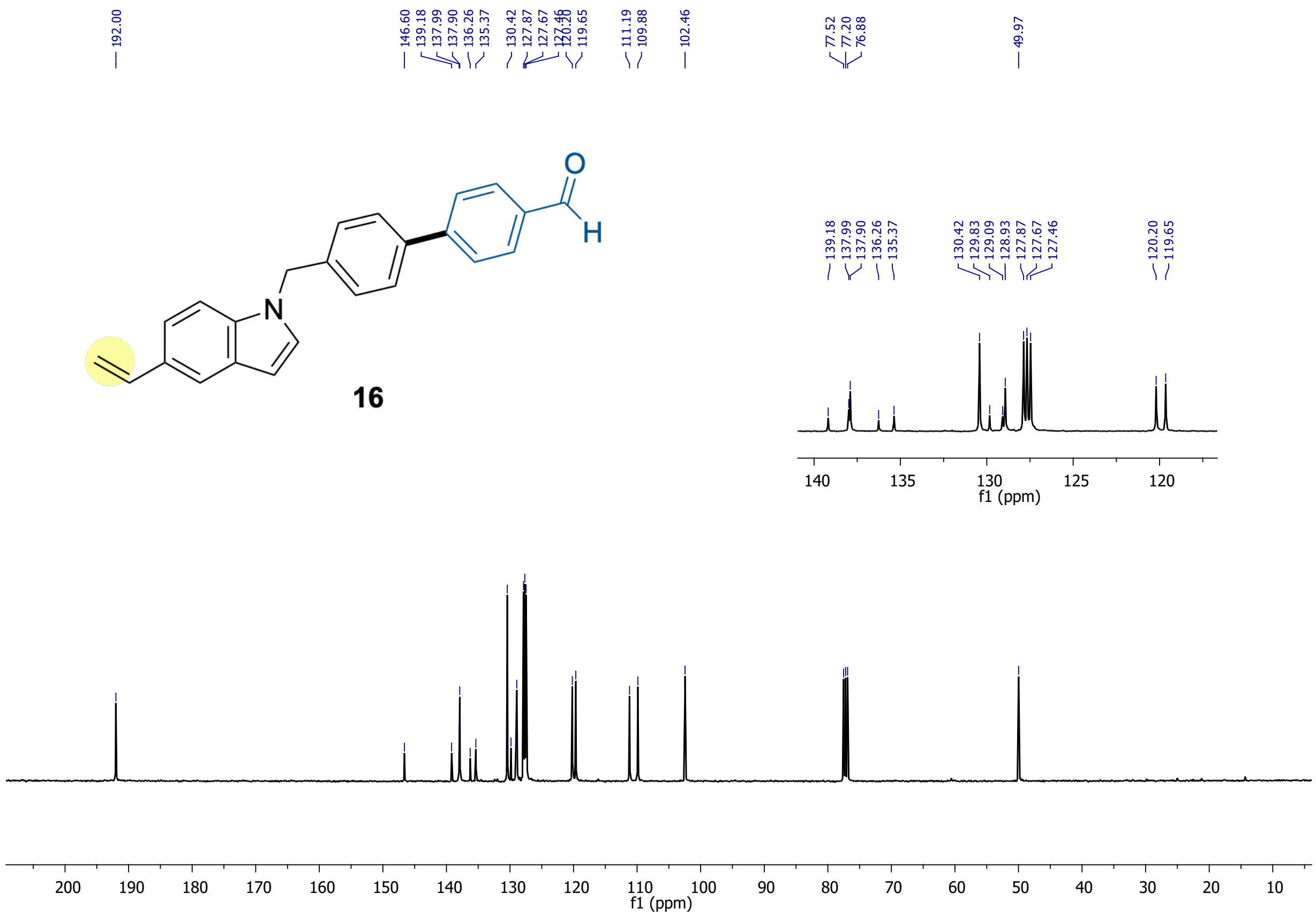

O
H
N
16
192.00
146.60 139.18 137.99 137.90 136.26 135.37 130.42 127.87 127.67 127.46 120.20 119.65
111.19 109.88
102.46
77.52 77.20 76.88
49.97
139.18 137.99 137.90 136.26 135.37 130.42 129.83 129.09 128.93 127.87 127.67 127.46 120.20 119.65
f1 (ppm)

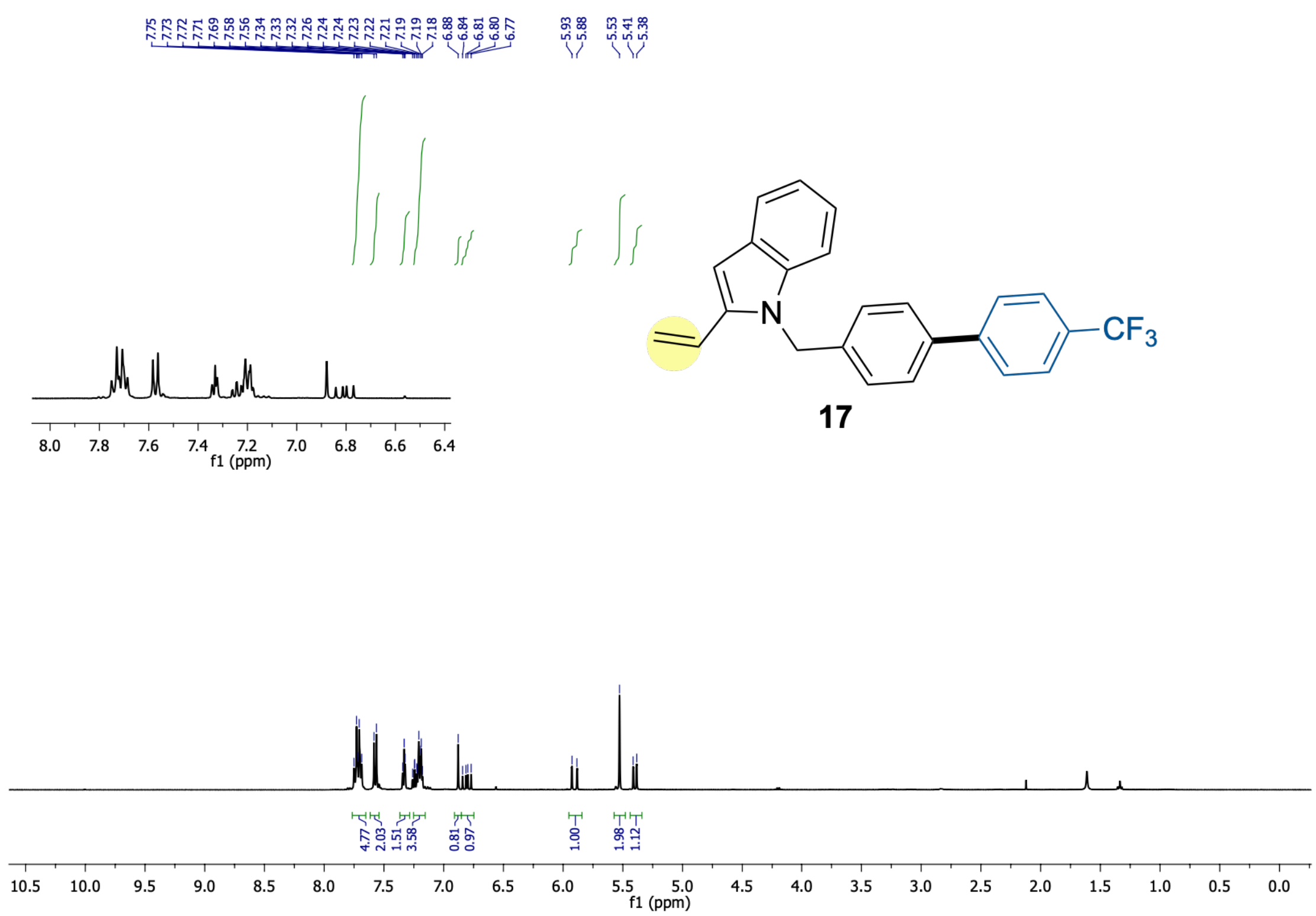

7.75 7.73 7.72 7.71 7.69 7.58 7.56 7.34 7.33 7.32 7.32 7.26 7.24 7.24 7.23 7.23 7.22 7.21 7.19 7.19 7.18 6.88 6.84 6.81 6.80 6.77
5.93 5.88
5.53 5.41 5.38
N
CF$_3$
17
4.77 2.03 1.51 3.58 0.81 0.97 1.00 1.98 1.12
f1 (ppm)

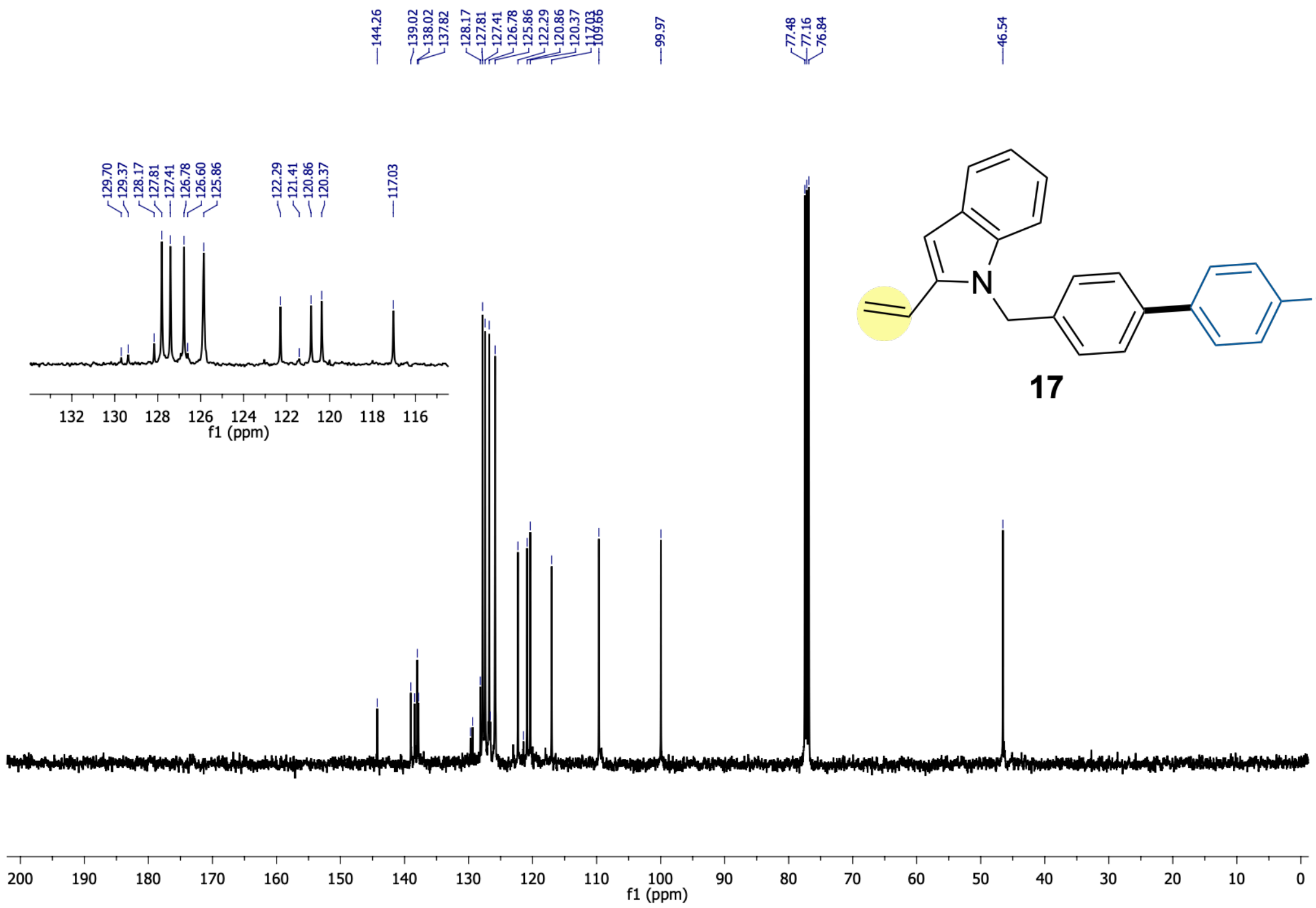
144.26
139.02
138.02
137.82
128.17
127.81
127.41
126.78
125.86
122.29
120.86
120.37
117.03
109.90
99.97
77.48
77.16
76.84
46.54
129.70
129.37
128.17
127.81
127.41
126.78
126.60
125.86
122.29
121.41
120.86
120.37
117.03
132 130 128 126 124 122 120 118 116
f1 (ppm)
N
CF3
17
200 190 180 170 160 150 140 130 120 110 100 90 80 70 60 50 40 30 20 10 0
f1 (ppm)

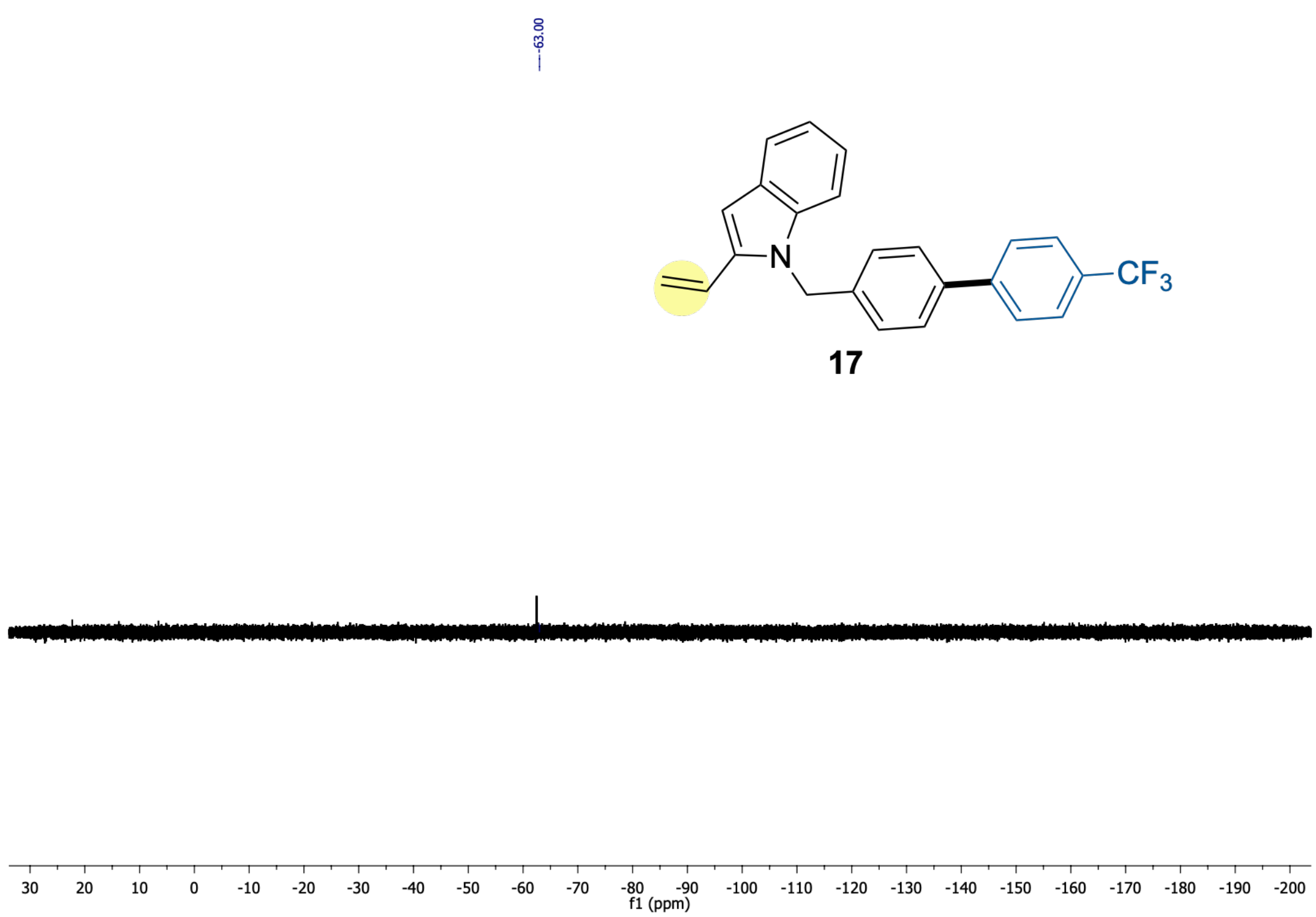
-63.00
N
CF3
17
30 20 10 0 -10 -20 -30 -40 -50 -60 -70 -80 -90 -100 -110 -120 -130 -140 -150 -160 -170 -180 -190 -200
f1 (ppm)

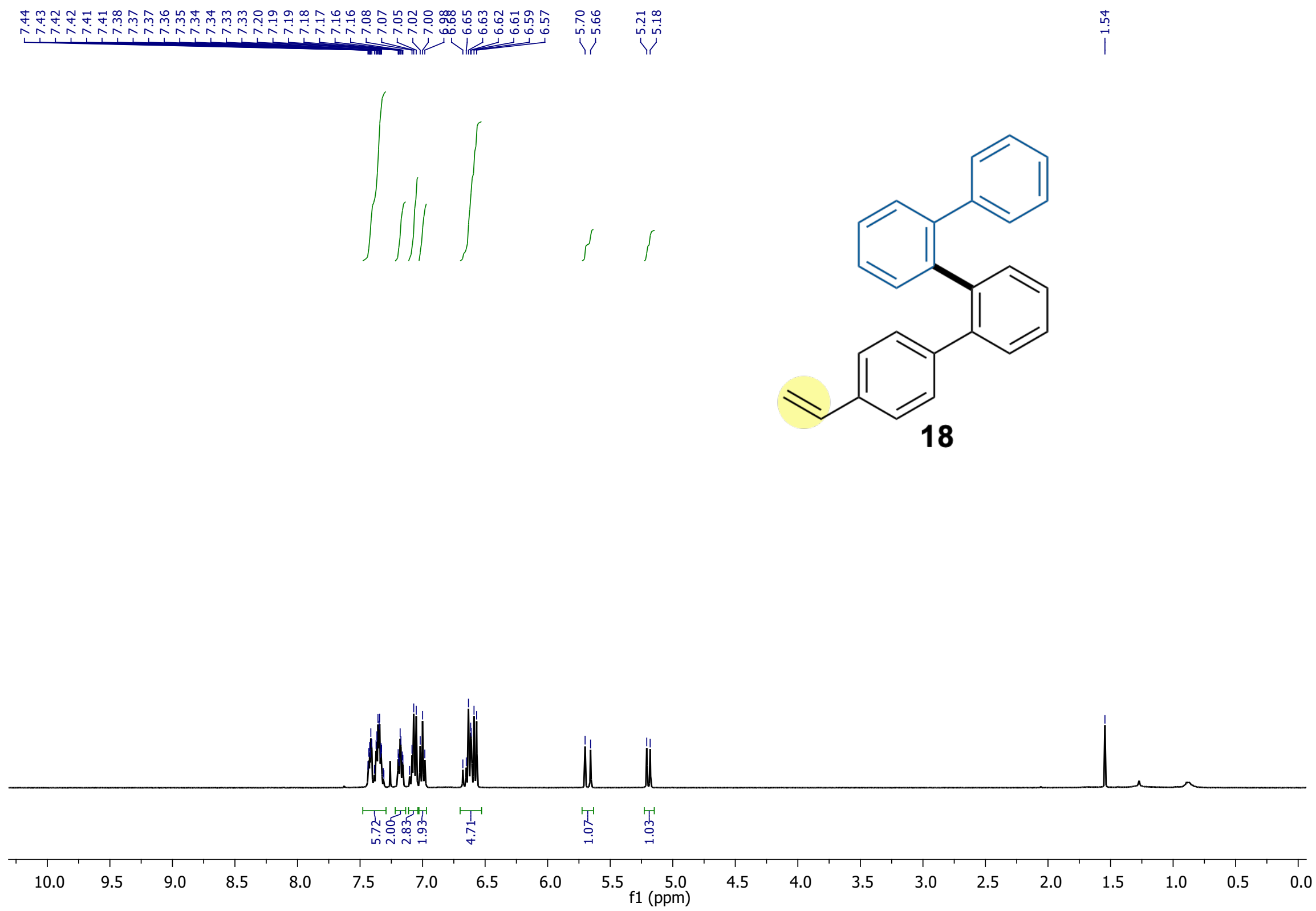
7.44 7.43 7.42 7.42 7.41 7.41 7.38 7.37 7.37 7.36 7.35 7.34 7.34 7.33 7.33 7.20 7.19 7.19 7.18 7.17 7.16 7.16 7.08 7.07 7.05 7.02 7.00 6.98 6.98 6.65 6.63 6.62 6.61 6.59 6.57
5.70 5.66
5.21 5.18
1.54
18
5.72 2.00 2.83 1.93
4.71
1.07
1.03
10.0 9.5 9.0 8.5 8.0 7.5 7.0 6.5 6.0 5.5 5.0 4.5 4.0 3.5 3.0 2.5 2.0 1.5 1.0 0.5 0.0
f1 (ppm)

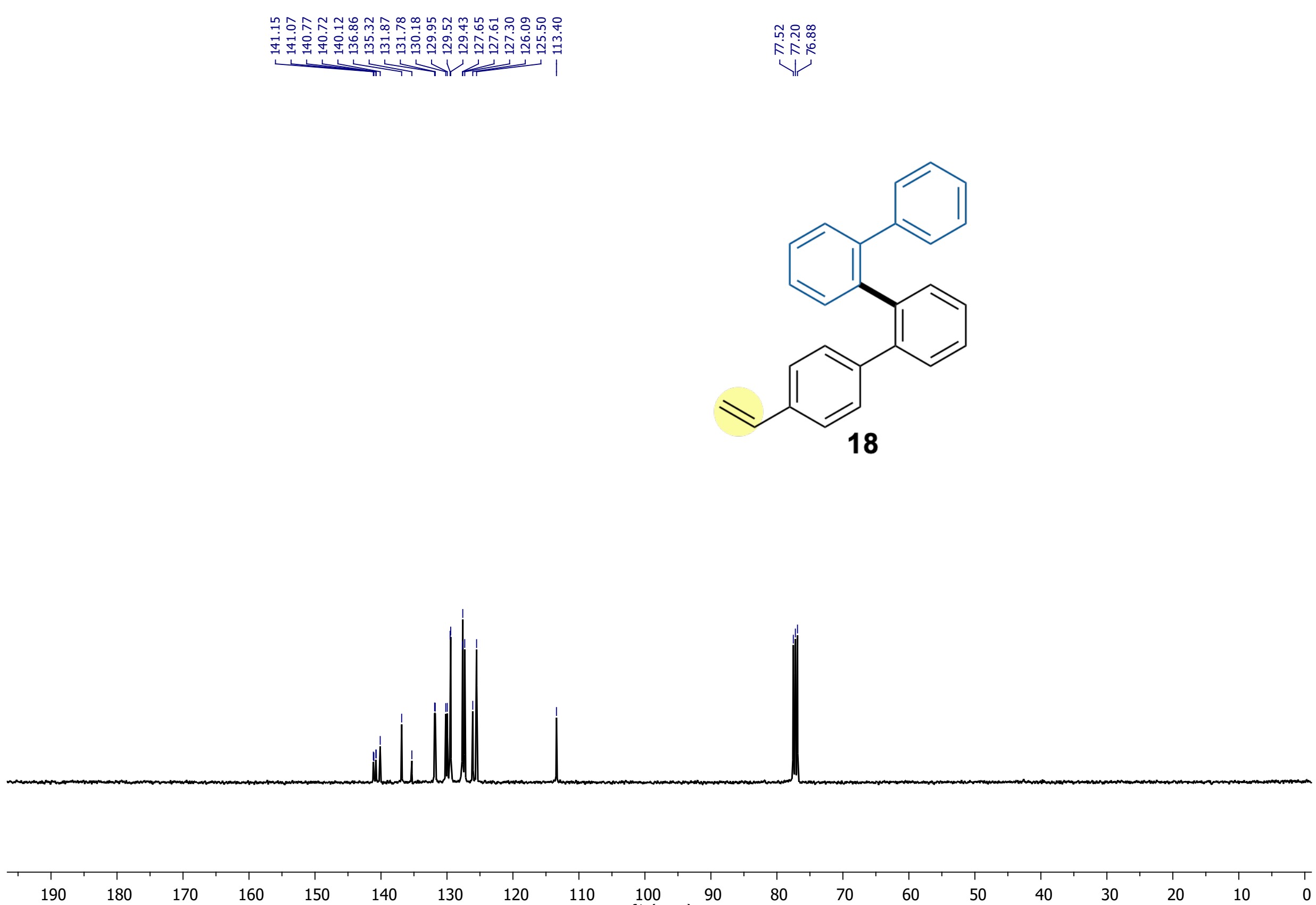
141.15 141.07 140.77 140.72 140.12 136.86 135.32 131.87 131.78 130.18 129.95 129.52 129.43 127.65 127.61 127.30 126.09 125.50 113.40
77.52 77.20 76.88
18
190 180 170 160 150 140 130 120 110 100 90 80 70 60 50 40 30 20 10 0
f1 (ppm)

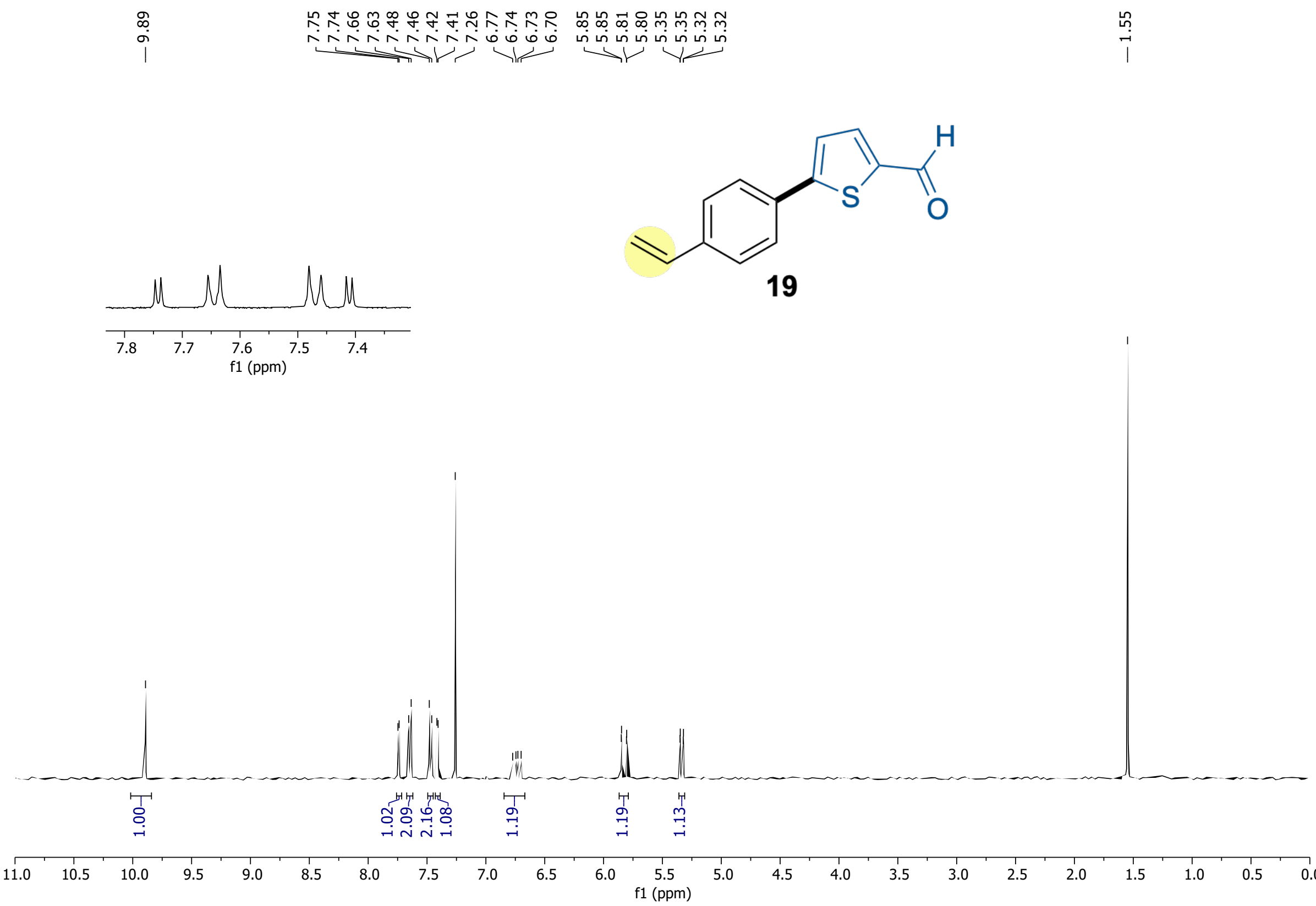
9.89
7.75
7.74
7.66
7.63
7.48
7.46
7.42
7.41
7.26
6.77
6.74
6.73
6.70
5.85
5.85
5.81
5.80
5.35
5.35
5.32
5.32
1.55
H
S
O
19
f1 (ppm)
1.00
1.02
2.09
2.16
1.08
1.19
1.19
1.13

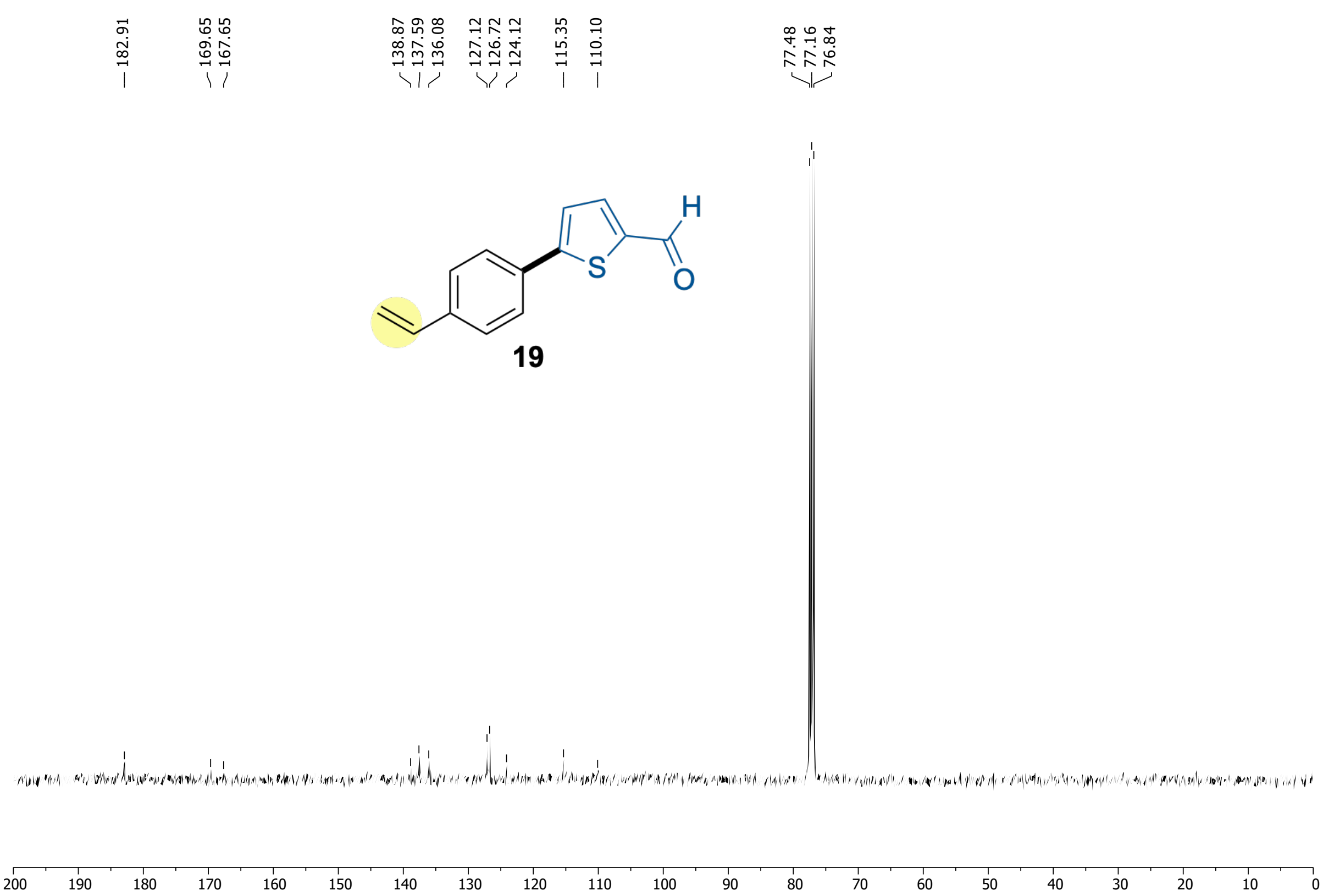
182.91
169.65
167.65
138.87
137.59
136.08
127.12
126.72
124.12
115.35
110.10
77.48
77.16
76.84
H
S
O
19
f1 (ppm)

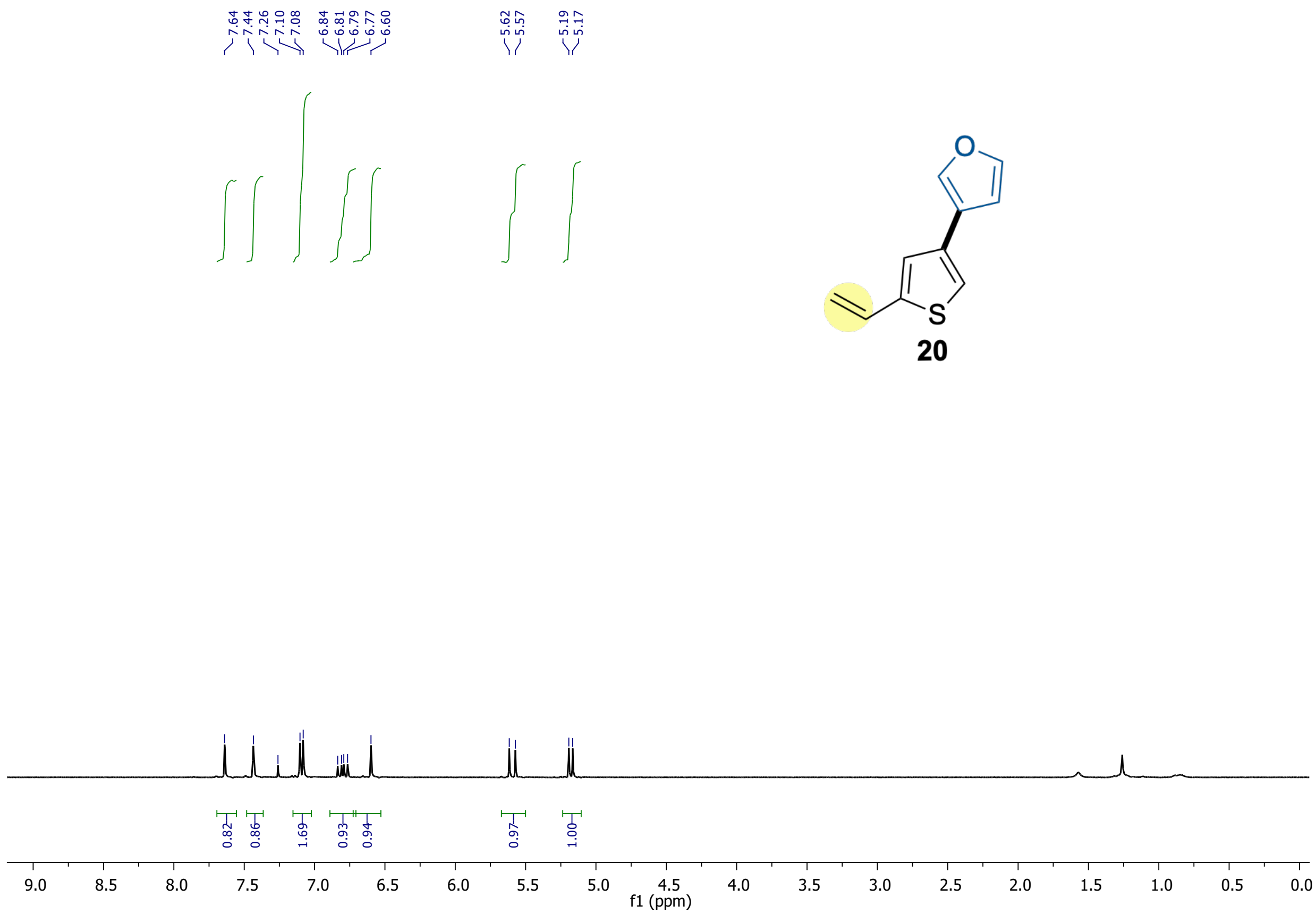
7.64
7.44
7.26
7.10
7.08
6.84
6.81
6.79
6.77
6.60
5.62
5.57
5.19
5.17
O
S
20
0.82
0.86
1.69
0.93
0.94
0.97
1.00
f1 (ppm)

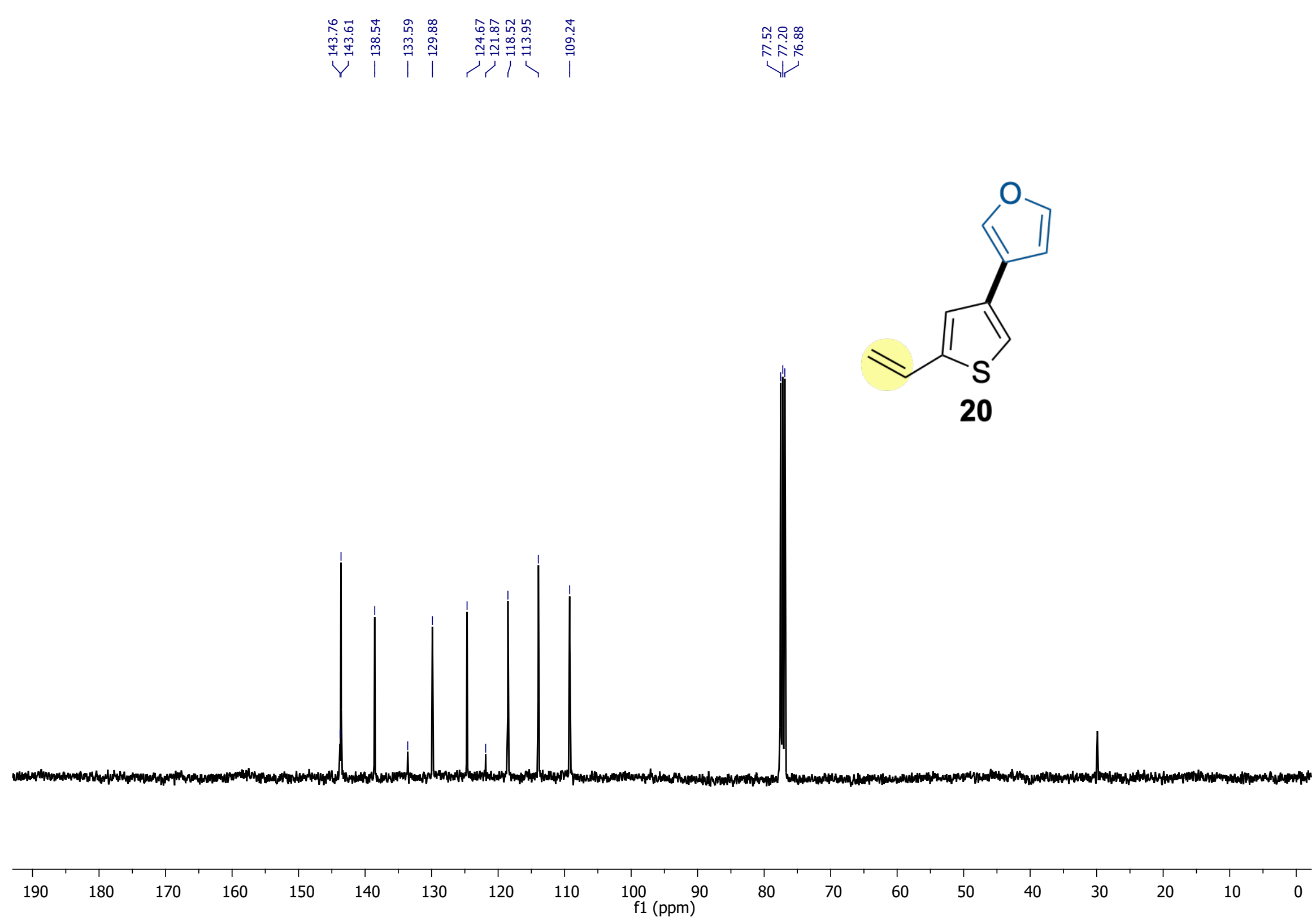
143.76
143.61
138.54
133.59
129.88
124.67
121.87
118.52
113.95
109.24
77.52
77.20
76.88
O
S
20
f1 (ppm)

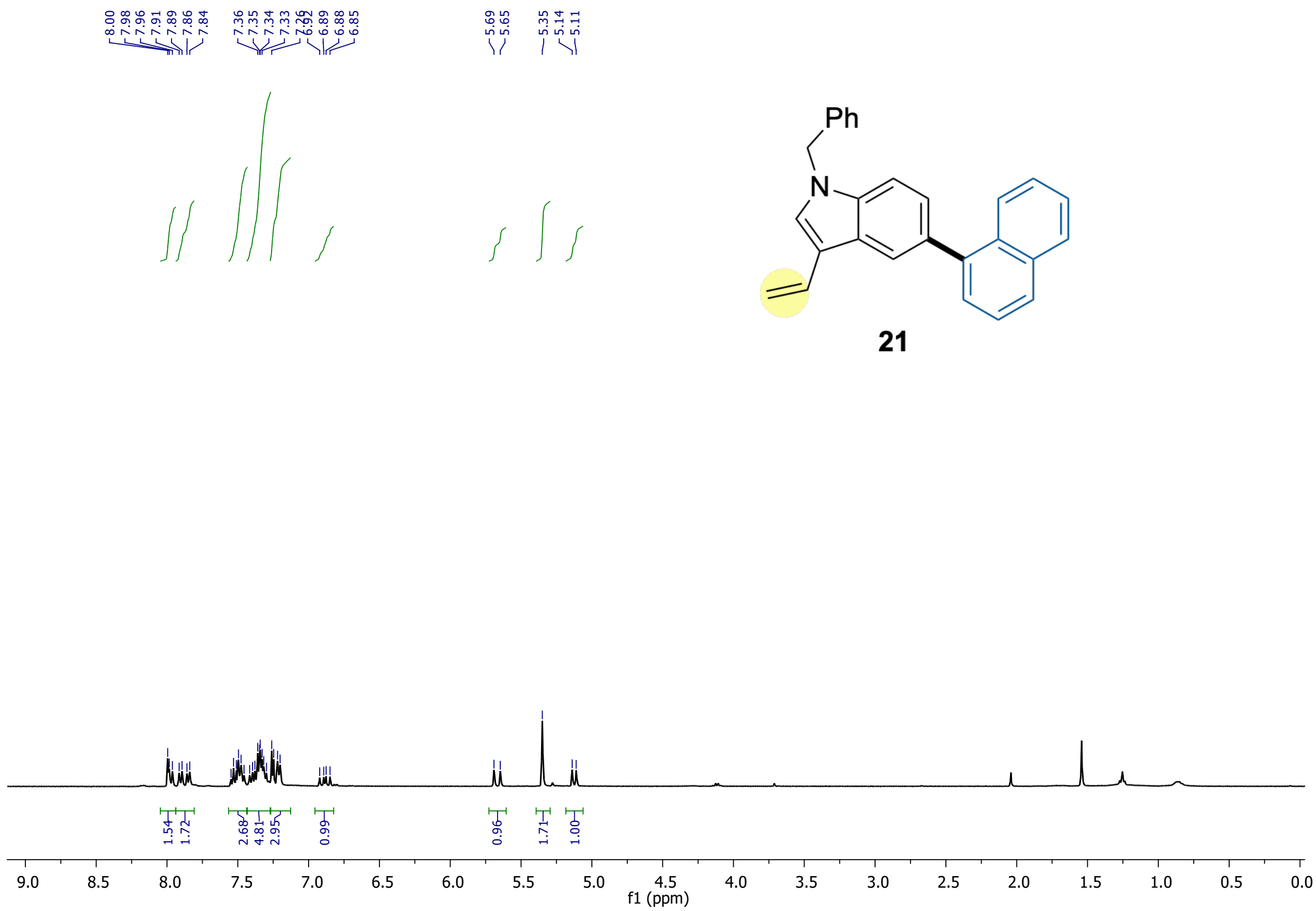
8.00
7.98
7.96
7.91
7.89
7.86
7.84
7.36
7.35
7.34
7.33
7.26
6.92
6.89
6.88
6.85
5.69
5.65
5.35
5.14
5.11
Ph
N
21
1.54
1.72
2.68
4.81
2.95
0.99
0.96
1.71
1.00
9.0 8.5 8.0 7.5 7.0 6.5 6.0 5.5 5.0 4.5 4.0 3.5 3.0 2.5 2.0 1.5 1.0 0.5 0.0
f1 (ppm)

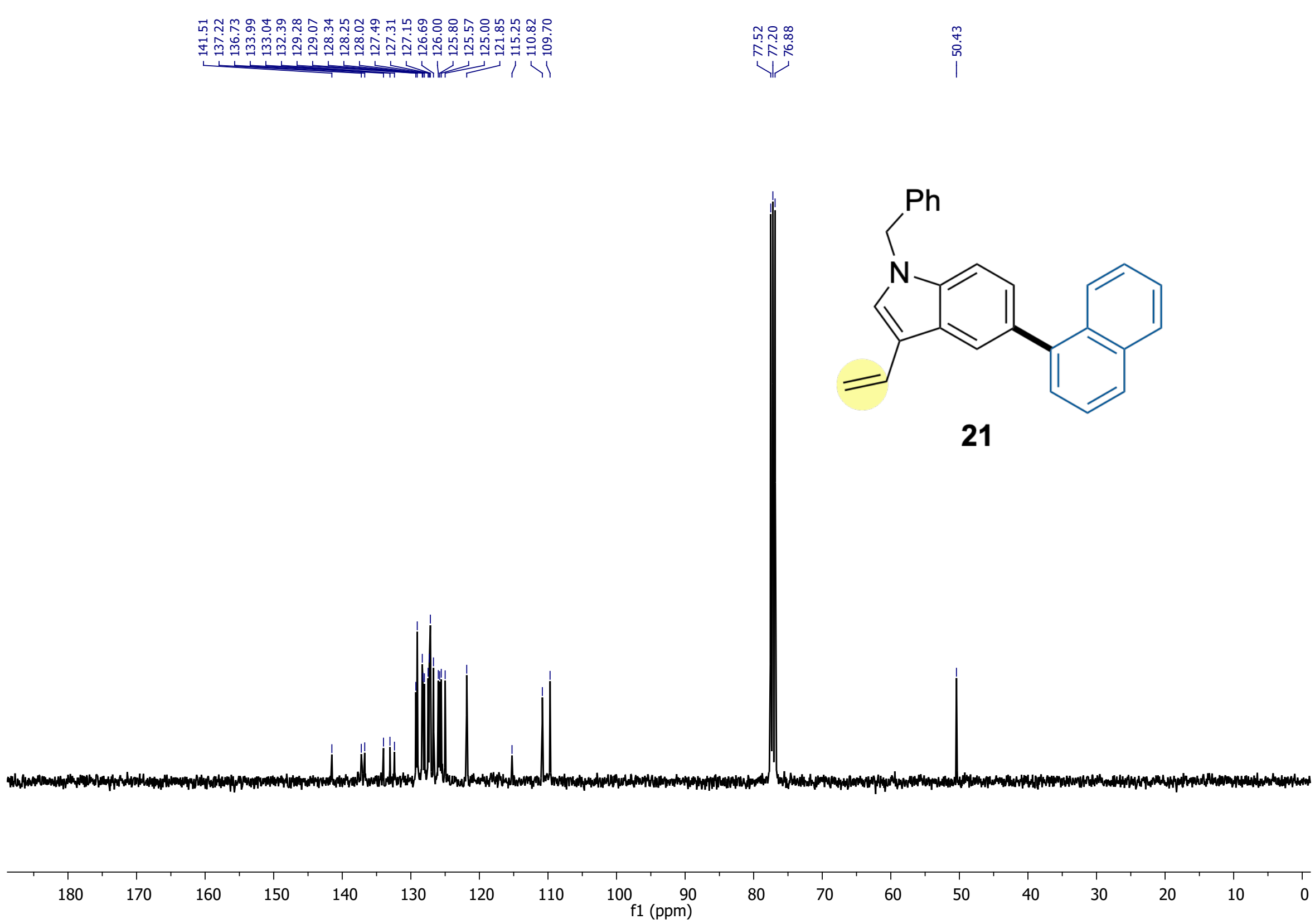
141.51
137.22
136.73
133.99
133.04
132.39
129.28
129.07
128.34
128.25
128.02
127.49
127.31
127.15
126.69
126.00
125.80
125.57
125.00
121.85
115.25
110.82
109.70
77.52
77.20
76.88
50.43
Ph
N
21
180 170 160 150 140 130 120 110 100 90 80 70 60 50 40 30 20 10 0
f1 (ppm)

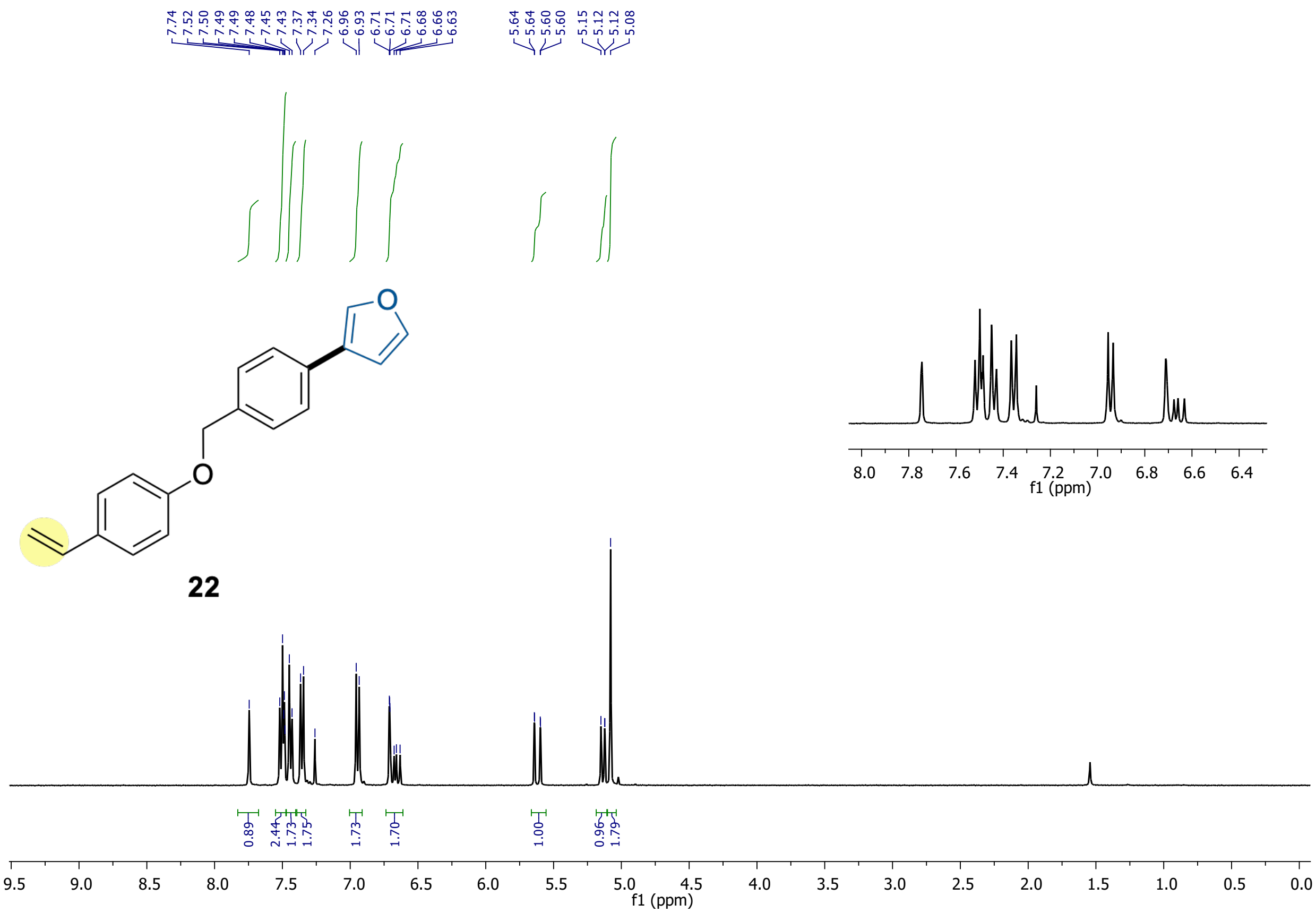
7.74 7.52 7.50 7.49 7.49 7.48 7.45 7.43 7.37 7.34 7.26 6.96 6.93 6.71 6.71 6.71 6.68 6.66 6.63
5.64 5.64 5.60 5.60 5.15 5.12 5.12 5.08
0.89 2.44 1.73 1.75 1.73 1.70 1.00 0.96 1.79
f1 (ppm)

22

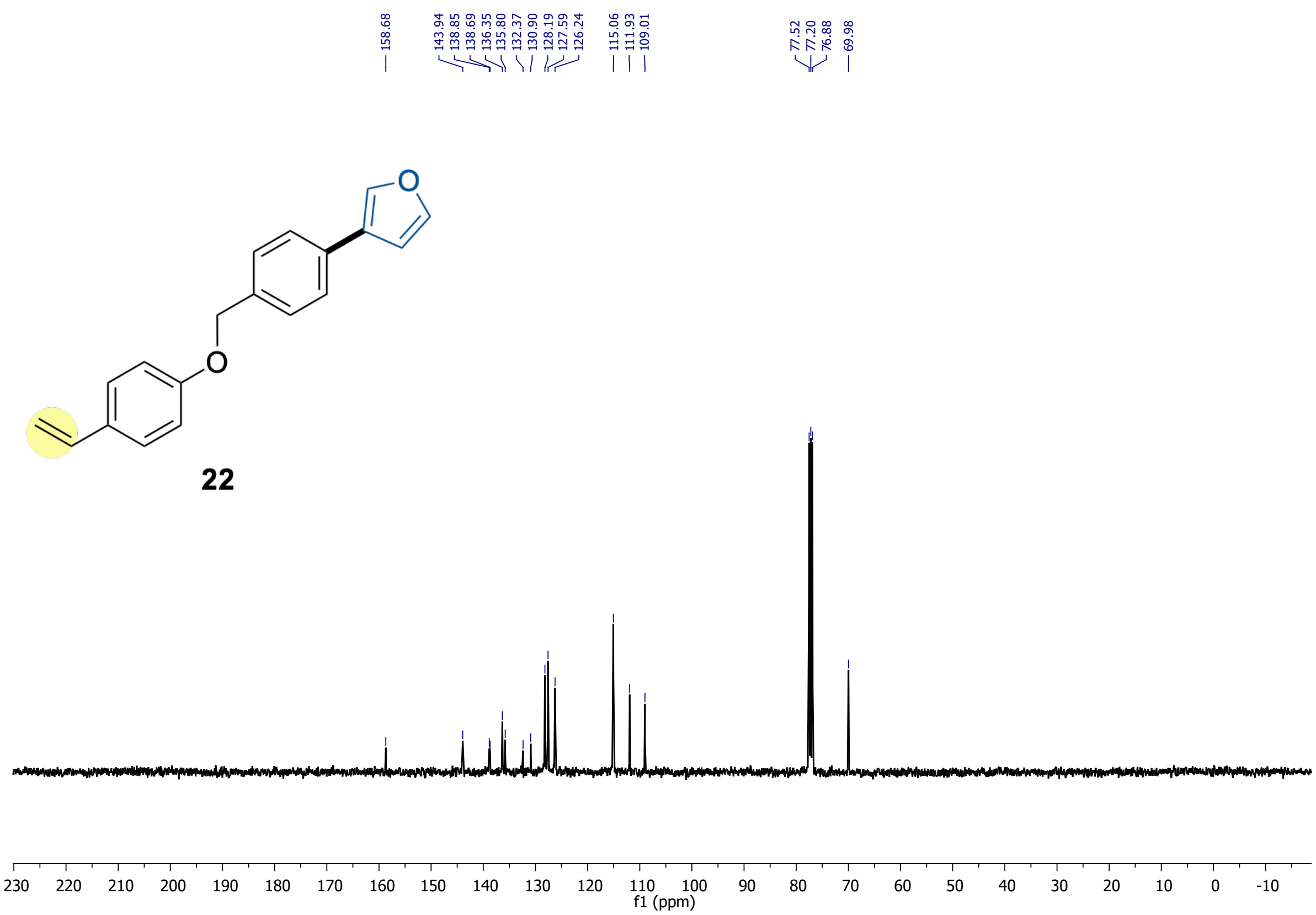
158.68
143.94 138.85 138.69 136.35 135.80 132.37 130.90 128.19 127.59 126.24
115.06 111.93 109.01
77.52 77.20 76.88 69.98
f1 (ppm)

22

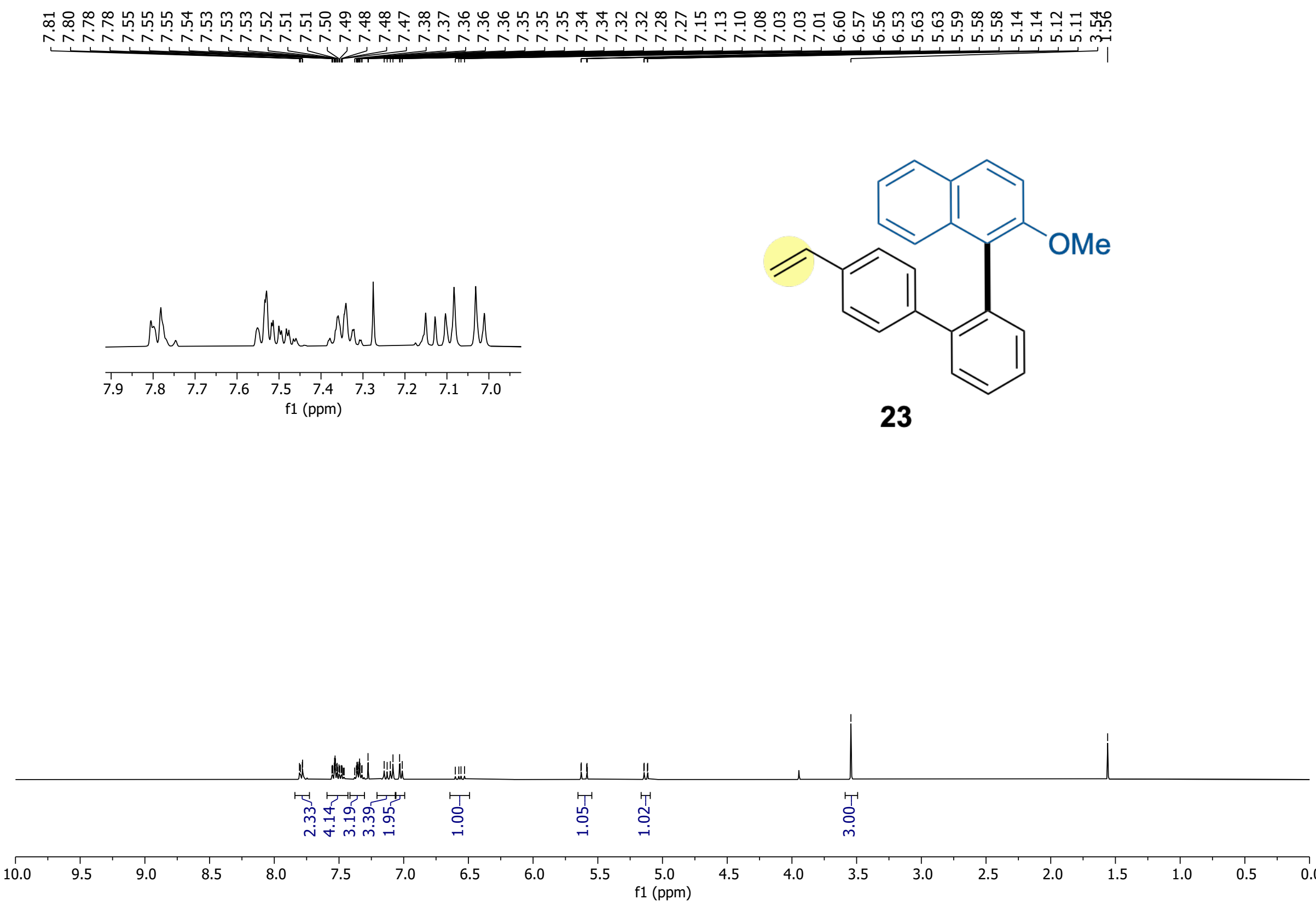
7.81 7.80 7.78 7.78 7.55 7.55 7.55 7.54 7.53 7.53 7.53 7.52 7.51 7.51 7.50 7.49 7.48 7.48 7.47 7.38 7.37 7.36 7.36 7.36 7.35 7.35 7.35 7.34 7.34 7.32 7.32 7.28 7.27 7.15 7.13 7.10 7.08 7.03 7.03 7.01 6.60 6.57 6.56 6.53 5.63 5.59 5.58 5.58 5.14 5.14 5.12 5.11 3.54 1.56
OMe
23
2.33 4.14 3.19 3.39 1.95 1.00 1.05 1.02 3.00
f1 (ppm)

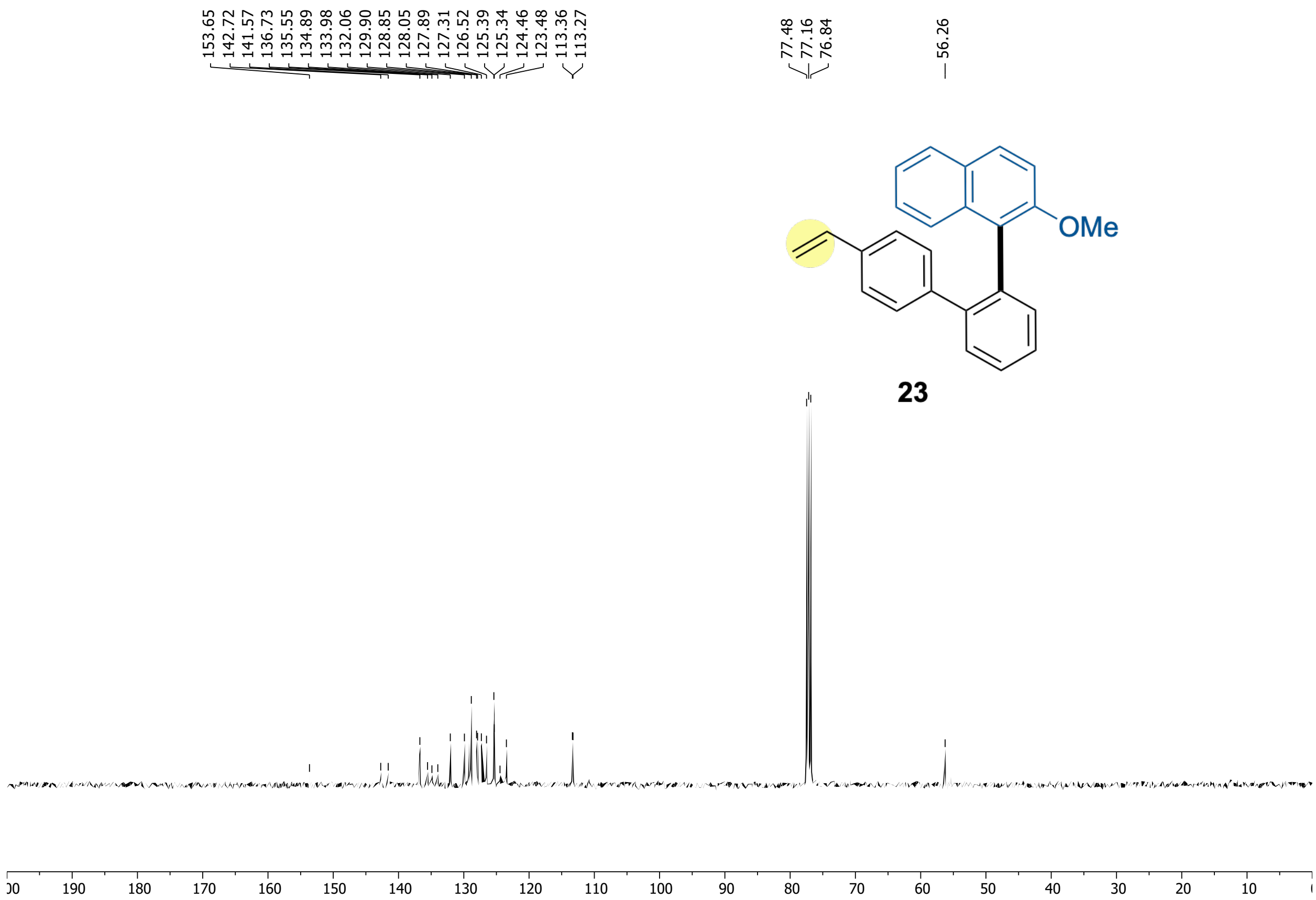
153.65 142.72 141.57 136.73 135.55 134.89 133.98 132.06 129.90 128.85 128.05 127.89 127.31 126.52 125.39 125.34 124.46 123.48 113.36 113.27
77.48 77.16 76.84
56.26
OMe
23
f1 (ppm)

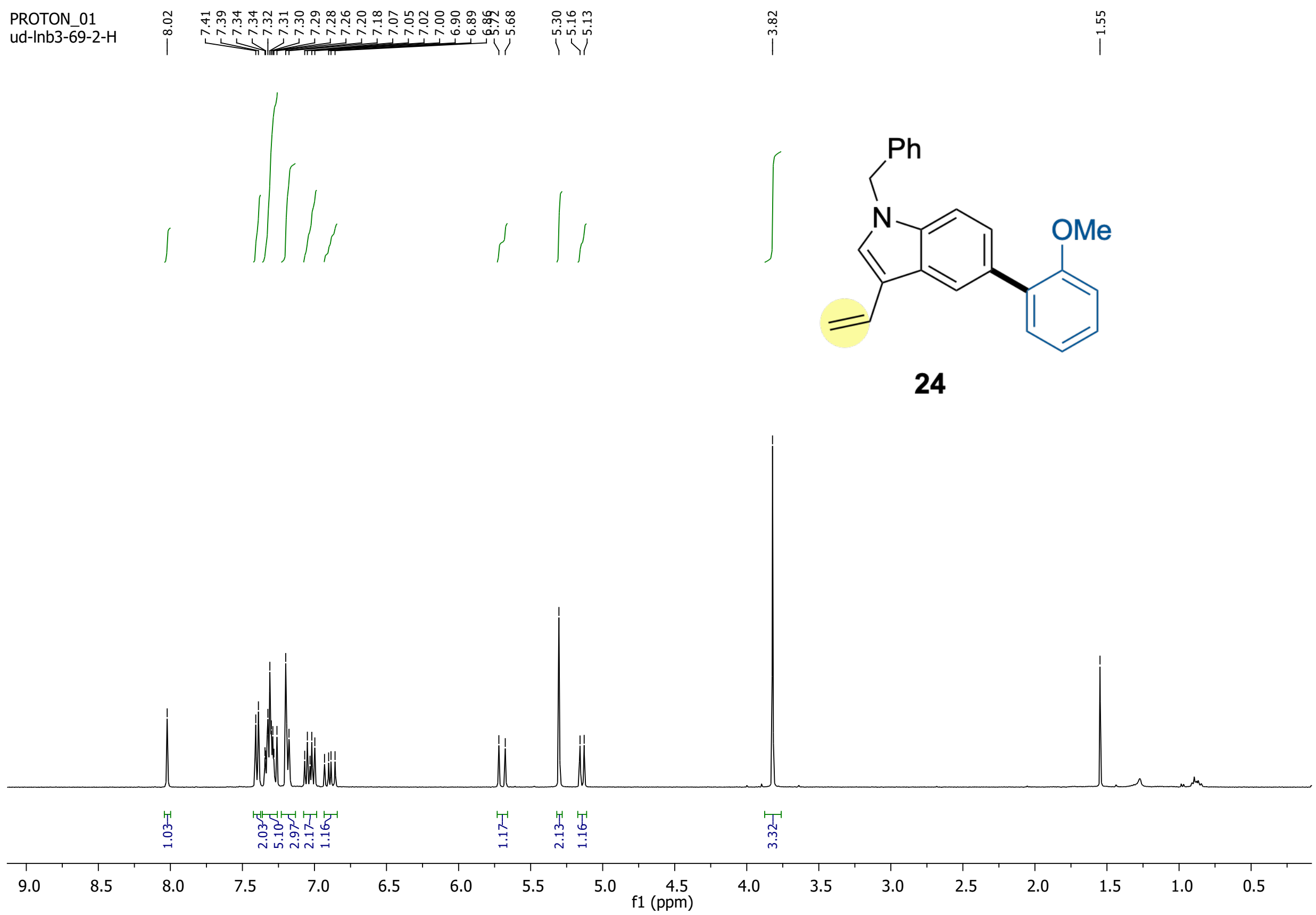
PROTON_01
ud-lnb3-69-2-H
8.02
7.41 7.39 7.34 7.34 7.32 7.31 7.30 7.29 7.28 7.26 7.20 7.18 7.07 7.05 7.02 7.00 6.90 6.89 6.86 5.72 5.68
5.30 5.16 5.13
3.82
1.55
Ph
N
OMe
24
1.03
2.03 5.10 2.97 2.17 1.16
1.17
2.13 1.16
3.32
f1 (ppm)

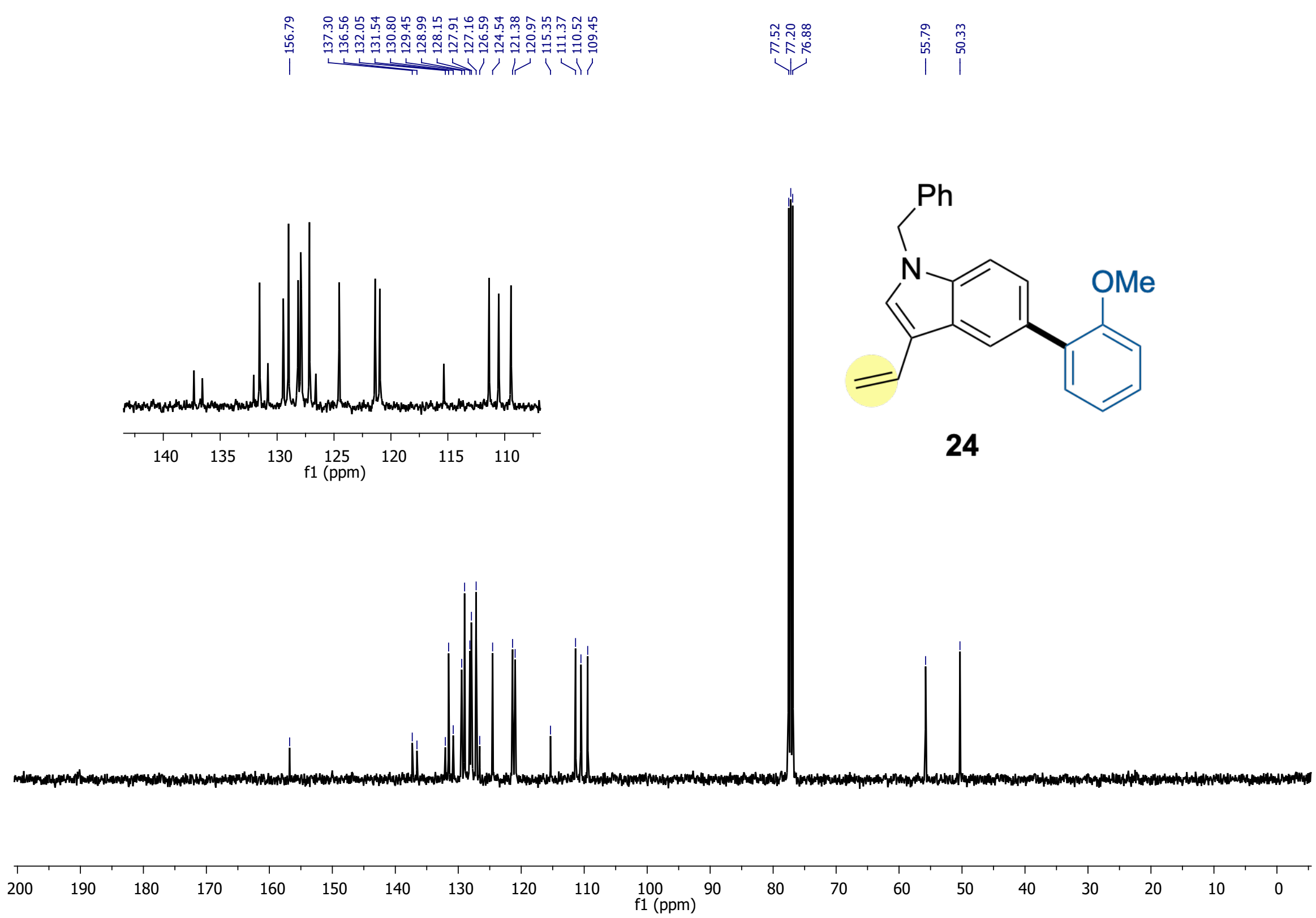
156.79
137.30 136.56 132.05 131.54 130.80 129.45 128.99 128.15 127.91 127.16 126.59 124.54 121.38 120.97 115.35 111.37 110.52 109.45
77.52 77.20 76.88
55.79
50.33
Ph
N
OMe
24
f1 (ppm)

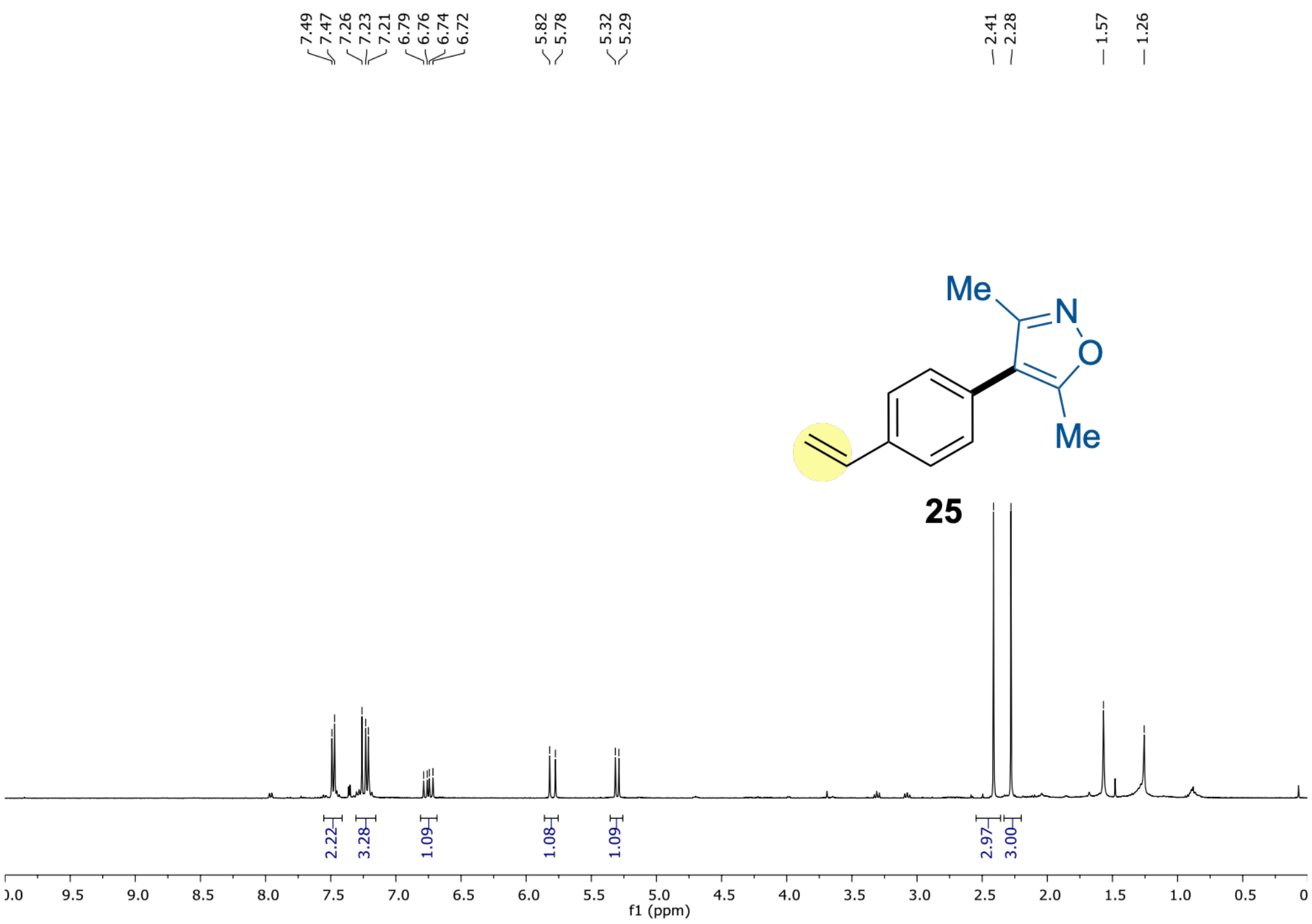
7.49
7.47
7.26
7.23
7.21
6.79
6.76
6.74
6.72
5.82
5.78
5.32
5.29
2.41
2.28
1.57
1.26
Me
N
O
Me
25
2.22
3.28
1.09
1.08
1.09
2.97
3.00
f1 (ppm)

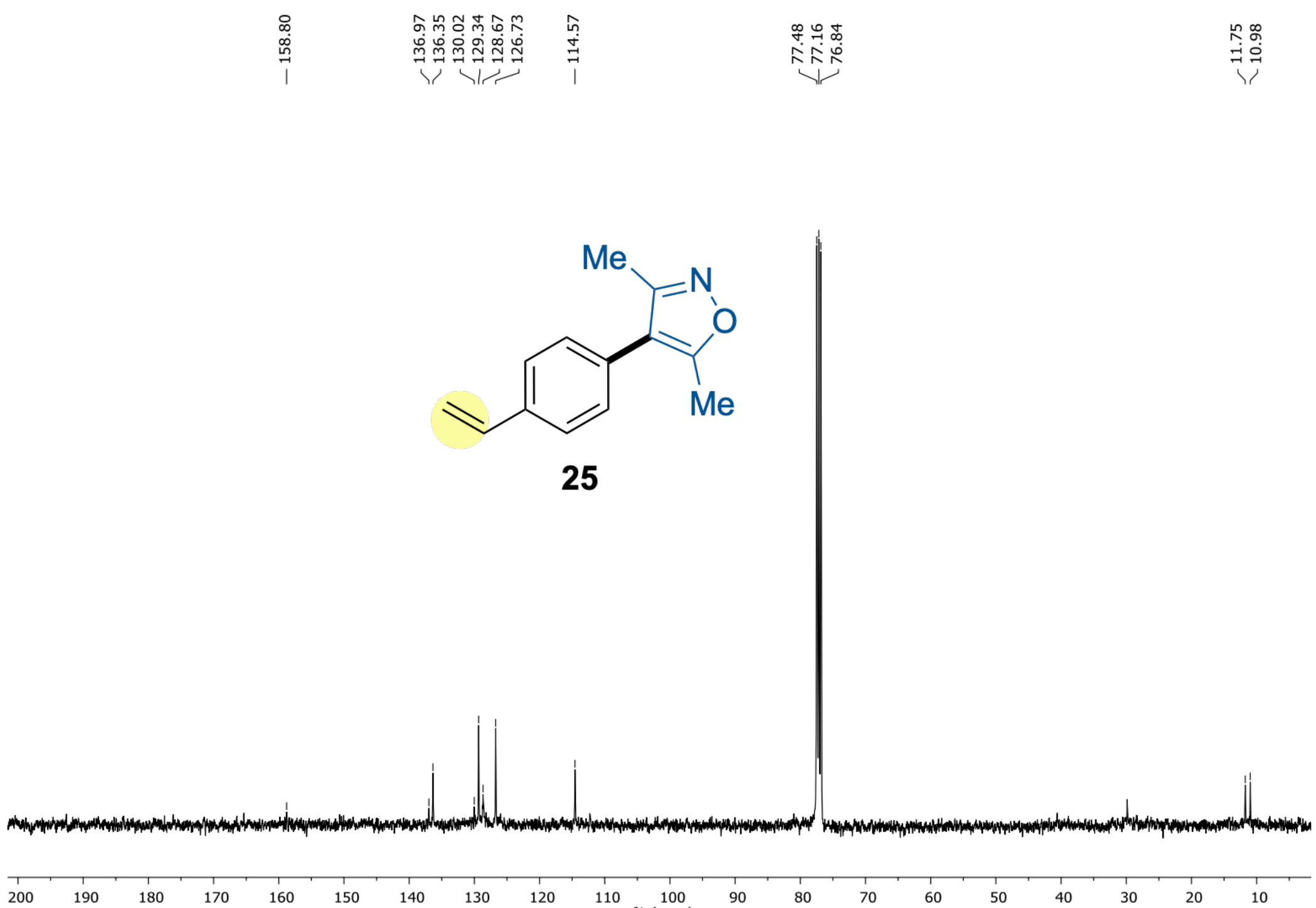
158.80
136.97
136.35
130.02
129.34
128.67
126.73
114.57
77.48
77.16
76.84
11.75
10.98
Me
N
O
Me
25
f1 (ppm)

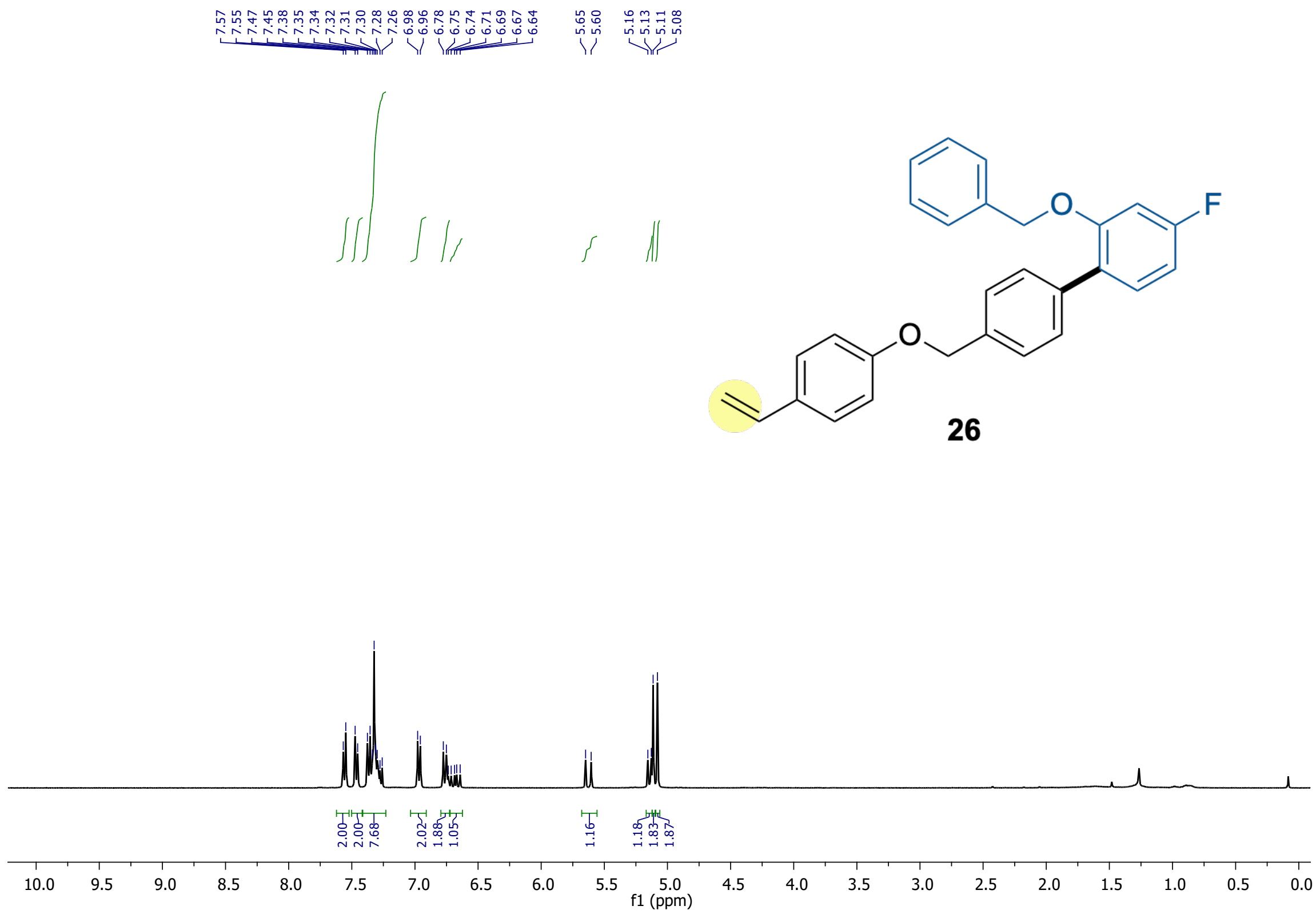
7.57 7.55 7.47 7.45 7.38 7.35 7.34 7.32 7.31 7.30 7.28 7.26 6.98 6.96 6.78 6.75 6.74 6.71 6.69 6.67 6.64
5.65 5.60
5.16 5.13 5.11 5.08
O
F
O
26
2.00 2.00 7.68 2.02 1.88 1.05 1.16 1.18 1.83 1.87
10.0 9.5 9.0 8.5 8.0 7.5 7.0 6.5 6.0 5.5 5.0 4.5 4.0 3.5 3.0 2.5 2.0 1.5 1.0 0.5 0.0
f1 (ppm)

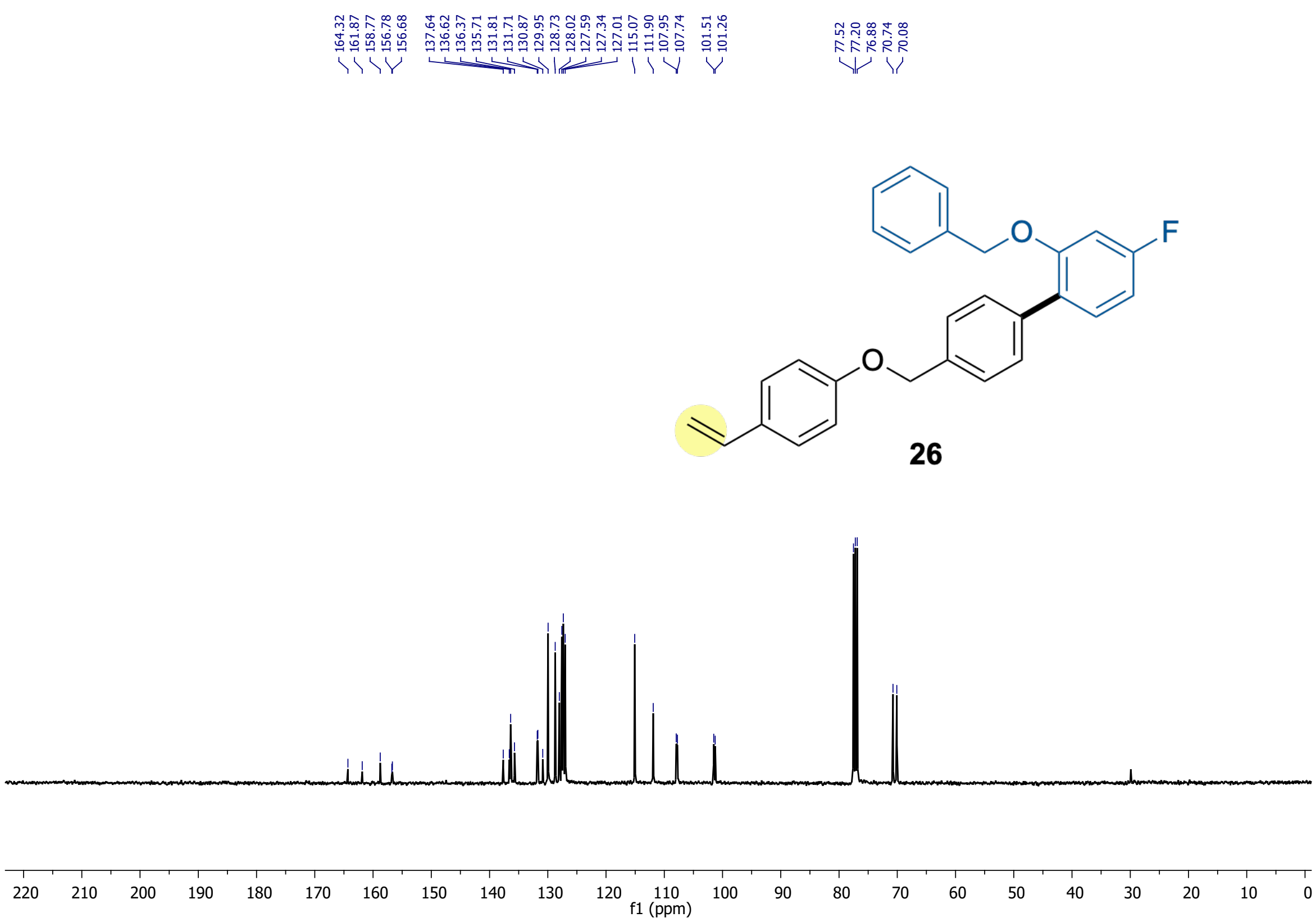
164.32 161.87 158.77 156.78 156.68
137.64 136.62 136.37 135.71 131.81 131.71 130.87 129.95 128.73 128.02 127.59 127.34 127.01 115.07 111.90 107.95 107.74
101.51 101.26
77.52 77.20 76.88 70.74 70.08
O
F
O
26
220 210 200 190 180 170 160 150 140 130 120 110 100 90 80 70 60 50 40 30 20 10 0
f1 (ppm)

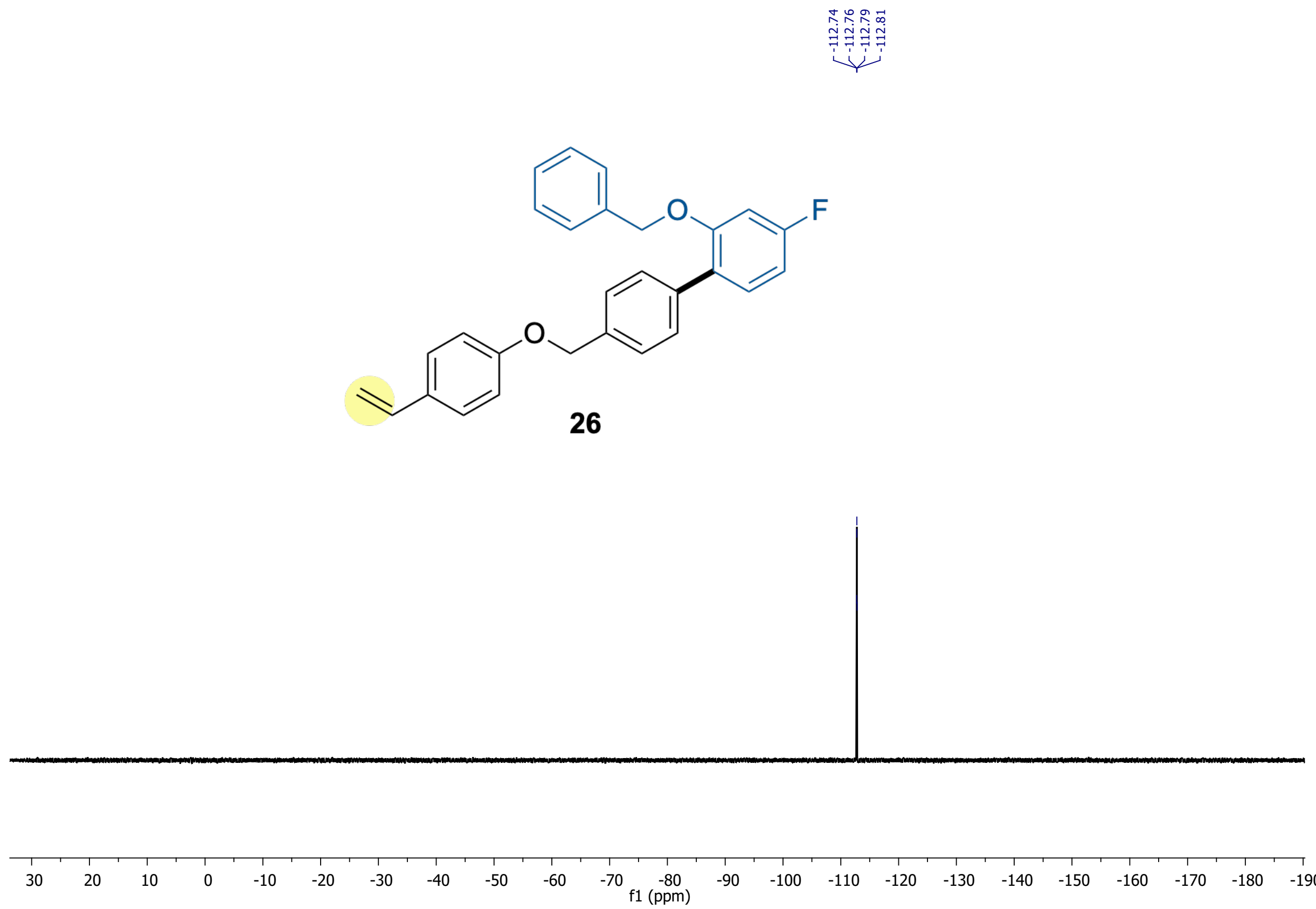
-112.74
-112.76
-112.79
-112.81
O
F
f1 (ppm)

26

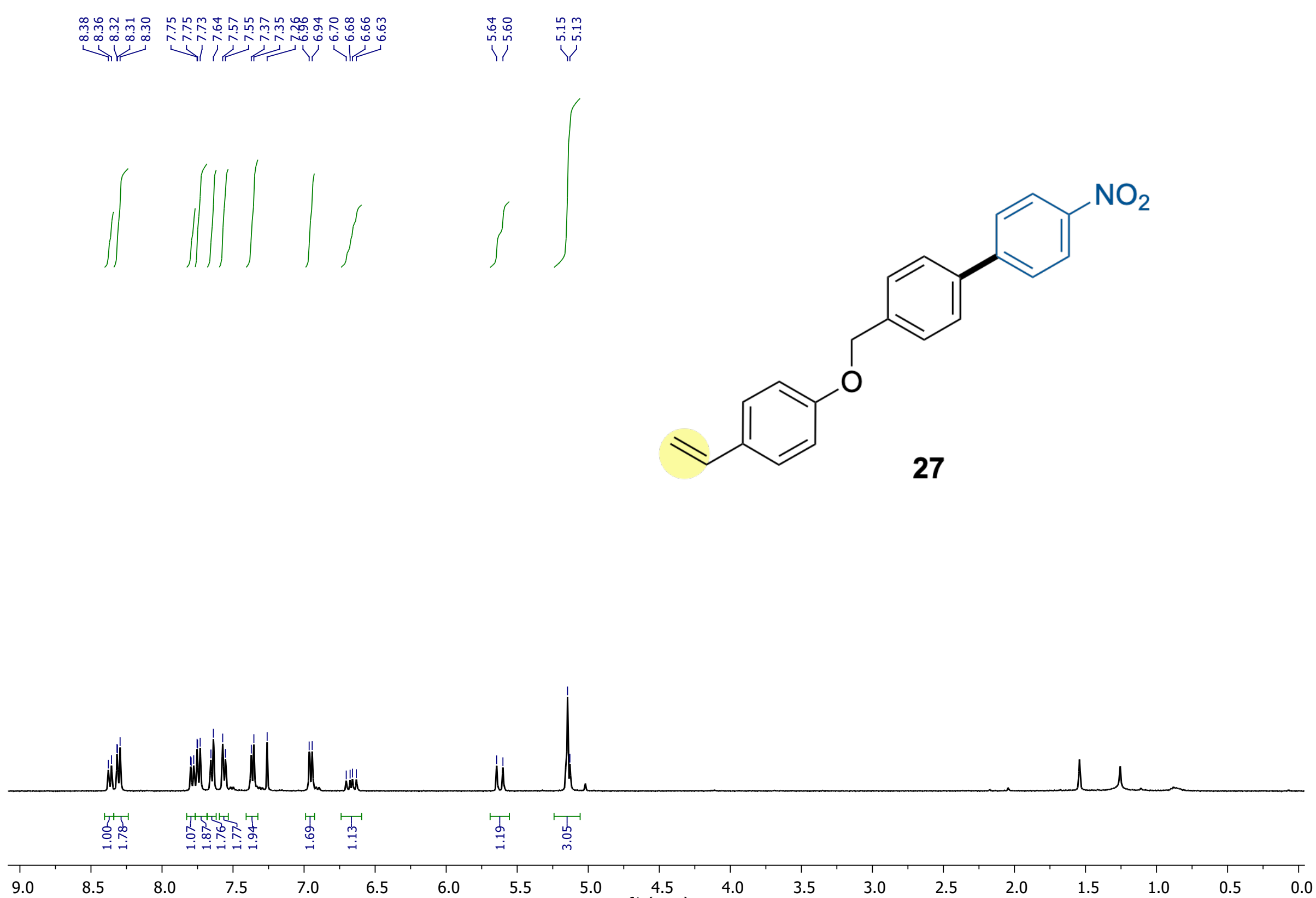
8.38
8.36
8.32
8.31
8.30
7.75
7.75
7.73
7.64
7.57
7.55
7.37
7.35
7.26
6.96
6.94
6.70
6.68
6.66
6.63
5.64
5.60
5.15
5.13
NO2
O
1.00
1.78
1.07
1.87
1.76
1.77
1.94
1.69
1.13
1.19
3.05
f1 (ppm)

27

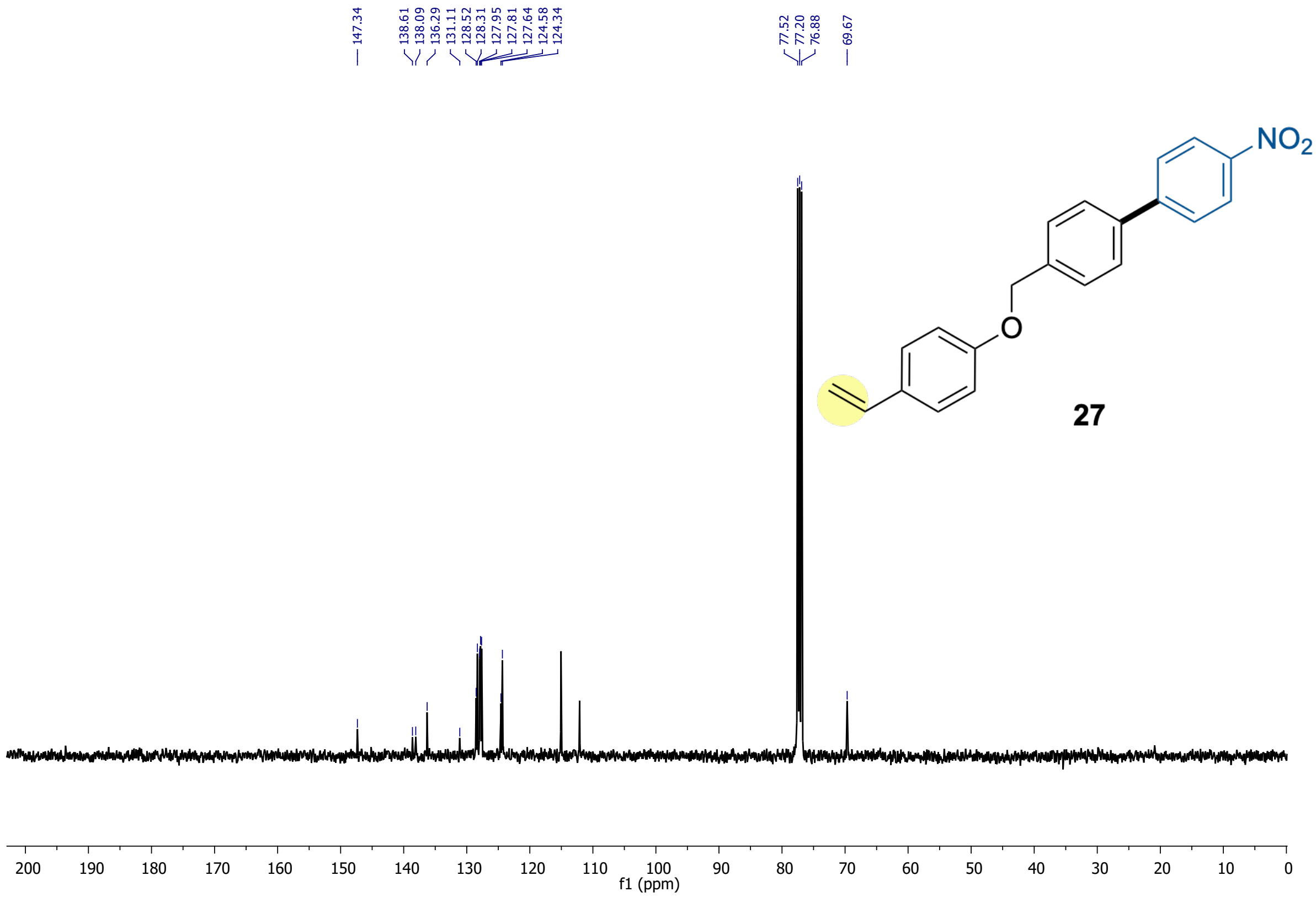
147.34
138.61
138.09
136.29
131.11
128.52
128.31
127.95
127.81
127.64
124.58
124.34
77.52
77.20
76.88
69.67
NO2
O
27
f1 (ppm)

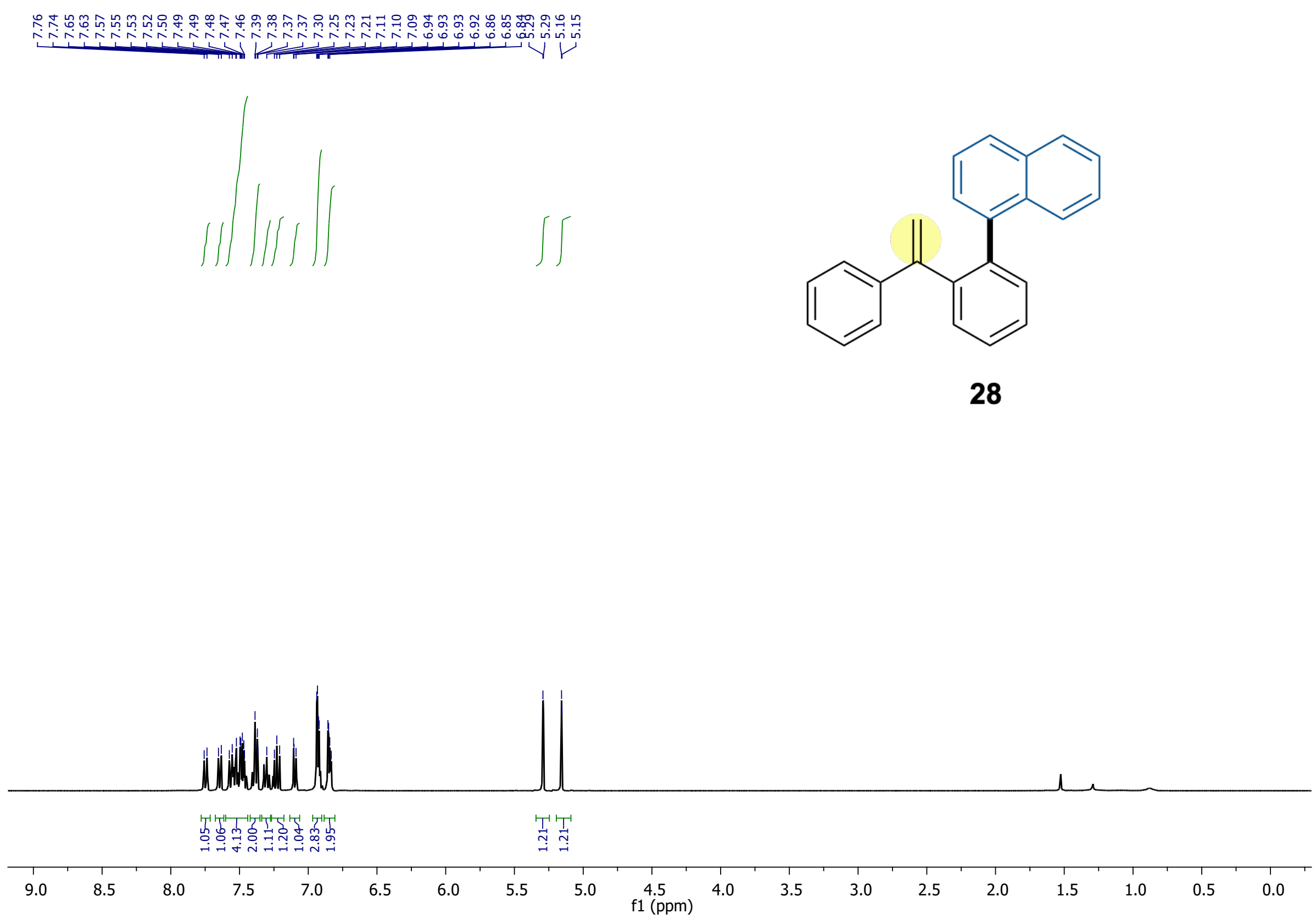
28
1.05
1.06
4.13
2.00
1.11
1.20
1.04
2.83
1.95
1.21
1.21
f1 (ppm)

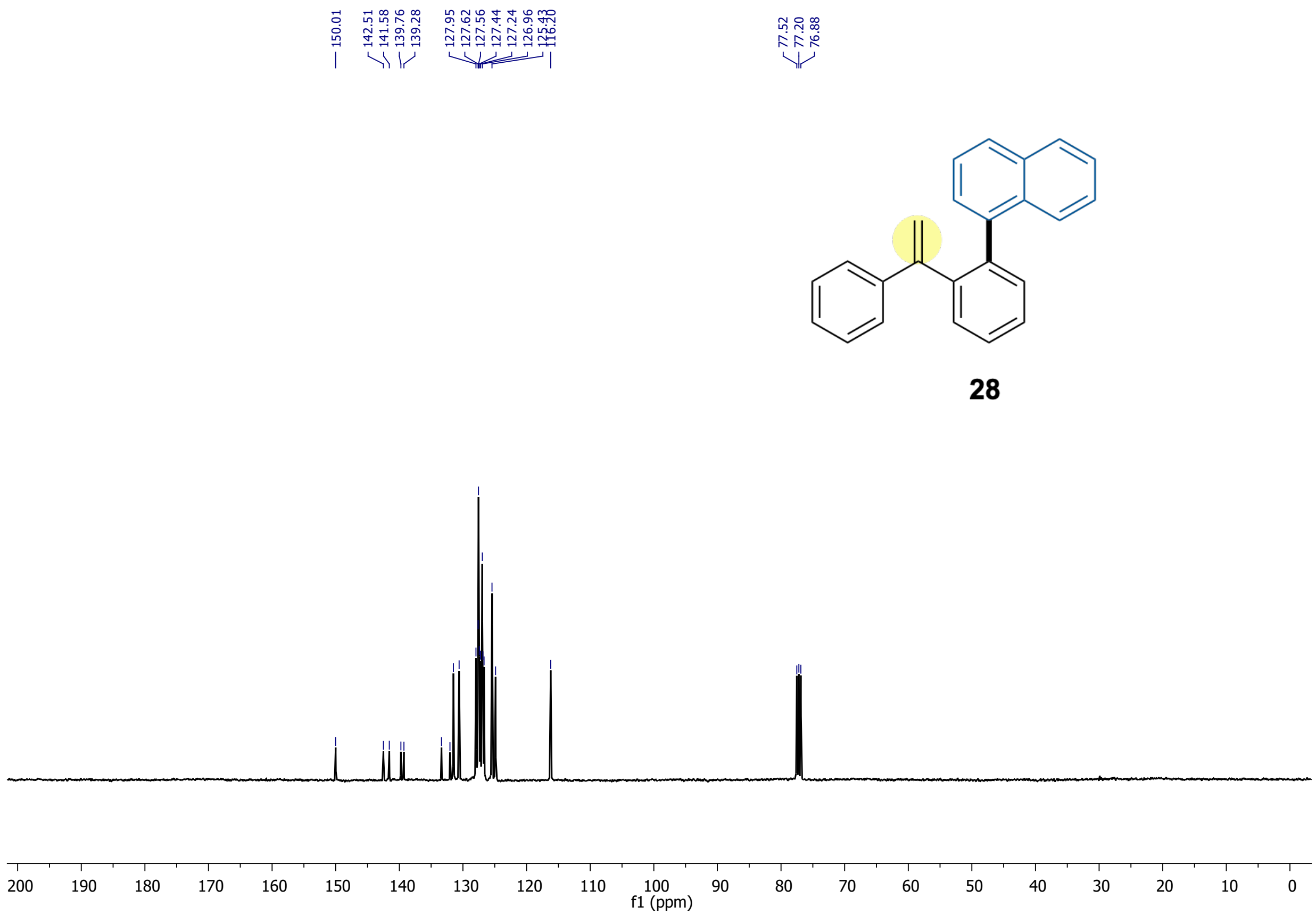
150.01
142.51
141.58
139.76
139.28
127.95
127.62
127.56
127.44
127.24
126.96
77.52
77.20
76.88
f1 (ppm)

28

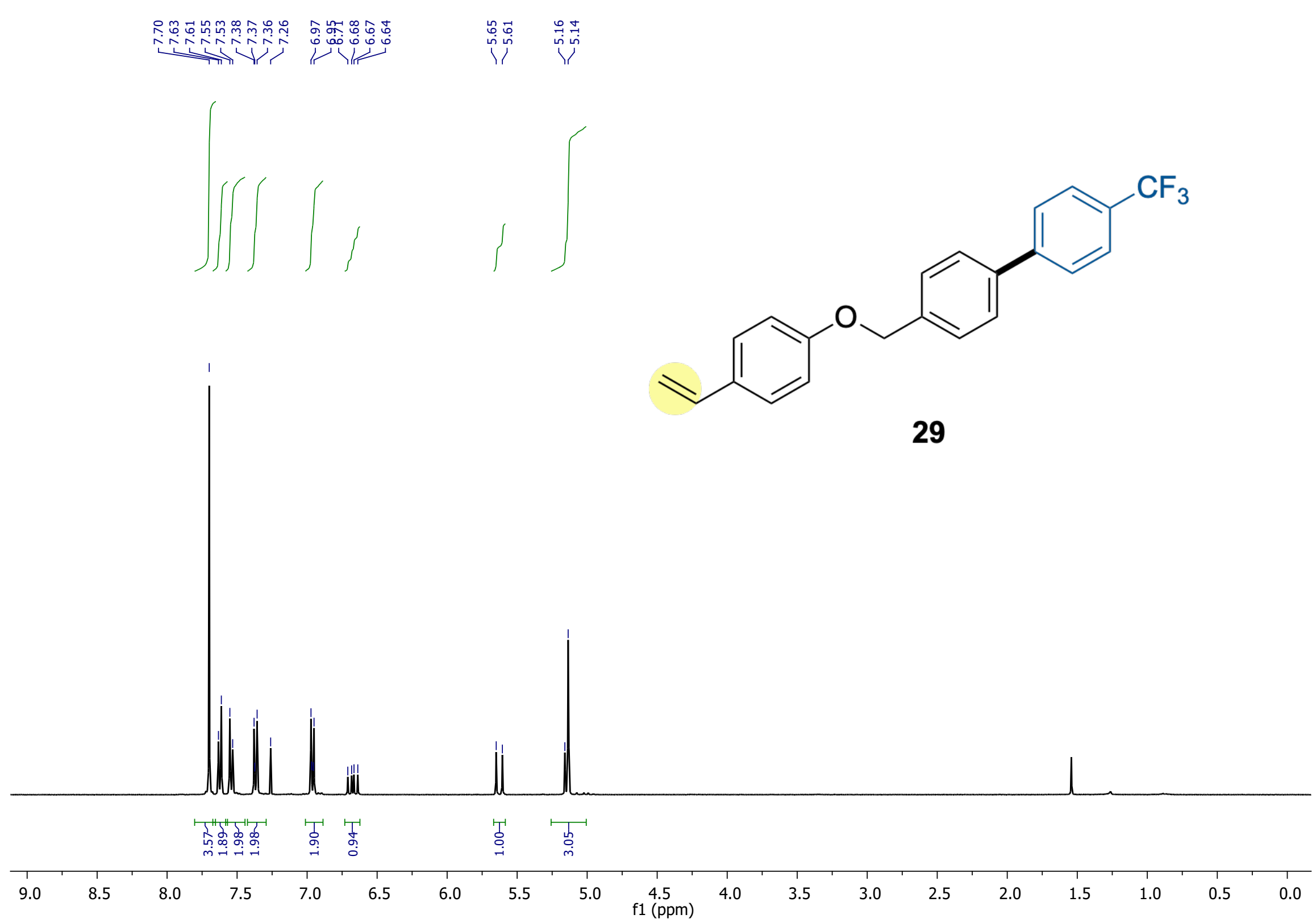
7.70
7.63
7.61
7.55
7.53
7.38
7.37
7.36
7.26
6.97
6.95
6.71
6.68
6.67
6.64
5.65
5.61
5.16
5.14
CF_3
O
f1 (ppm)

29

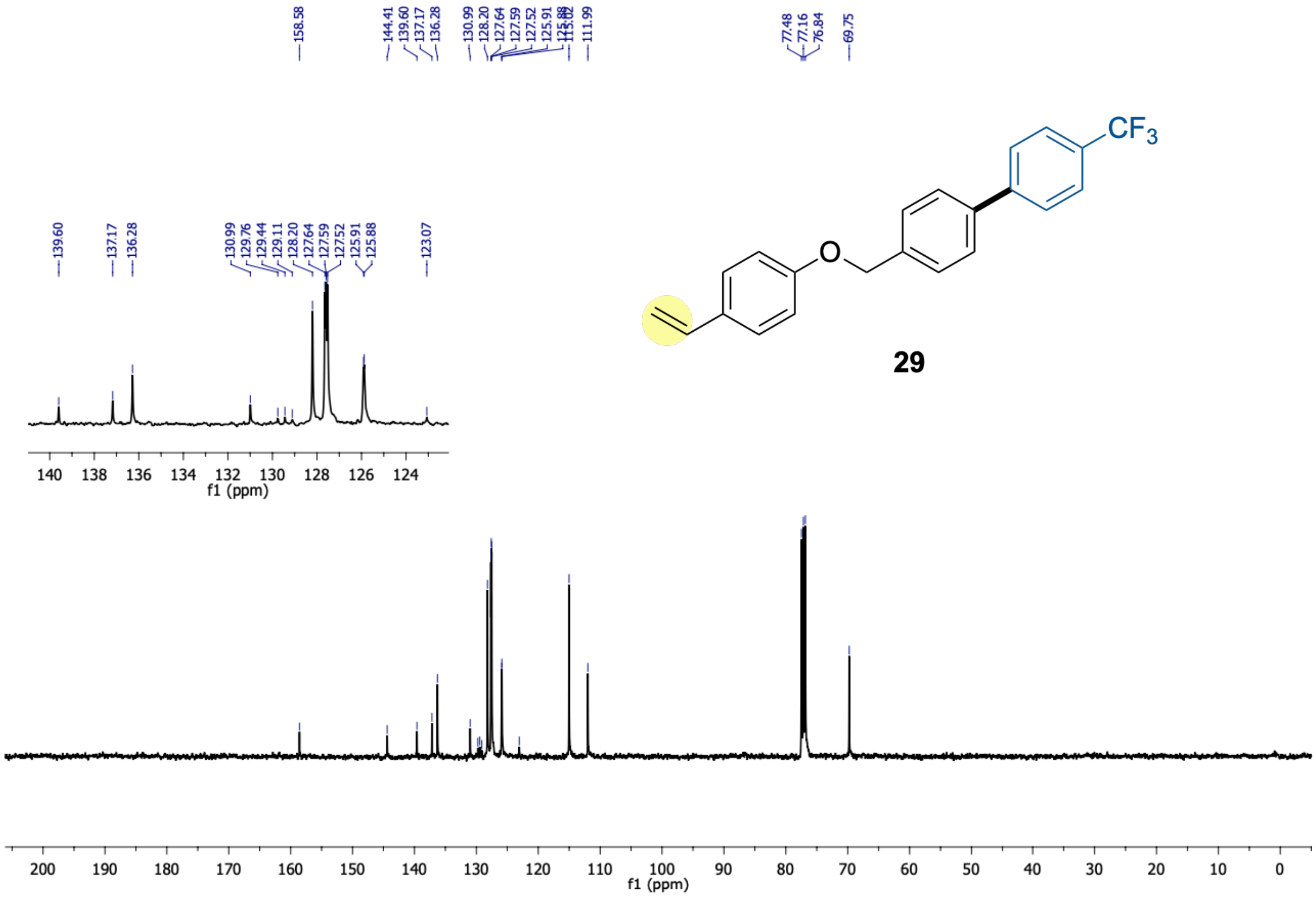
158.58
144.41
139.60
137.17
136.28
130.99
128.20
127.64
127.59
127.52
125.91
125.88
115.02
111.99
77.48
77.16
76.84
69.75
139.60
137.17
136.28
130.99
129.76
129.44
129.11
128.20
127.64
127.59
127.52
125.91
125.88
123.07
f1 (ppm)
CF$_3$
O
29
f1 (ppm)

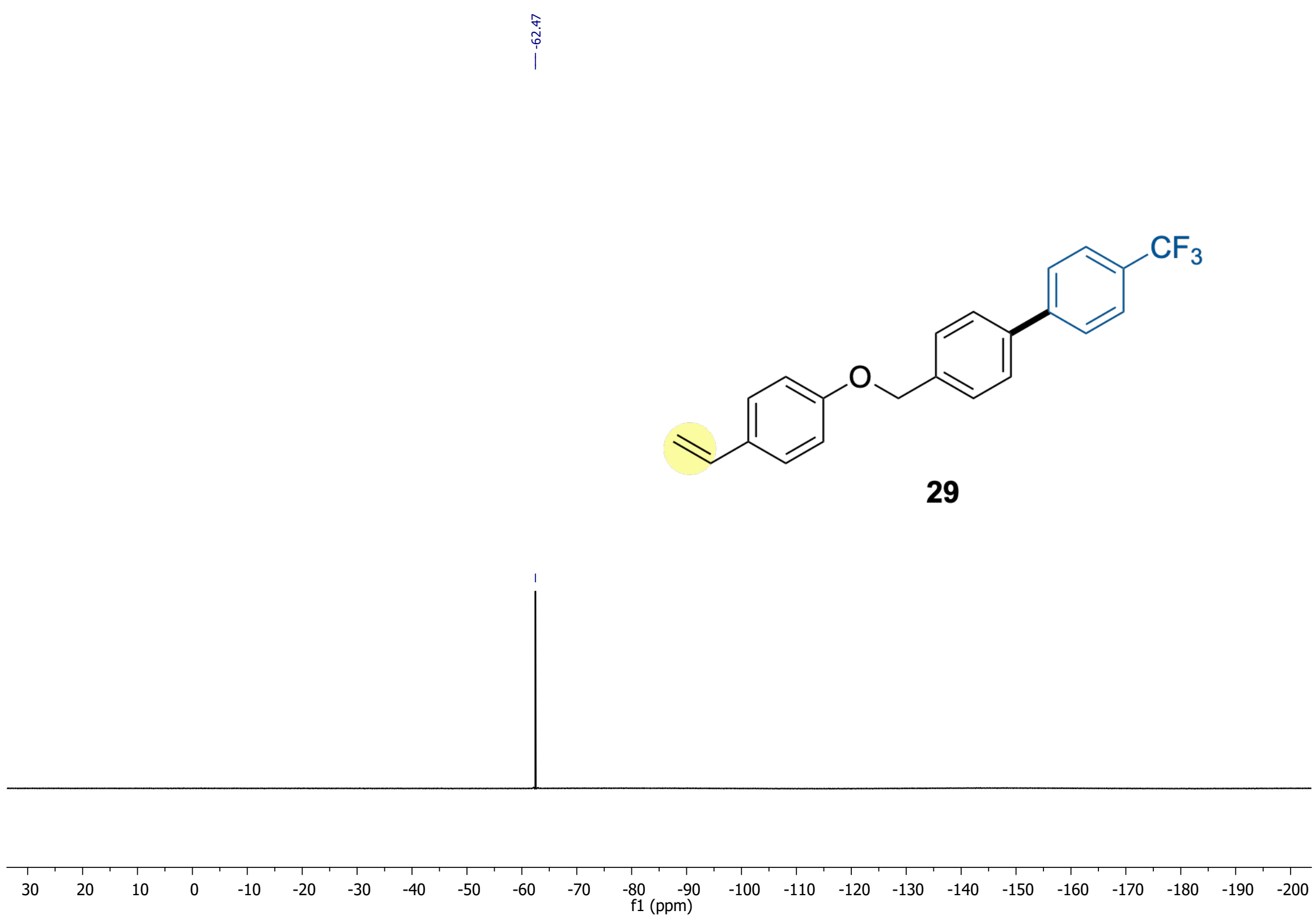
-62.47
CF$_3$
O
29
f1 (ppm)

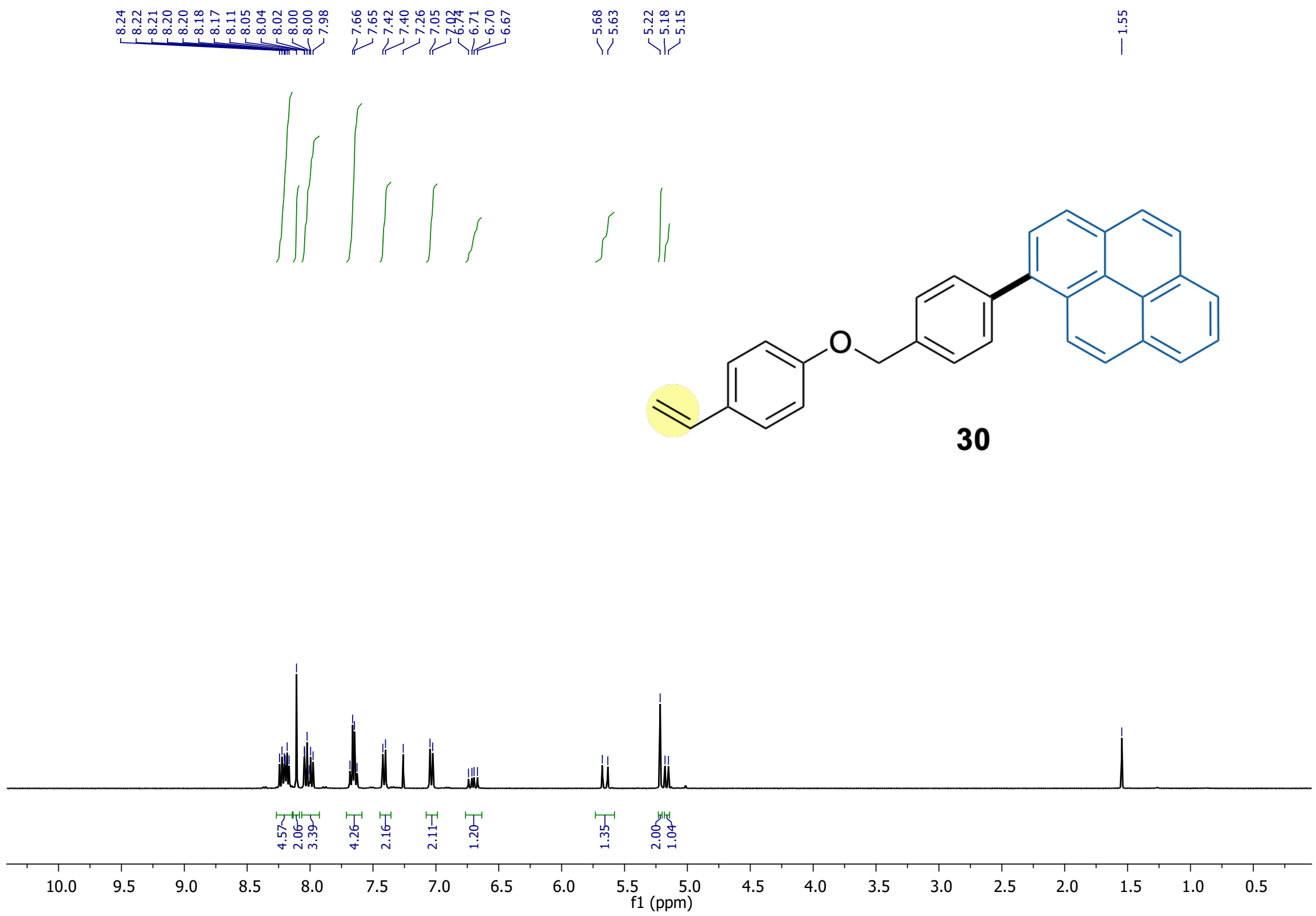
8.24 8.22 8.21 8.20 8.20 8.18 8.17 8.11 8.05 8.04 8.02 8.00 8.00 7.98
7.66 7.65 7.42 7.40 7.26 7.05 7.02 6.74 6.71 6.70 6.67
5.68 5.63
5.22 5.18 5.15
1.55
O
30
4.57 2.06 3.39 4.26 2.16 2.11 1.20 1.35 2.00 1.04
10.0 9.5 9.0 8.5 8.0 7.5 7.0 6.5 6.0 5.5 5.0 4.5 4.0 3.5 3.0 2.5 2.0 1.5 1.0 0.5
f1 (ppm)

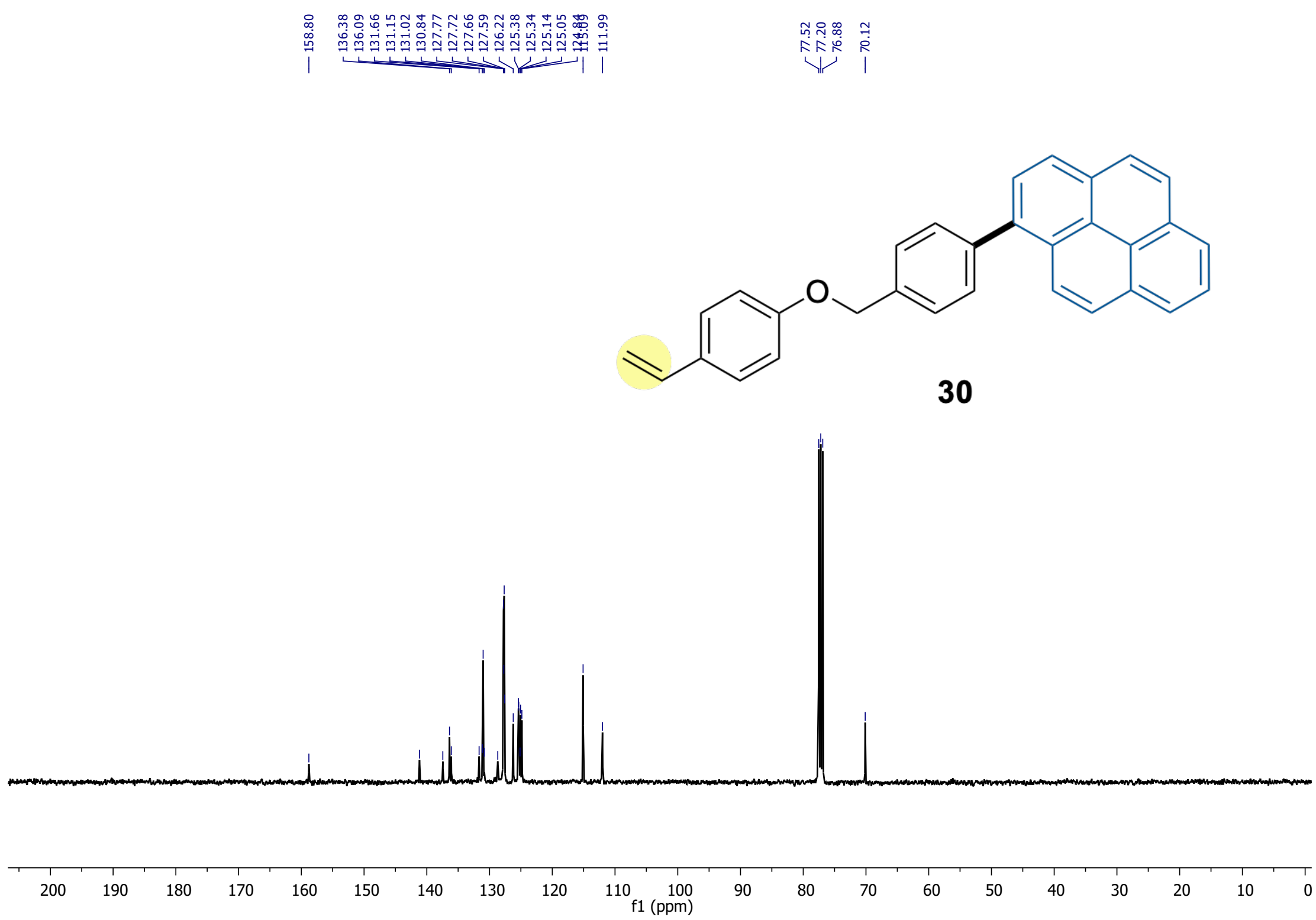
158.80
136.38 136.09 131.66 131.15 131.02 130.84 127.77 127.72 127.66 127.59 126.22 125.38 125.34 125.14 125.05
111.99
77.52 77.20 76.88
70.12
O
30
200 190 180 170 160 150 140 130 120 110 100 90 80 70 60 50 40 30 20 10 0
f1 (ppm)

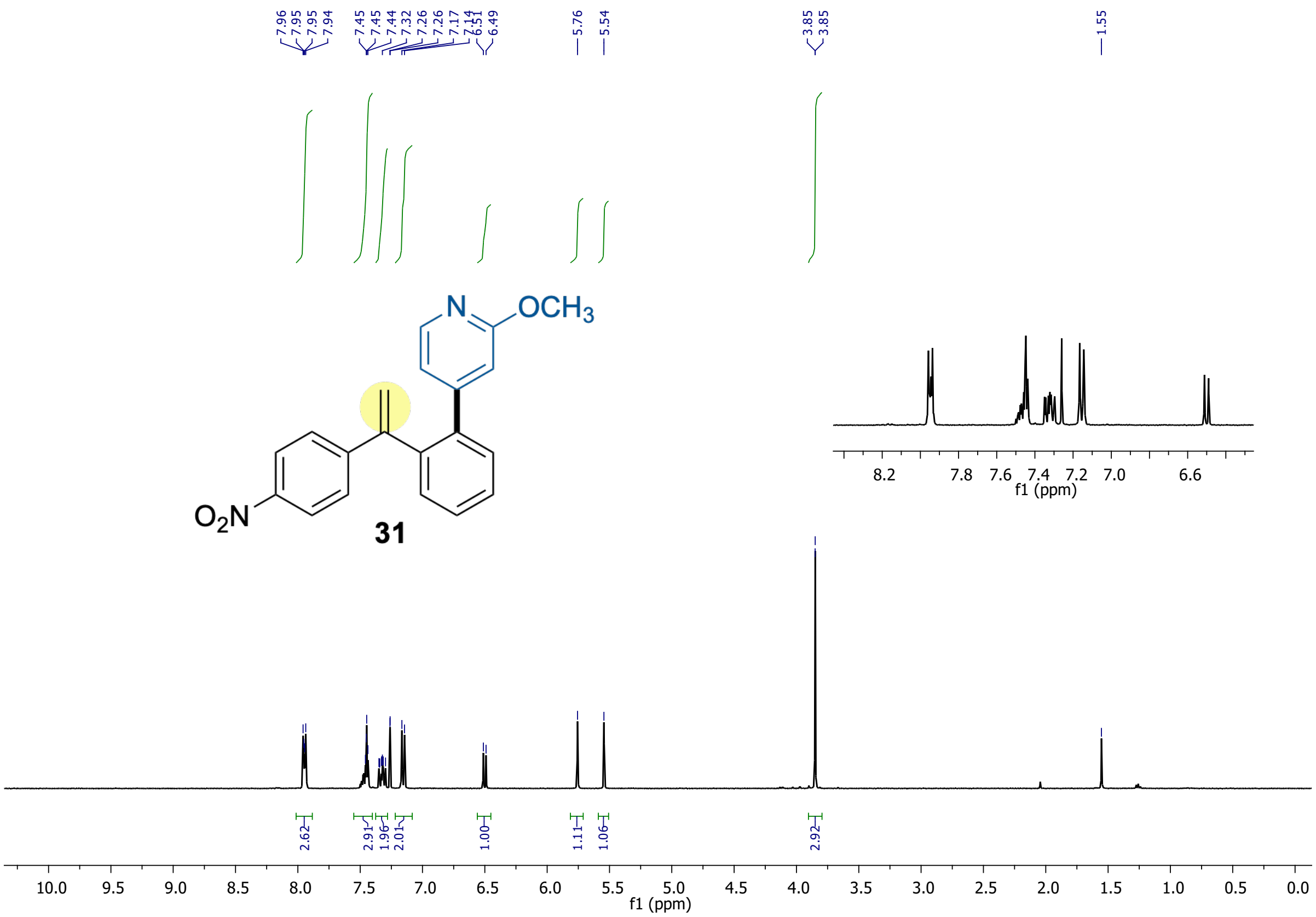
7.96
7.95
7.95
7.94
7.45
7.45
7.44
7.32
7.26
7.26
7.17
7.14
6.51
6.49
5.76
5.54
3.85
3.85
1.55
N
OCH3
O2N
31
8.2 7.8 7.6 7.4 7.2 7.0 6.6
f1 (ppm)
2.62
2.91
1.96
2.01
1.00
1.11
1.06
2.92
10.0 9.5 9.0 8.5 8.0 7.5 7.0 6.5 6.0 5.5 5.0 4.5 4.0 3.5 3.0 2.5 2.0 1.5 1.0 0.5 0.0
f1 (ppm)

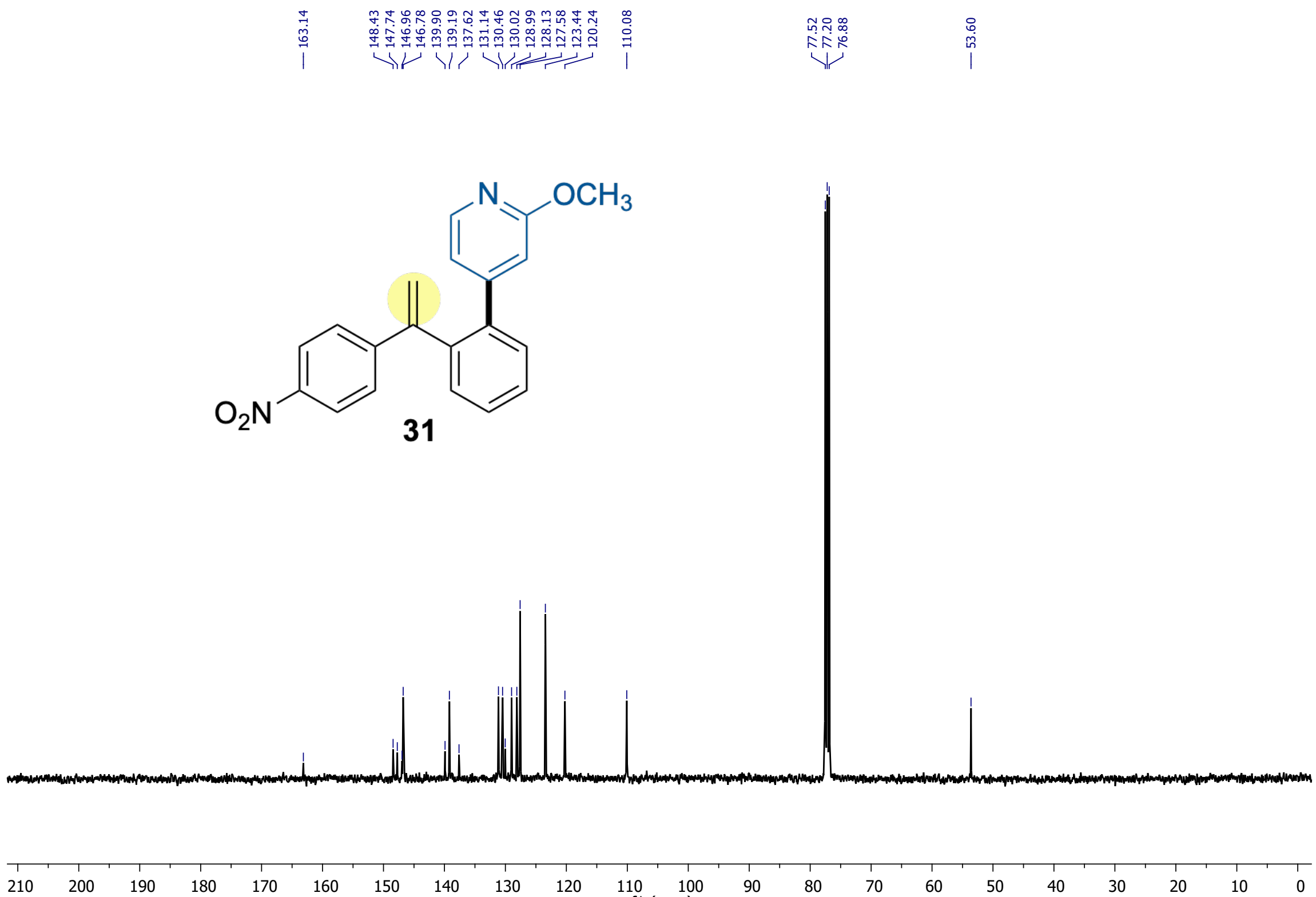
163.14
148.43
147.74
146.96
146.78
139.90
139.19
137.62
131.14
130.46
130.02
128.99
128.13
127.58
123.44
120.24
110.08
77.52
77.20
76.88
53.60
N
OCH3
O2N
31
210 200 190 180 170 160 150 140 130 120 110 100 90 80 70 60 50 40 30 20 10 0
f1 (ppm)

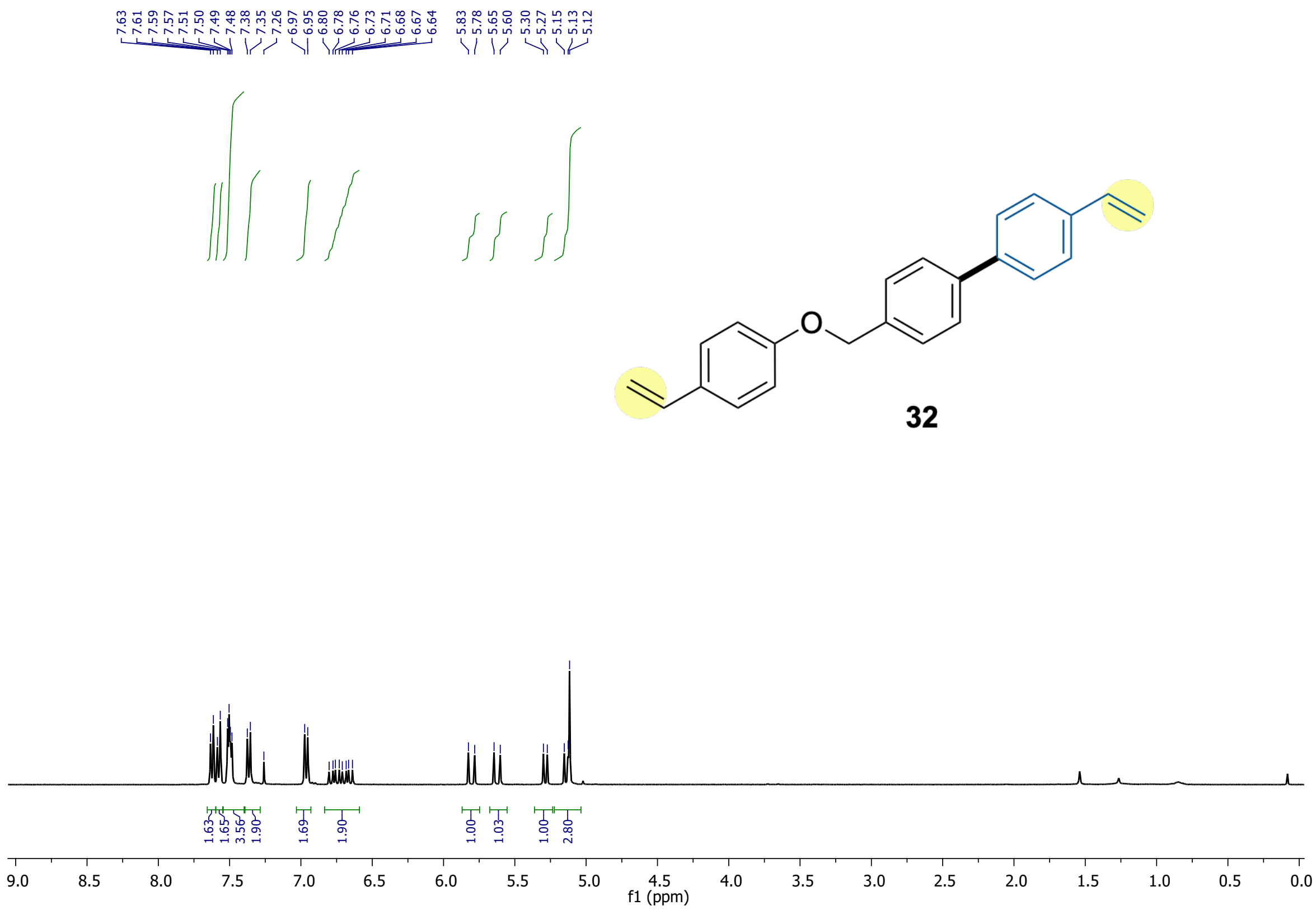

7.63 7.61 7.59 7.57 7.51 7.50 7.49 7.48 7.38 7.35 7.26 6.97 6.95 6.80 6.78 6.76 6.73 6.71 6.68 6.67 6.64 5.83 5.78 5.65 5.60 5.30 5.27 5.15 5.13 5.12
1.63 1.65 3.56 1.90 1.69 1.90 1.00 1.03 1.00 2.80
O
f1 (ppm)

32

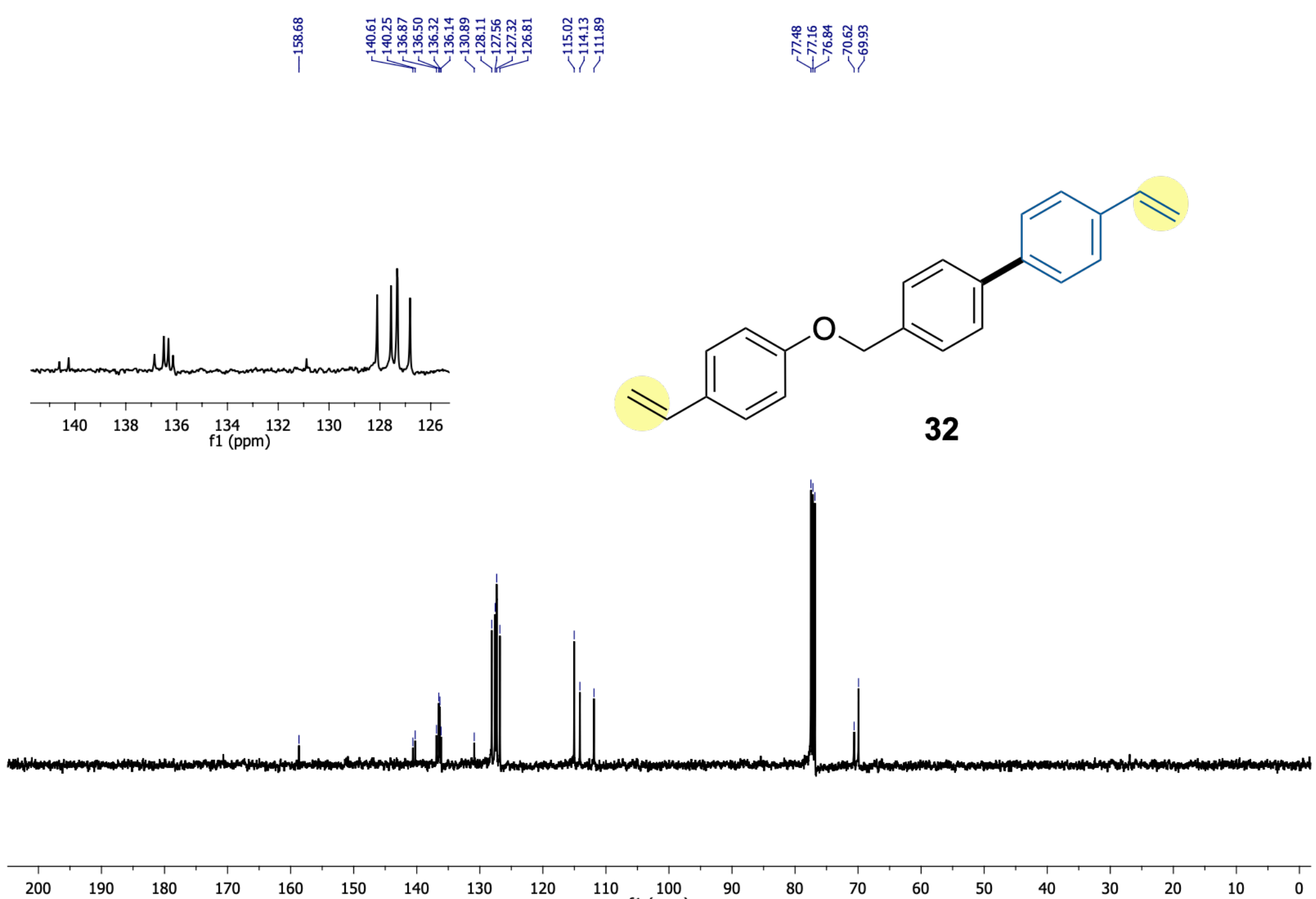

158.68 140.61 140.25 136.87 136.50 136.32 136.14 130.89 128.11 127.56 127.32 126.81 115.02 114.13 111.89 77.48 77.16 76.84 70.62 69.93
O
f1 (ppm)
f1 (ppm)

32

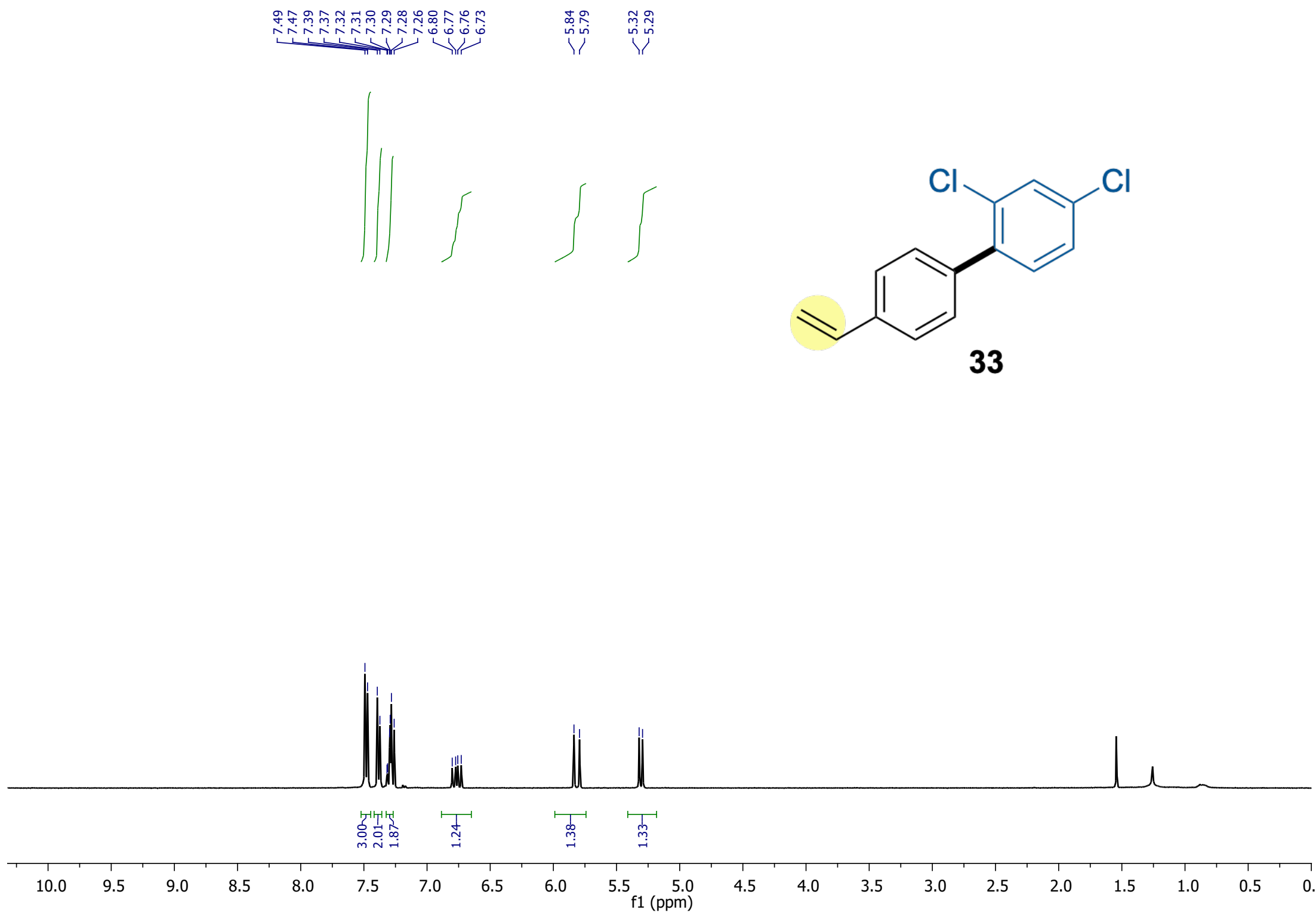
7.49
7.47
7.39
7.37
7.32
7.31
7.30
7.29
7.28
7.26
6.80
6.77
6.76
6.73
5.84
5.79
5.32
5.29
3.00
2.01
1.87
1.24
1.38
1.33
f1 (ppm)
Cl
Cl

33

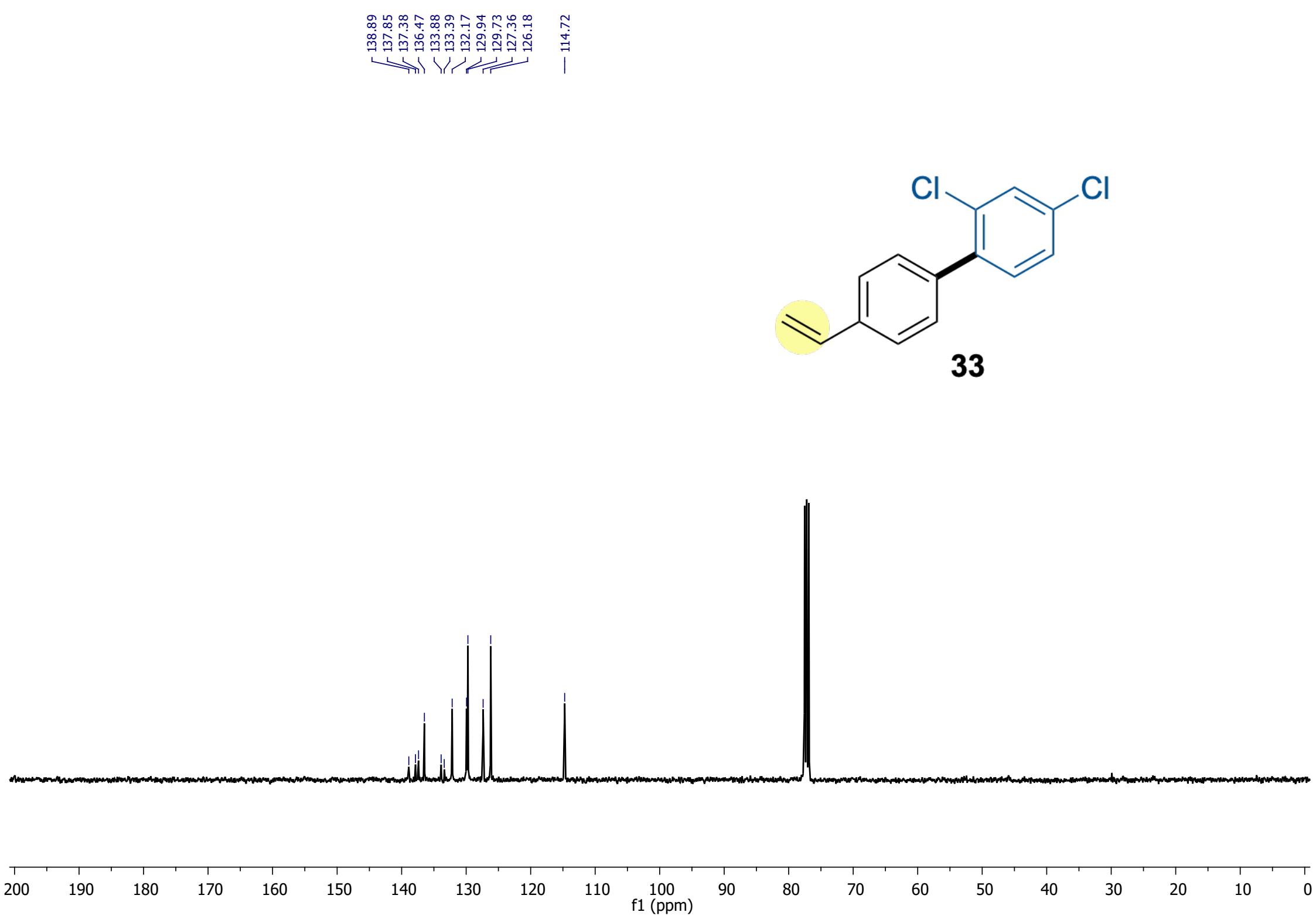
138.89
137.85
137.38
136.47
133.88
133.39
132.17
129.94
129.73
127.36
126.18
114.72
f1 (ppm)
Cl
Cl

33

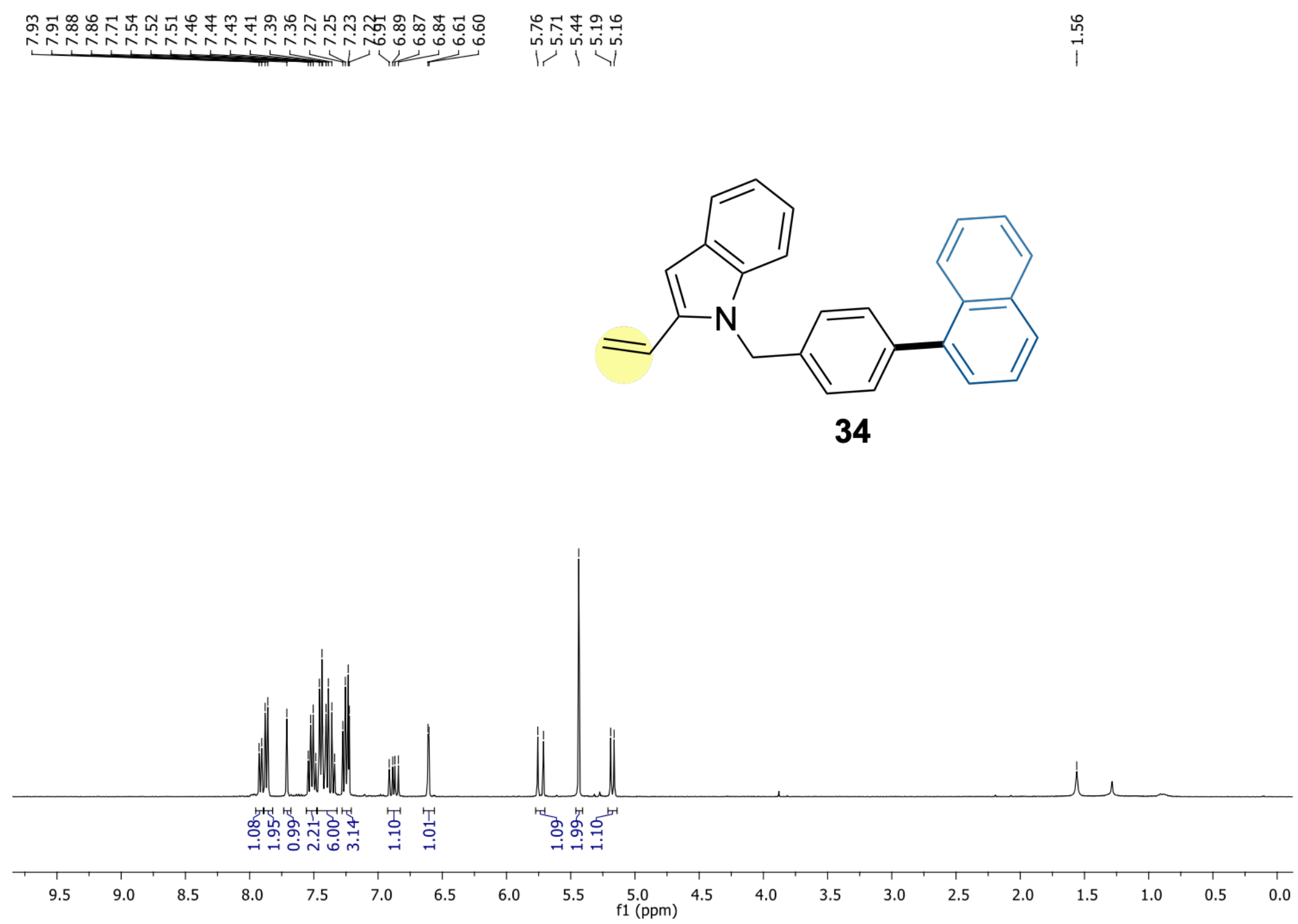
7.93
7.91
7.88
7.86
7.71
7.54
7.52
7.51
7.46
7.44
7.43
7.41
7.39
7.36
7.27
7.25
7.23
7.22
6.91
6.89
6.87
6.84
6.61
6.60
5.76
5.71
5.44
5.19
5.16
1.56
N
34
1.08
1.95
0.99
2.21
6.00
3.14
1.10
1.01
1.09
1.99
1.10
9.5 9.0 8.5 8.0 7.5 7.0 6.5 6.0 5.5 5.0 4.5 4.0 3.5 3.0 2.5 2.0 1.5 1.0 0.5 0.0
f1 (ppm)

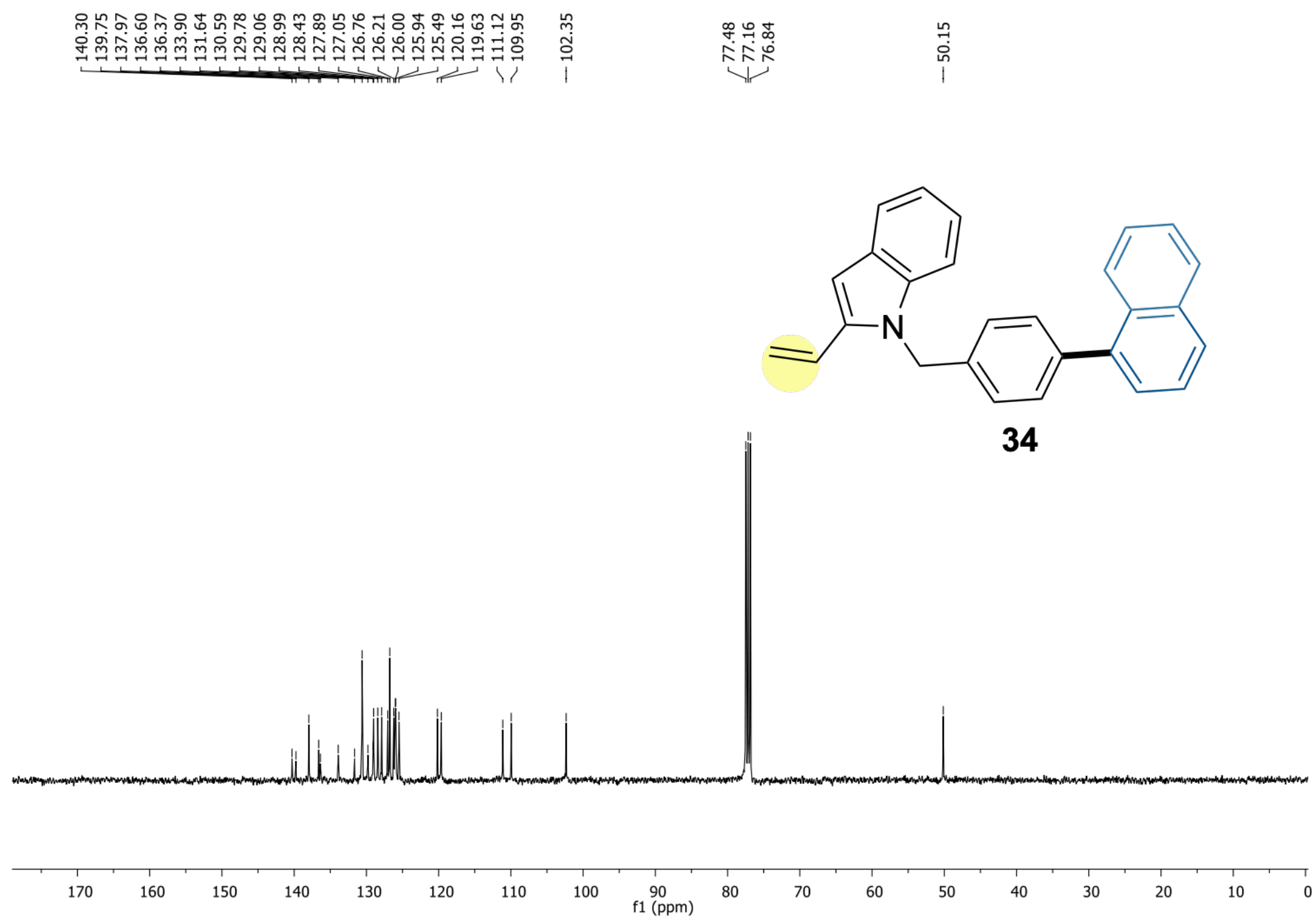
140.30
139.75
137.97
136.60
136.37
133.90
131.64
130.59
129.78
129.06
128.99
128.43
127.89
127.05
126.76
126.21
126.00
125.94
125.49
120.16
119.63
111.12
109.95
102.35
77.48
77.16
76.84
50.15
N
34
170 160 150 140 130 120 110 100 90 80 70 60 50 40 30 20 10 0
f1 (ppm)

16. HRMS ANALYSIS

2,4-Dimethoxy-4'-vinyl-1,1'-biphenyl (4)

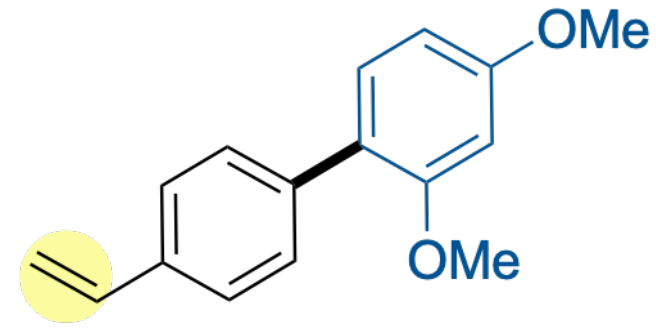

MS Zoomed Spectrum

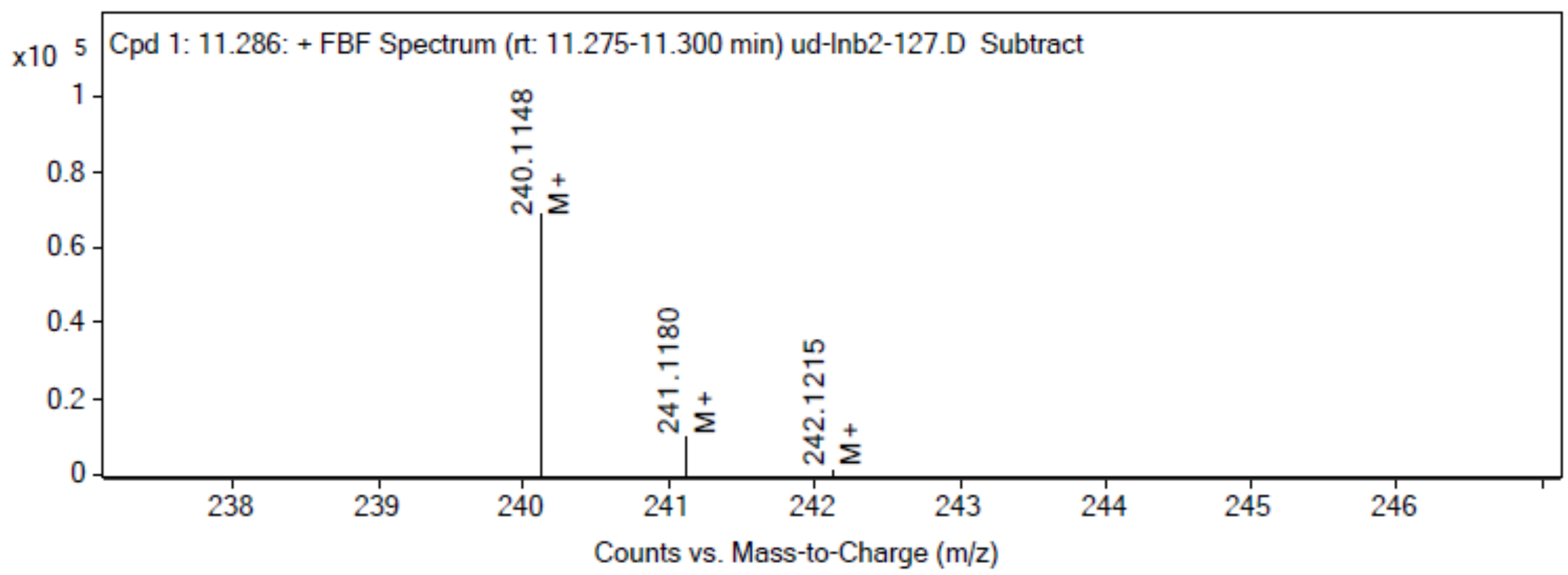

MS Spectrum Peak List

m/z	z	Abund	Ion
240.1148	1	69154.35	M+
241.118	1	9618.23	M+
242.1215	1	888.91	M+

MS Spectrum

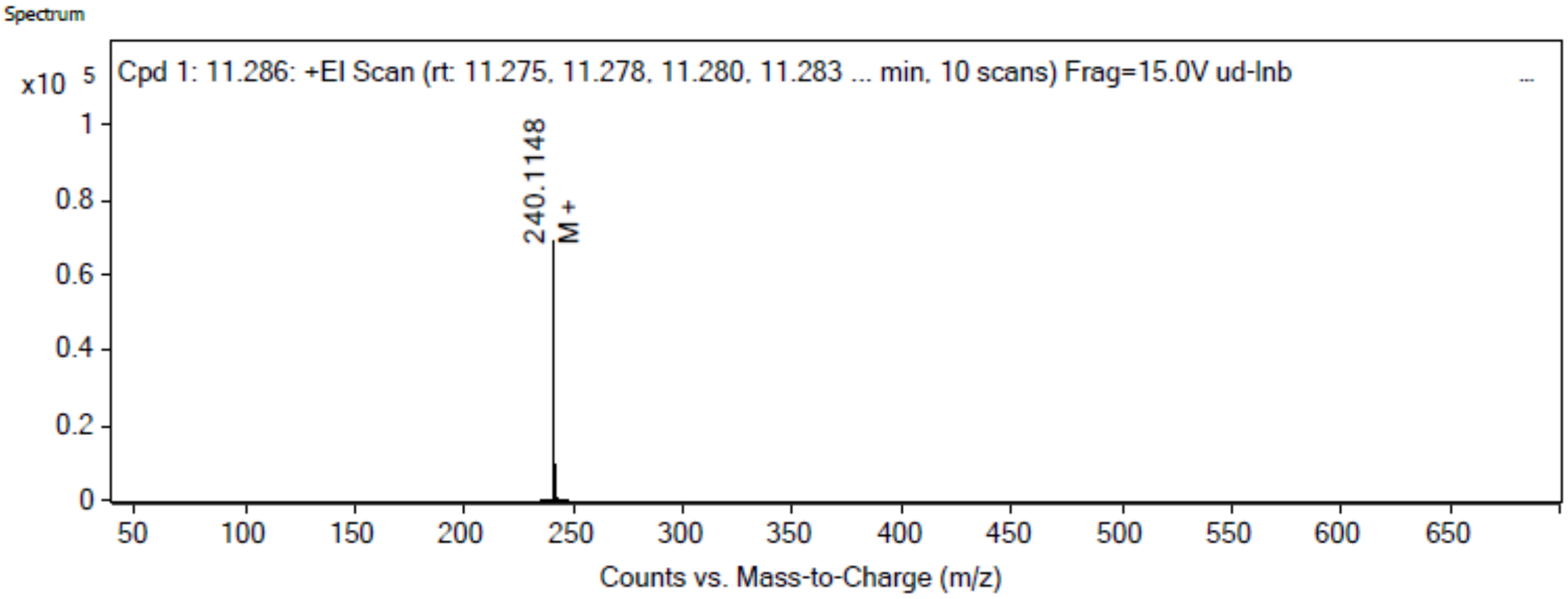

MS Zoomed Spectrum

4-(4-Fluoro-2-methylphenyl)-2-vinylthiophene (5)

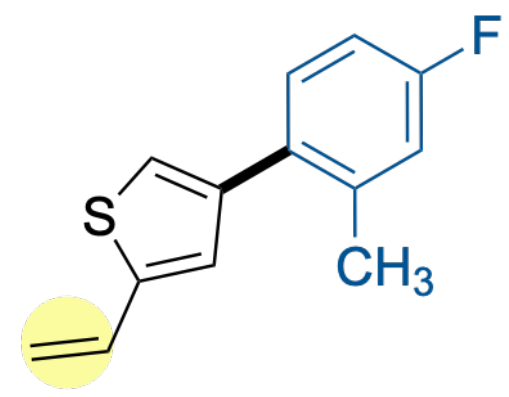

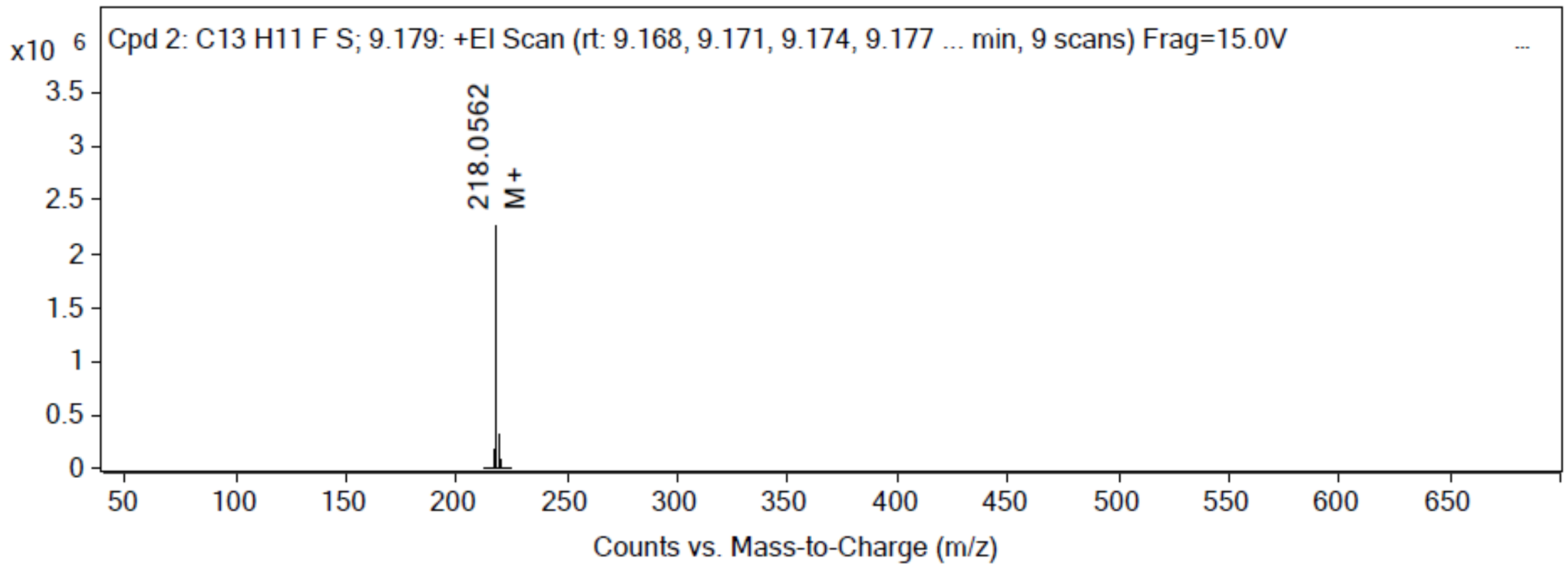

MS Zoomed Spectrum

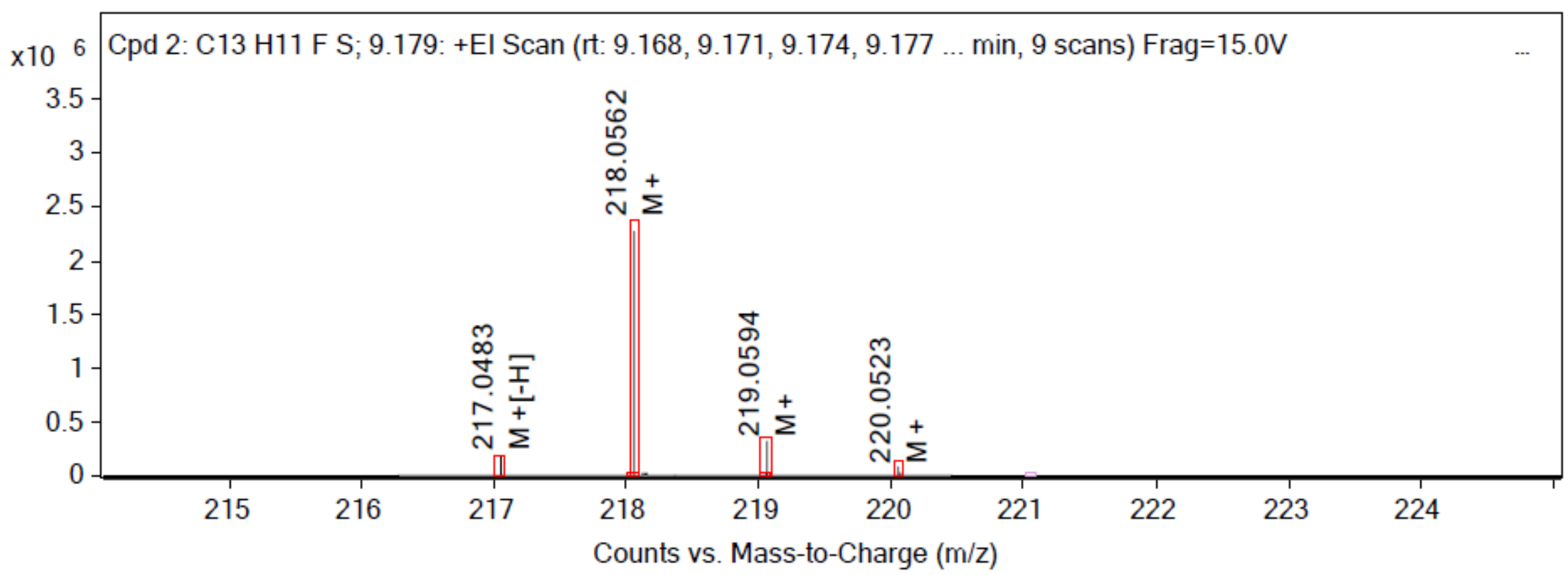

MS Spectrum Peak List

m/z	Calc *m/z*	Diff(ppm)	z	Abund	Ion
217.0483	217.0482	-0.61	1	182363.58	M+[-H]
218.0481	218.0513	15.07	1	3577.11	M+[-H]
218.0562				2361450.8	

4-Chloro-4'-((4-vinylphenoxy)methyl)-1,1'-biphenyl (7)

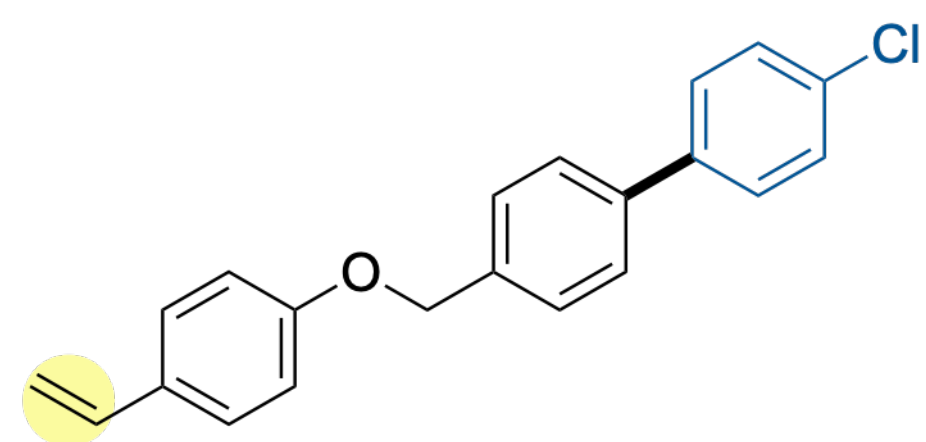

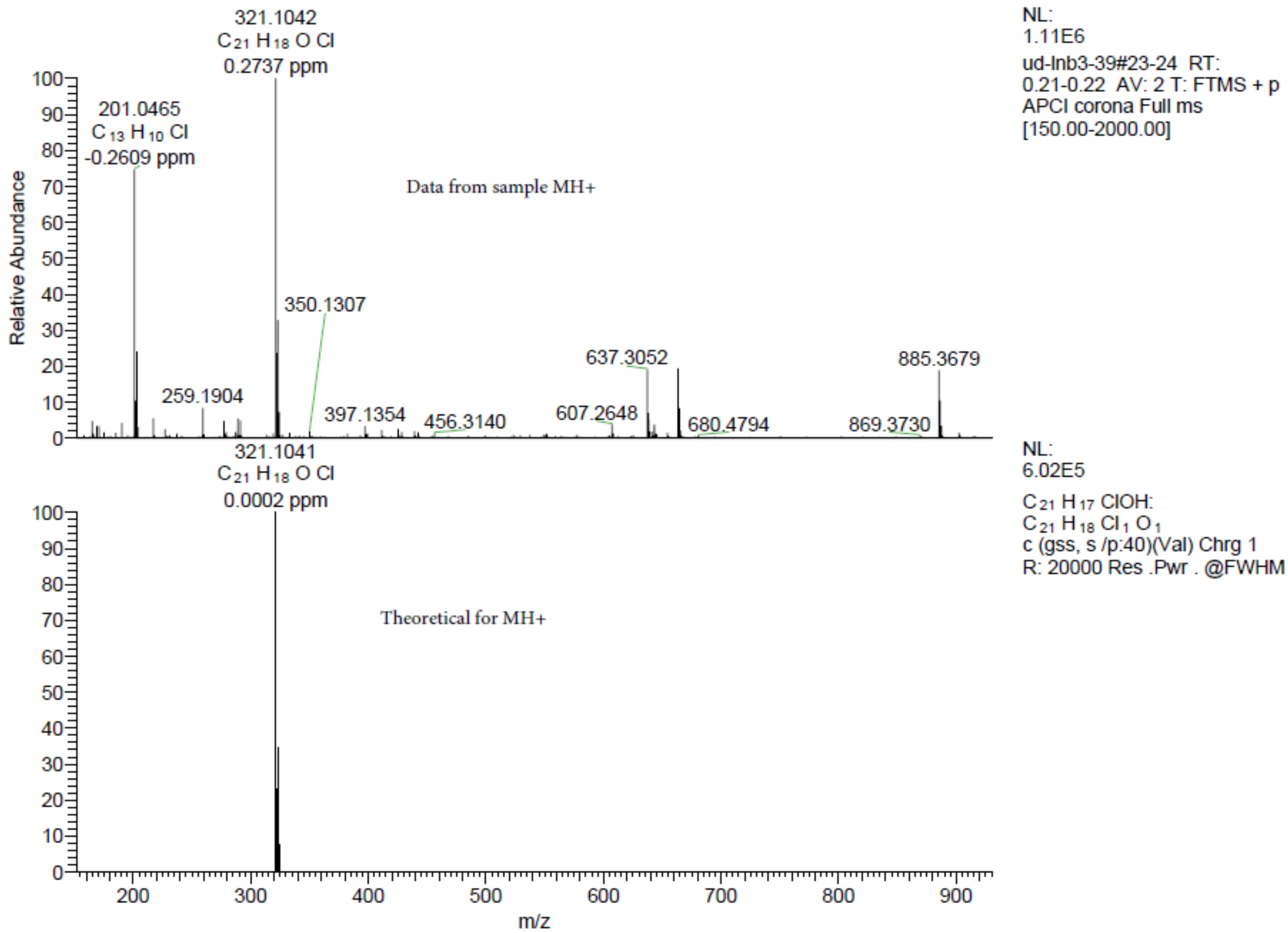

2-(4'-Vinyl-[1,1'-biphenyl]-2-yl)thiophene (8)

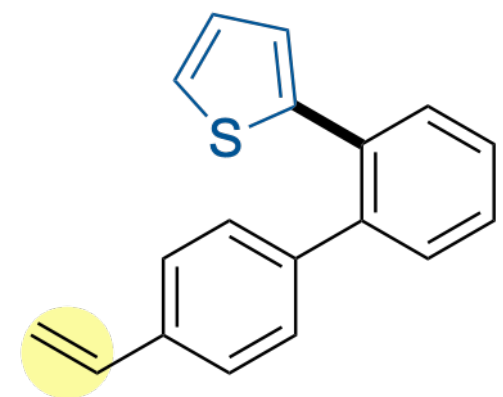

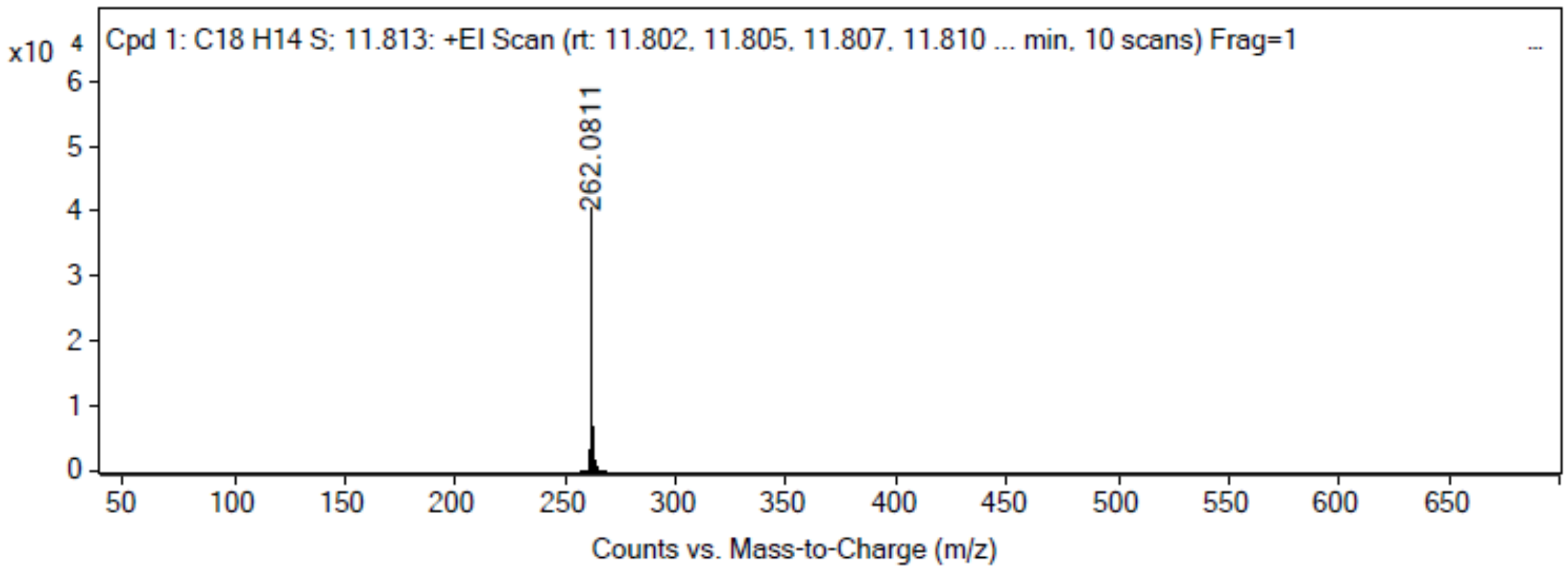

MS Zoomed Spectrum

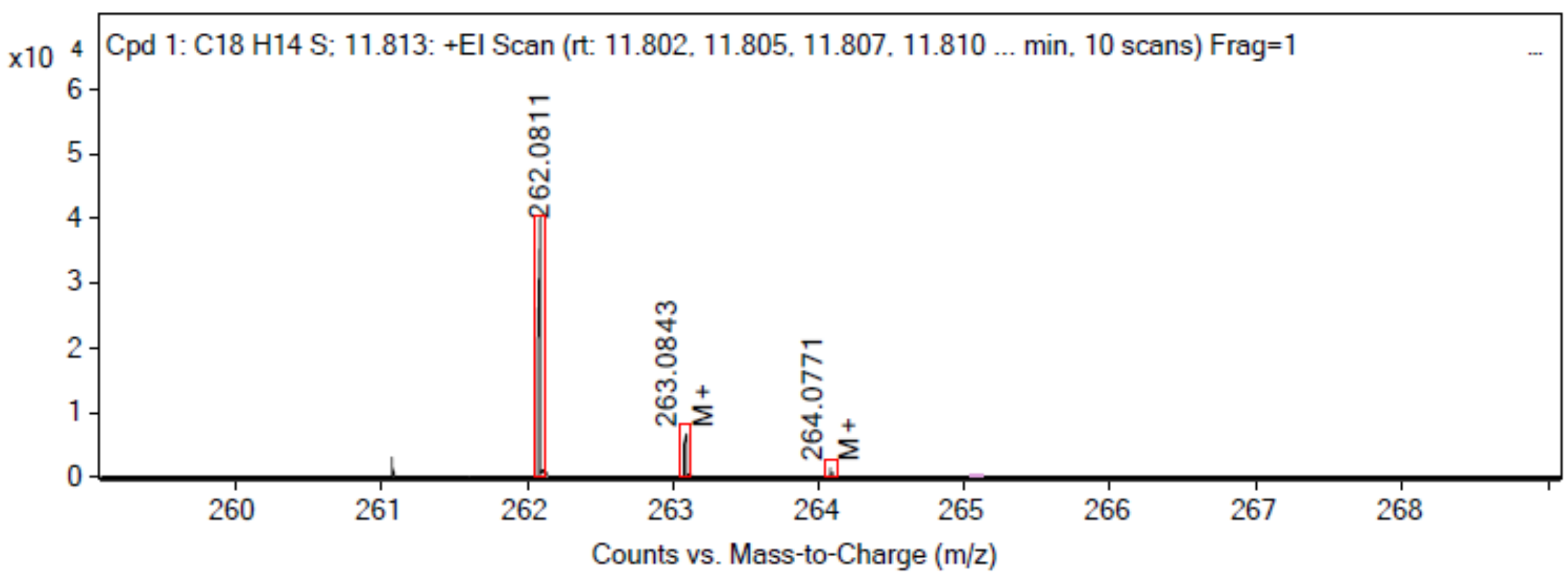

MS Spectrum Peak List

m/z	Calc *m/z*	Diff(ppm)	z	Abund	Ion
262.0811	262.0811	-0.25	1	40530.05	M+
262.0811				40546.34	
263.0843	263.0843	-0.02	1	6739.65	M+
264.0771	264.0801	11.42	1	1400.08	M+

2-Chloro-3-(4-vinylphenyl)pyridine (9)

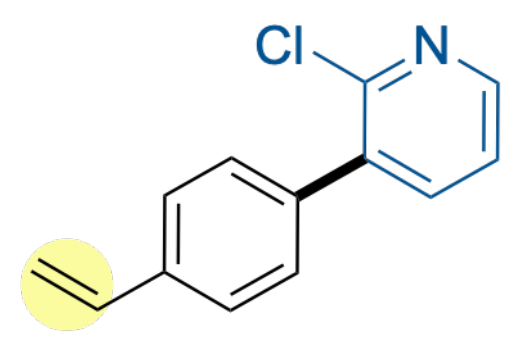

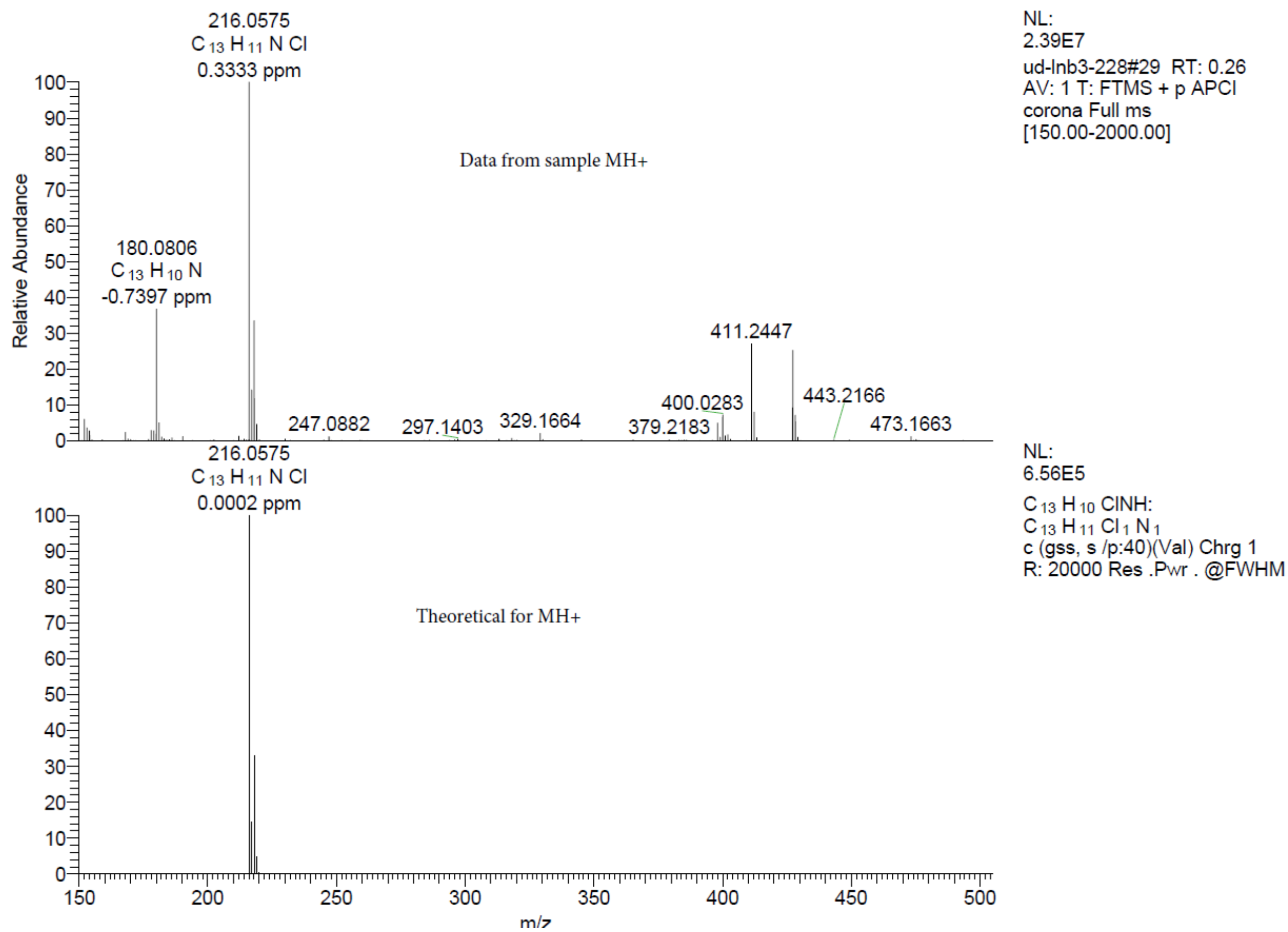

2-Chloro-4-fluoro-4''-vinyl-1,1':2',1''-terphenyl (10)

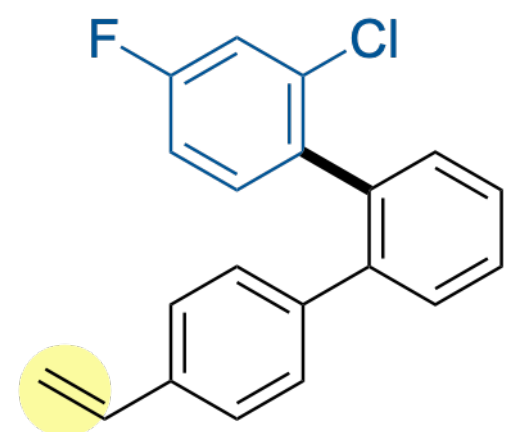

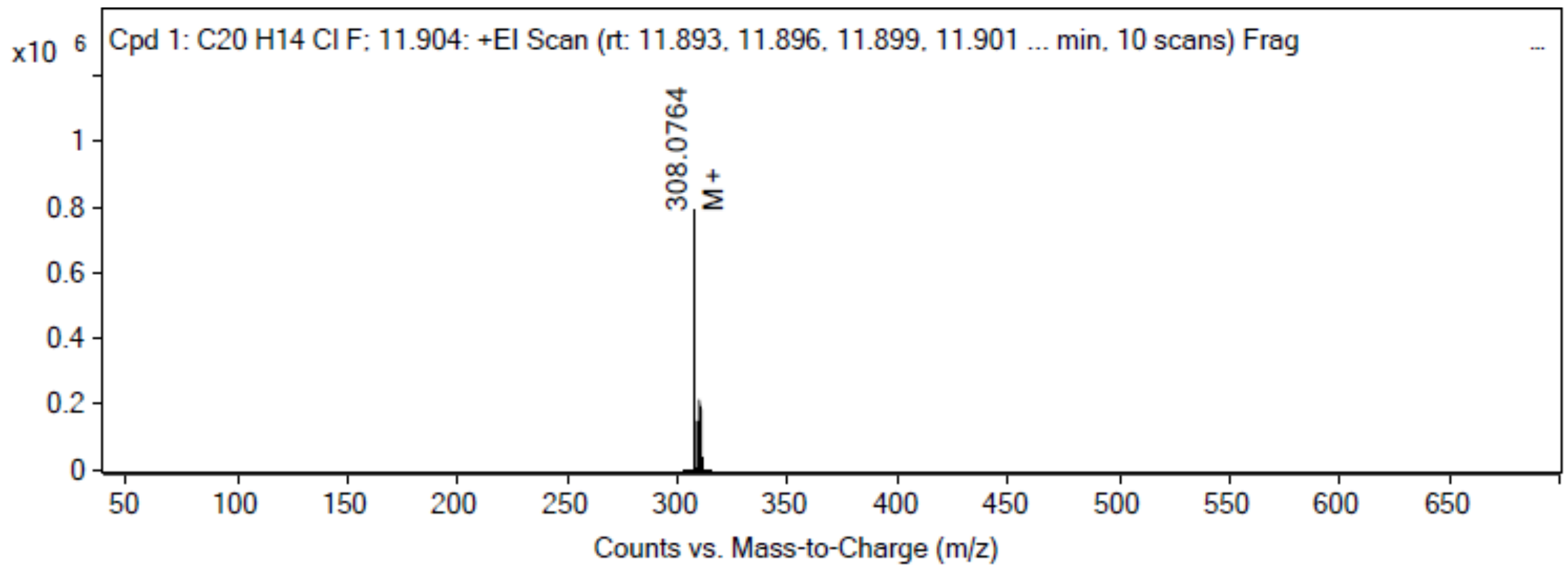

MS Zoomed Spectrum

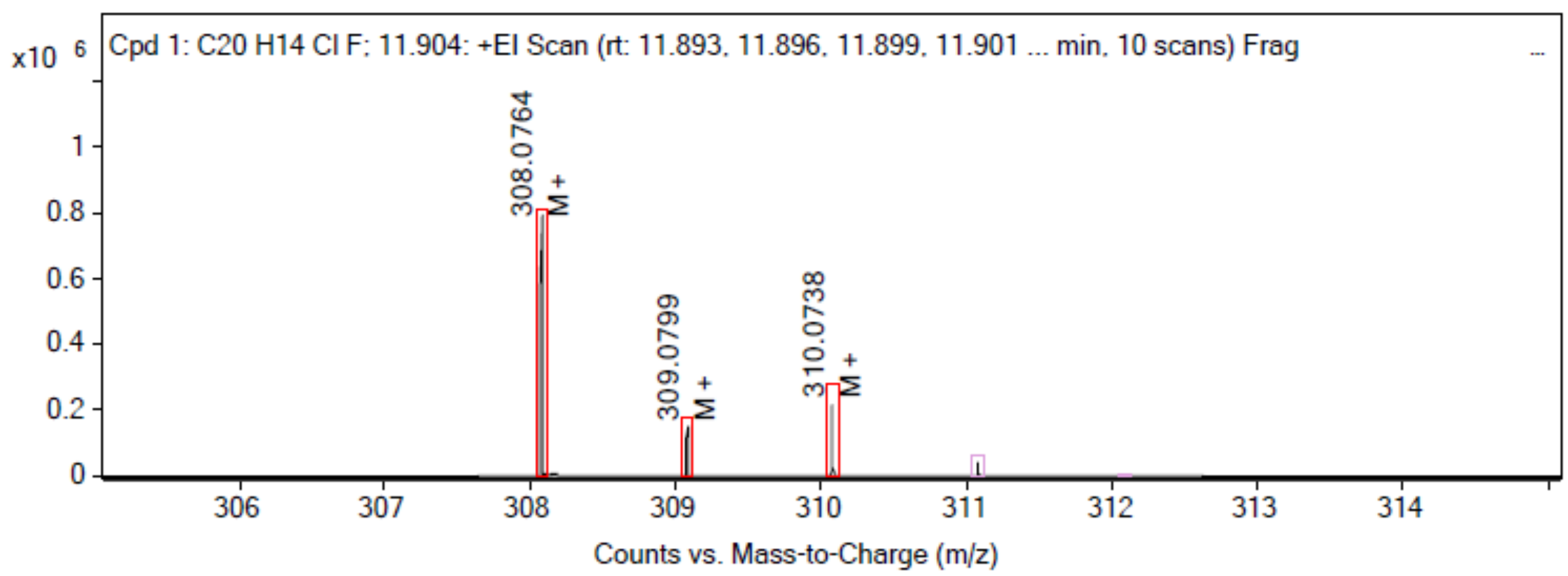

MS Spectrum Peak List

m/z	Calc *m/z*	Diff(ppm)	z	Abund	Ion
308.0764	308.0763	-0.46		810969.08	
308.0764	308.0763	-0.48	1	811298.63	M+
309.0799	309.0796	-0.82	1	152508.23	M+
310.0738	310.0739	0.4	1	221588.91	M+

Compound Label	RT	Algorithm
Cpd 2: 11.904	11.904	Find by Integration

Compound Chromatograms

2-(4-Vinylphenyl)benzofuran (11)

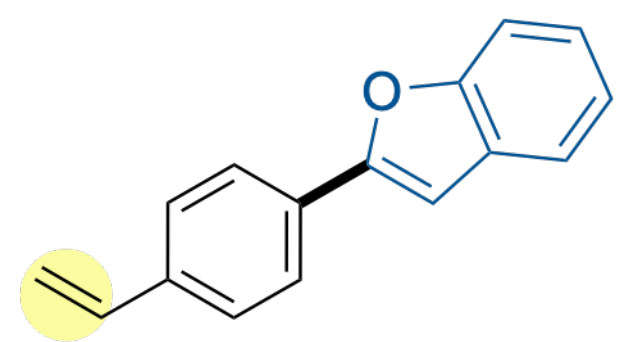

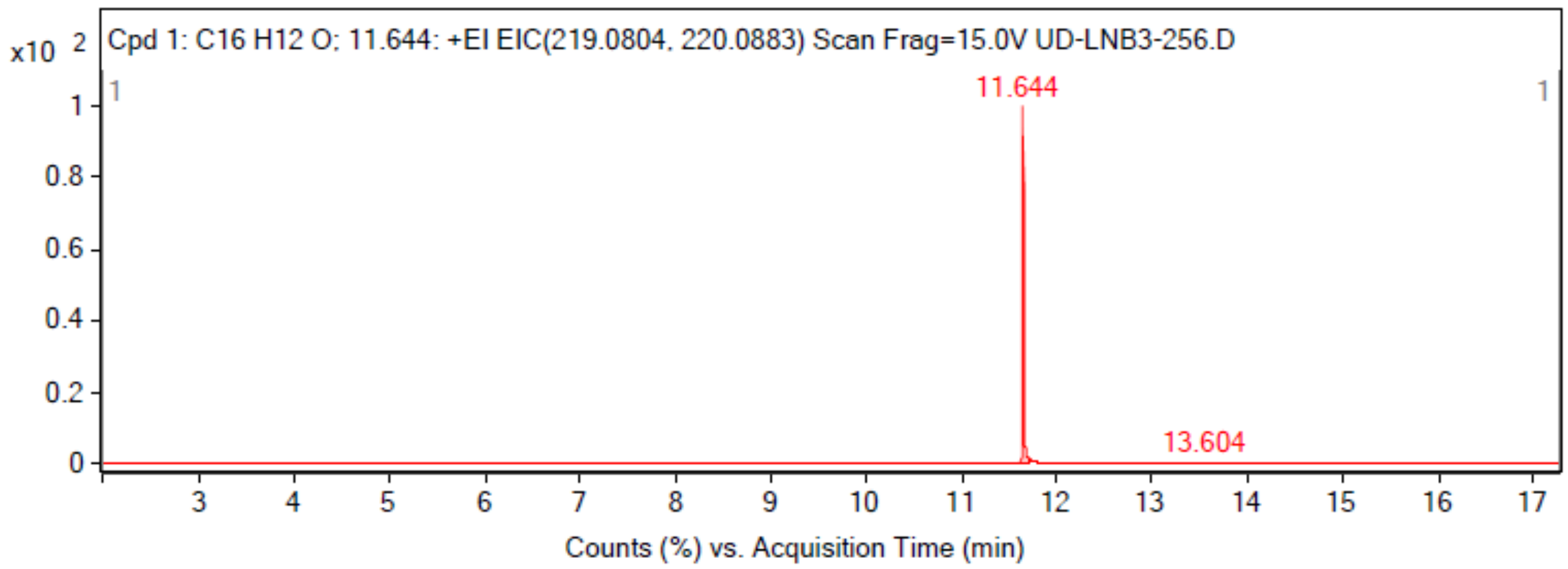

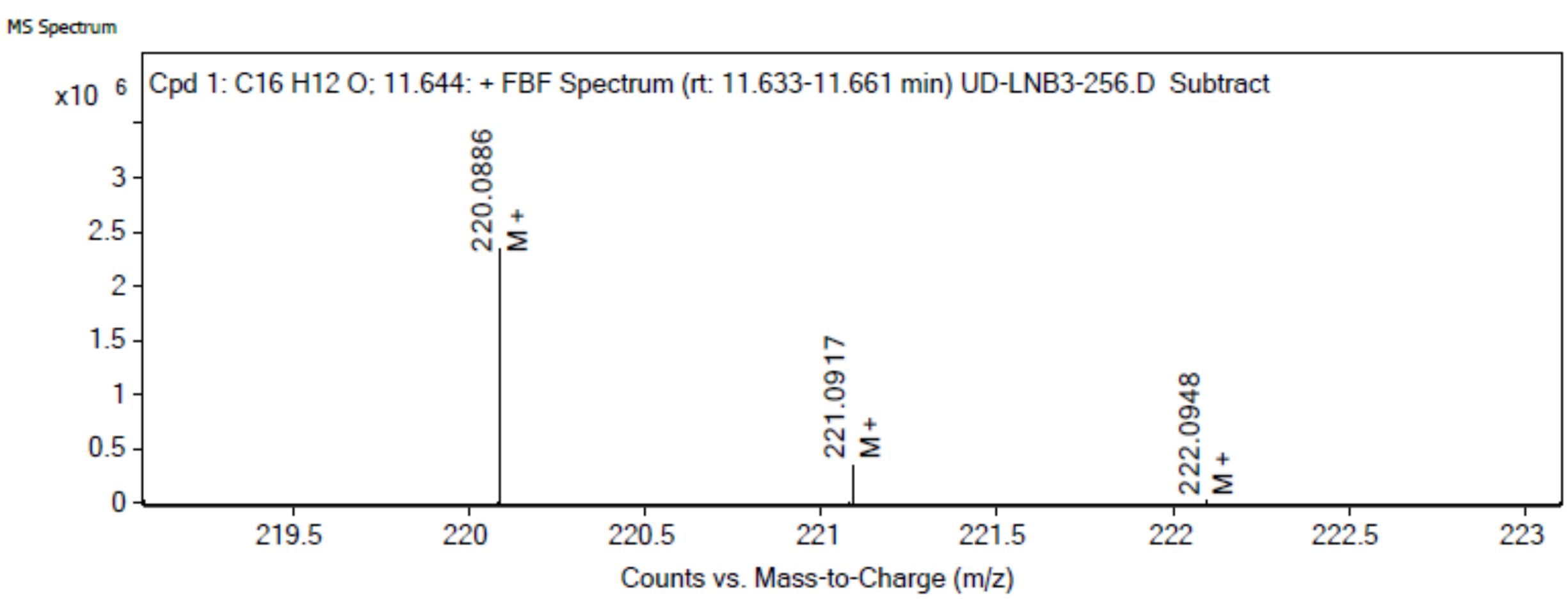

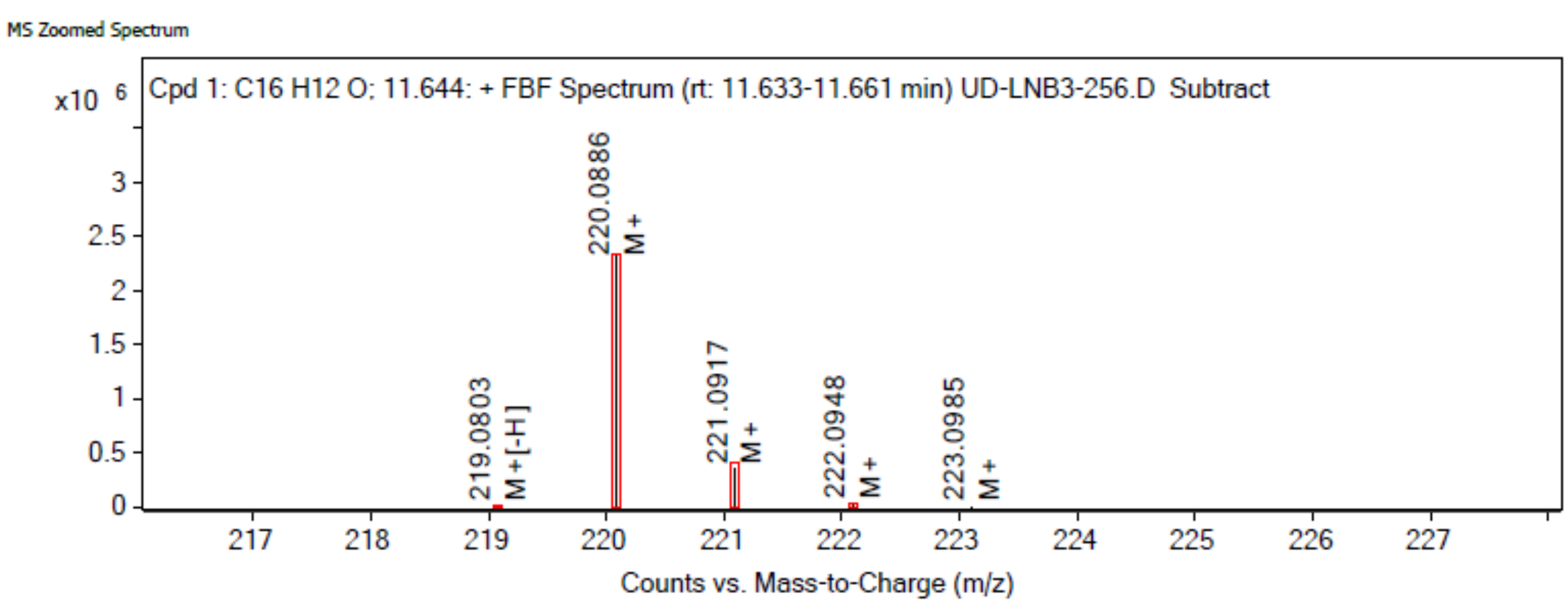

4-(2-fluorophenyl)-2-vinylthiophene (12)

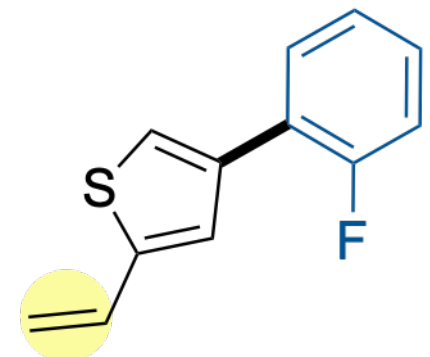

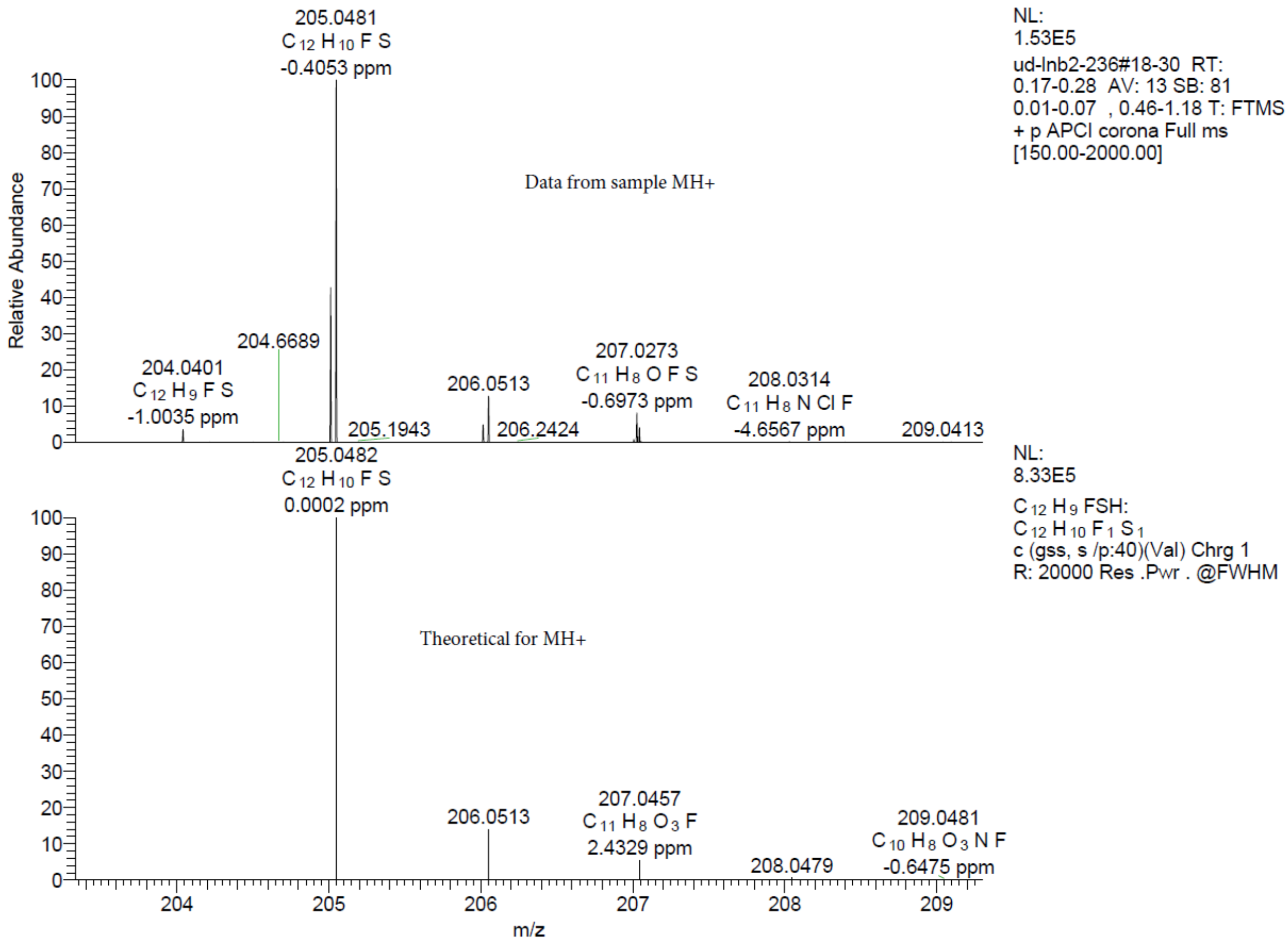

3-(4-vinylphenyl)furan (13)

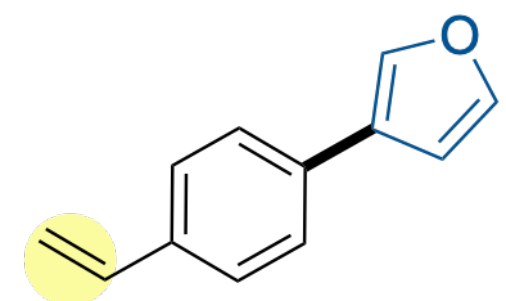

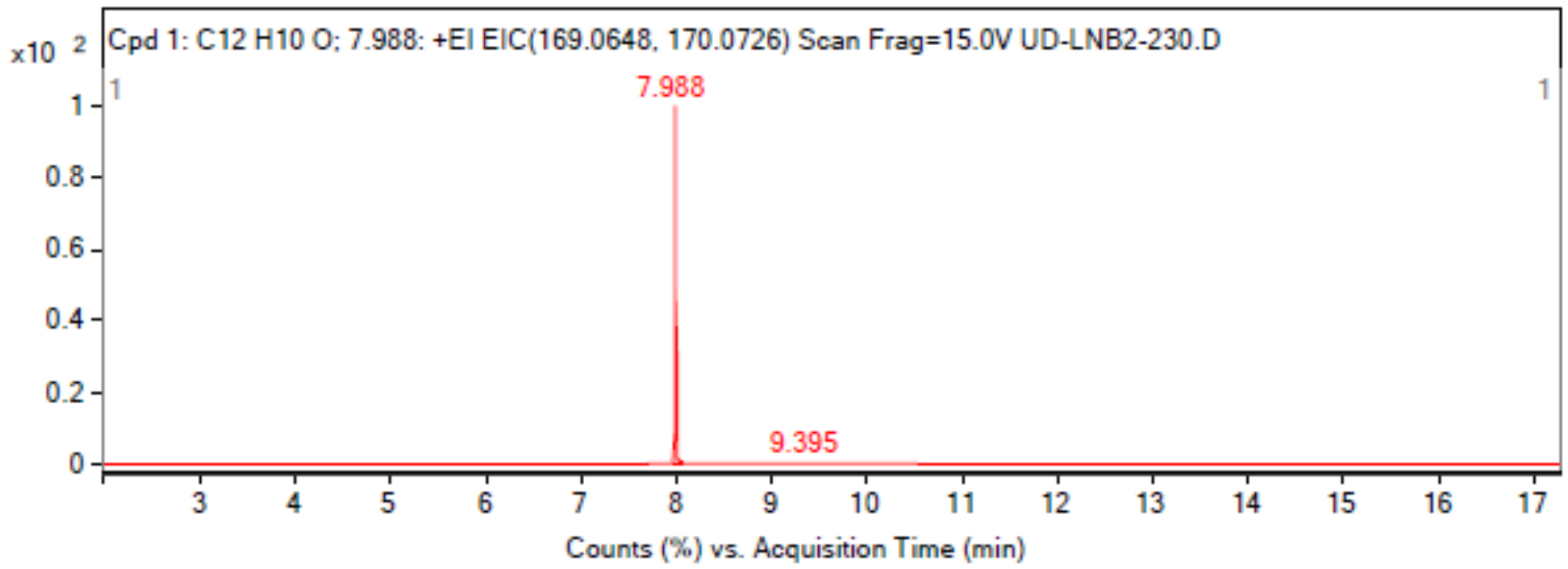

MS Spectrum

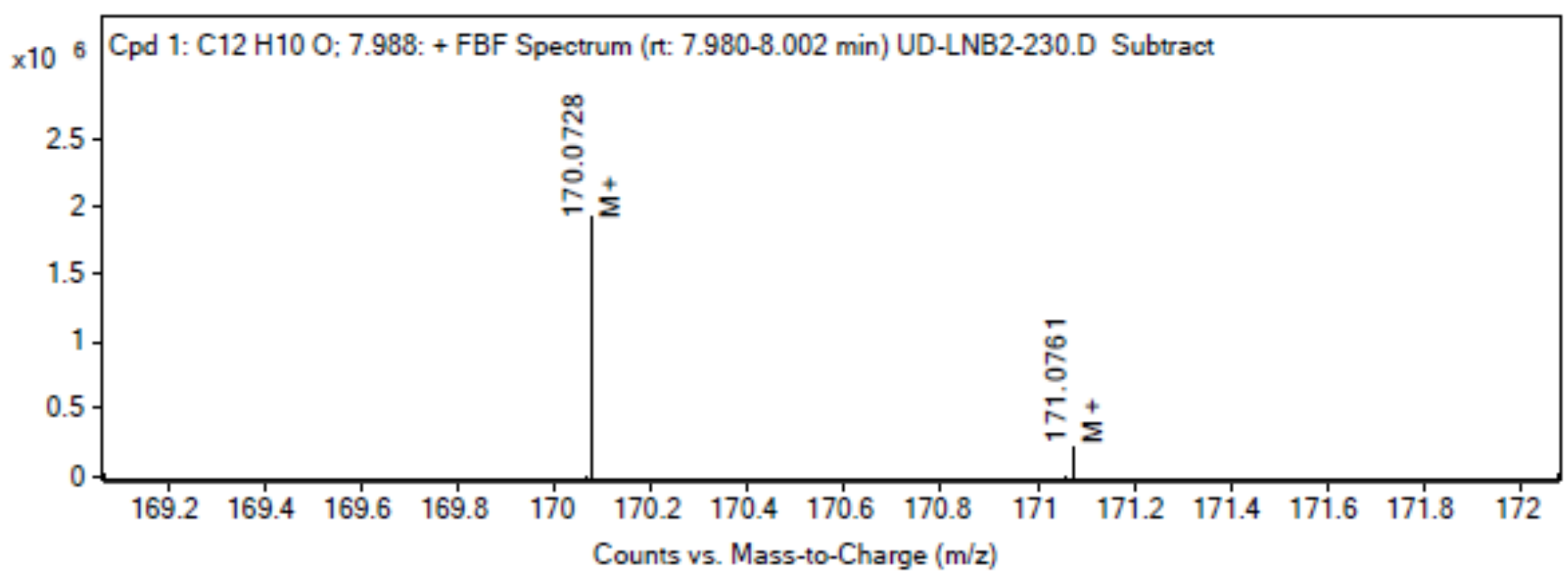

MS Zoomed Spectrum

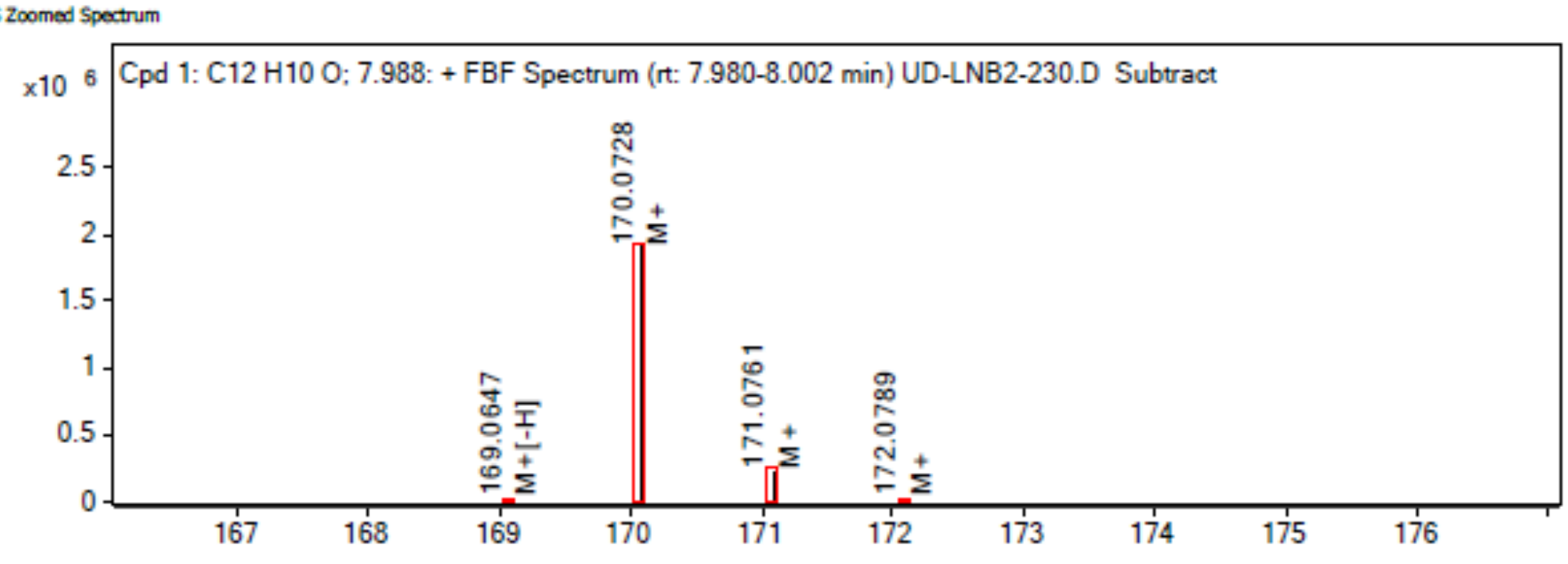

1-(4-vinylphenyl)pyrene (14)

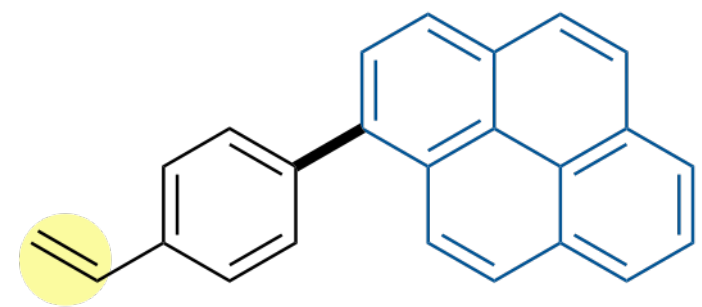

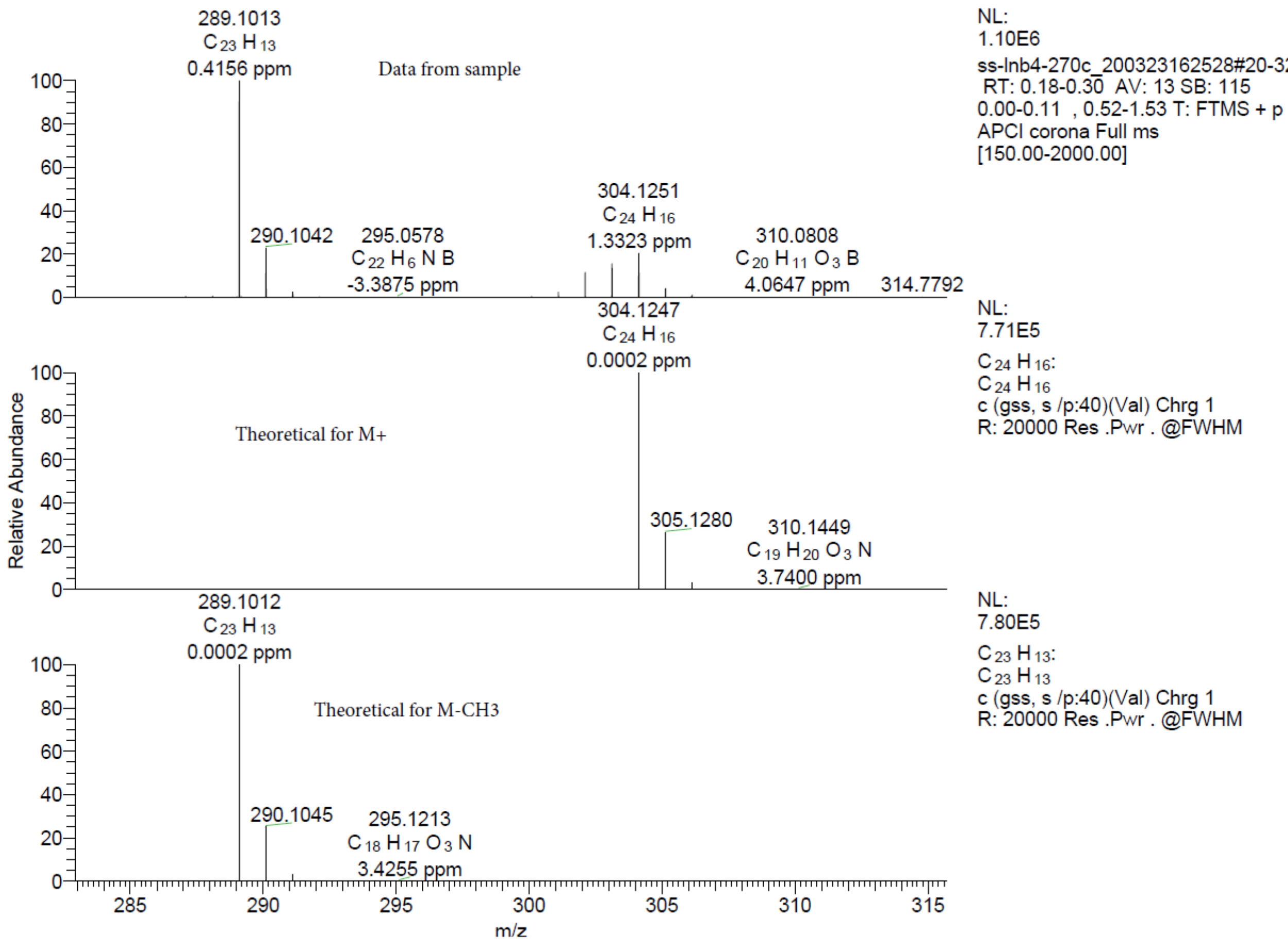

1-((4'-chloro-[1,1'-biphenyl]-4-yl)methyl)-5-vinyl-1*H*-indole (15)

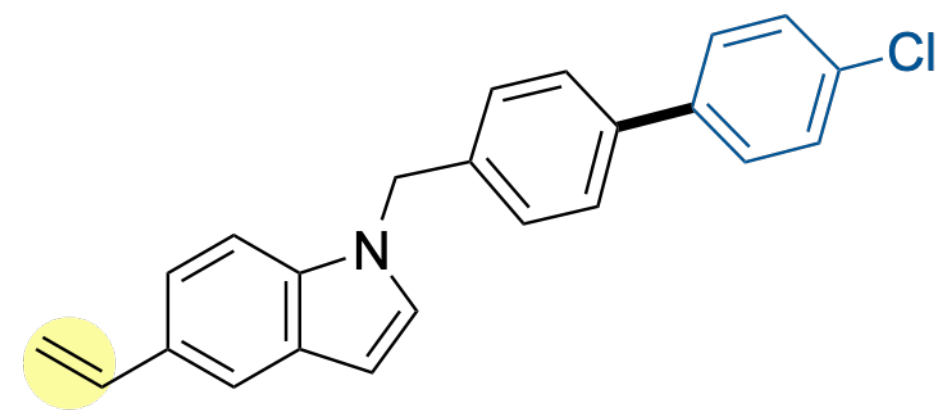

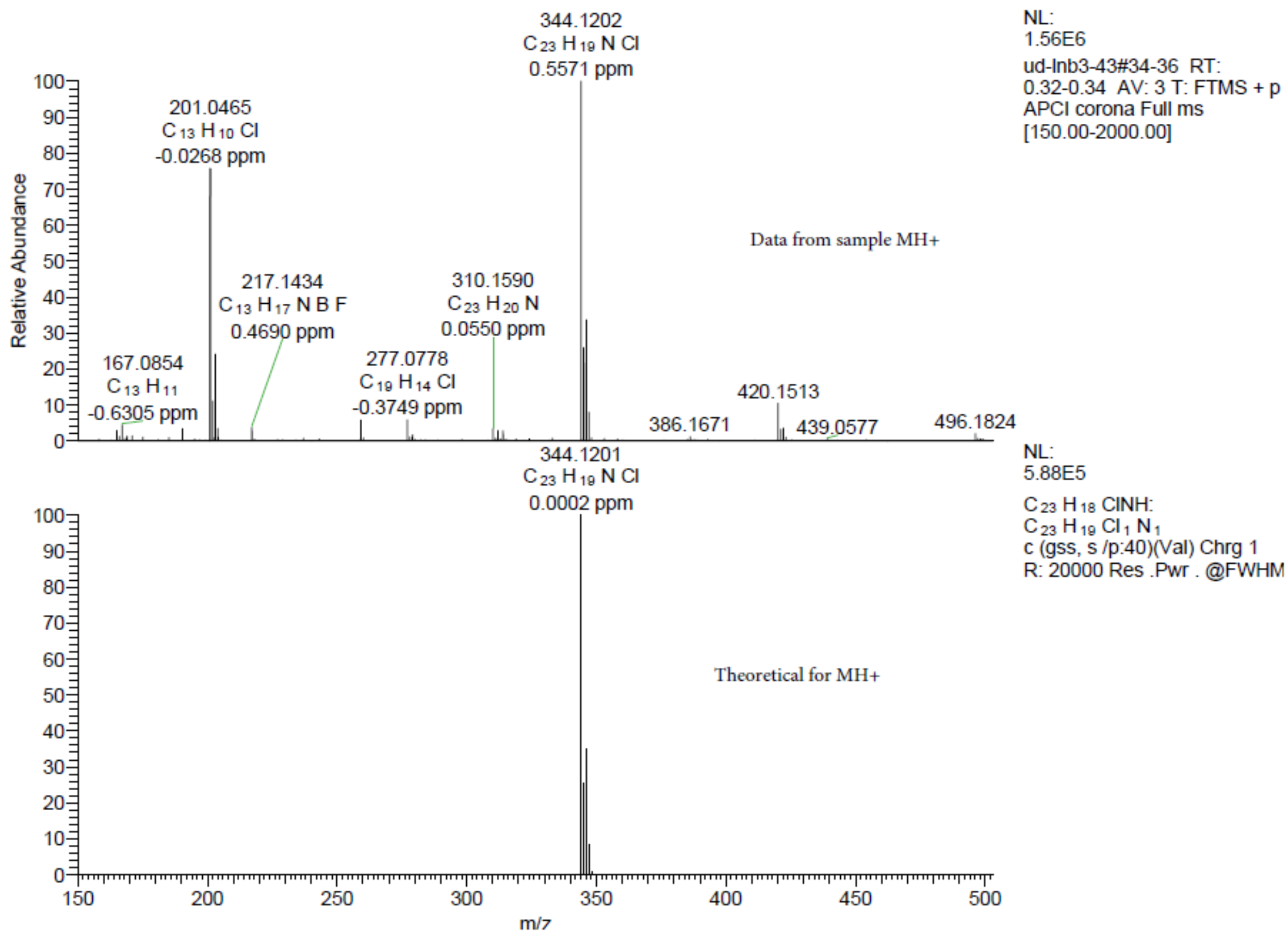

4'-((5-vinyl-1*H*-indol-1-yl)methyl)-[1,1'-biphenyl]-4-carbaldehyde (16)

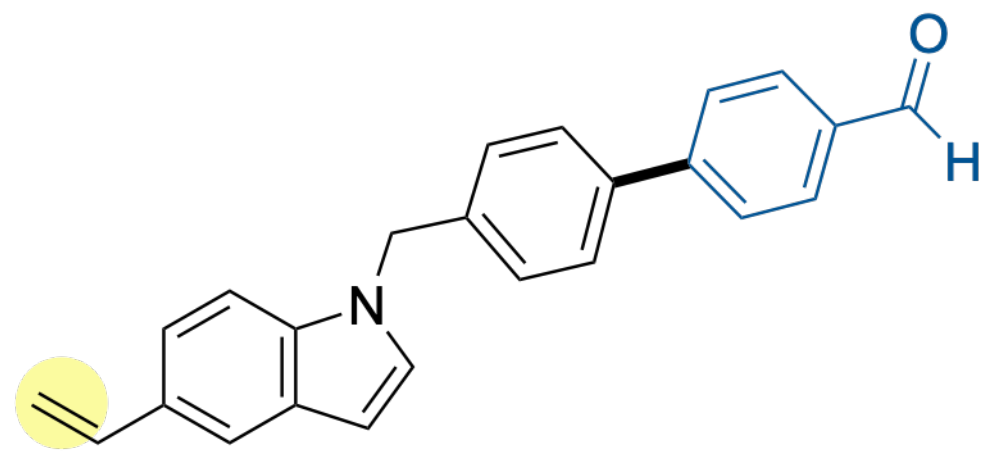

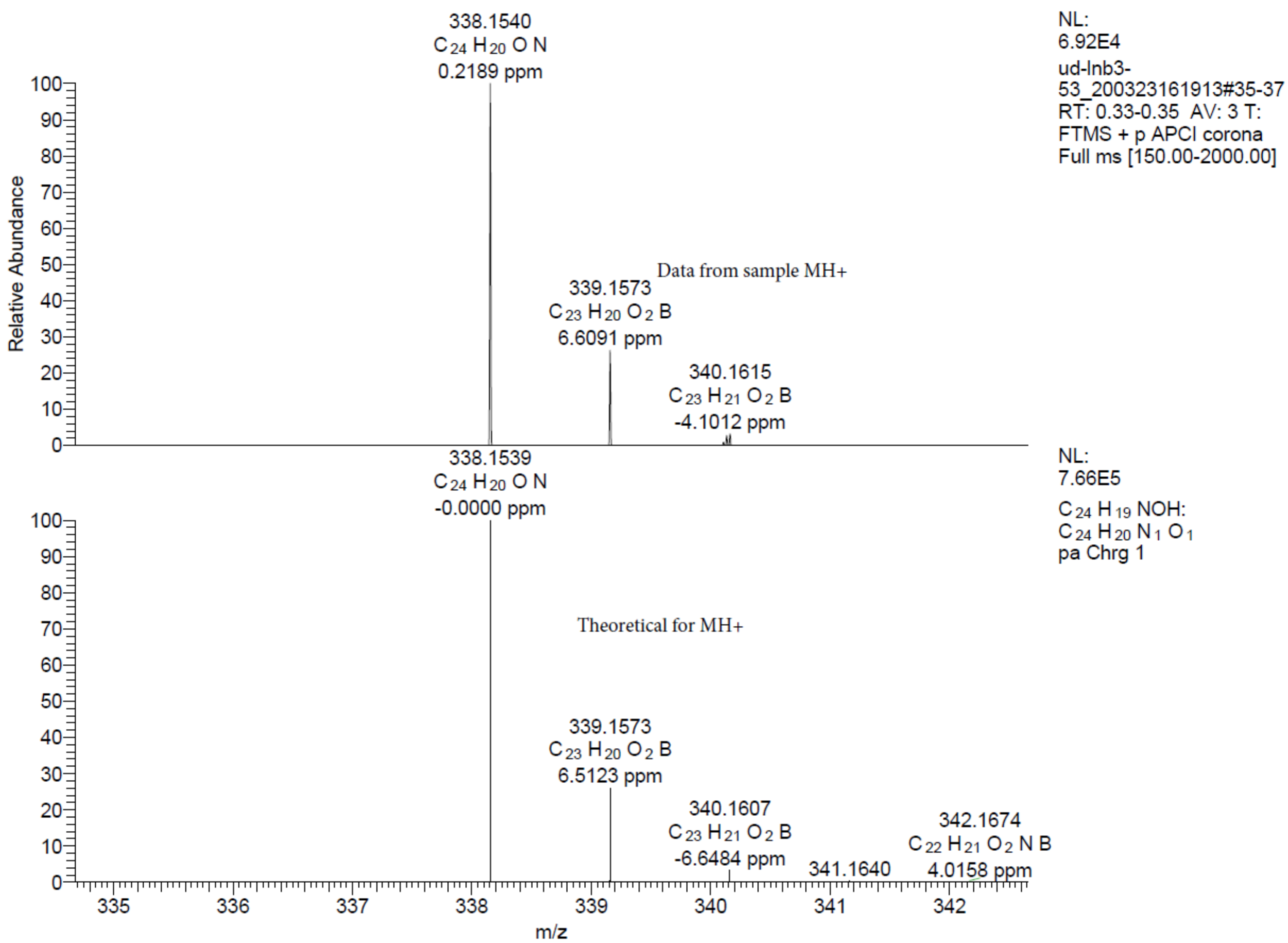

1-((4'-(Trifluoromethyl)-[1,1'-biphenyl]-4-yl)methyl)-2-vinyl-1*H*-indole (17)

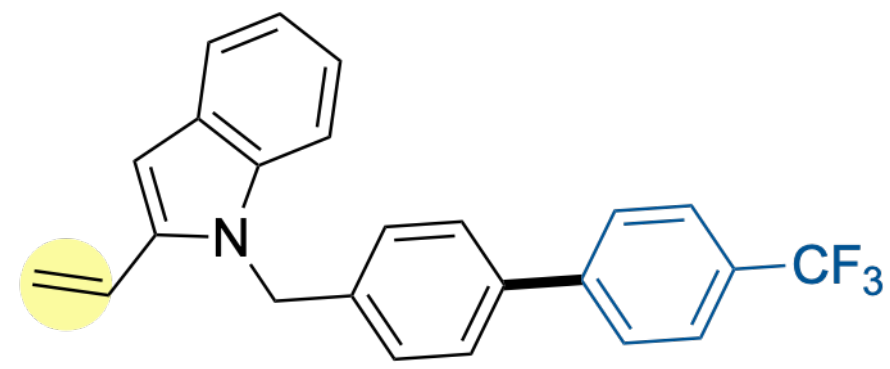

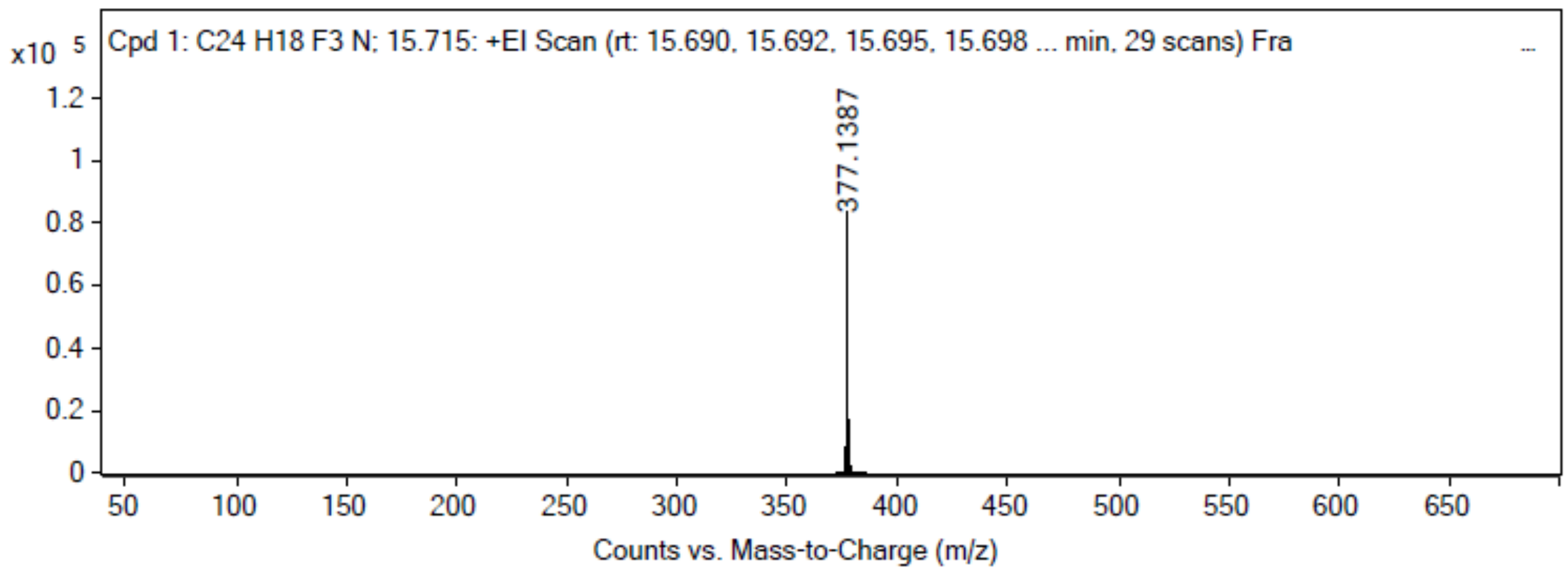

MS Zoomed Spectrum

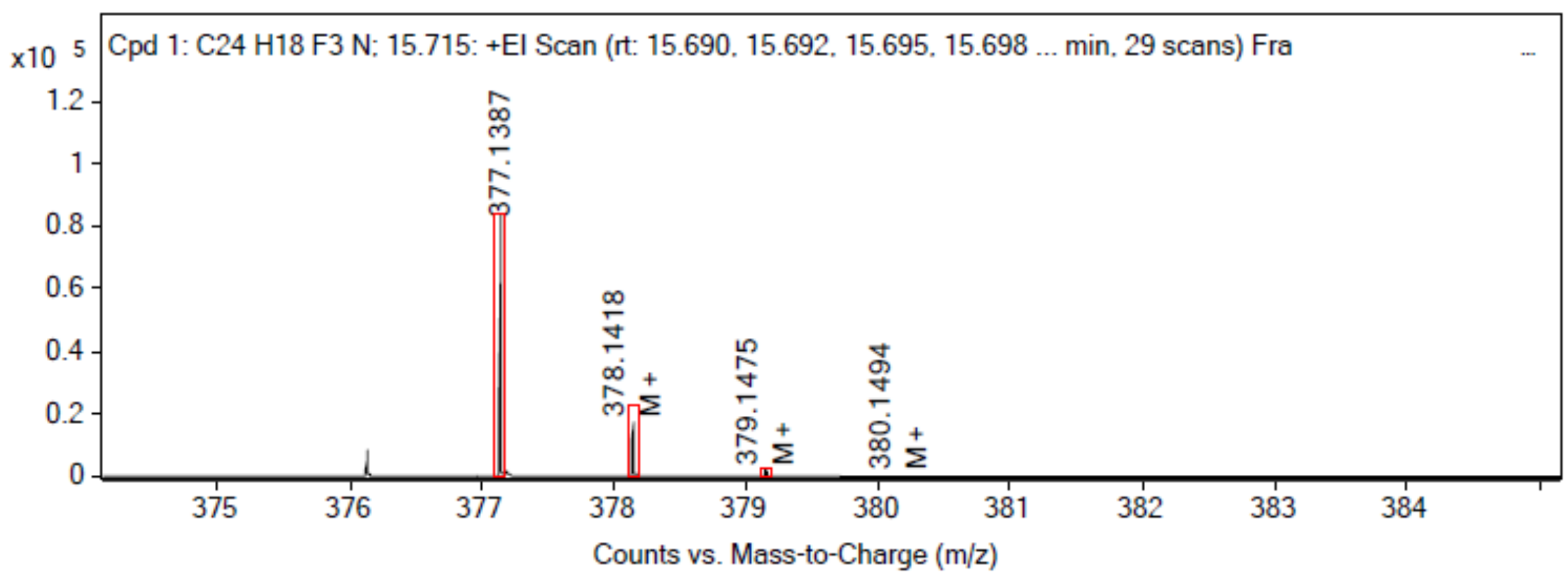

MS Spectrum Peak List

m/z	Calc *m/z*	Diff(ppm)	z	Abund	Ion
377.1387	377.1386	-0.22	1	83847.94	M+
377.1387				83874.89	
378.1418	378.1419	0.14	1	17232.36	M+
379.1475	379.1452	-6.16	1	1926.72	M+
380.1494	380.1484	-2.38	1	168.48	M+

4-vinyl-1,1’:2’,1’’:2’’,1’’’-quaterphenyl (18)

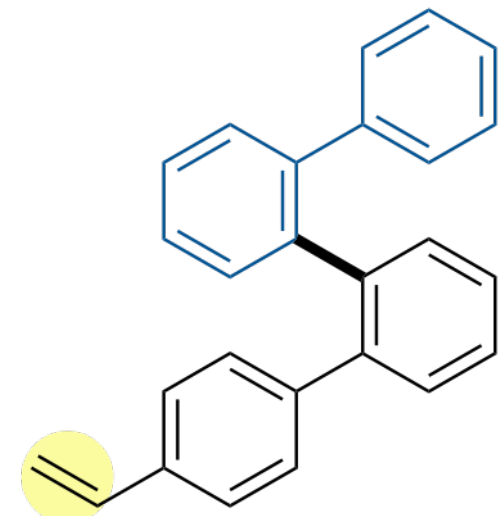

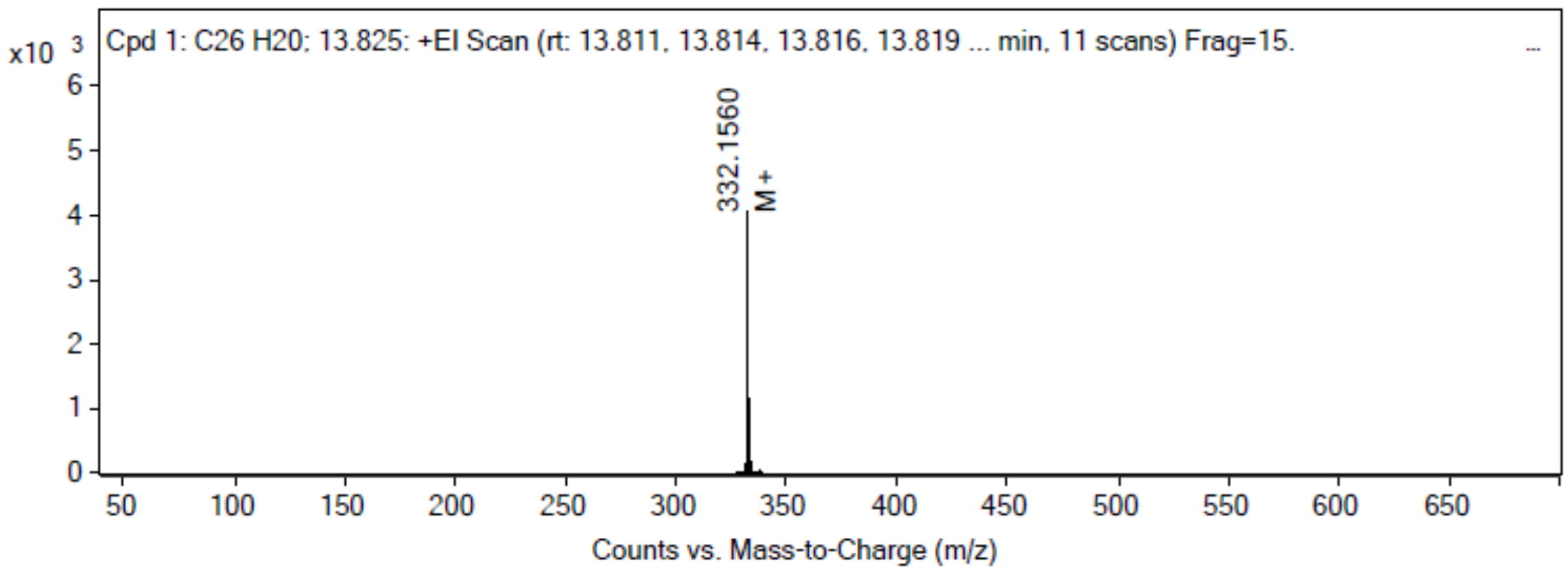

MS Zoomed Spectrum

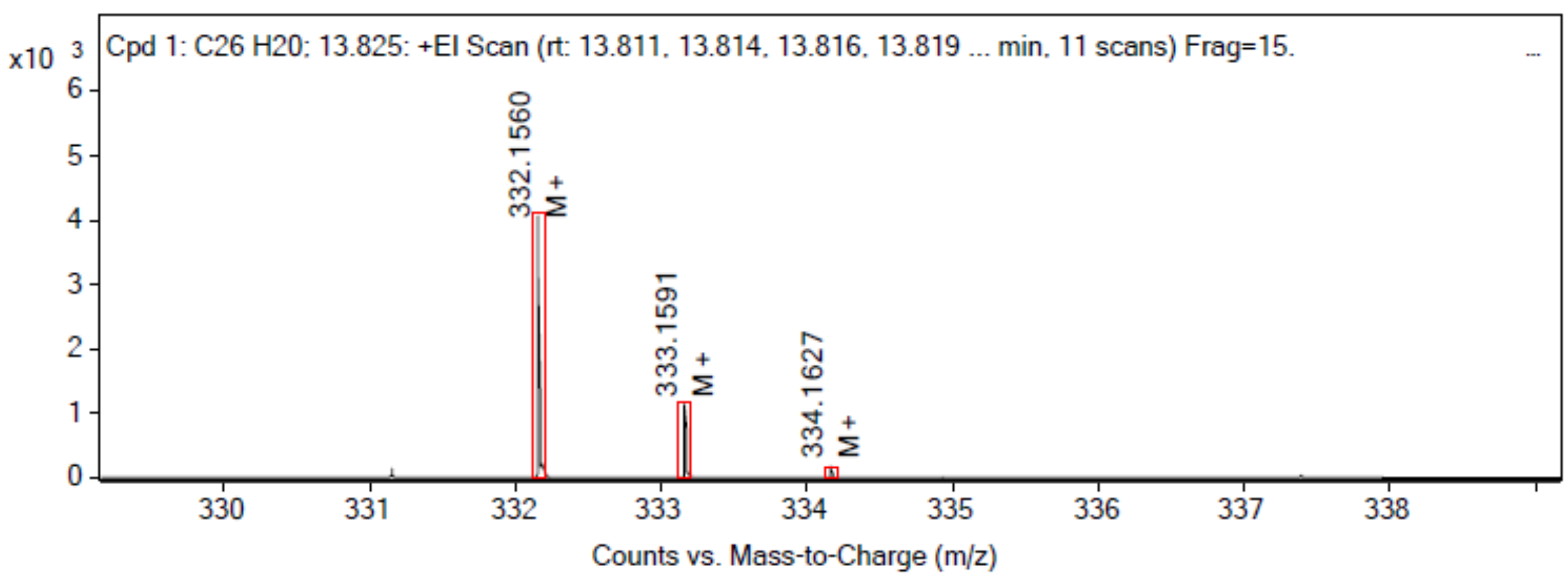

MS Spectrum Peak List

m/z	Calc *m/z*	Diff(ppm)	z	Abund	Ion
332.156	332.156	-0.25	1	4071.85	M+
332.1561				4046.65	
333.1591	333.1593	0.61	1	1171.72	M+
334.1627	334.1627	-0.05	1	218.79	M+

Compound Label	RT	Algorithm
Cpd 3: 13.936	13.936	Find by Integration

Compound Chromatograms

5-(4-vinylphenyl)thiophene-2-carbaldehyde (19)

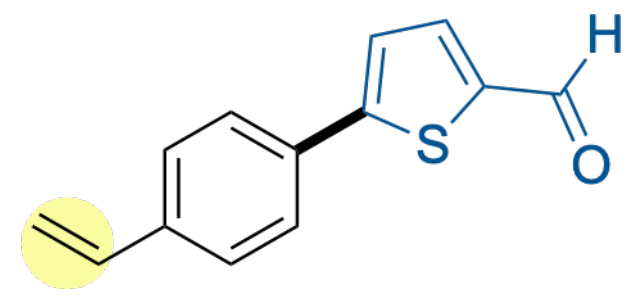

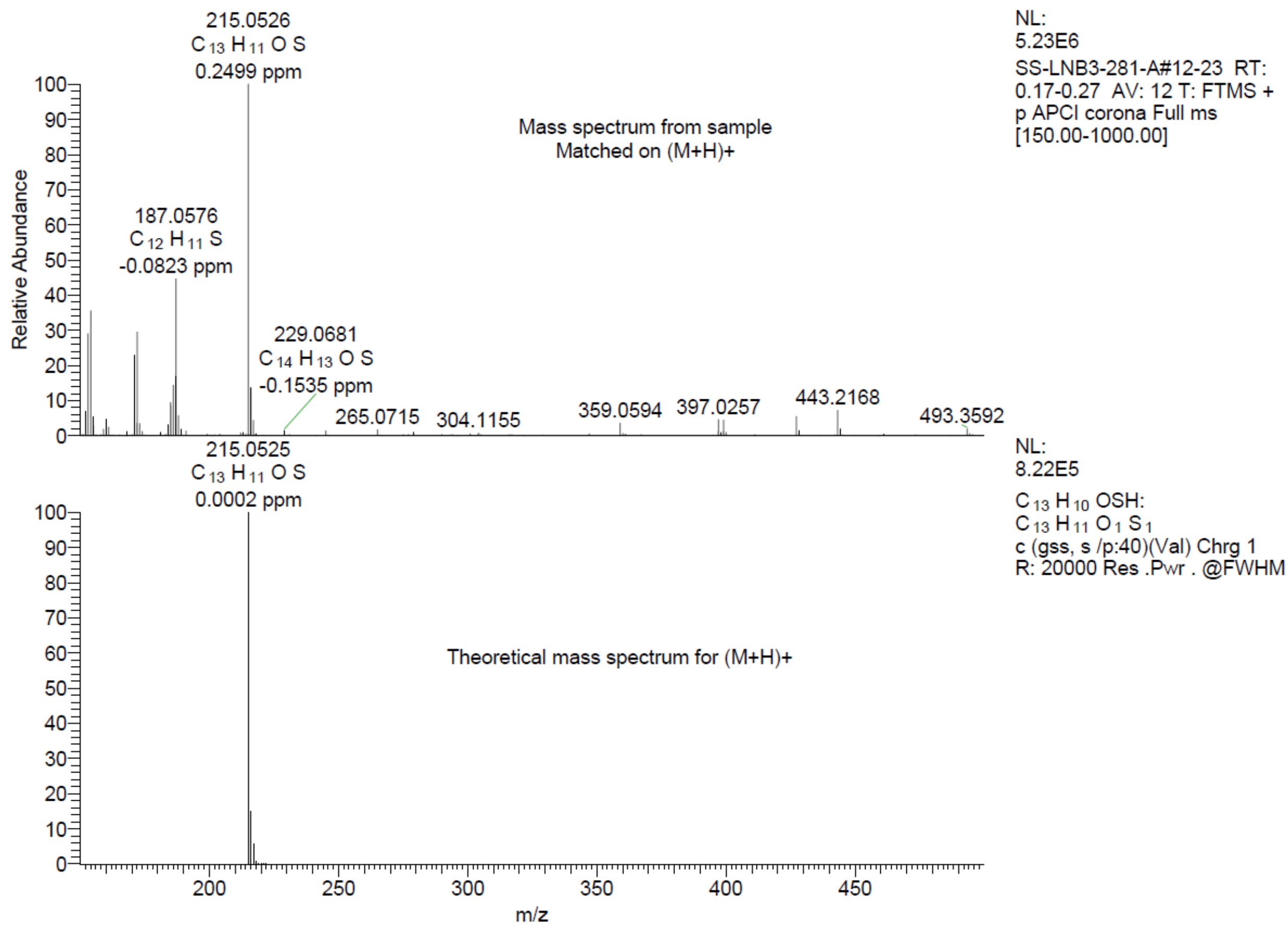

3-(5-vinylthiophen-3-yl)furan (20)

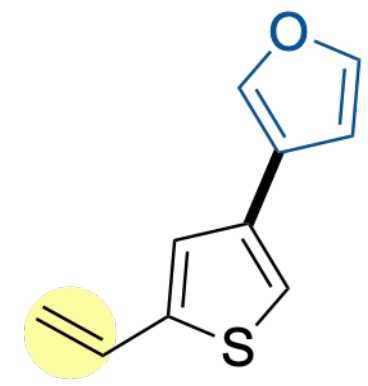

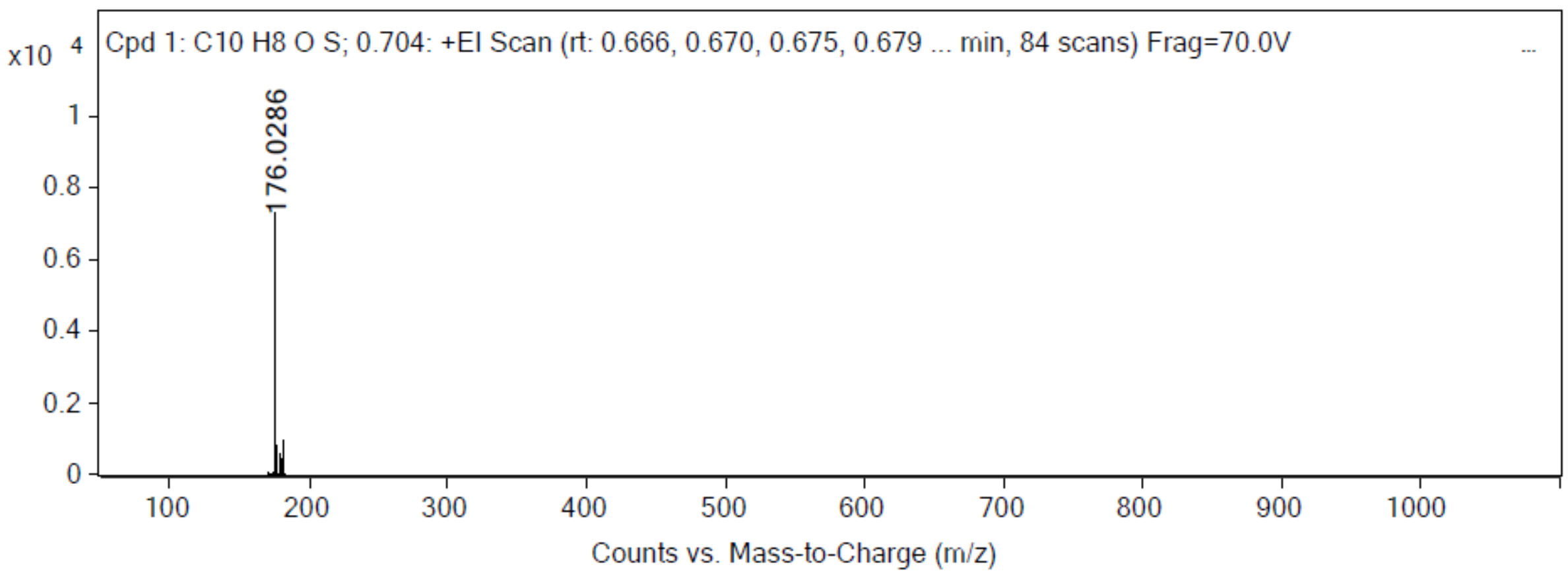

MS Zoomed Spectrum

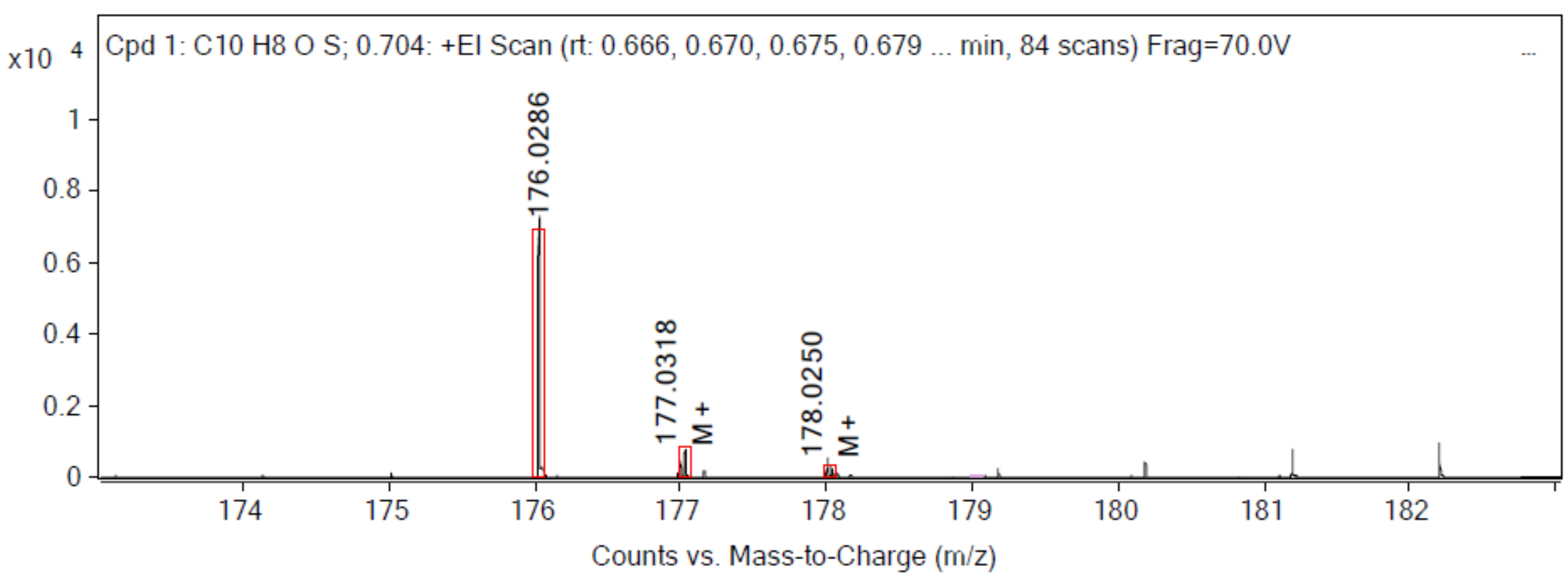

MS Spectrum Peak List

m/z	Calc *m/z*	Diff(ppm)	z	Abund	Ion
176.0286	176.029	2.75	1	6954.3	M+
176.0286				7307.92	
177.0318	177.0322	2.05	1	752.32	M+
178.025	178.0264	7.81	1	373.74	M+

1-benzyl-5-(naphthalen-1-yl)-3-vinyl-1*H*-indole (21)

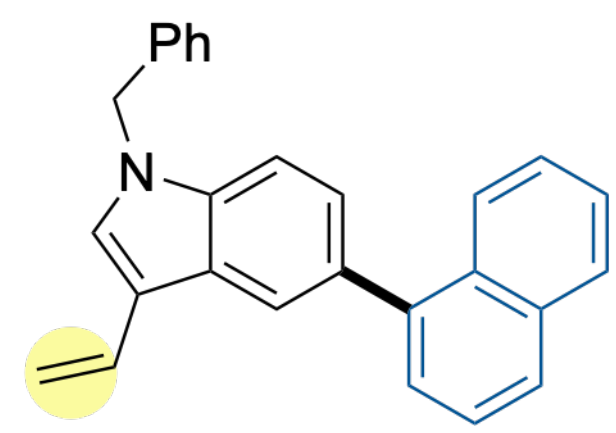

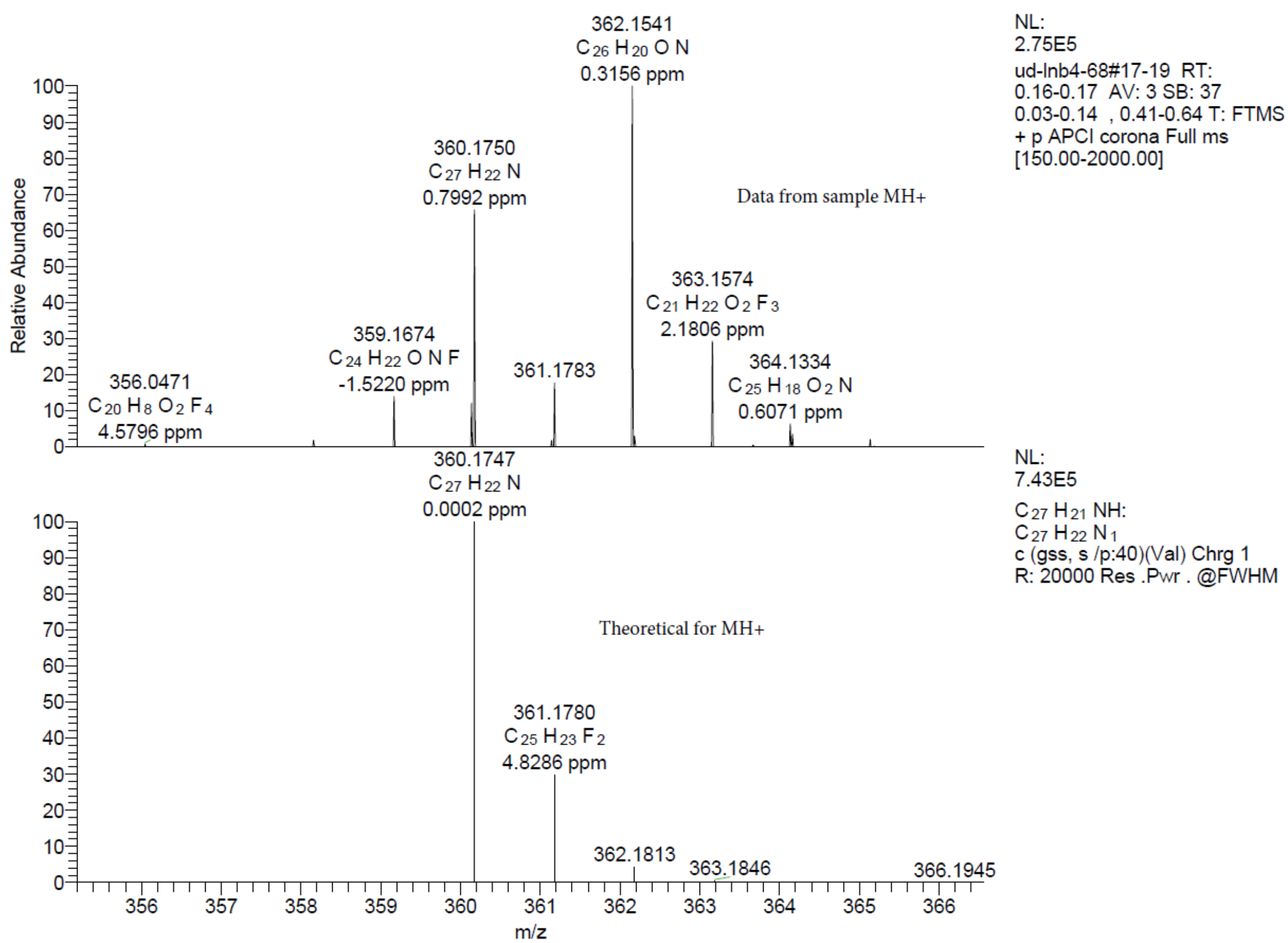

3-(4-((4-vinylphenoxy)methyl)phenyl)furan (22)

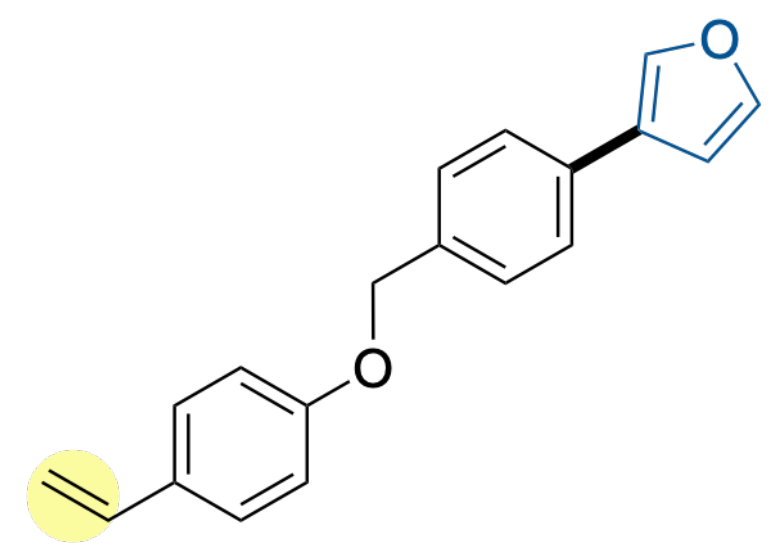

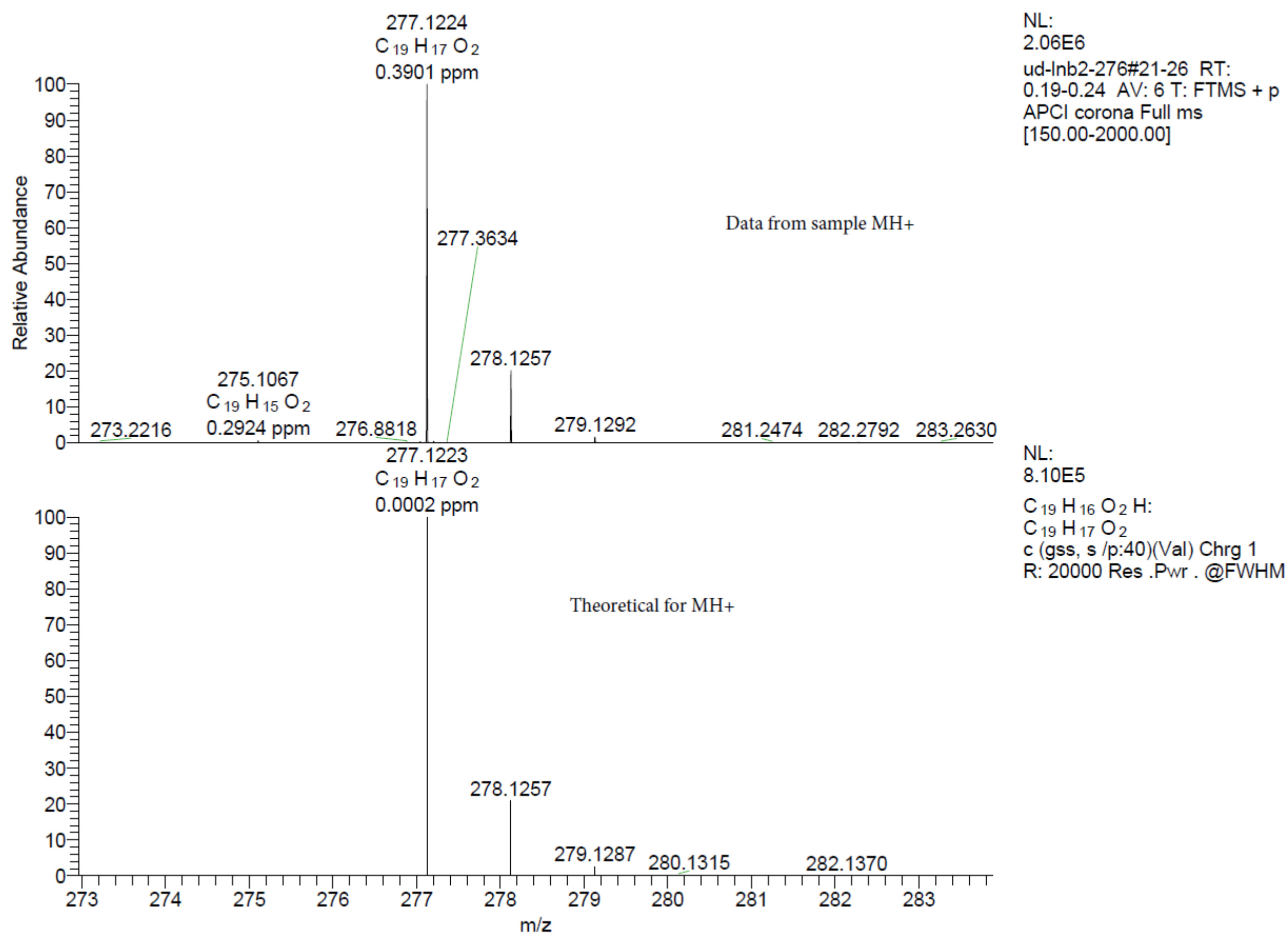

2-methoxy-1-(4'-vinyl-[1,1'-biphenyl]-2-yl)naphthalene (23)

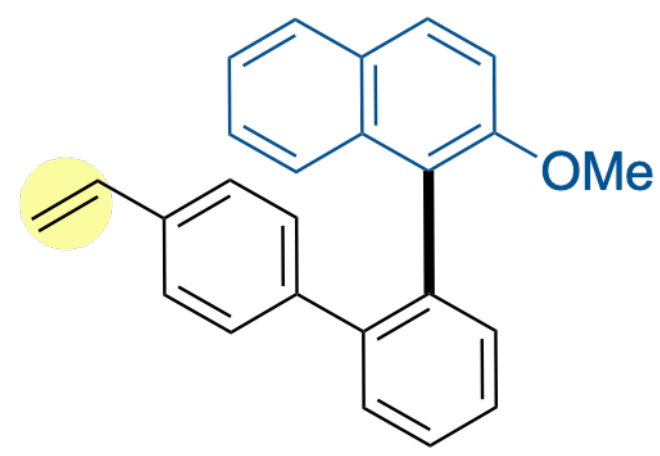

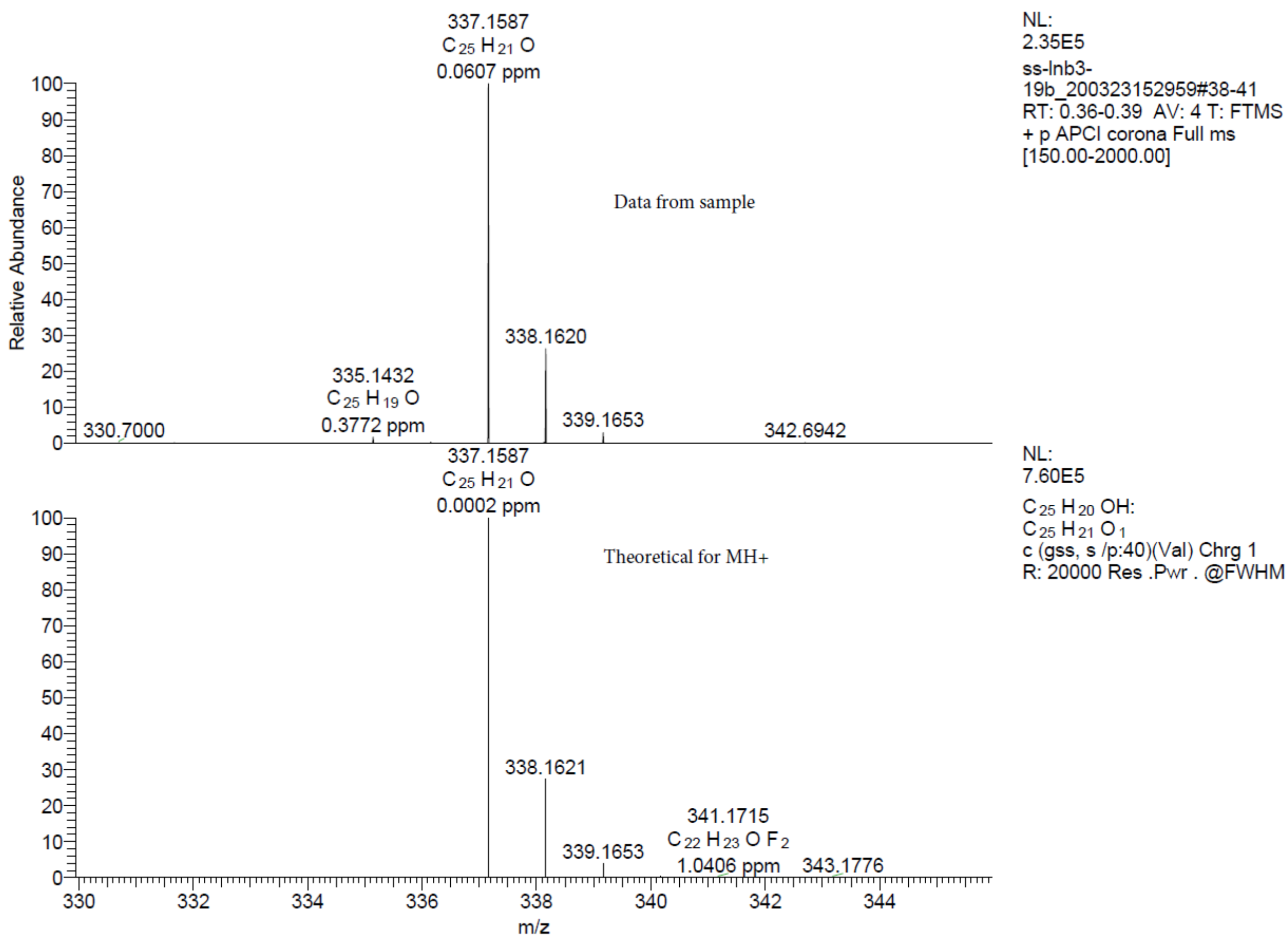

1-benzyl-5-(2-methoxyphenyl)-3-vinyl-1*H*-indole (24)

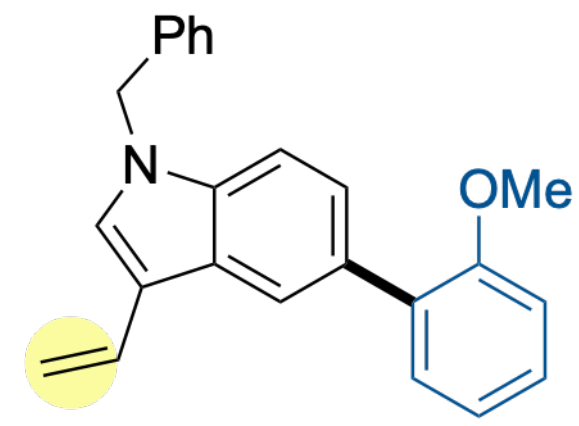

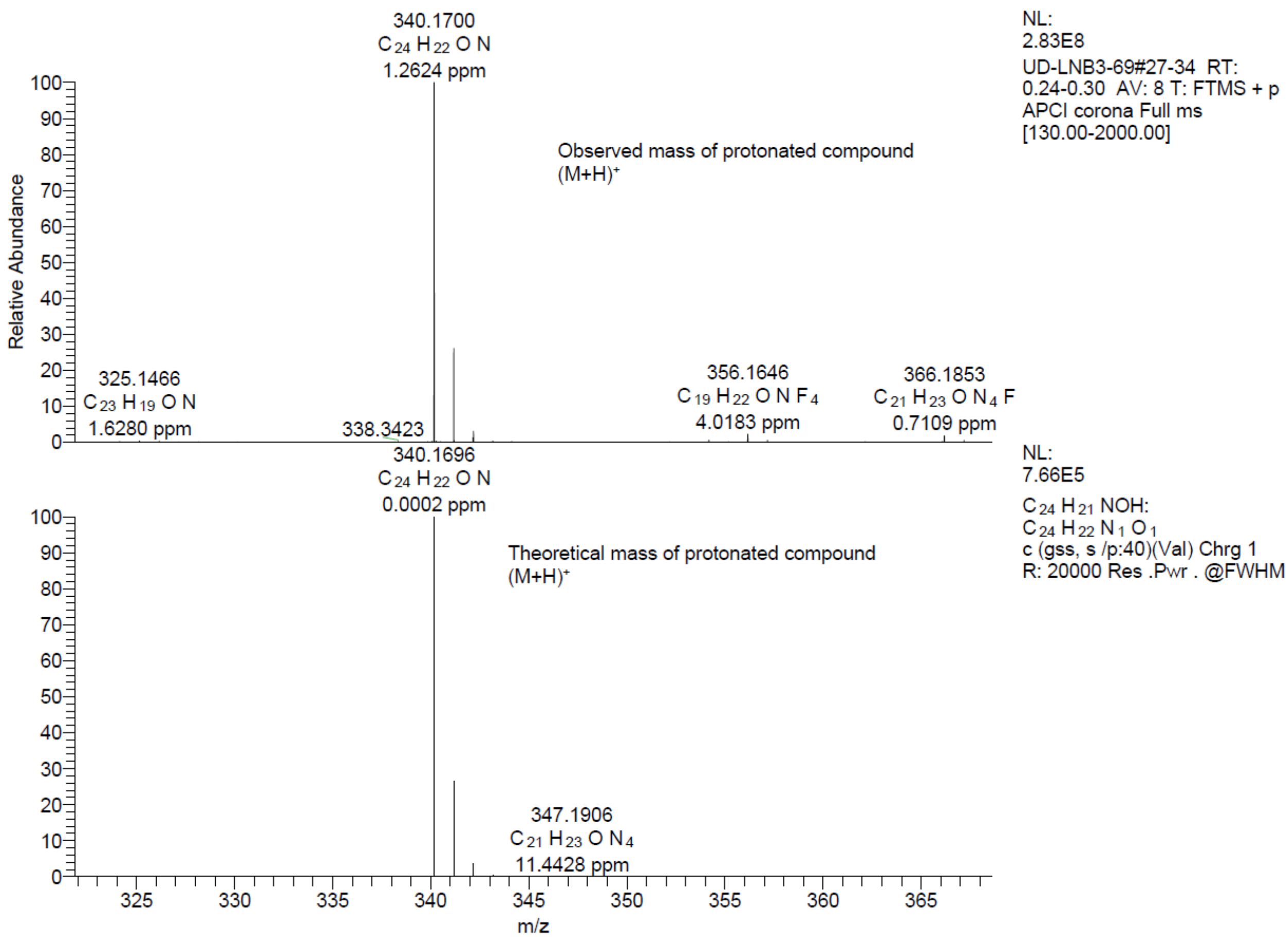

3,5-Dimethyl-4-(4-vinylphenyl)isoxazole (25)

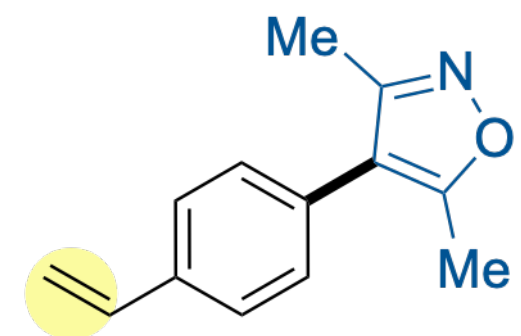

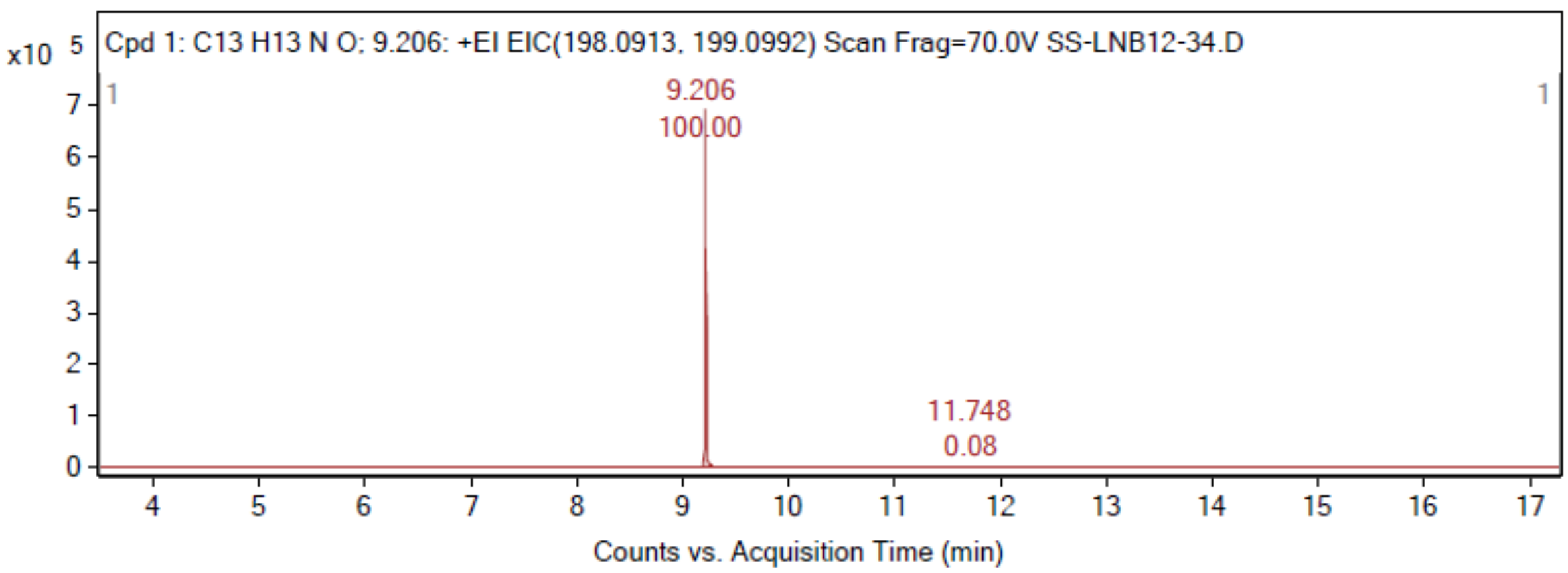

MS Spectrum

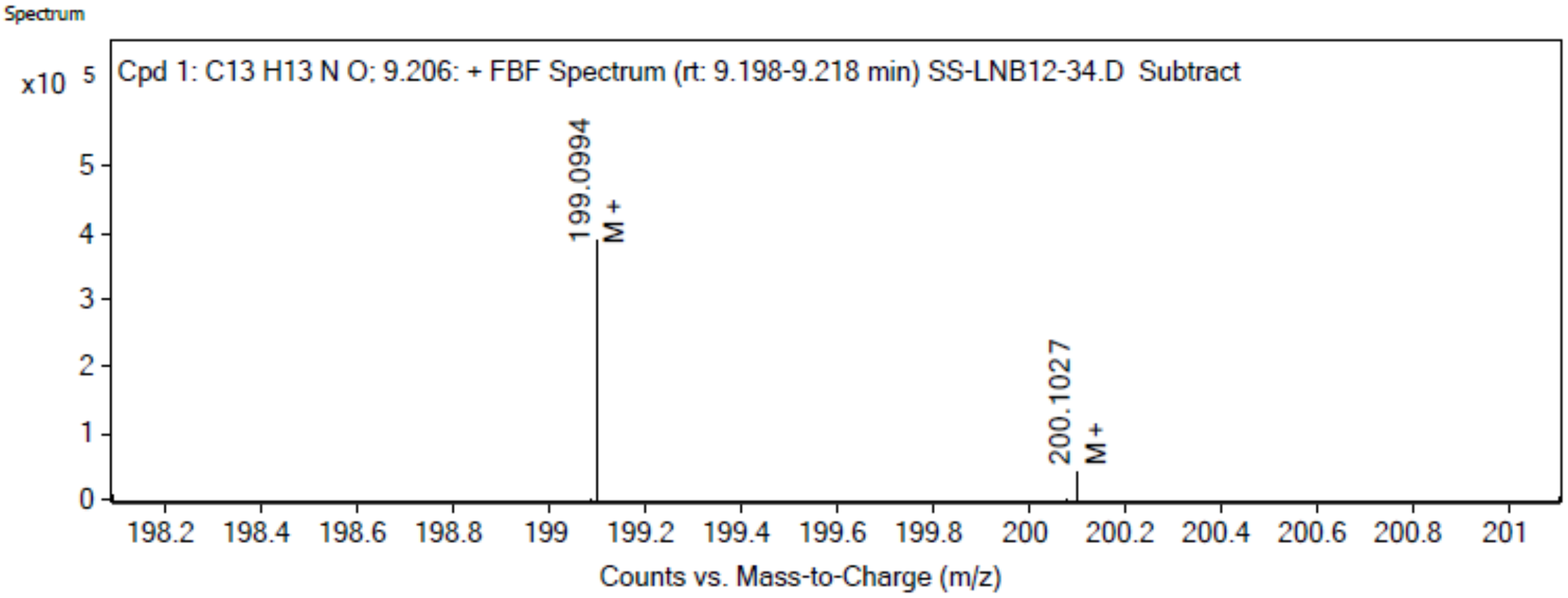

MS Zoomed Spectrum

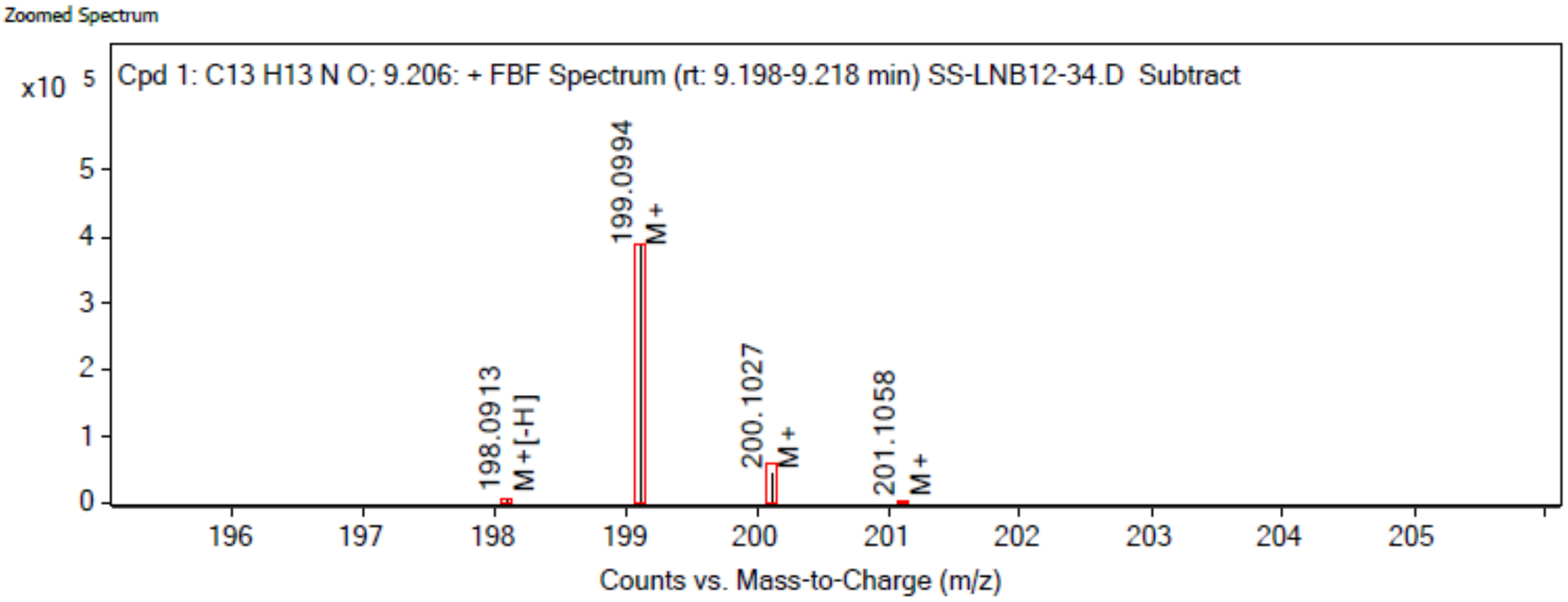

2-(benzyloxy)-4-fluoro-4'-((4-vinylphenoxy)methyl)-1,1'-biphenyl (26)

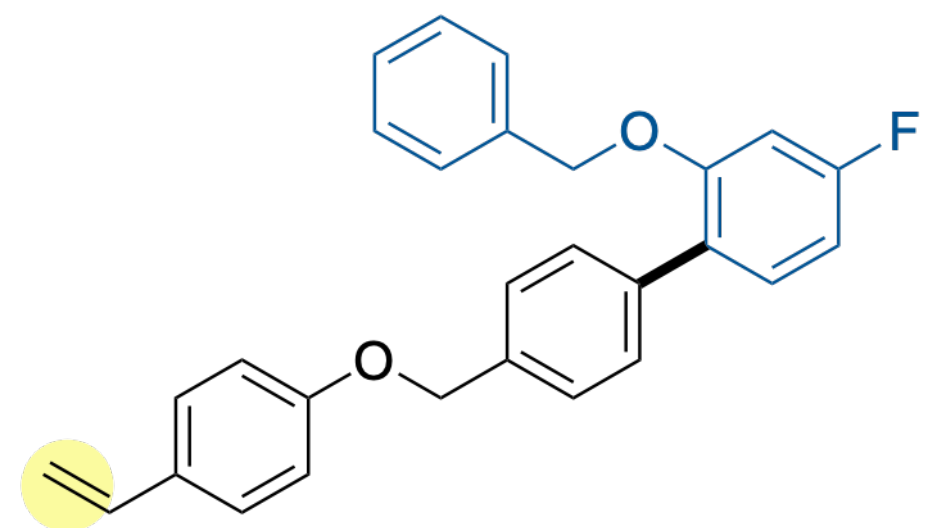

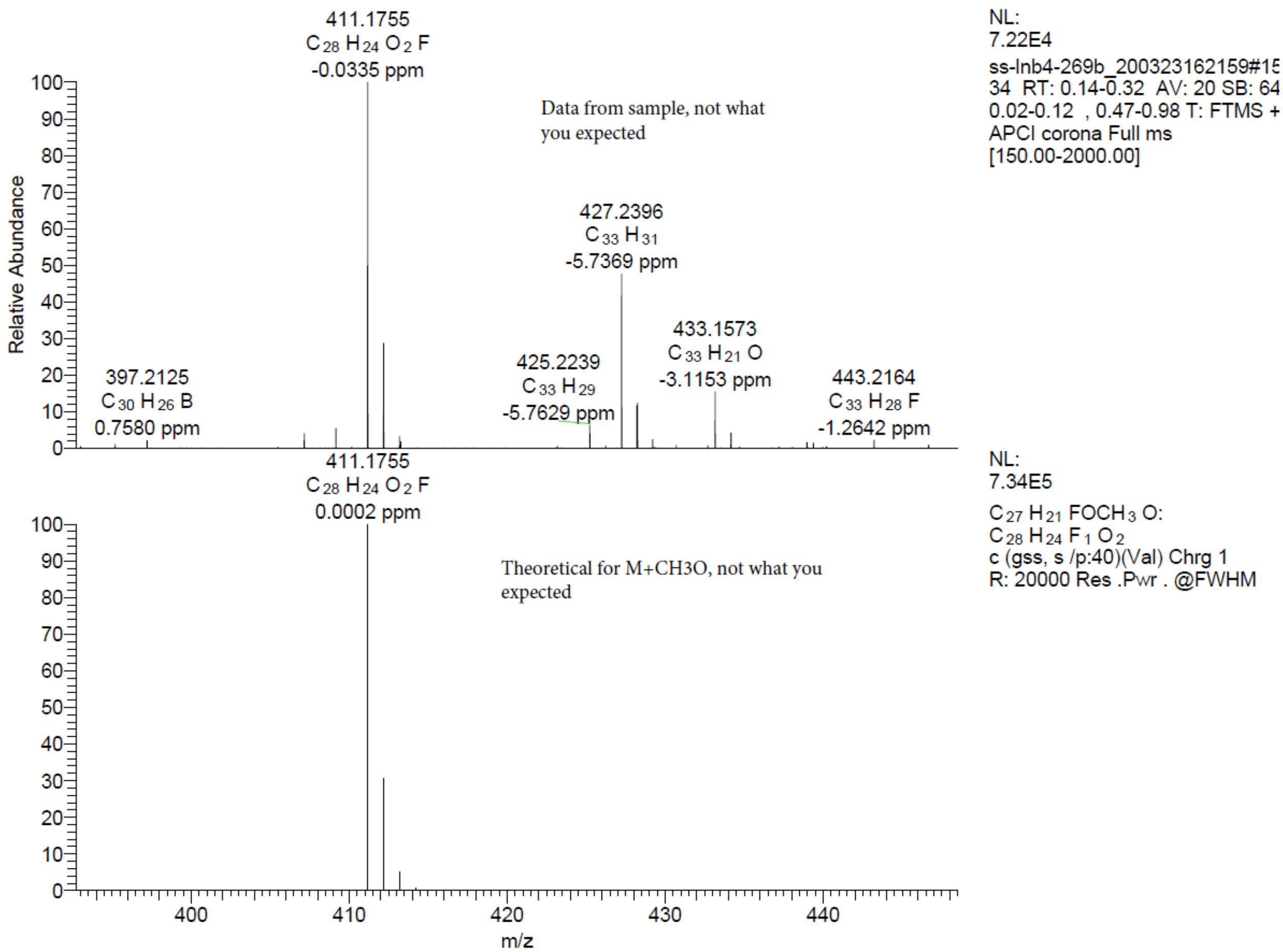

4-nitro-4'-((4-vinylphenoxy)methyl)-1,1'-biphenyl (27)

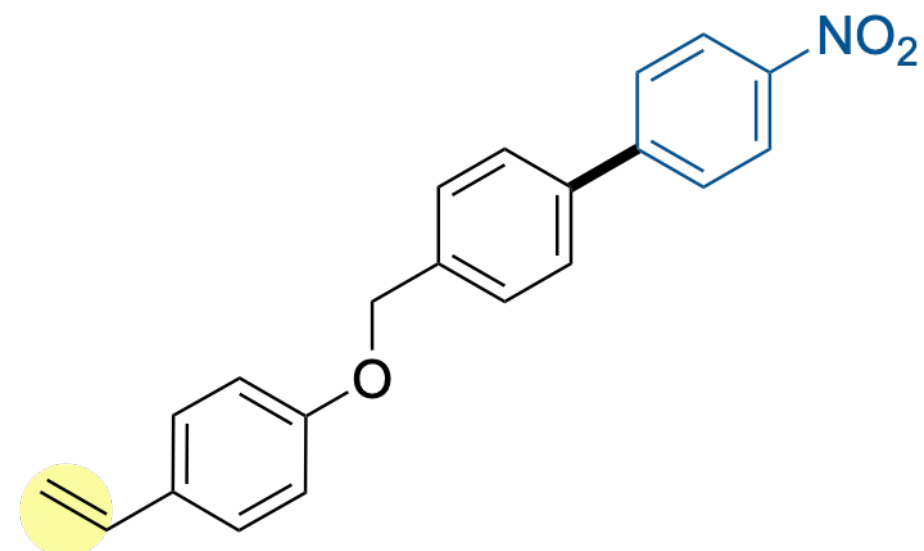

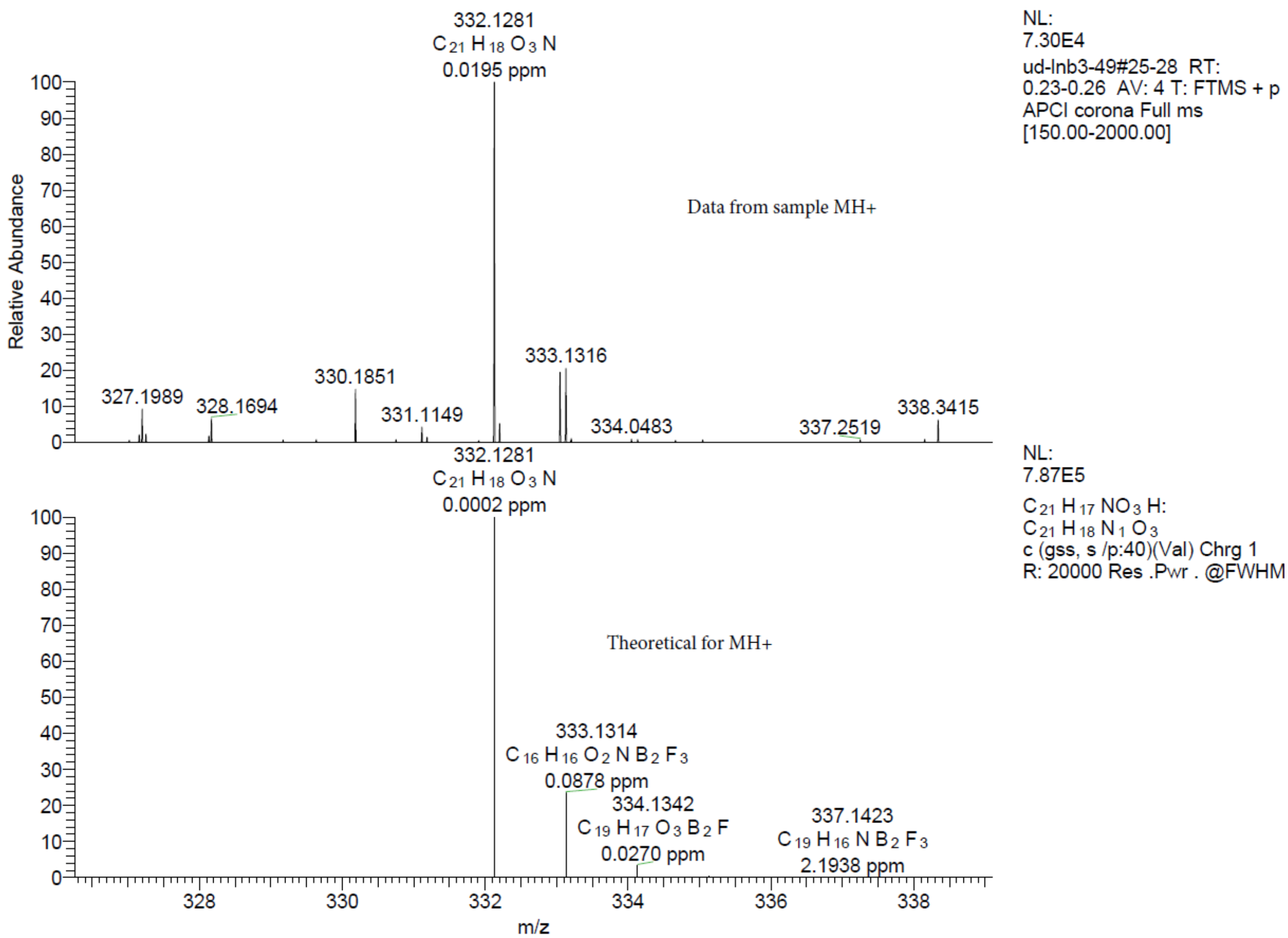

1-(2-(1-phenylvinyl)phenyl)naphthalene (28)

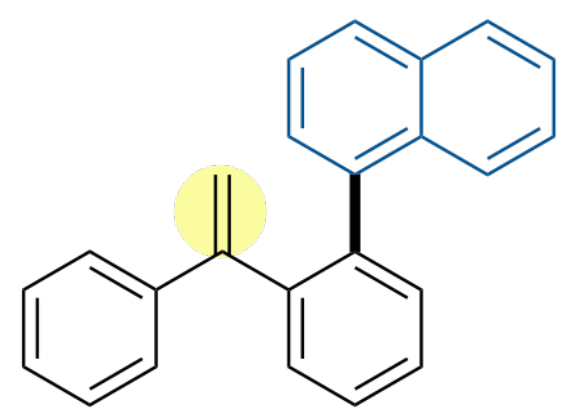

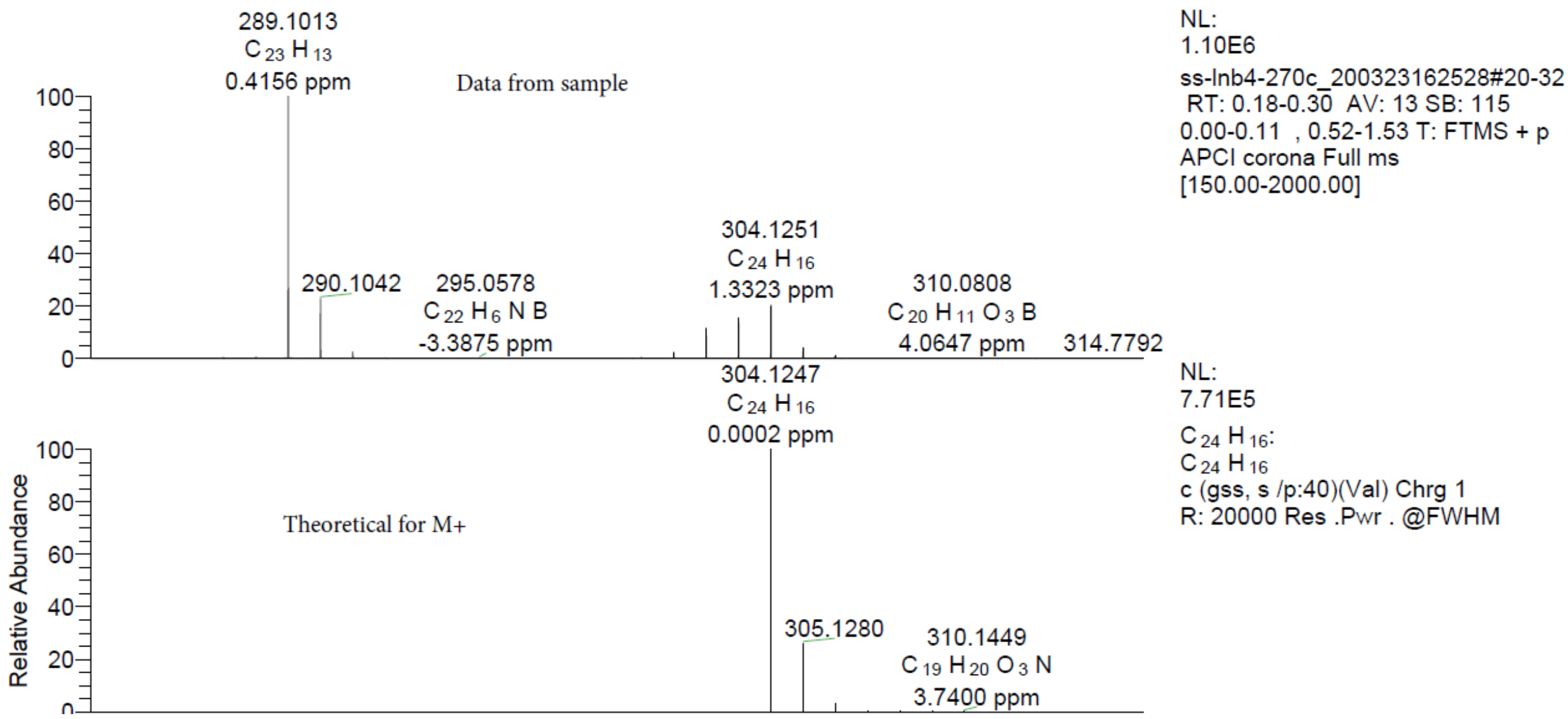

4-(trifluoromethyl)-4'-((4-vinylphenoxy)methyl)-1,1'-biphenyl (29)

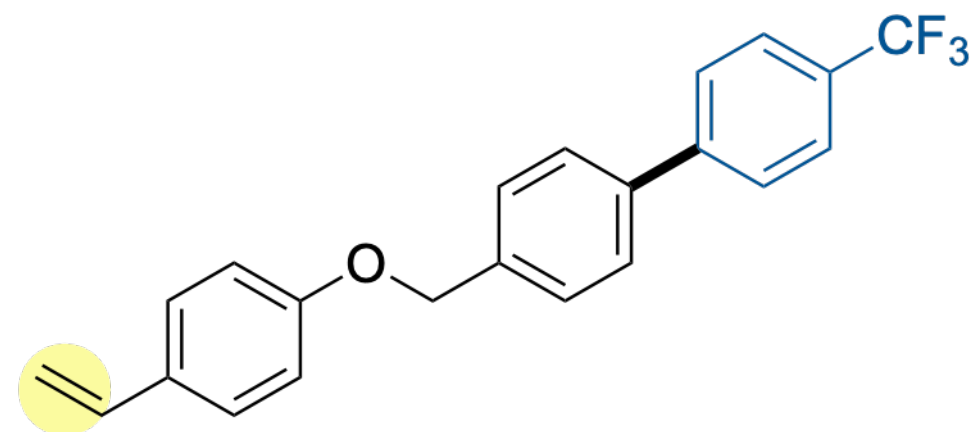

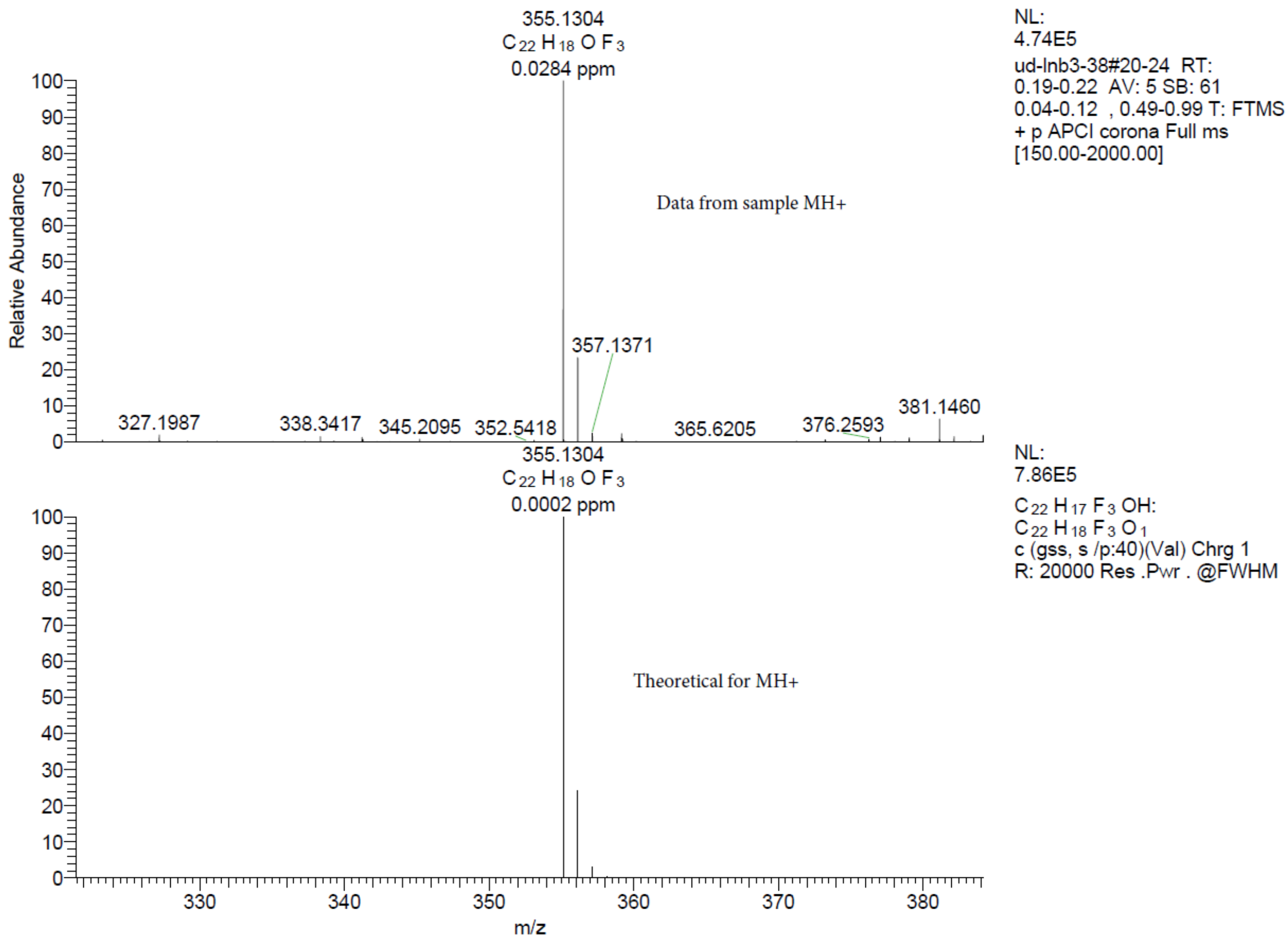

1-(4-((4-vinylphenoxy)methyl)phenyl)pyrene (30)

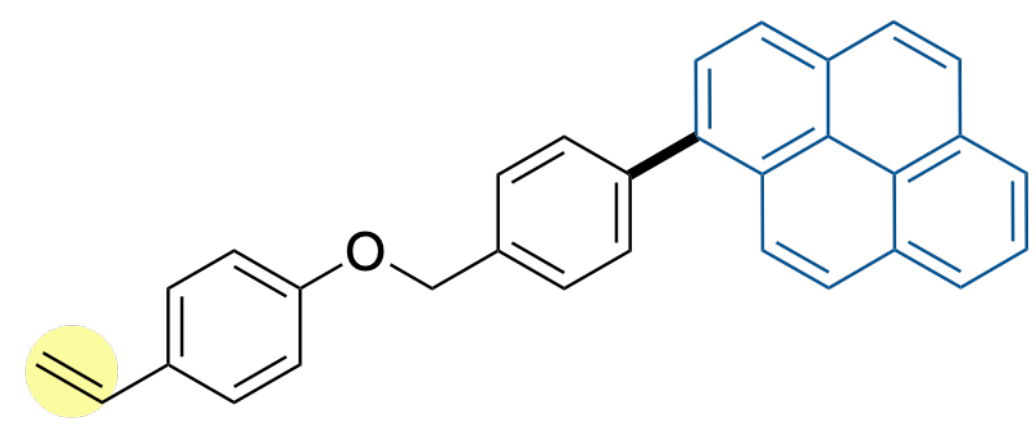

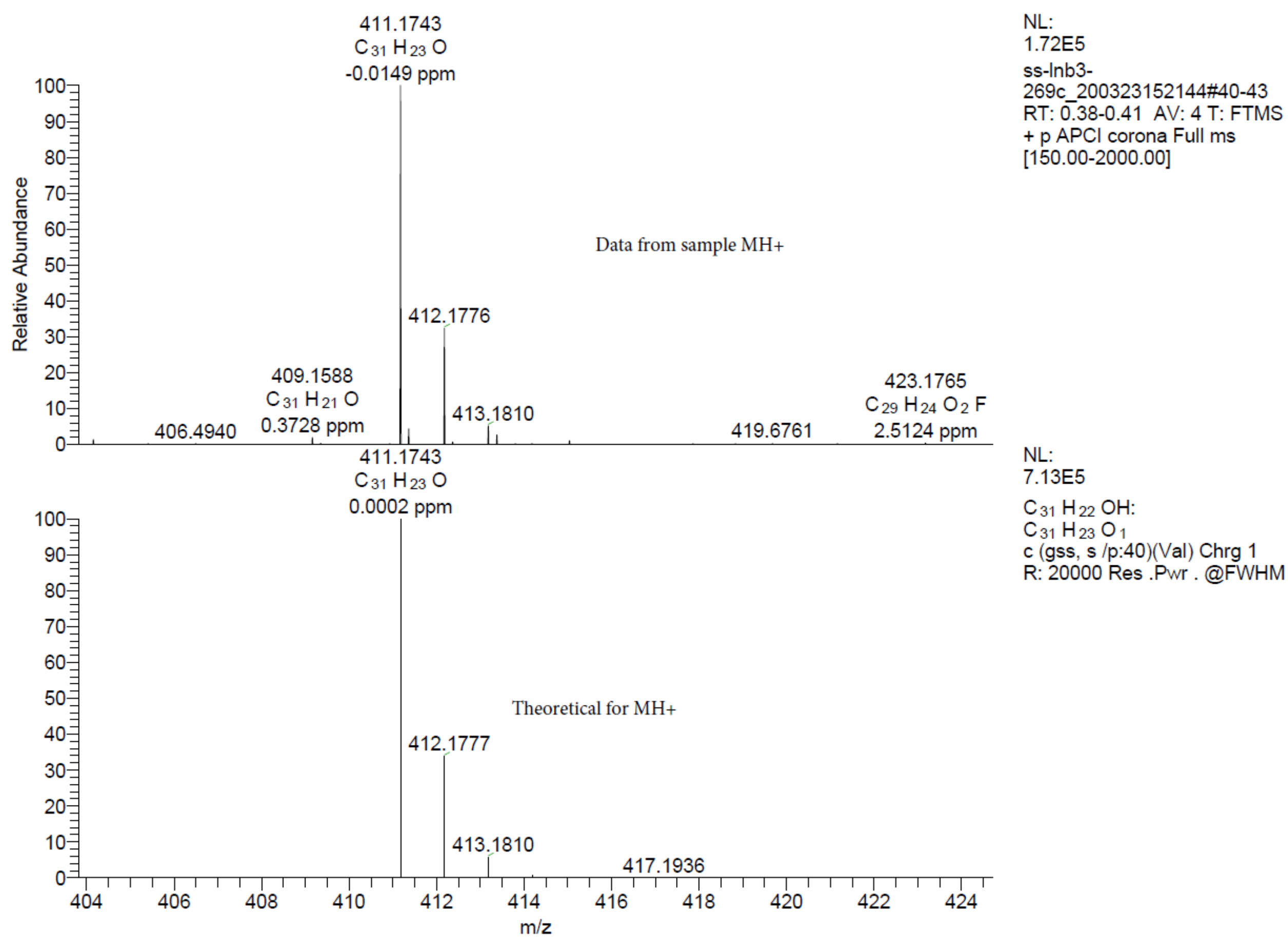

2-methoxy-4-(2-(1-(4-nitrophenyl)vinyl)phenyl)pyridine (31)

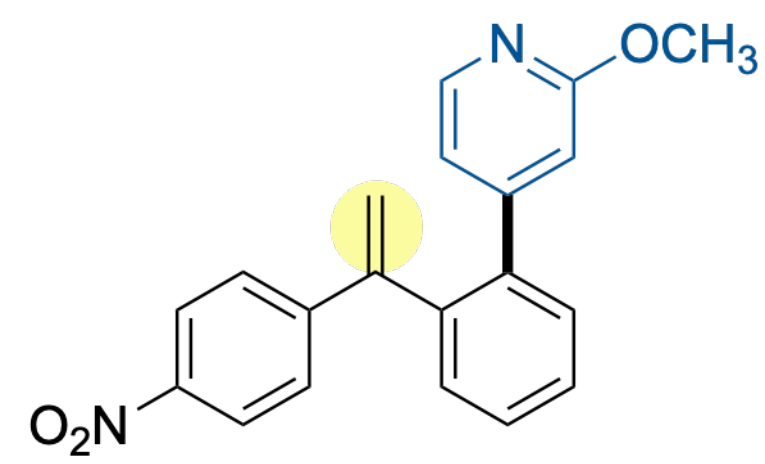

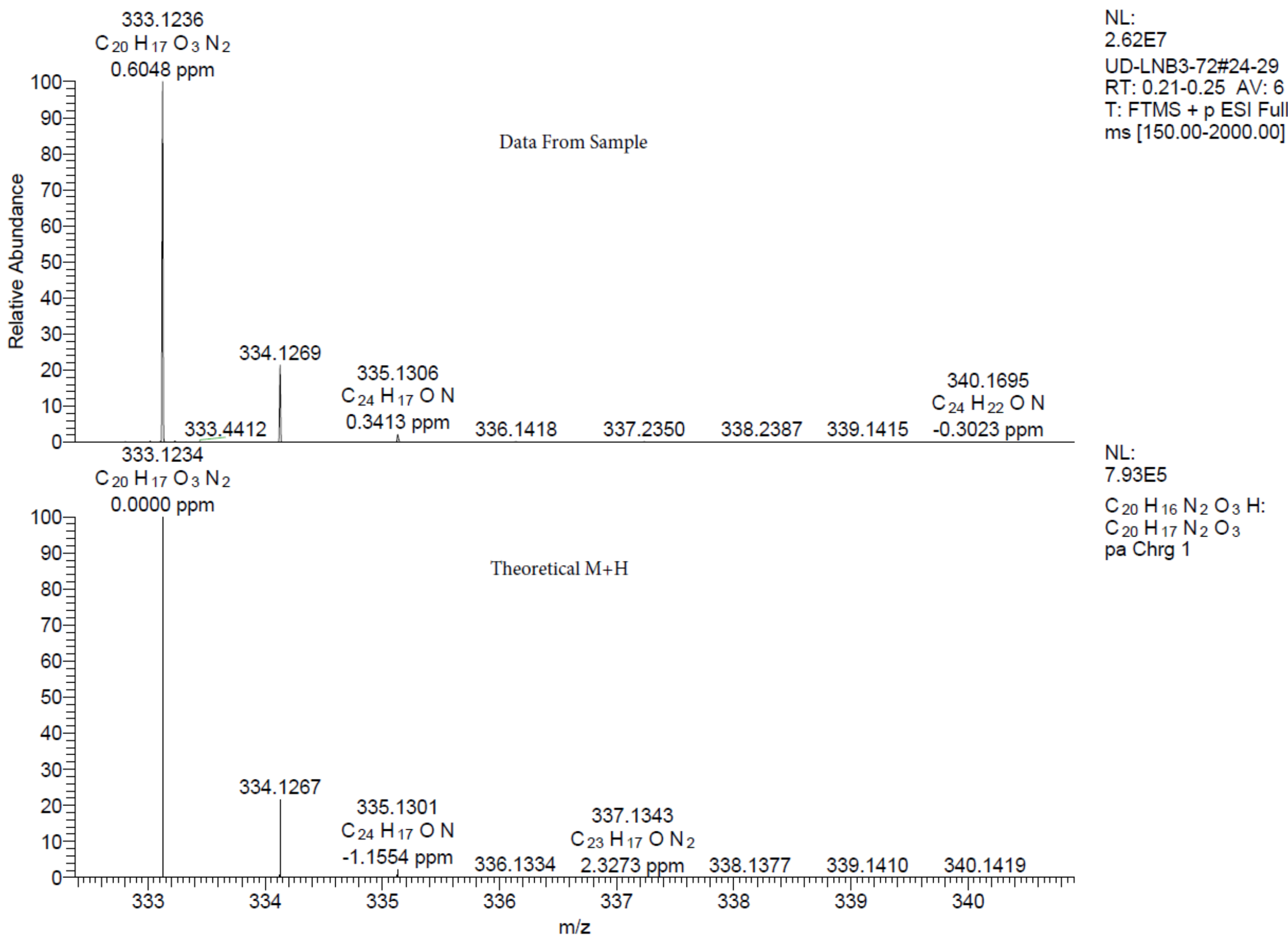

4-vinyl-4'-((4-vinylphenoxy)methyl)-1,1'-biphenyl (32)

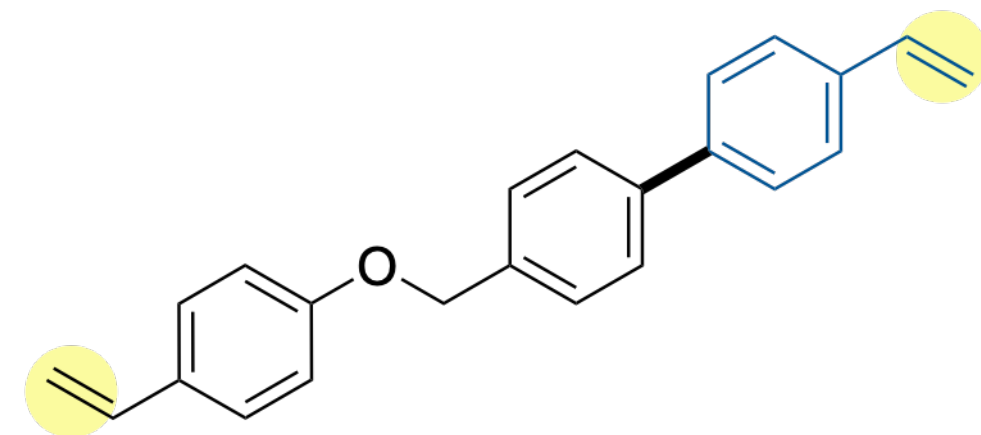

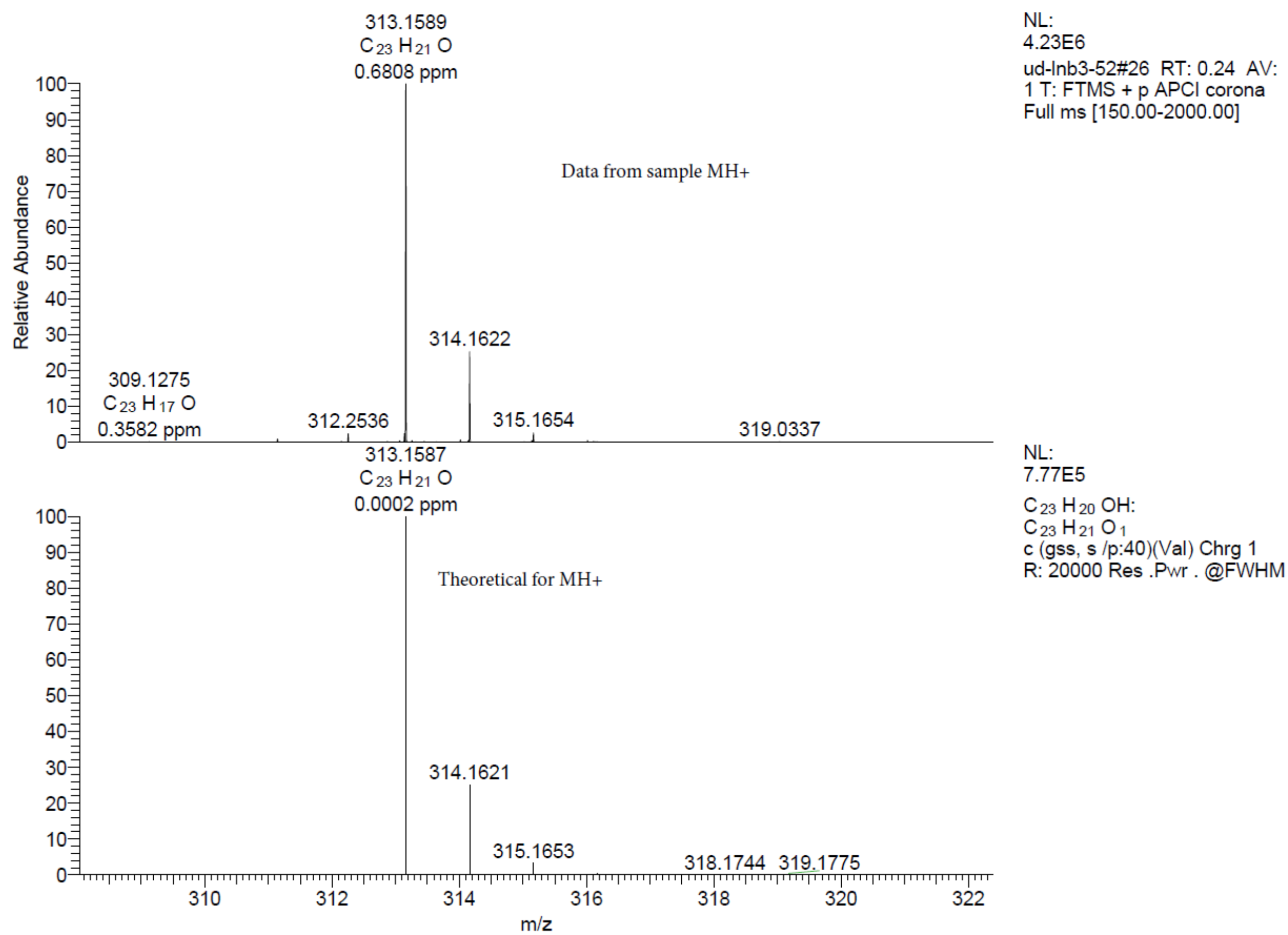

2,4-dichloro-4'-vinyl-1,1'-biphenyl (33)

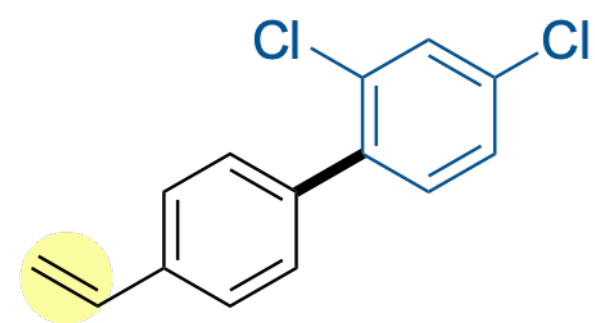

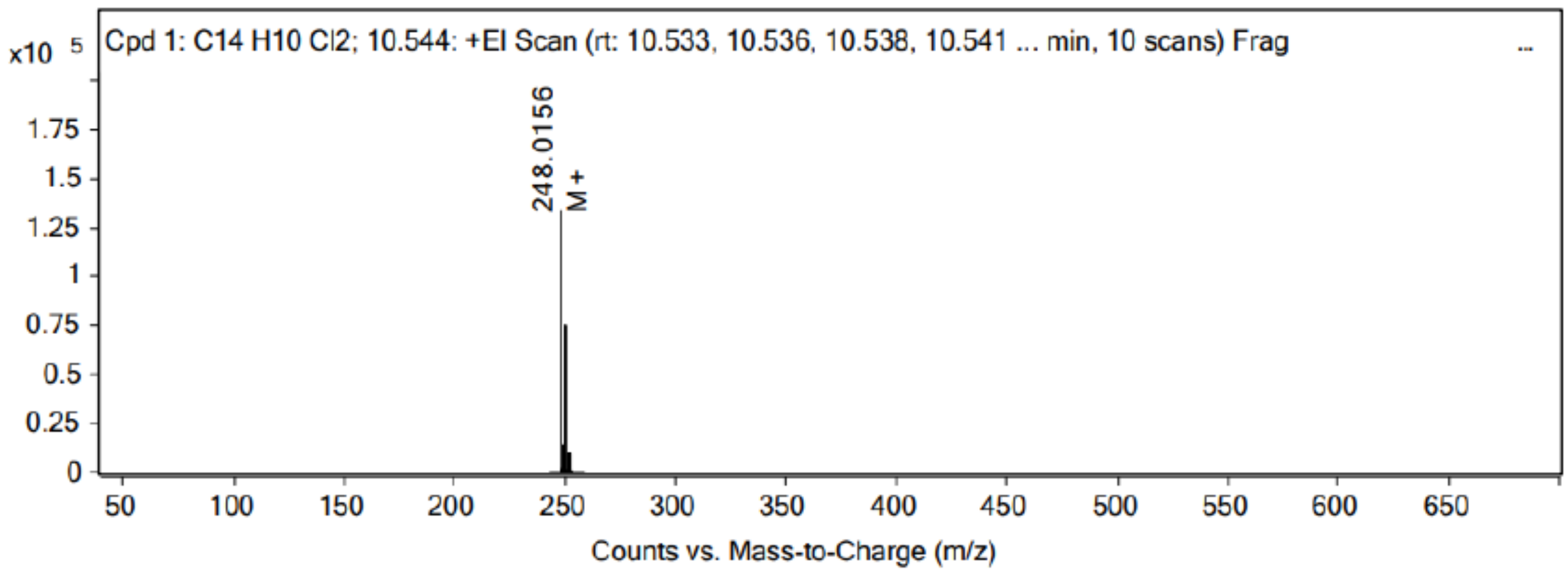

MS Zoomed Spectrum

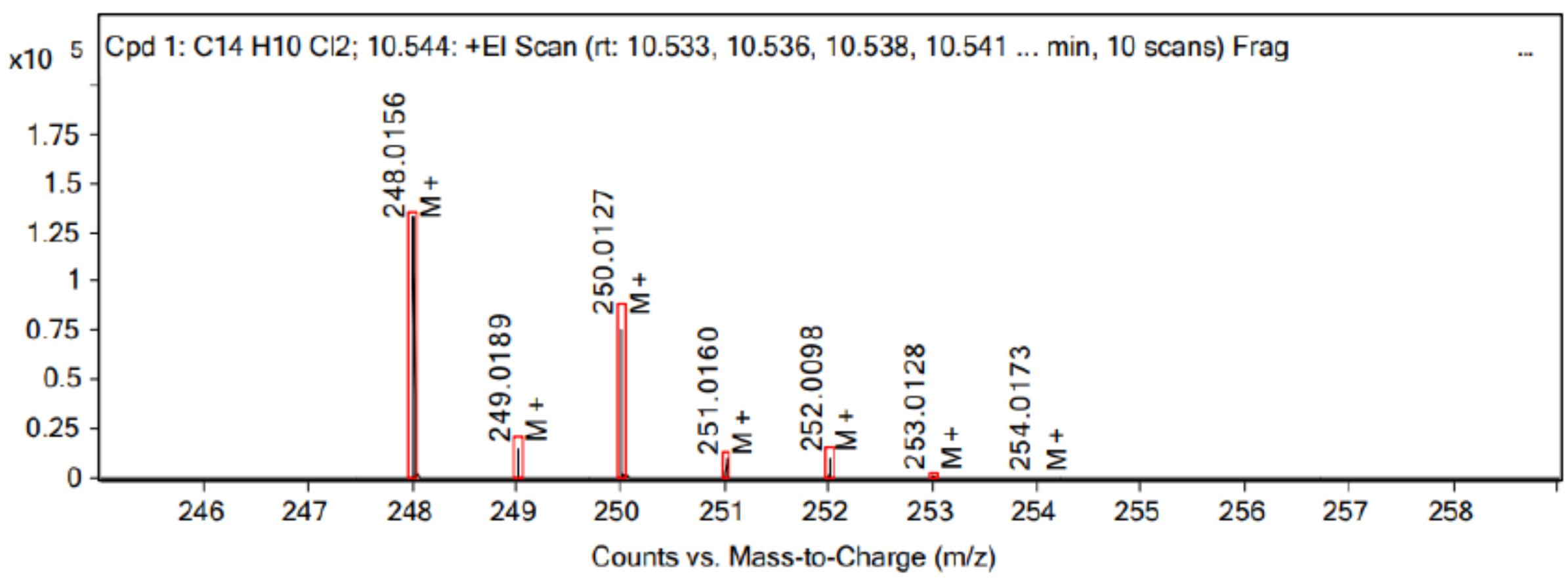

MS Spectrum Peak List

m/z	Calc *m/z*	Diff(ppm)	z	Abund	Ion
248.0156	248.0154	-0.89		135925.61	
248.0156	248.0154	-0.89	1	135963.88	M+
249.0189	249.0188	-0.43	1	15971.16	M+
250.0127	250.0126	-0.44	1	80834.55	M+
251.016	251.0159	-0.31	1	9787.5	M+
252.0098	252.0101	1.37	1	10166.43	M+
253.0128	253.0131	0.92	1	1594.87	M+
254.0173	254.0163	-3.71	1	140.19	M+

1-(4-(Naphthalen-1-yl)benzyl)-5-vinyl-1*H*-indole (34)

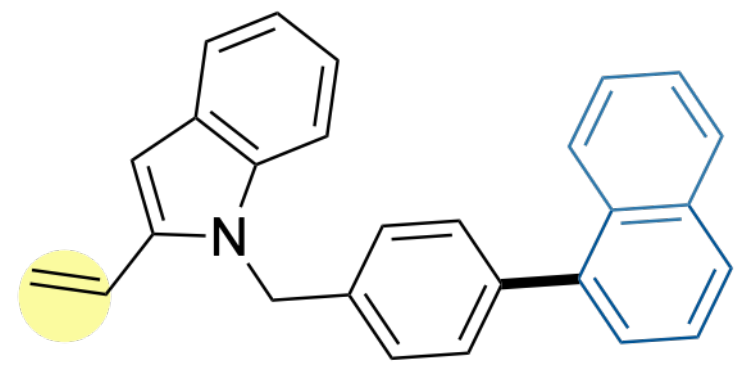

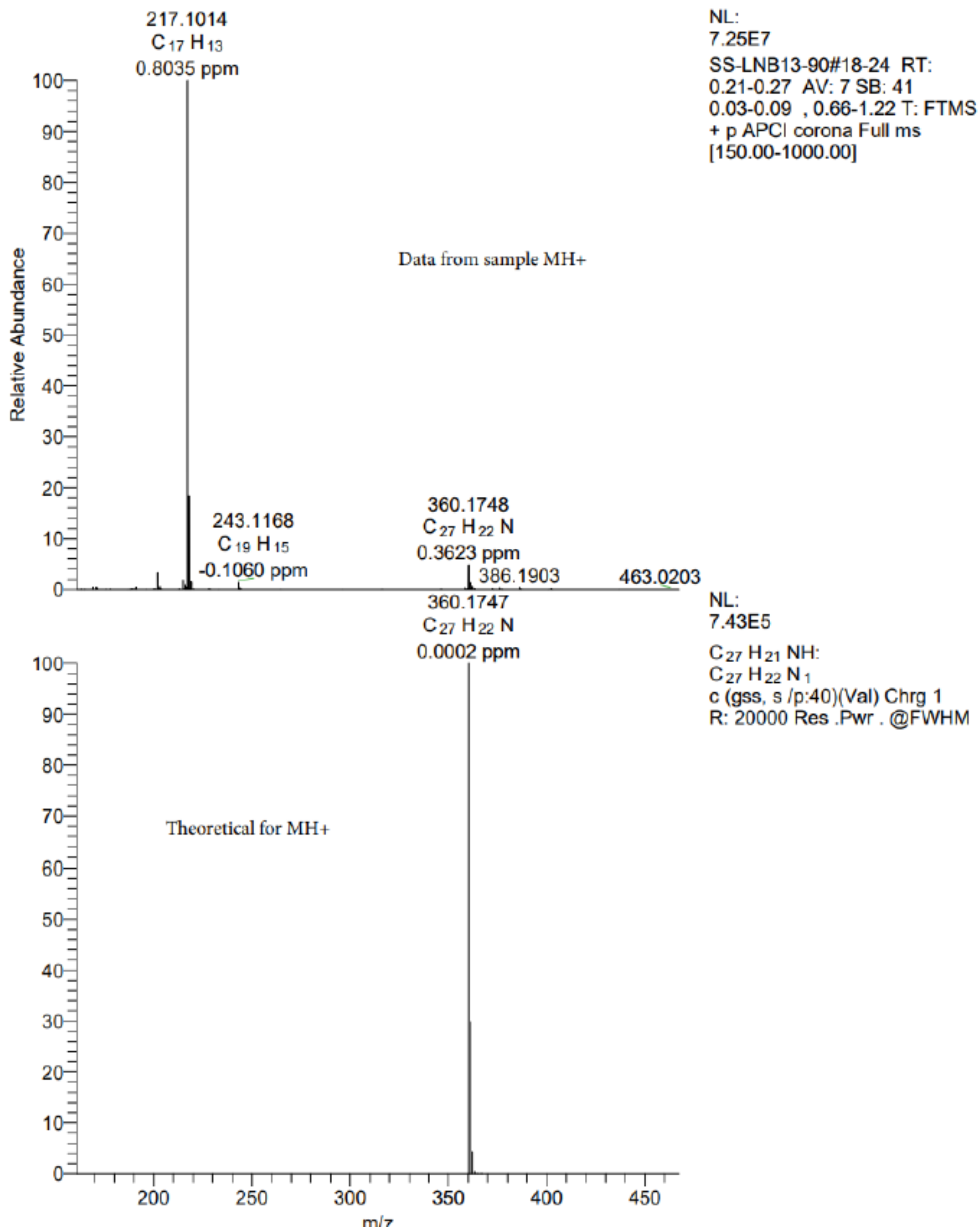

Supplement: Supplementary file 1 [file au6c00796_si_001.pdf]
